# Turning Enantiomeric Relationships into Diastereomeric Ones: Self-Resolving α-Ureidophosphonates and Their Organocatalytic Enantioselective Synthesis

**DOI:** 10.1021/jacs.2c10911

**Published:** 2022-12-14

**Authors:** Vanda Dašková, Damián Padín, Ben L. Feringa

**Affiliations:** Stratingh Institute for Chemistry, University of Groningen, Nijenborgh 4, 9747 AGGroningen, The Netherlands

## Abstract

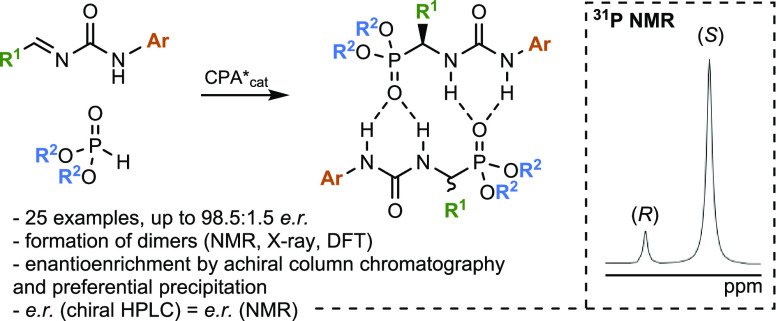

Controlling chiral recognition and chiral information transfer has major implications in areas ranging from drug design and asymmetric catalysis to supra- and macromolecular chemistry. Especially intriguing are phenomena associated with chiral self-recognition. The design of systems that show self-induced recognition of enantiomers, i.e., involving homochiral versus heterochiral dimers, is particularly challenging. Here, we report the chiral self-recognition of α-ureidophosphonates and its application as both a powerful analytical tool for enantiomeric ratio determination by NMR and as a convenient way to increase their enantiomeric purity by simple achiral column chromatography or fractional precipitation. A combination of NMR, X-ray, and DFT studies indicates that the formation of homo- and heterochiral dimers involving self-complementary intermolecular hydrogen bonds is responsible for their self-resolving properties. It is also shown that these often unnoticed chiral recognition phenomena can facilitate the stereochemical analysis during the development of new asymmetric transformations. As a proof of concept, the enantioselective organocatalytic hydrophosphonylation of alkylidene ureas toward self-resolving α-ureidophosphonates is presented, which also led us to the discovery of the largest family of self-resolving compounds reported to date.

## Introduction

Since the discovery of molecular chirality by Pasteur in the 19th century^1^ and the introduction of the tetrahedral carbon by van ’t Hoff^2^ and Le Bel,^3^ the chemical community has been fascinated by the myriad of phenomena associated with stereochemistry. The homochiral nature of the essential building blocks of biological systems, often denoted as “a signature of life,”^4^ shows the key role of chiral information transfer, which is far from being fully understood. Especially intriguing is chiral self-recognition of enantiomers, i.e., homochiral versus heterochiral dimer (and higher order aggregates) formation, governing phenomena ranging from conglomerate and racemate formation in crystallization^5^ to nonlinear effects in asymmetric (auto)catalysis.^6^ Among the most prominent examples are the attrition-enhanced deracemization^7^ and the chiral “sergeant and soldier” effect in supramolecular systems.^8^ However, predicting chiral recognition at the molecular level is still challenging, and transmission of chiral information along length scales is mainly based on serendipitous discovery.

**Figure 1:**
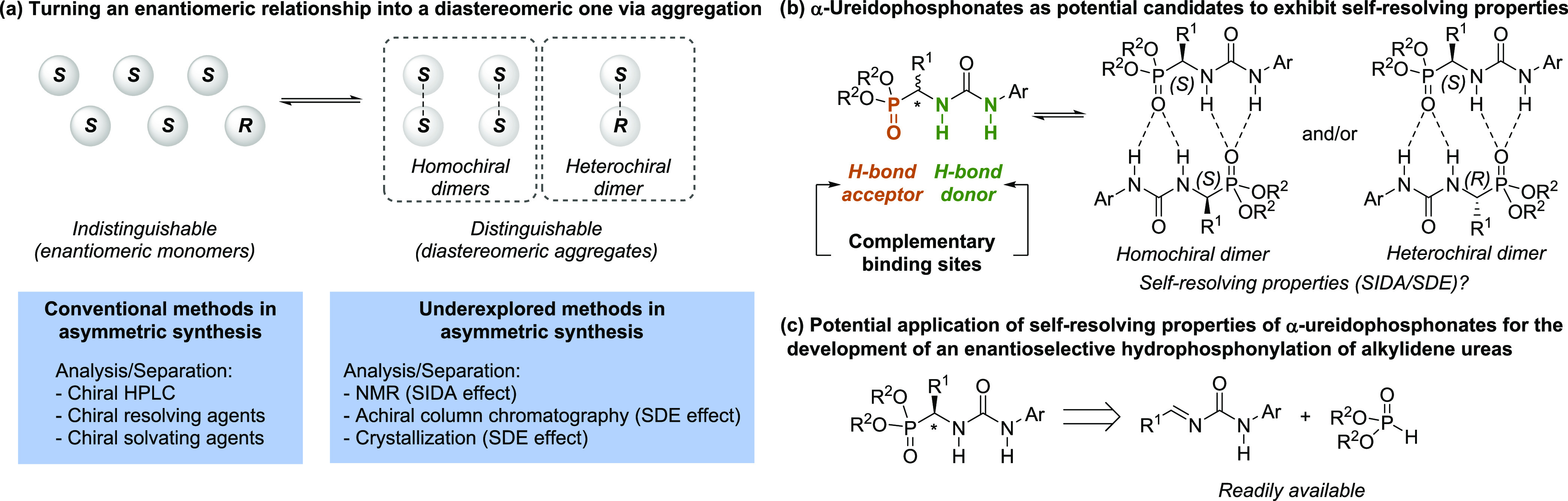
Concept of association of enantiomers (a) and its application to the development of an enantioselective synthesis of α-ureidophosphonates via hydrophosphonylation of alkylidene ureas (b and c).

A remarkable case of chiral self-recognition is the self-induced diastereomeric anisochronism effect^9^ in NMR (SIDA, also known as self-induced recognition of enantiomers). This rather unknown and often overlooked phenomenon arises from the spontaneous aggregation of certain chiral nonracemic molecules in solution, leading to diastereomeric homo- and heterochiral associates that exhibit different NMR signals (Figure 1a).^10^ Consequently, under ideal conditions,^11^ this effect enables the direct determination of the enantiomeric ratio in a scalemic mixture by simple NMR analysis.

Although it is difficult to predict whether a chiral compound will show the SIDA effect, certain families of compounds, including some chiral ureas,^12^ P(V)-based compounds,^13^ carboxylic acids,^14^ amides,^12,15^ and alcohols,^10,16^ have been found to exhibit this phenomenon. All of these chiral compounds display an evident structural feature, the presence of self-complementary hydrogen-bond acceptor and donor groups that lead to the formation of aggregates in solution. Furthermore, the presence of a stereogenic center next to these functionalities imposes a distinct three-dimensional (3D) arrangement of the homo- and heterochiral aggregates,^17^ giving rise to diastereomeric species with different physicochemical properties (NMR spectra, solubility, polarity). Additionally, these aggregates are also prone to exhibit spontaneous fractionation of enantiomers into enantiomerically enriched and depleted fractions in the absence of any chiral inductor (also denoted as self-disproportionation of enantiomers, SDE).^18^ Both effects have been recognized as potential sources of misinterpretation of chiral information, but they also provide unique opportunities for the convenient analysis of scalemic mixtures and, in some cases, ready access to enantiopure compounds.^10,19^ Clearly, exploring these effects might also greatly impact the development of new enantioselective transformations, as they can significantly accelerate the screening of reaction conditions and the analysis of the scope and provide an expedient way to increase the enantiopurity of the compounds.^15k^ Nevertheless, to the best of our knowledge, these effects have never been exploited systematically in reaction development. Furthermore, addressing chiral self-recognition by design might also be highly valuable to rationally approach, e.g., chiral communication, asymmetric catalysis, supramolecular chirality, and the fundamental understanding of the origin of homochirality.

Based on our long-standing efforts on antipodal and nonlinear effects,^20^ chiral auto-amplification,^21^ and supramolecular chirality^22^ we have taken up the challenge to design molecules that would show chiral self-recognition. Encouraged by our previous findings on the ability of ureas to bind to phosphonates and phosphates through moderately strong inter- or intramolecular hydrogen bonds,^23^ we envisioned a system based on multiple self-complementary intermolecular hydrogen bonds combining phosphonate and urea moieties in chiral α-ureidophosphonates, potentially leading to a self-induced recognition of enantiomers (Figure 1b). Additionally, this chiral self-recognition could be used as an analytical tool to rapidly assess the stereochemical outcome of an asymmetric route toward α-ureidophosphonates,^24^ showcasing the principle. In that case, it would significantly accelerate the screening of the reaction conditions, opening new avenues in asymmetric synthesis. To this end, we conceived an enantioselective hydrophosphonylation^25,26^ of readily available alkylidene ureas as a convenient platform to test our hypothesis (Figure 1c). Here, we report the discovery of an entirely new family of so far underexplored compounds, i.e., α-ureidophosphonates, that exhibit self-resolving properties (SIDA + SDE). In addition, we demonstrate that such self-resolving properties facilitated the optimization of the reaction conditions, the analysis of the scope, and the enantiomer purification of the final products.

## Results and Discussion

While the origin of the SIDA effect has been described in the literature,^10b,13b,15c^ a succinct explanation is provided first for the sake of clarity. In the simplest case, considering a mixture of two enantiomers, *R* and *S*, that tend to reversibly form dimers in solution, the following equilibria can be proposed:
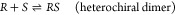
1
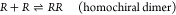
2
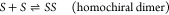
3In a scenario where binary associations occur under fast exchange conditions between monomeric and dimeric species in the NMR scale, two sets of peaks would appear in the NMR spectra as a result of the distinct time-averaged local environments for the homochiral and heterochiral associates. Specifically, one set of peaks corresponds to the weighted average of the three species where *R* enantiomer is present (*R* + *RR* + *RS*, 4), and another set of peaks corresponds to the weighted average of the three species where *S* enantiomer is present (*S* + *SS* + *RS*, 5)
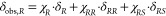
4
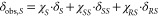
5where δ_obs,*R*_ and δ_obs,*S*_ are the averaged chemical shifts for each set of signals observed in the NMR spectra corresponding to the *R* and *S* enantiomers, respectively; δ_*R*_ and δ_*S*_ are the chemical shifts of the monomeric enantiomers; δ_*RR*_, δ_*SS*_, and δ_*RS*_ are the chemical shifts of the dimeric homo- and heterochiral species; χ_*R*_ and χ_*S*_ are the molar fractions of each monomeric enantiomer; and χ_*RR*_, χ_*SS*_, and χ_*RS*_ are the molar fractions of the homo- and heterochiral dimers. From the above equations, it becomes evident that a change in the enantiomeric ratio of a scalemic mixture will alter the position of the equilibria shown in 1 and 2 and, consequently, change the chemical shift of the two sets of peaks (4 and 5). As a result, racemic and enantiopure solutions of a chiral compound show different NMR spectra and, in the case of a scalemic mixture, where 50:50 < *e.r.* < 100:0, two sets of peaks are observed and integration of the signals directly provides the *e.r*.

Importantly, as previously pointed out by Harger,^13b^ if the exchange rate between the species in equilibria was slow, the position of the two sets of signals would be independent of the enantiomeric ratio, and their relative intensities would be equal to the ratio of diastereoisomers rather than the ratio of enantiomers.

### On the Formation of Self-Resolving Homo- and Heterochiral Aggregates Derived from α-Ureidophosphonates

**Scheme 1:**
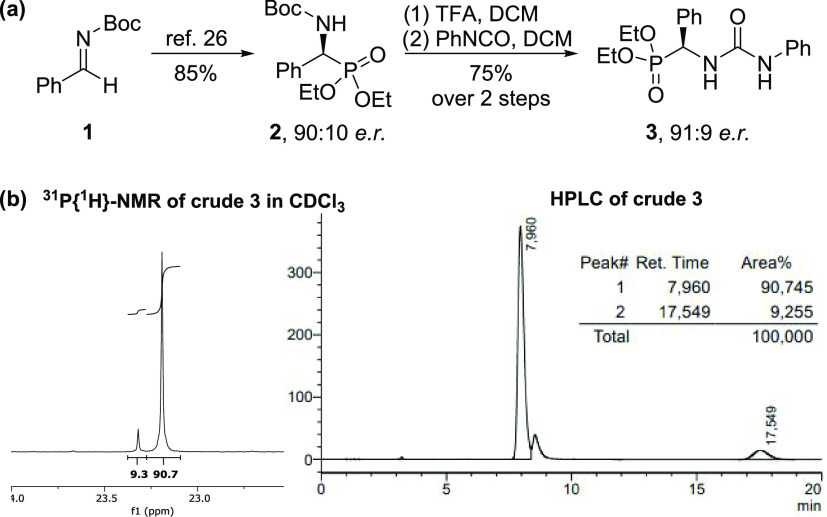
(a) Preliminary Access to Scalemic **3** and (b) Comparison of the ^31^P{^1^H}-NMR (Left) and Chiral HPLC Chromatogram (right) of Compound **3** Showcasing the SIDA Effect

To determine whether α-ureidophosphonates can form homo- and heterochiral aggregates, the preparation of a nonracemic α-ureidophosphonate was required. However, to the best of our knowledge, such compounds have never been prepared in an enantioenriched form. Consequently, we conceived a preliminary multistep sequence to access to enantioenriched α-ureidophosphonate **3** (Scheme 1a), which involved an enantioselective hydrophosphonylation of Boc-protected imine **1**,^27^ followed by deprotection of the α-amino phosphonate ester **2** and coupling with phenyl isocyanate. Upon analysis by ^1^H-NMR and ^31^P{^1^H}-NMR of the crude mixture of **3** in CDCl_3_, we clearly observed the formation of two sets of peaks whose integration matched the expected enantiomeric ratio (90:10 of precursor **2** vs 91:9 of α-ureidophosphonate **3**). Further analysis by chiral HPLC confirmed that the enantiomeric ratio of **3** was 91:9 (Scheme 1b). These results indicated that α-ureidophosphonates might be aggregating in solution and, indeed, show the SIDA effect.

**Figure 2:**
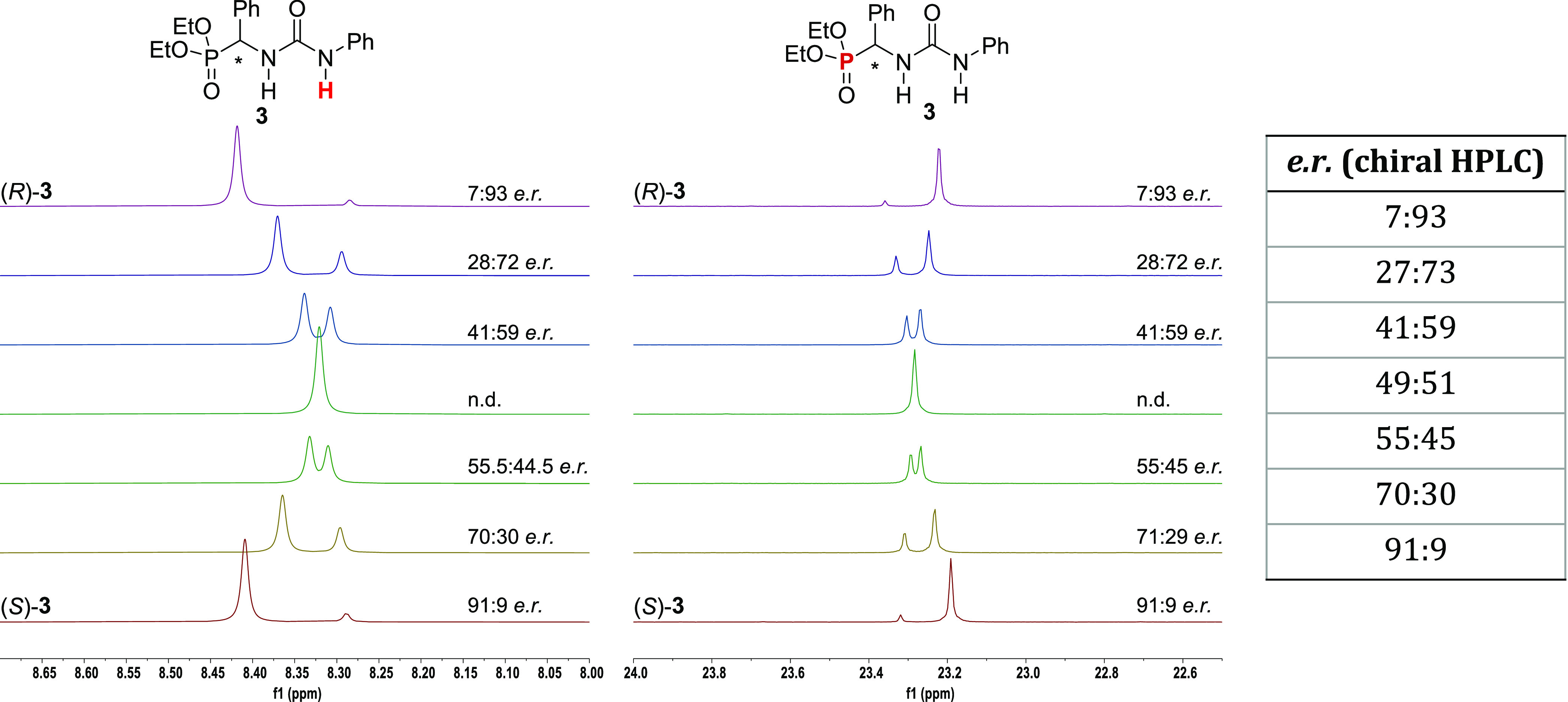
Comparison of the enantiomeric ratios of **3** determined by ^1^H-NMR (left, 400 MHz, 0.05 M, CDCl_3,_ 25 °C), ^31^P{^1^H}-NMR (middle, 162 MHz, 0.05 M, CDCl_3_, 25 °C), and chiral HPLC (right table). See the Supporting Information for details.

It is known that SIDA strongly depends on the dielectric constant of the medium, and solvents capable of disrupting intermolecular hydrogen-bonding interactions can lead to its disappearance.^10a^ Therefore, we performed a systematic study to disclose in which solvents compound **3** showed splitting of the NMR signals. While highly polar solvents such as DMSO-*d*_6_, MeOD, or DMF-*d*_7_ led to the disappearance of the SIDA effect, lower-polarity solvents (CDCl_3_, CD_2_Cl_2_, toluene-*d*_8_, acetone-*d*_6_) gave rise to two sets of signals both in ^1^H-NMR and ^31^P{^1^H}-NMR whose relative ratio matched the expected enantiomeric ratio (see the Supporting Information). ^31^P{^1^H}-NMR spectroscopy proved particularly suitable for the determination of the optical purity of compound **3** thanks to the excellent separation of the two sets of peaks for a wide range of enantiomeric ratios (Figure 2).

### Evidence for the Formation of Dimers

**Figure 3:**
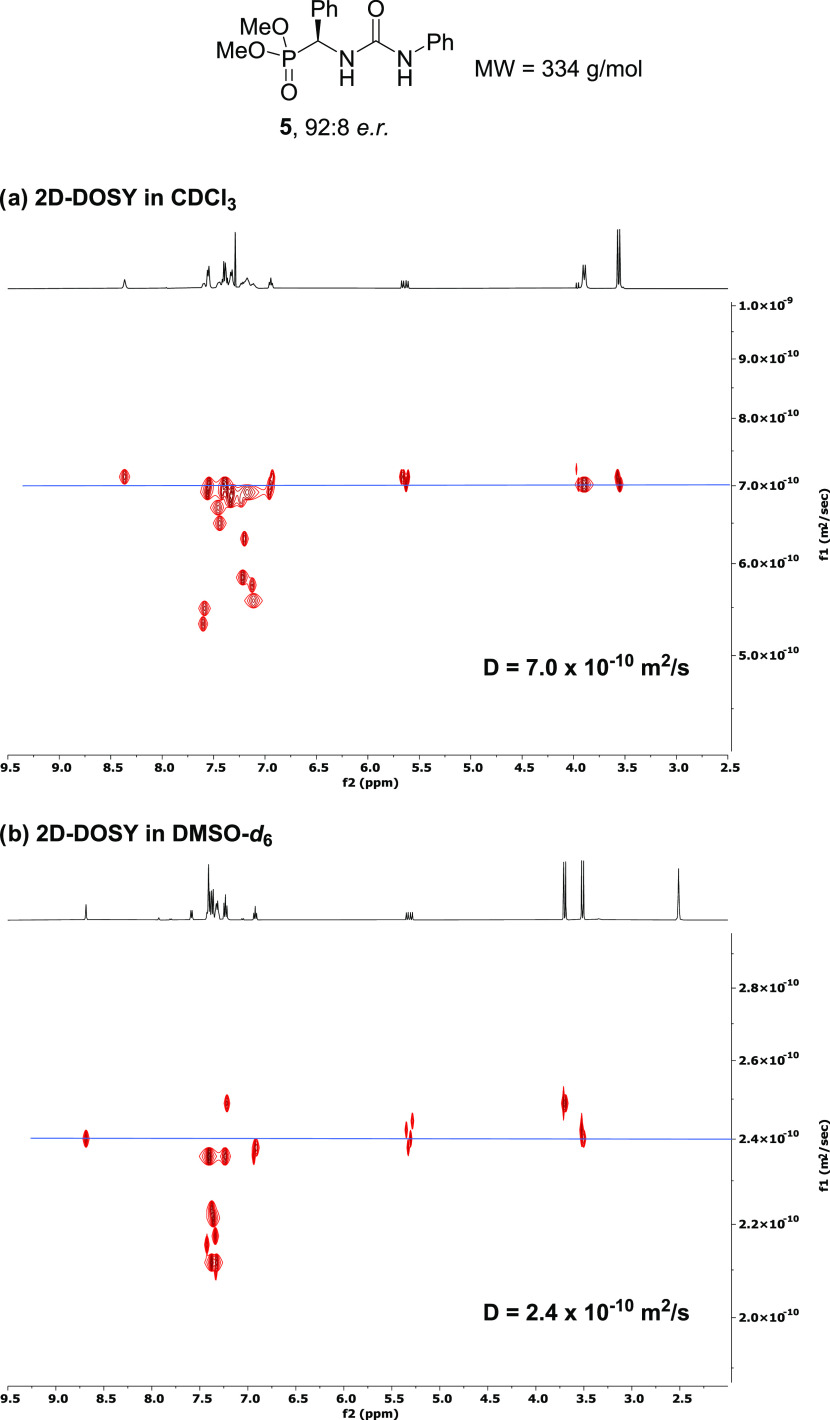
Two-dimensional (2D)-DOSY analysis of **5** in CDCl_3_ (a) and DMSO-*d*_6_ (b).

The nonequivalency of NMR spectra between enantiomers of **3** in low-polarity solvents pointed to the formation of diastereomeric aggregates. While the presence of self-complementary binding sites in α-ureidophosphonates suggested a dimeric structure of the aggregate^28,29^ (Figure 1b), a combined experimental and theoretical study was performed to confirm this assumption. First, diffusion-ordered spectroscopy (DOSY) measurements allowed us to calculate the diffusion coefficients of **5** in different solvents and, eventually, estimate the molecular weight of the aggregates.^30^ Thus, in CDCl_3_, compound **5** (92:8 *e.r.*) showed a diffusion coefficient of *D* = 7.0 × 10^–10^ m^2^/s, which corresponds to an estimated molecular weight of MW (measured, CDCl_3_) = 865 g/mol (Figure 3a). Given that the molecular weight of monomeric **5** is MW (calculated) = 334 g/mol, the estimation of 865 g/mol suggests the formation of a dimeric structure in CDCl_3_. In contrast, the measurement of the diffusion coefficient of **5** in DMSO-*d*_6_ gave a value of *D* = 2.4 × 10^–10^ m^2^/s, corresponding to an estimated molecular weight of MW (measured, DMSO-*d*_6_) = 390 g/mol, indicative of the formation of a monomeric species in this solvent (Figure 3b). Overall, this data is in excellent agreement with the observation of the SIDA effect in CDCl_3_, where aggregation is expected, and not in DMSO-*d*_6_.^31^

**Figure 4:**
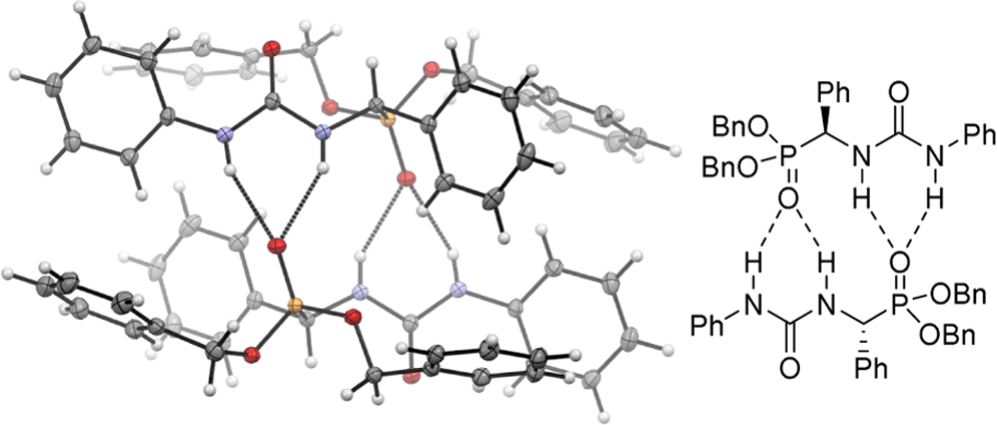
X-ray diffraction analysis of racemic **4**.

Single crystals suitable for X-ray diffraction analysis could be grown for the heterochiral aggregate (racemic) of the related compound **4** (Figure 4). Its structure in the solid state revealed a clear intermolecular association between two α-ureidophosphonates with opposite chirality in an antiparallel orientation (head-to-tail), where both N–H groups of the urea functionality of one molecule are engaged in a hydrogen-bonding interaction with the phosphonate group of its partner (N–H···O=P bond distances between 2.14 and 2.15 Å).

**Figure 5:**
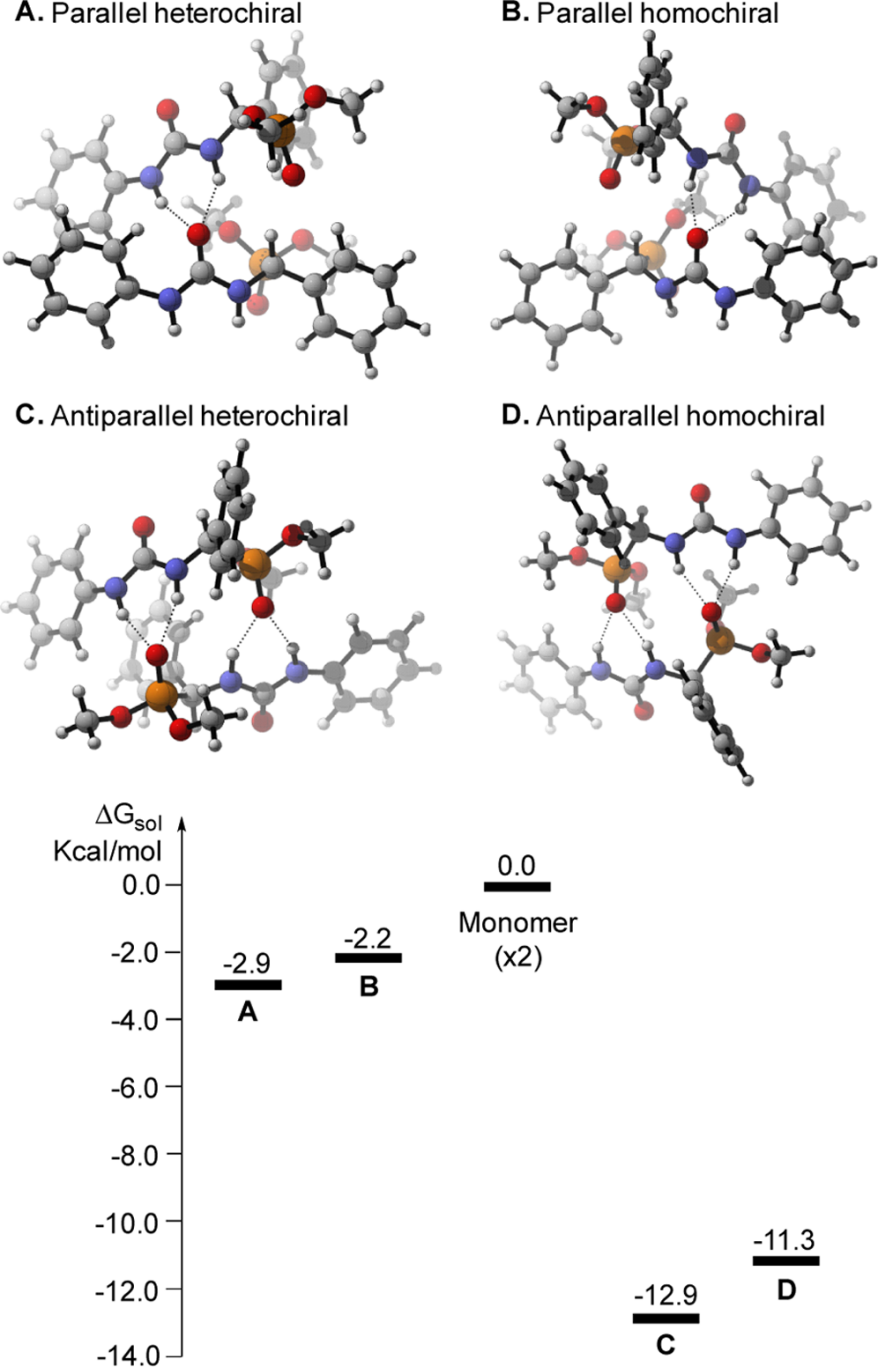
DFT-optimized structures of dimeric **5**. See the Supporting Information for details.

Additionally, DFT calculations at the ωb97XD/6–311++G(d,p)-SMD(PhMe)//ωb97XD/6–31+G(d,p) level^32^ indicated that such antiparallel disposition of α-ureidophosphonates is greatly preferred over other possible (parallel) dimeric structures (Figure 5). Remarkably, such an antiparallel arrangement stabilizes the dimeric structure up to 12.9 kcal/mol for the heterochiral aggregate and 11.3 kcal/mol for the homochiral associate, as compared to the monomeric form.

The analysis of the binding isotherms for compound **5**, both in racemic and enantioenriched form, by ^1^H-NMR titration^33^ allowed us to quantify the magnitude of the dimerization (see the Supporting Information). Both homochiral and heterochiral dimers showed association constants (*K*_a_) in the range of 10^3^ M^–1^, (3.3 ± 0.8) × 10^3^ M^–1^ for the former and (8.3 ± 0.2) × 10^3^ M^–1^ for the latter. While the SIDA effect is concentration-dependent, these relatively high association constants ensured the observation of the SIDA effect in a broad range of concentrations (0.2–0.01 M, in this study).

### SDE by Achiral Column Chromatography and Fractional Precipitation

The results shown above clearly suggest that α-ureidophosphonates form dimers in solution, leading to diastereomeric aggregates. As a result of this dimerization, the phenomenon of SDE by achiral column chromatography was also investigated with α-ureidophosphonate **5**, having an initial 92:8 *e.r.* of the crude material. A single chromatographic run using *n*-hexane/^*i*^PrOH 9:1 as eluent afforded **5** in 96% combined yield, where the first fraction provided a highly enantioenriched sample (98.5:1.5 *e.r.*) accounting for 31% yield (Table 1). The majority of the product (48%) eluted with the initial 92:8 *e.r*. The final fractions showed strong depletion of enantiopurity (58.5:41.5 and 52.5:47.5 *e.r.*) with 17% of the overall yield.^34^

**Table 1:**
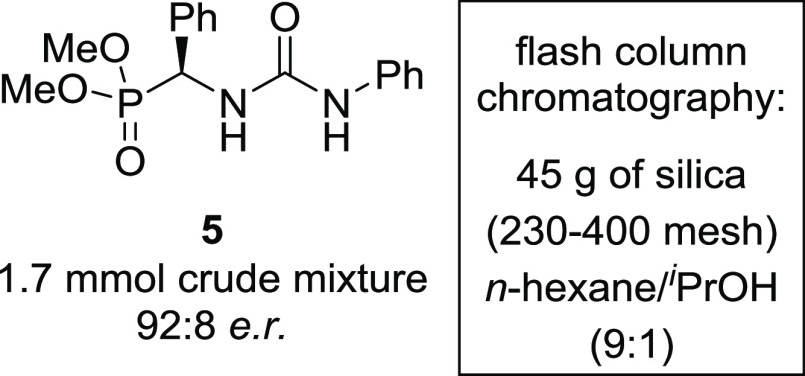
Achiral Phase Flash Column Chromatography of **5** with an Initial 92:8 *e.r*

fraction	*e.r.*	% yield
1	98.5:1.5	31
2	92:8	48
3	58.5:41.5	9
4	52.5:47.5	8

**Figure 6:**
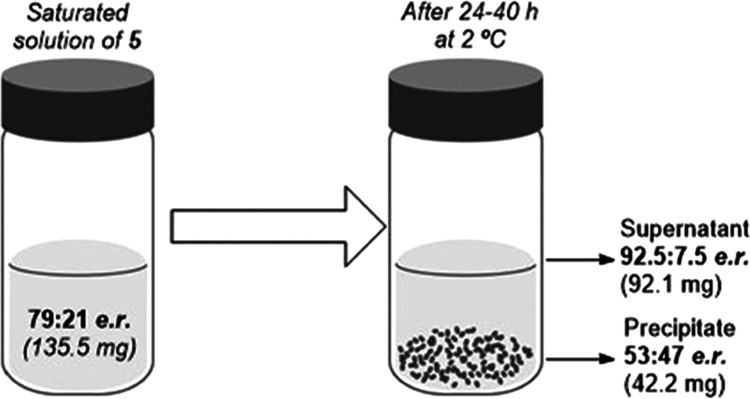
Spontaneous fractionation of enantiomers based on the different solubility of homo- and heterochiral dimers of **3**.

Alternatively, the spontaneous separation of enantiomers can also be achieved by harnessing the different solubility of the homo- and heterochiral dimers in organic solvents. Thus, when a saturated solution of compound **3** (135.5 mg) with an initial 79:21 *e.r.* in hexane/^*i*^PrOH 8:2 was cooled to 2 °C for 24–40 h, a white solid precipitated (Figure 6). The analysis of the supernatant solution and the solid fraction revealed a remarkable fractionation of enantiomers. While the precipitate consisted of nearly racemic **3** (42.2 mg, 53:47 *e.r.*), the supernatant contained compound **3** with significantly increased enantiopurity (92.1 mg, 92.5:7.5 *e.r.*). These experiments showcase the potential of SDE to obtain highly enantioenriched compounds by employing inexpensive purification methods.

### Application of the Self-Resolving Properties of α-Ureidophosphonates to the Development of an Enantioselective Hydrophosphonylation Reaction

At the onset of our studies, we selected the reaction comprising the readily available (*E*)-1-benzylidene-3-phenylurea^35^**6** and the commercially available diethyl phosphite as our model system. In the past decades, a wide range of catalysts have been used in enantioselective hydrophosphonylations of imines,^25,26^ although, to the best of our knowledge, a direct comparison of different catalyst classes is missing, and asymmetric hydrophosphonylation of alkylidene ureas is unprecedented. Motivated by addressing this gap, Lewis acidic metal complexes (**A**, **B**), cinchona alkaloids (**C**, **D**), thiourea-derived catalysts (**E**, **F**),^36^ chiral cyclopentadienyl-based Brønsted acid catalysts (**G**, **H**),^37^ and chiral phosphoric acids (**I**–**L**) were examined (Table 2). A model reaction between alkylidene urea **6** and diethyl phosphite was carried out in the presence of 10 mol % of a chiral catalyst. Al-salen complex **A** showed high catalytic activity; however, the hydrophosphonylation resulted in a nearly racemic product **3**. In comparison, Sharpless-catalyst **B** gave the product with better enantioselectivity (36:64 *e.r.*), albeit in lower conversion. Cinchona alkaloids **C** and **D** (entry 2), thiourea-based catalysts **E** and **F** (entry 3), and cyclopentadienyl Brønsted acids **G** and **H** (entry 4) did not prove to be suitable for our transformation, giving the product with low to good conversions and <55:45 *e.r*. Finally, using the simple (*S*)-BINOL-derived chiral phosphoric acid **I**, product **3** was obtained in a promising 82% conversion and 62.5:37.5 *e.r*. Encouraged by this result, we further explored the catalytic activity of other chiral phosphoric acids **J**–**L** (entries 5 and 6). Among them, the commercially available MacMillan TiPSY catalyst **L** afforded the desired product with 87:13 *e.r.* while maintaining a conversion of 79% (entry 6).

**Table 2:**
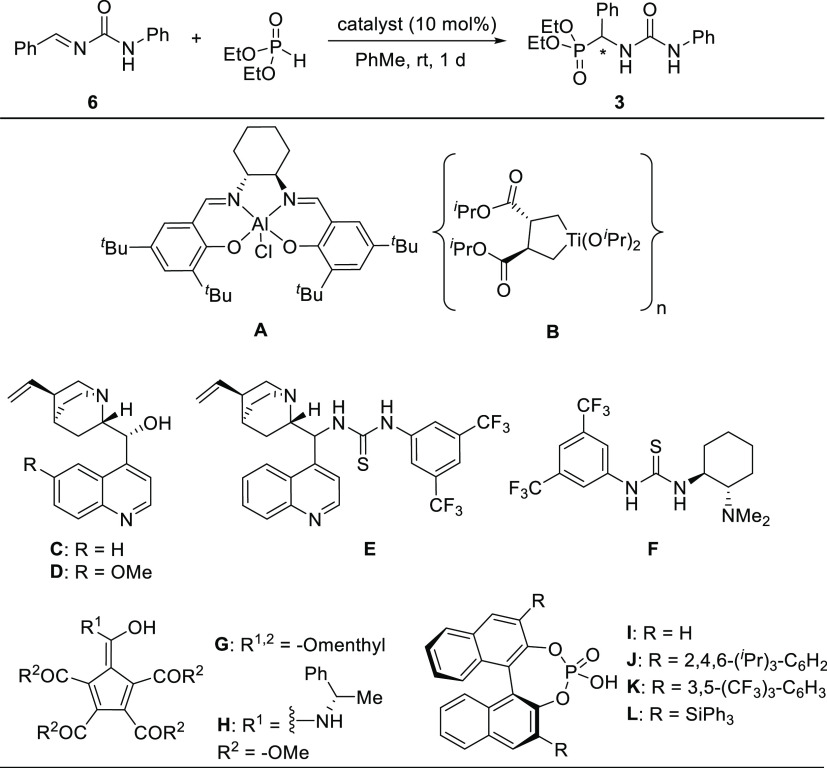
Catalyst Screening^a^

entry	catalyst	conversion (%)^b^	*e.r.*^c^
1	metal complexes **A**, **B**	88/26	49:51/36:64
2	*Cinchona* alkaloids **C**, **D**	31–43	<52.5:47.5
3	thioureas **E**, **F**	<10	<55:45
4	cyclopentadienyl-based Brønsted acids **G**, **H**	56–60	<52.5:47.5
5	phosphoric acids **I–K**	61–83	62.5:37.5–78:22
6	phosphoric acid **L**	79	87:13
7	phosphoric acid **L**^d^	88 (63)^e^	92.5:7.5

a General reaction conditions: alkylidene urea **6** (0.2 mmol), the indicated catalyst (0.02 mmol, 10 mol%), and diethyl phosphite (0.24 mmol) in PhMe (1 mL) at room temperature for 18 h.

b Determined by ^31^P{^1^H}-NMR.

c Enantiomeric ratios were determined by ^1^H-NMR/^31^P{^1^H}-NMR analysis, benefiting from the SIDA effect, and/or chiral HPLC analysis (see the Supporting Information for details).

d Under optimal conditions: Alkylidene urea **6** (0.2 mmol), **L** catalyst (0.01 mmol, 5 mol%), and diethyl phosphite (0.24 mmol) in deoxygenated PhMe (2 mL) at 30 °C for 48 h.

e Isolated yield in brackets.

Taking advantage of the SIDA effect enabling efficient reaction analysis, we further conducted an extensive reaction optimization, including an investigation of the solvent effect, temperature, catalyst loading, stoichiometry, and concentration (see the Supporting Information for details). We found that the best results were obtained using 1.2 equiv of diethyl phosphite and 5 mol % of TiPSY catalyst **L** in deoxygenated toluene (0.1 m) at 25 °C. Under these conditions, compound **3** was obtained in 63% isolated yield and 92.5:7.5 *e.r*. (entry 7).

**Scheme 2:**
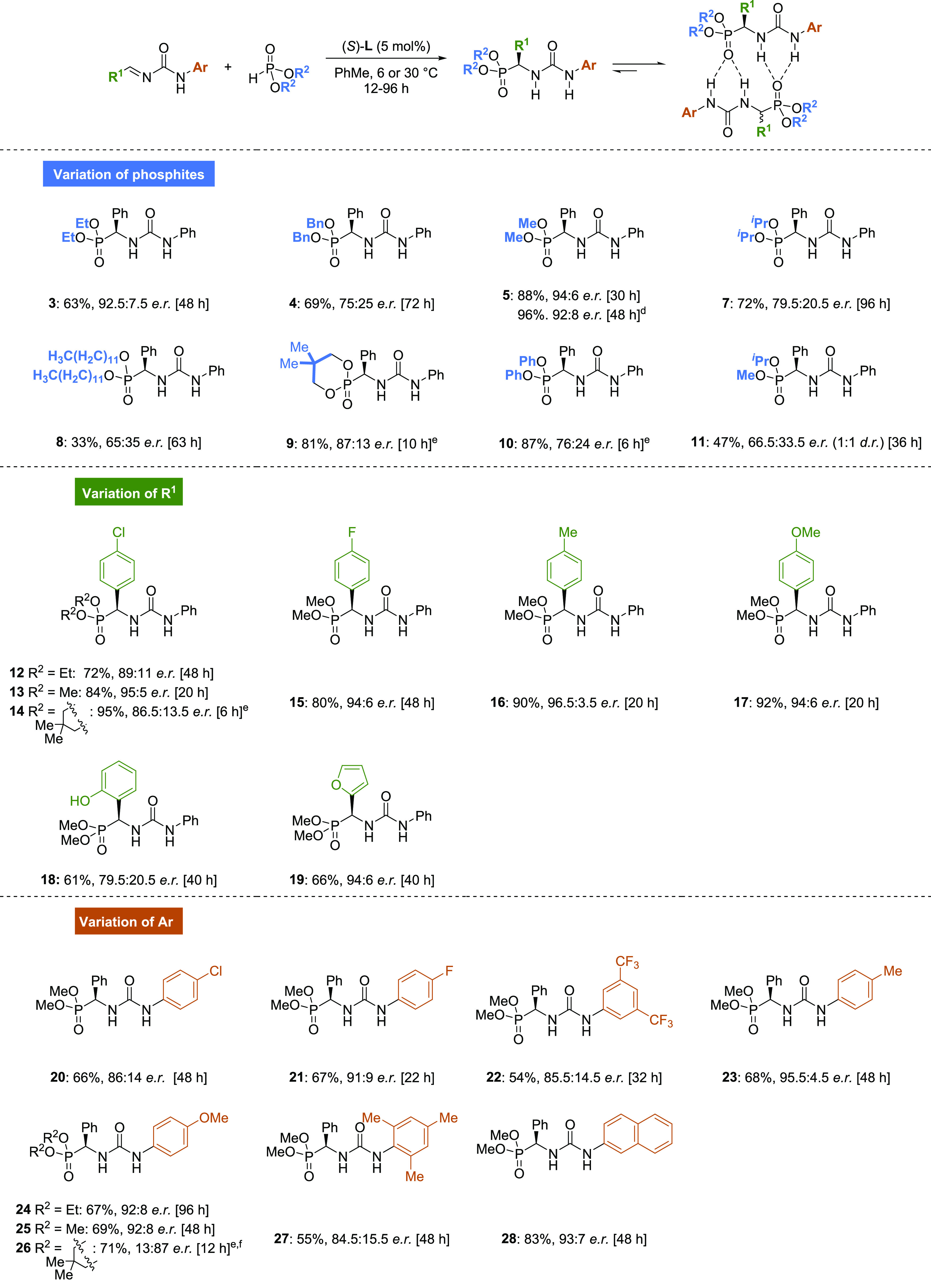
Scope of the Enantioselective Hydrophosphonylation of Alkylidene Ureas with Phosphites,, General reaction conditions: alkylidene urea (0.2 mmol), the corresponding alkyl/aryl phosphite (0.24 mmol), and catalyst (*S*)-**L** (0.01 mmol, 5 mol %) in deoxygenated PhMe (2 mL) at 30 °C for the indicated time. Yields of isolated products are given. Enantiomeric ratios were determined by ^1^H-NMR/^31^P{^1^H}-NMR analysis, benefiting from the SIDA effect, and chiral HPLC analysis for selected examples as controls (see the Supporting Information for details). Performed in a 2 mmol scale. Reaction performed at 6 °C. Using (*R*)-**L** as the catalyst.

Having identified the optimal reaction conditions, we explored the scope of phosphites and alkylidene ureas with various substitution patterns (Scheme 2).^38^ Initially, we tested a broad variety of phosphites in the hydrophosphonylation reaction of **6**. While diethyl phosphite and dimethyl phosphite afforded the corresponding α-ureidophosphonates **3** and **5**, respectively, in good yields and enantioselectivities, other bulkier phosphites led to the desired products with lower stereoselectivities (**4**, **7**–**11**). Importantly, we observed that for the more reactive diphenyl and neopentylene phosphites, lower temperatures (6 °C) were required to achieve better stereocontrol (**9** and **10**, 87:13 *e.r.* and 76:24 *e.r.*, respectively). Remarkably, the hydrophosphonylation reaction using dimethyl phosphite could be performed in a 2 mmol scale using 2 mol% of catalyst **L**, resulting in α-ureidophosphonate **5** in 96% isolated yield and 92:8 *e.r*.

Next, we analyzed the variations on the alkylidene urea backbone by introducing both electron-donating and electron-withdrawing groups in *ortho*-, *meta*-, or *para*-positions. Substitution in *R*^1^ was well tolerated, and α-ureidophosphonates **12**–**18** were obtained in good to excellent yields and enantiomeric ratios ranging from 79.5:20.5 to 96.5:3.5. We were pleased to observe that the reaction tolerated not only electron-poor (**12**–**15**) and electron-rich aromatic groups (**16**–**19**) but also the presence an unprotected phenol (**18**) and a heteroaromatic group (**19**).

The substitution effect was more pronounced in the case of variation on the Ar group. The introduction of electron-withdrawing substituents led to a slight reduction of the enantioselectivity. Thus, products **20** and **21**, bearing halogen groups in the *para*-position, and **22**, possessing two trifluoromethyl groups in *meta*-positions, were obtained in moderate yields and enantioselectivities. In contrast, higher isolated yields and enantioselectivities were obtained for α-ureidophosphonates bearing electron-rich aryl rings (**23**–**28**). An exception to this observation was noted for α-ureidophosphonate **27** having a bulky mesitylene group. We reasoned that such bulky substituent might lead to an unfavorable conformation of the starting alkylidene urea, as the urea group and mesitylene moiety cannot adopt a coplanar conformation, giving rise to a diminished reactivity and stereoselectivity.

It is important to remark that all α-ureidophosphonates shown in Scheme 2 form dimers in low-polarity solvents, which enabled the facile analysis of their enantiomeric purity by simple NMR techniques, as shown earlier for compound **3**. The analysis of the scope of the hydrophosphonylation reaction provided us with a library of up to 25 examples of compounds exhibiting chiral self-recognition properties (SIDA and SDE), which, to the best of our knowledge, represents the largest family of self-resolving compounds reported to date.

## Conclusions

To conclude, we have described an entirely new family of compounds, α-ureidophosphonates, that show self-resolving properties, namely, their enantiomeric purity can be directly determined by simple NMR techniques (SIDA effect) and their optical purity can be easily increased by inexpensive physicochemical techniques (achiral column chromatography and fractional precipitation). A combined experimental and computational work enabled us to determine that the formation of stable homo- and heterochiral dimers anchored through multiple intermolecular hydrogen bonds between the urea moieties and phosphonate groups is, ultimately, responsible for their self-resolving properties. Moreover, such self-resolving properties were systematically applied to the development of their first enantioselective synthesis via an unprecedented organocatalytic hydrophosphonylation of alkylidene ureas. Remarkably, the hydrophosphonylation reaction proved to be general and provided the desired α-ureidophosphonates in good yields and good enantiomeric excesses.

The fact that aggregation of enantiomers can transform an enantiomeric relationship into a diastereomeric one has important implications in the development of new asymmetric transformations and chiral supramolecular systems. Its potential to significantly simplify reaction analysis and be explored in phenomena ranging from chiral recognition to self-assembly and control of dynamic molecular systems might provide ample opportunities in future molecular design.

## Supporting Information Available

Experimental procedures, characterization of new compounds, computational details, NMR experiments, and chromatograms (PDF)

## Abbreviations

SIDA: self-induced diastereomeric anisochronism
SDE: self-disproportionation of enantiomers
DOSY: diffusion-ordered spectroscopy
