# Supplementary material for: Turning Enantiomeric Relationships into Diastereomeric Ones: Self-Resolving α-Ureidophosphonates and Their Organocatalytic Enantioselective Synthesis
Source: J Am Chem Soc. 2022 Dec 14;144(51):23603–13. doi: 10.1021/jacs.2c10911 (PMC9801384; doi:10.1021/jacs.2c10911)
Supplement: Supplementary file 1 — ja2c10911_si_001.pdf [file ja2c10911_si_001.pdf]

Supporting Information

Turning Enantiomeric Relationships into Diastereomeric Ones: Self-resolving α-Ureidophosphonates and their Organocatalytic Enantioselective Synthesis

Vanda Dašková,[‡] Damián Padín, [‡] Ben L. Feringa*

Stratingh Institute for Chemistry, University of Groningen, Nijenborgh 4, 9747 AG Groningen, The Netherlands

Corresponding author e-mail: b.l.feringa@rug.nl

SUPPORTING INFORMATION **1**

1. GENERAL EXPERIMENTAL PROCEDURES **3**

2. COMPOUNDS PREPARED FOLLOWING LITERATURE PROCEDURES **4**

3. STUDIES ON α-UREIDOPHOSPHONATE DIMERIZATION **6**

3.1. Preliminary multi-step access to α-ureidophosphonate 3 6
3.2. Studies on the SIDA effect. NMR spectra of 3 in different solvents. 7
3.3. Studies on the SIDA effect. NMR spectra of 3 with varying enantiomeric ratios. Comparison with chiral HPLC 15
3.4. 2D-DOSY NMR of 5 in $CDCl_3$ and DMSO-d_6 17
3.5. Determination of association constants 20

4. OPTIMIZATION OF THE ENANTIOSELECTIVE HYDROPHOSPHONYLATION OF ARYLIDENE UREAS **22**

5. DETERMINATION OF THE ABSOLUTE CONFIGURATION OF 3 .... **26**

6. INCREASING OPTICAL PURITY BY SELF-DISPROPORTIONATION OF ENANTIOMERS (SDE) **27**

6.1. Achiral column chromatography 27
6.2. Preferential dissolution of the homochiral dimer 28

7. SYNTHETIC PROCEDURES AND SPECTRAL DATA **29**

7.1. Synthesis and characterization of α-ureidosulfones 29
General method A for the preparation of α-ureidosulfones *29*
7.2. Synthesis and characterization of (hetero)arylideneureas 35
General method B for the preparation of (hetero)arylideneureas *35*
7.3. Synthesis and characterization of α-ureidophosphonates 40
General procedure C for the enantioselective hydrophosphonylation of (hetero)arylidene ureas *40*
General procedure D for the 2 mmol-scale preparation of 5 .... *40*
7.4. Unsuccessful arylidene ureas 55

8. X-RAY CRYSTALLOGRAPHIC ANALYSIS OF 4 .... **56**

9. COMPUTATIONAL DATA **61**

10. NMR SPECTRA **76**

11. *e.r.* DETERMINATION BY NMR AND/OR HPLC **143**

1. General experimental procedures

Unless otherwise specified, all reagents were obtained from commercial sources and used without further purification. Diethyl phosphite, diisopropyl phosphite and dimethyl phosphite were purified by distillation and stored under inert atmosphere. Prior to use, cesium carbonate (Cs_2CO_3) was dried under high vacuum at 70 °C overnight and stored under inert atmosphere. Dry solvents were obtained using a MBraun SPS 800 system and stored under N_2. For the hydrophosphonylation reactions, dry toluene was degassed through the freeze-pump-thaw method (4-6 cycles) and stored in a glovebox.

All reactions under N_2 atmosphere were carried out in oven-dried or heat gun-dried glassware with magnetic stirring. When specified, reactions were monitored by analytical thin layer chromatography on silica-coated aluminum plates (silica gel 60 F254 Merck) and components were visualized by UV light and $KMnO_4$ staining (1.5 g $KMnO_4$, 10 g K_2CO_3, 1.25 mL 10% NaOH, 200 mL H_2O). Flash column chromatography was performed on silica gel 60 (Merck, 230-400 mesh). Celite® 521 was used as filtering agent.

^{1}H-NMR, ^{13}C-NMR, ^{31}P{^{1}H}-NMR and ^{19}F-NMR experiments were carried out using Varian AMX400, Varian Oxford AS 500 MHz and Bruker Innova 600 MHz spectrometers. Chemical shift values are reported in ppm with the residual solvent resonances as the internal standards. Coupling constants (J) are given in Hertz (Hz). Multiplicities are reported as follows: s = singlet, d = doublet, t = triplet, q = quartet, p = pentet, m = multiplet or as a combination of them.

Reactions at 6 ºC were performed in a cold room. For the sake of reproducibility, hydrophosphonylation reactions at 30 ºC were performed using an oil bath. High Resolution Mass spectrometry (HRMS) analysis was carried out using a LTQ Orbitrap XL (ESI+, ESI-).

Optical rotations were determined with a SCHMIDT + HAENSCH Polartronic MH8 polarimeter at 589 nm and 20 °C. Data are reported as follows: $[\alpha]_{\lambda}^{temp}$(concentration [in g/100 mL], solvent). The determination of the enantiomeric ratios of α-ureidophosphonates was conveniently performed using their self-resolving properties (SIDA effect) by ^{1}H-NMR and ^{31}P{^{1}H}-NMR in $CDCl_3$ (*e.r.* of the dimer by NMR = *e.r.* of the monomer by HPLC). For selected compounds, enantiomeric ratios were also independently determined by HPLC analysis using a Shimadzu SPD M10AVP diode array detector using a Chiralpak columns with mixtures of HPLC-grade *n*-heptane and 2-propanol as the eluent and a column temperature of 40 °C or TharSFC Investigator II. Due to the self-disproportionation of enantiomers observed in α-ureidophosphonates, the enantiomeric ratios are reported before (crude mixture) and after purification by column chromatography. Additionally, due to the dimerization of the α-ureidophosphonates, specific rotation measurements were performed in DMSO or MeOH, where these compounds exist only as monomeric species, avoiding the Horeau effect.[1]

X-ray diffraction analysis was performed Bruker APEX-II CCD diffractometer. The crystal was kept at 100.0 K during data collection. See section 9 for further details.

[1] Polavarapu, P. L. *Org. Biomol. Chem.* **2020**, *18*, 6801-6806.

2. Compounds prepared following literature procedures

Thiourea-based **E**[2] and **F**,[3] cyclopentadiene-based **G** and **H**,[4] (*S*)-TiPSY[5] and (*R*)-TiPSY[6,5] were prepared following previously described procedures.

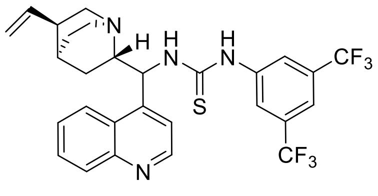

E

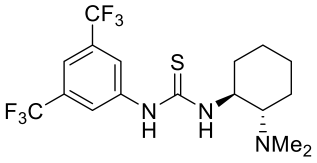

F

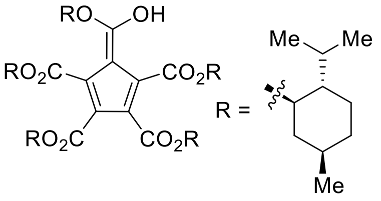

G

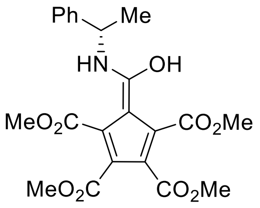

H

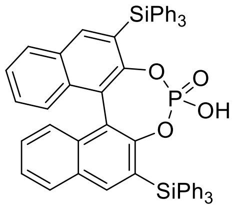

(*R*)/(*S*)-TiPSY

Didodecyl phosphite[7] and isopropyl methyl phosphite[8] were prepared using previously described procedures.

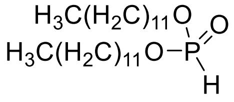

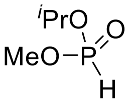

N-(2-Naphthyl)urea[9] was prepared using a previously described procedure.

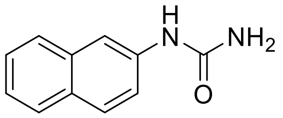

Phenyl (3,5-bis(trifluoromethyl)phenyl)carbamate was prepared following previously described method.[10]

---

[2] Tripathi, C. B.; Kayal, S.; Mukherjee, S. *Org. Lett.* **2012**, *14*, 3296-3299.
[3] Okino, T.; Hoashi, Y.; Furukawa, T.; Xu, X.; Takemoto, Y. *J. Am. Chem. Soc.* **2005**, *127*, 119-125.
[4] Gheewala, C. D.; Collins, B. E.; Lambert, T. H. *Science* **2016**, *351*, 961-965.
[5] Storer, R. I.; Carrera D. E.; Ni, Y.; MacMillan, D. W. C. *J. Am. Chem. Soc.* **2006**, *128*, 84-86.
[6] Keiji, M.; Takayuki, I.; Yoshitaka, A.; Tadashi, S.; Hisashi, Y. *Bull. Chem. Soc. Jpn.* **1988**, *61*, 2975-2976.
[7] Prepared using the procedure described in: Kuiper, J. M.; Hulst, R.; Engberts, J. B. F. N. *Synthesis* **2003**, *5*, 695-698. Characterization agreed with that reported in: Aitken, R. A.; Collet, C. J.; Mesher, S. T. E. *Synthesis* **2012**, *44*, 2515-2518.
[8] Guin, J.; Wang, Q.; van Gemmeren, M.; List, B. *Angew. Chem. Int. Ed.* **2015**, *54*, 355-358.
[9] Wang, C.-C.; Qu, Y.-L.; Liu, X.-H.; Ma, Z.-W.; Yang, B.; Liu, Z.-J.; Chen, X.-P., Chen, Y.-J. *J. Org. Chem.* **2021**, *86*, 3546–3554.
[10] Derasp, J. S.; Beauchemin, A. M. *ACS Catal.* **2019**, *9*, 8104-8109.

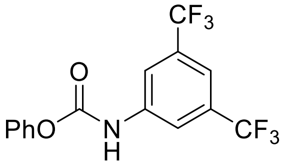

^{1}H NMR (400 MHz, $CDCl_3$) δ 7.96 (s, 2H), 7.61 (s, 1H), 7.47 – 7.37 (m, 2H), 7.31 – 7.26 (m, 1H), 7.25 (s broad, 1H), 7.22 – 7.17 (m, 2H). **^{19}F NMR** (376 MHz, $CDCl_3$) δ -63.14. **^{13}C NMR** (101 MHz, $CDCl_3$) δ 151.6, 150.2, 139.1, 132.8 (d, J = 33.5 Hz), 129.7, 126.4, 123.1 (q, J = 272.7 Hz), 121.6, 118.6, 117.4. **HRMS** (ESI) calculated for $C_{15}H_9F_6NO_2Na$: 372.04297, found: 372.04326.

1-(3,5-bis(trifluoromethyl)phenyl)urea was prepared was prepared following previously described method.[11]

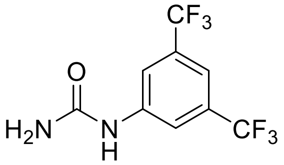

^{1}H NMR (600 MHz, DMSO-d_6) δ 9.29 (s, 1H), 8.08 (s, 2H), 7.48 (s, 1H), 6.21 (s, 2H); **^{13}C NMR** (151 MHz, DMSO-d_6) δ 155.7, 142.8, 130.6 (q, J = 32.6 Hz), 123.4 (q, J = 272.6 Hz), 117.3 (d, J = 4.3 Hz), 113.4 (p, J = 4.0 Hz); **^{19}F NMR** (565 MHz, DMSO-d_6) δ -61.95; **HRMS** (ESI+, m/z): calculated for $C_9H_5N_2F_6O^-$ $[M – H^+]^-$: 271.0312, found 271.0315.

[11] B. Thavonekham, Synthesis **1997**, *10*, 1189-1194.

3. Studies on α-ureidophosphonate dimerization

3.1. Preliminary multi-step access to α-ureidophosphonate 3

Preliminary studies regarding the dimerization of α-ureidophosphonates were carried out with α-ureidophosphonate **3**, prepared through the multi-step sequence depicted below.

Step 1) α-Amidosulfone **S1** was prepared following a literature procedure.[12]

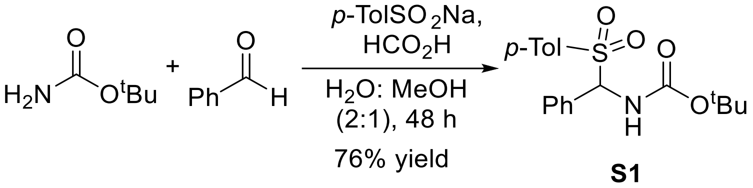

Step 2) Imine **1** was prepared following a literature procedure.[13]

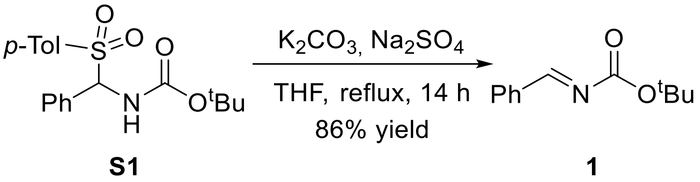

Step 3) α-Amidophosphonate **2** was prepared following a literature procedure.[14]

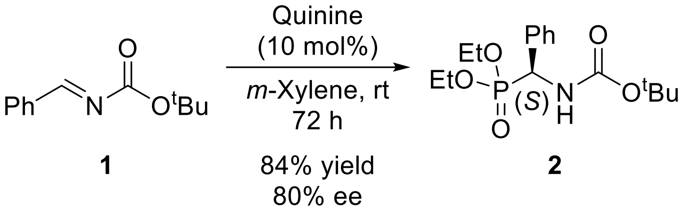

Step 4) α-Aminophosphonate **S2** was prepared following a literature procedure.[14,15] The crude product after work-up was used in the next step.

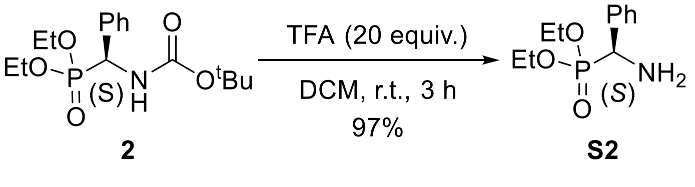

Step 5) To a stirred solution of α-aminophosphonate **S2** (70 mg, 0.288 mmol) in DCM (1.2 mL) at room temperature, phenylisocyanate (40 µL, 0.368 mmol, 1.28 equiv.) was added dropwise. The resulting colorless solution was stirred at room temperature overnight. Then, the reaction mixture was concentrated under reduced pressure and the crude product was purified by flash column chromatography on silica gel using hexane/^{i}PrOH 90:10 to afford α-ureidophosphonate **3** as a white solid (78 mg, 75% yield). Spectroscopic data of **3** obtained through this route proved

---

[12] E. Gomez-Bengoa, A. Linden, R. López, I. Múgica-Mendiola, M. Oiarbide, C. Palomo, *J. Am. Chem. Soc.* **2008**, *130*, 7955–7966 and references therein.

[13] Huang, L.; Wulff, W. D. *J. Am. Chem. Soc.* **2011**, *133*, 8892–8895.

[14] Pettersen, D.; Marcolini, M.; Bernardi, L.; Fini, F.; Herrera, R. P.; Sgarzani, V.; Ricci, A. *J. Org. Chem.* **2006**, *71*, 6269–6272.

[15] Smith, A. B. III.; Yager, K. M.; Taylor, C. M. *J. Am. Chem. Soc.* **1995**, *117*, 10879-10888.

to be identical to that obtained through the enantioselective hydrophosphonylation reaction presented in section 7.3.

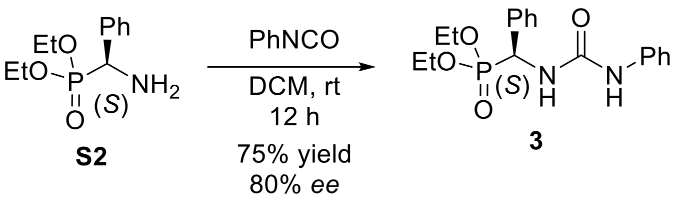

3.2. Studies on the SIDA effect. NMR spectra of 3 in different solvents.

A systematic study aiming to determine in which solvents compound **3** exhibits self-induced diastereomeric anisochronism (SIDA) effect was performed using **3** (3 mg, from a batch having a 85:15 *e.r.*) dissolved in the indicated deuterated solvent (0.6 mL). The ^{1}H-NMR and ^{31}P{^{1}H}-NMR spectra of **3** were recorded by 400 MHz NMR. The spectra obtained are shown below.

As explained in the manuscript, we could observe the splitting of the NMR signals in $CDCl_3$, CD_2Cl_2, PhMe-d_8, acetone-d_6 and CD_3CN, both in ^{1}H-NMR and, especially, in ^{31}P{^{1}H}-NMR. In all these solvents, the ratio between the major and the minor compound was 85:15, matching the *e.r.* observed by enantioselective HPLC analysis. In contrast, only one set of signals was observed in DMSO-d_6, MeOD-d_4 and DMF-d_7. NOTE: In ^{1}H-NMR, the signal used to determine the *e.r.* of the dimer (*e.r.* of monomer) is highlighted in red.

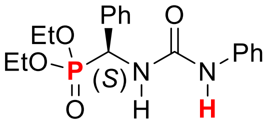

3

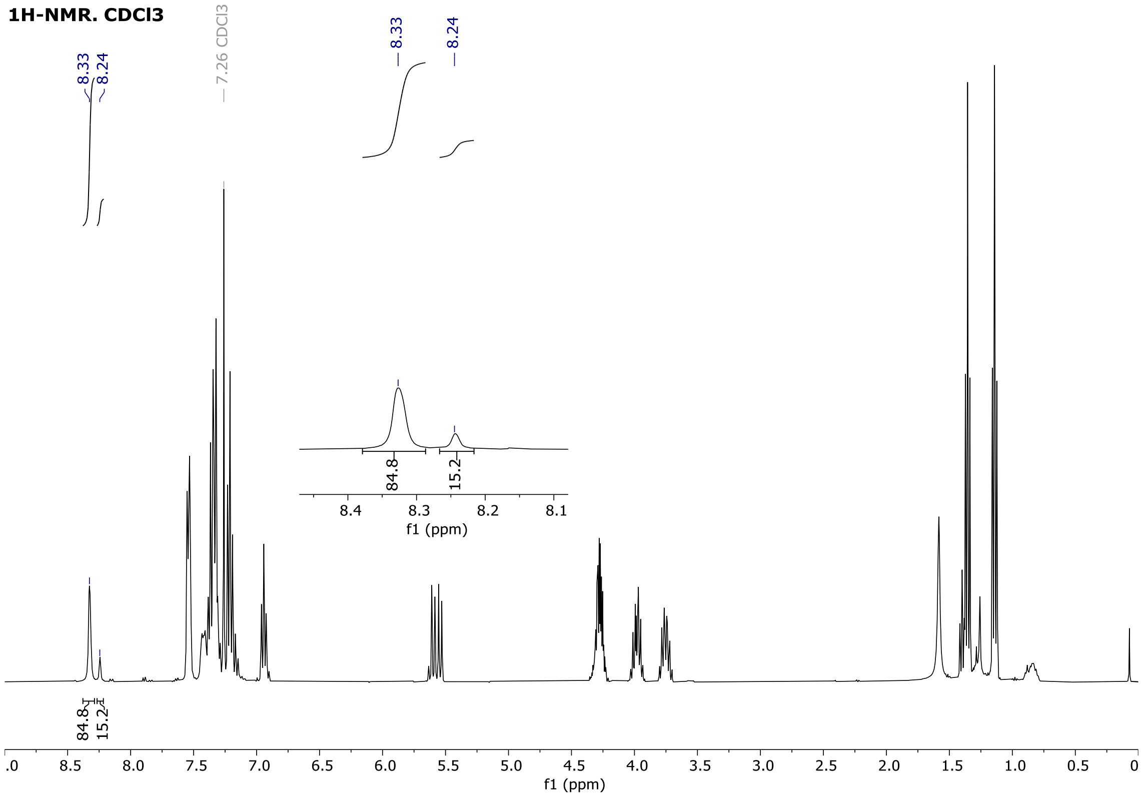

Figure S1 ^{1}H-NMR spectrum of **3** in CDCl₃.

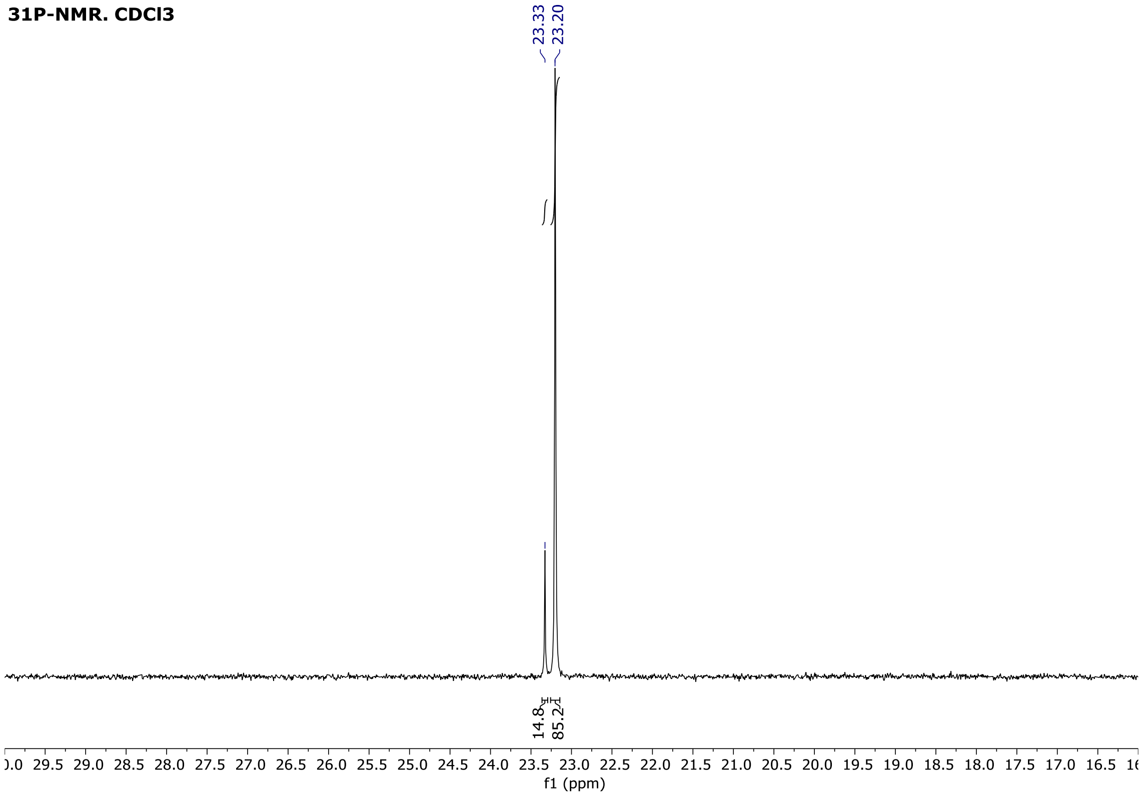

Figure S2 $^{31}P\{^{1}H\}$-NMR spectrum of **3** in $CDCl_3$.

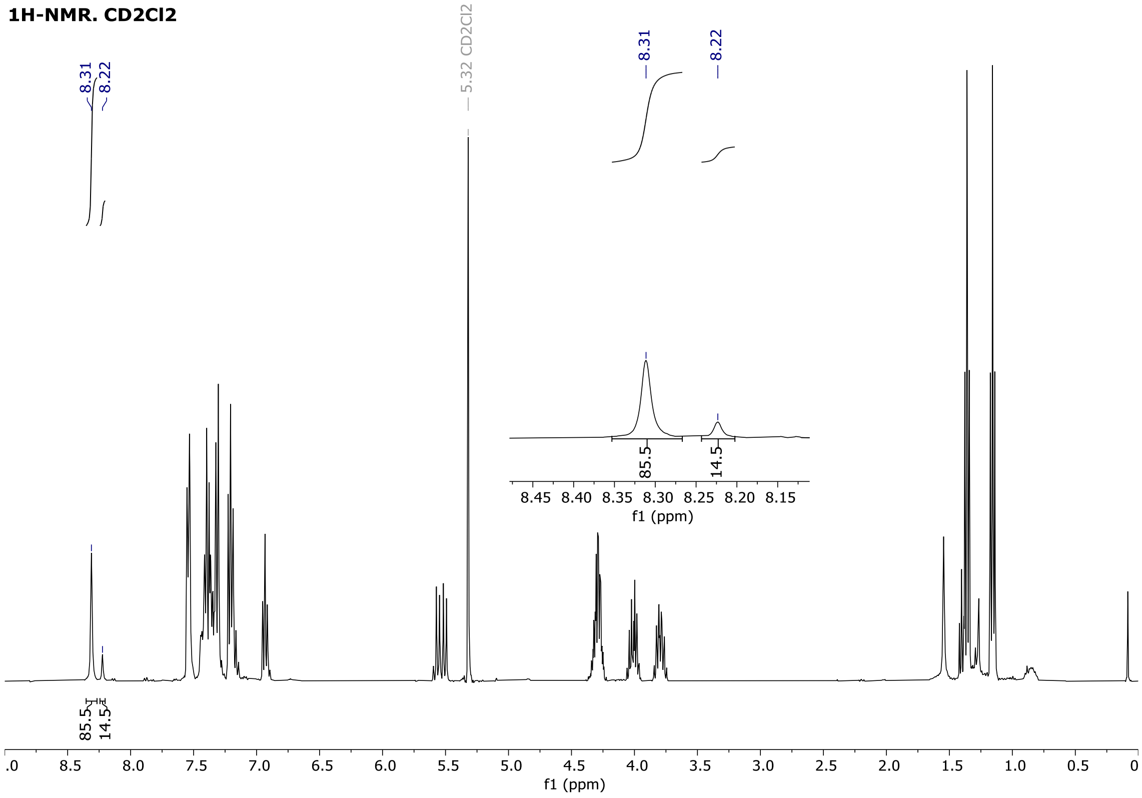

Figure S3 ^{1}H-NMR spectrum of **3** in CD_2Cl_2.

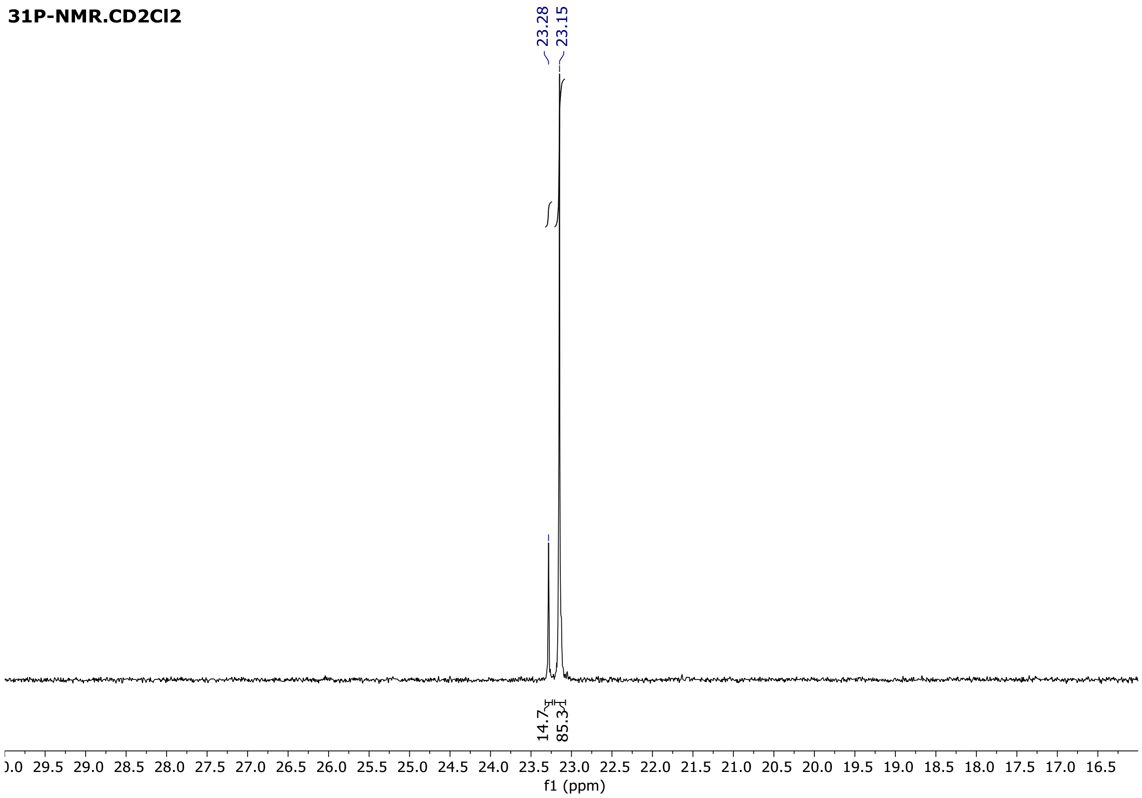

Figure S4 ^{31}P{^{1}H}-NMR spectrum of **3** in CD_2Cl_2.

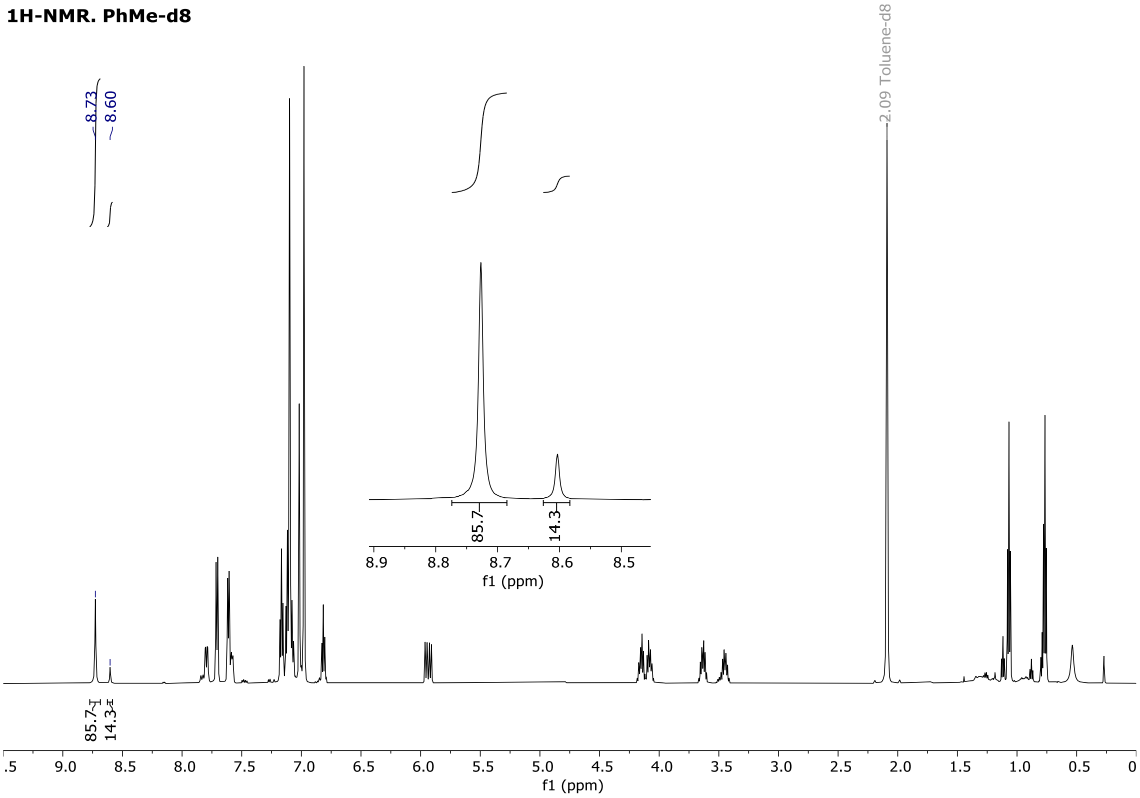

Figure S5 ^{1}H-NMR spectrum of **3** in PhMe-d_8.

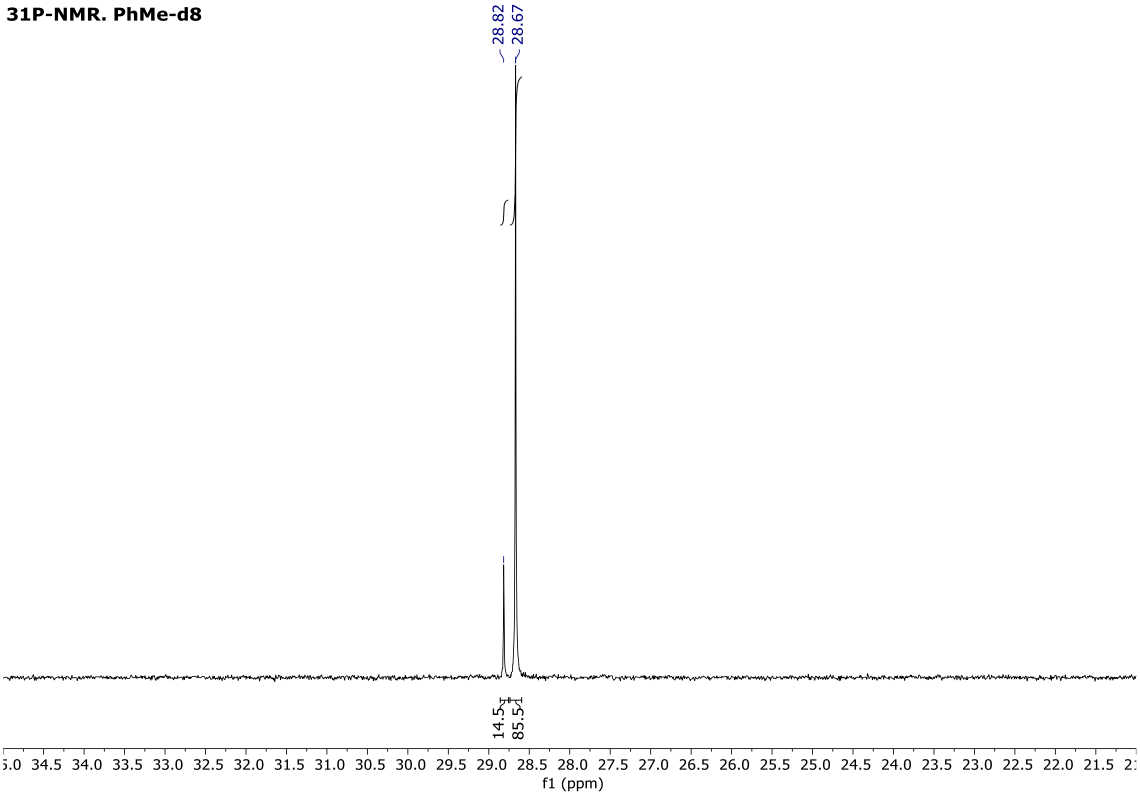

Figure S6 ^{31}P{^{1}H}-NMR spectrum of **3** in PhMe-d_8.

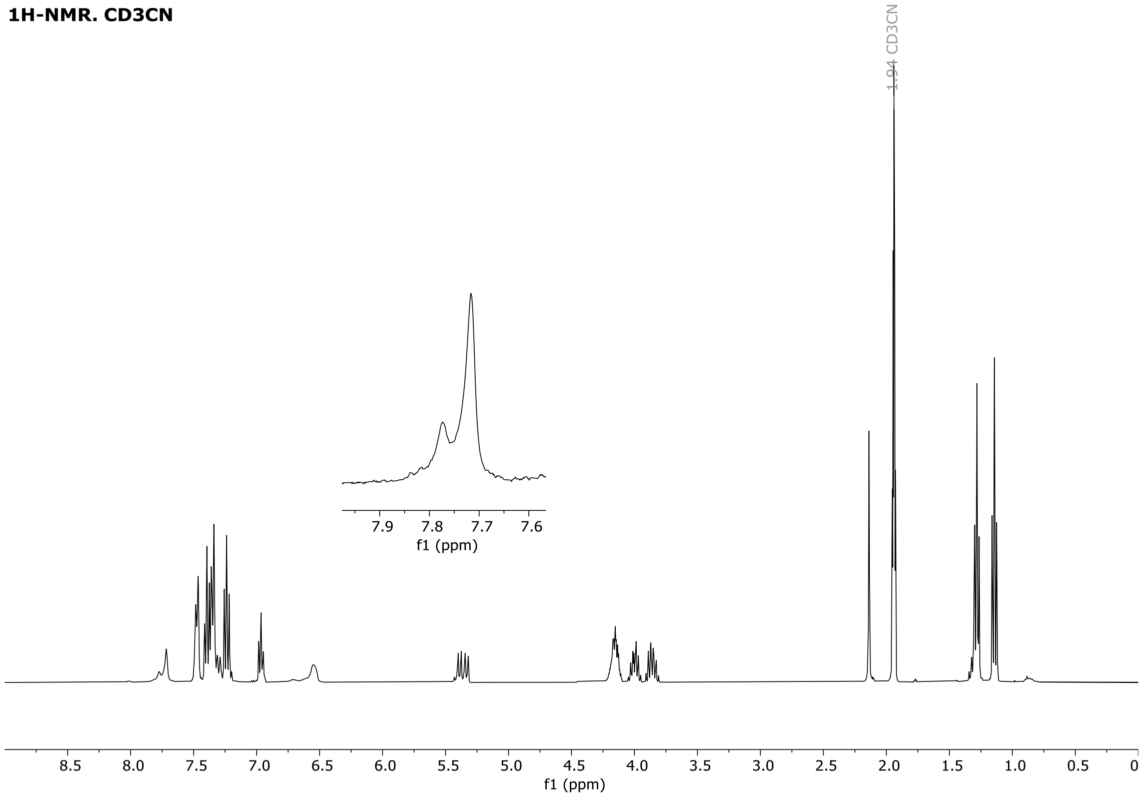

Figure S7 ^{1}H-NMR spectrum of **3** in CD_3CN.

NOTE: The broad N-H signal obtained in CD_3CN precluded their accurate integration by ^{1}H-NMR.

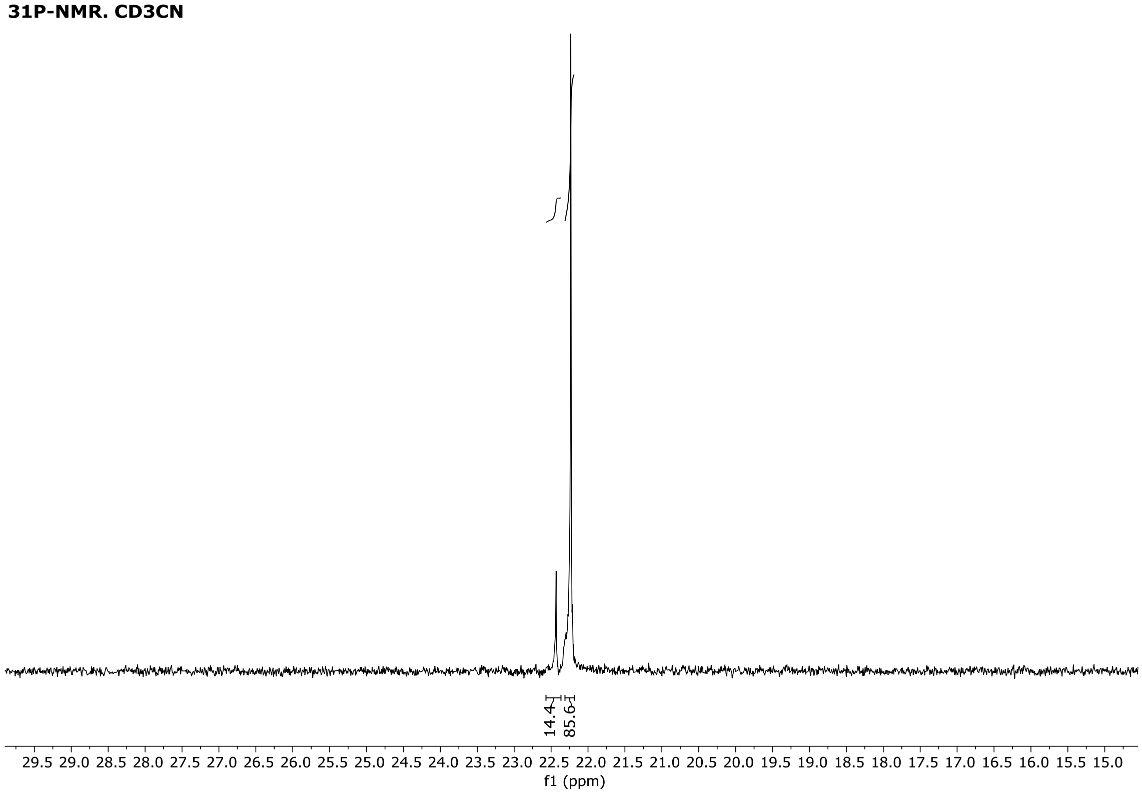

Figure S8 $^{31}P\{^{1}H\}$-NMR spectrum of **3** in CD_3CN.

1H-NMR. DMSO-d6

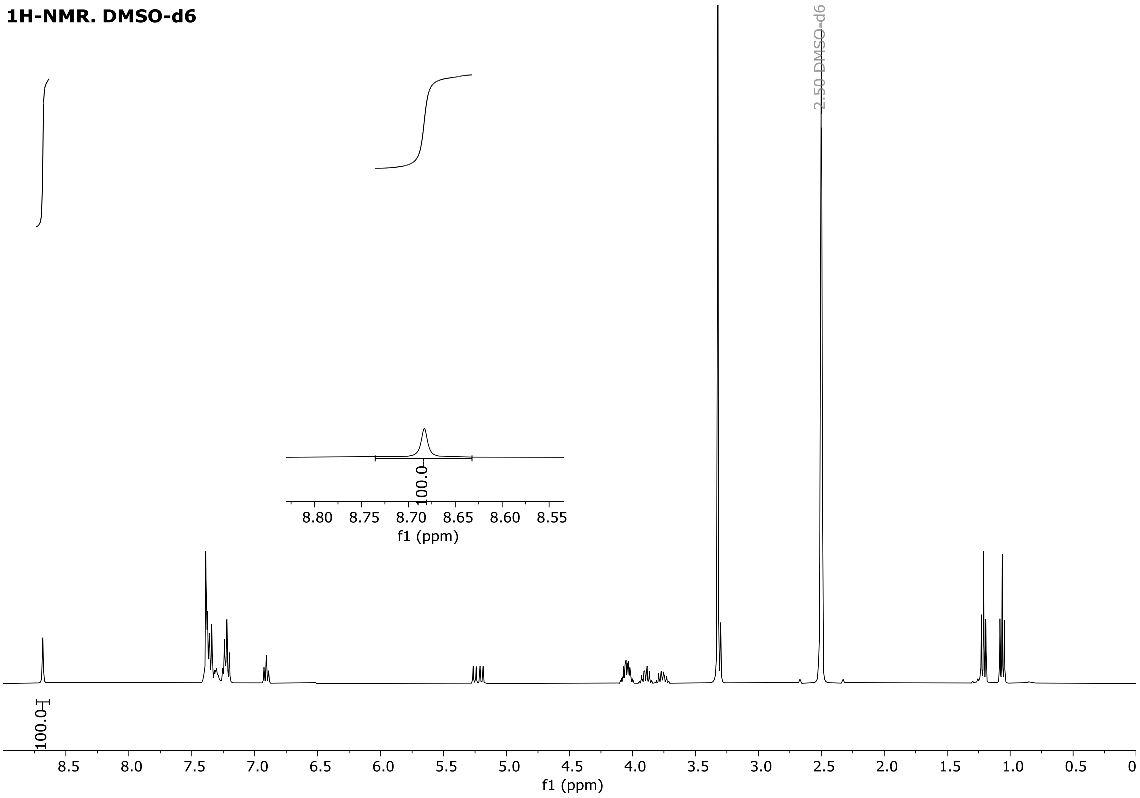

Figure S9 ^{1}H-NMR spectrum of **3** in DMSO-d_6.

31P-NMR. DMSO-d6

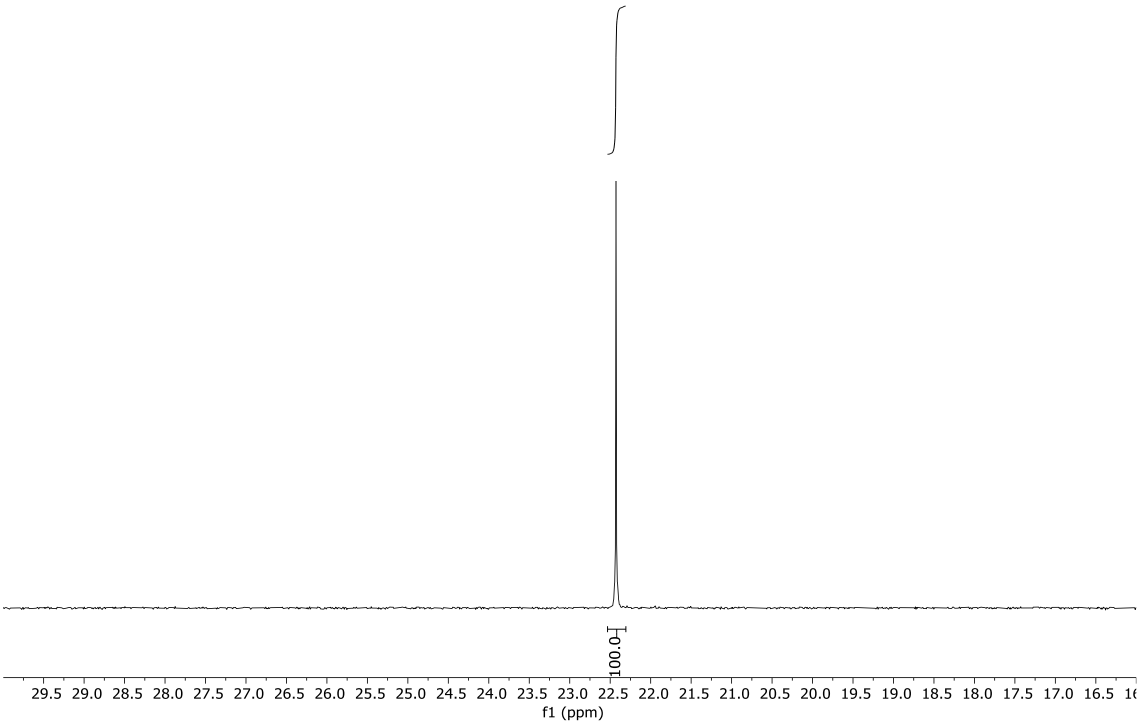

Figure S10 ^{31}P{^{1}H}-NMR spectrum of **3** in DMSO-d_6.

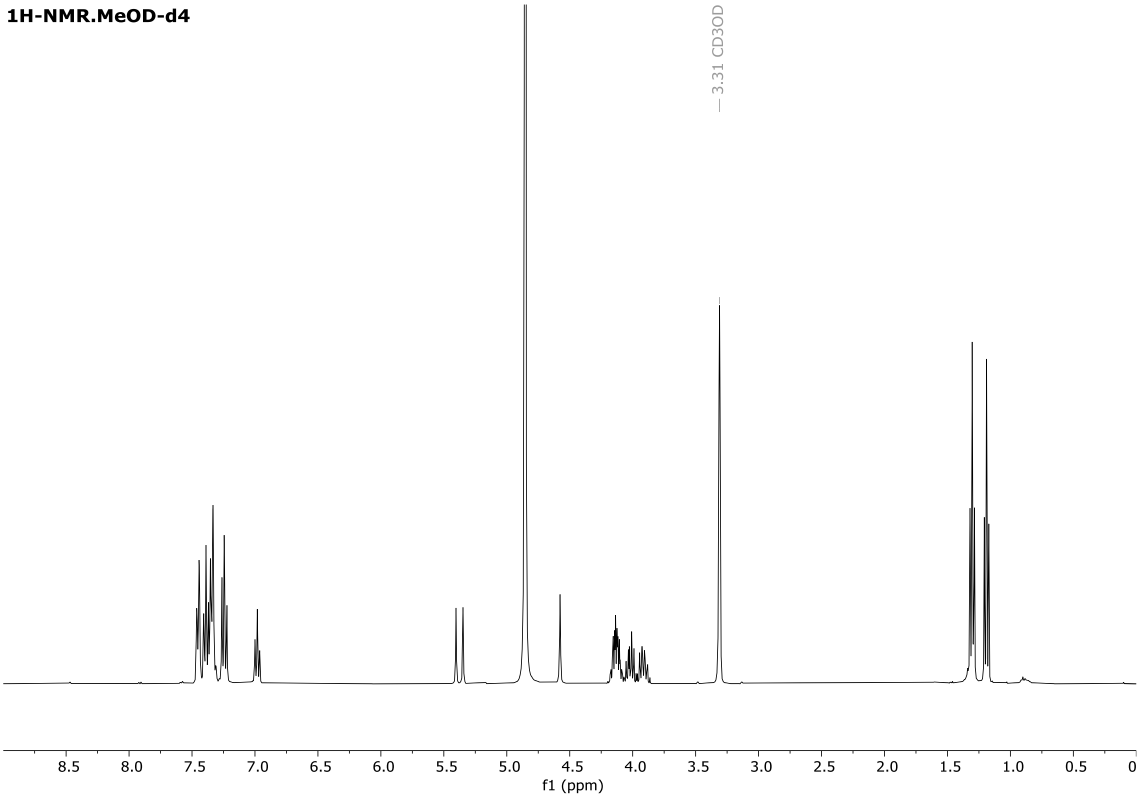

Figure S11 ^{1}H-NMR spectrum of **3** in MeOD-d_4.

NOTE: In MeOD-d_4, the N-H signals disappear due to proton-deuterium exchange with the solvent.

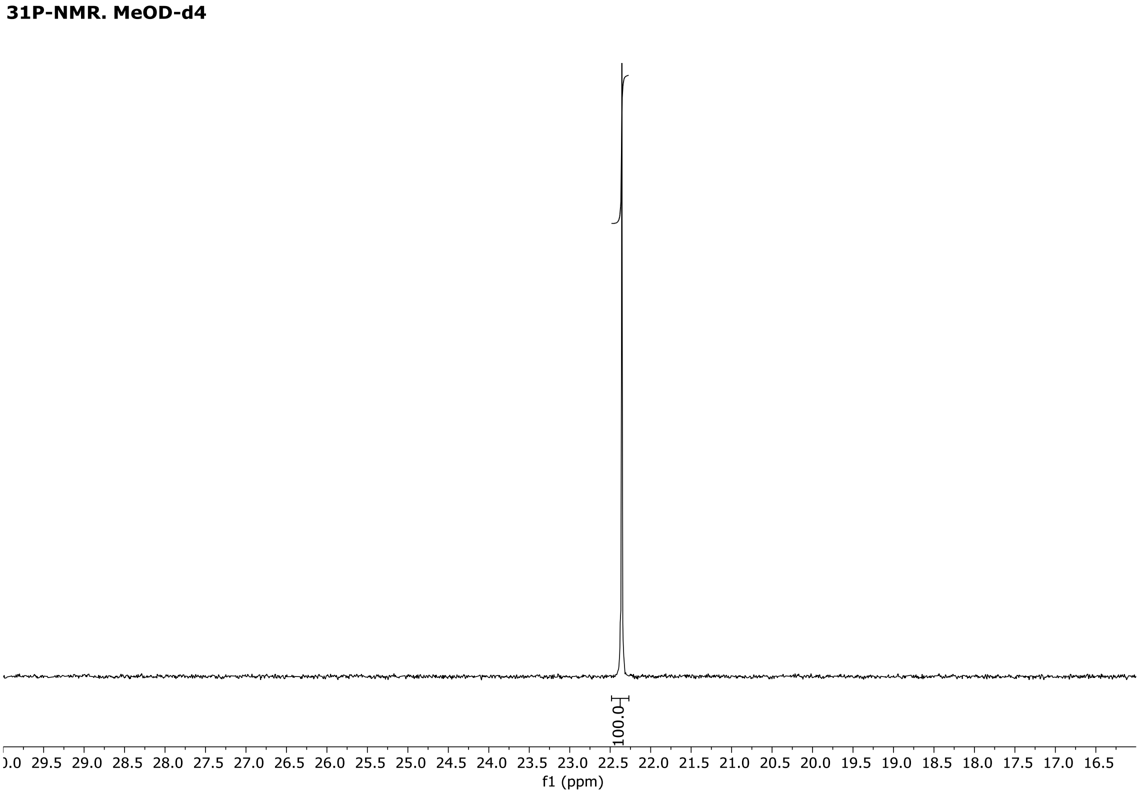

Figure S12 ^{31}P{^{1}H}-NMR spectrum of **3** in MeOD-d_4.

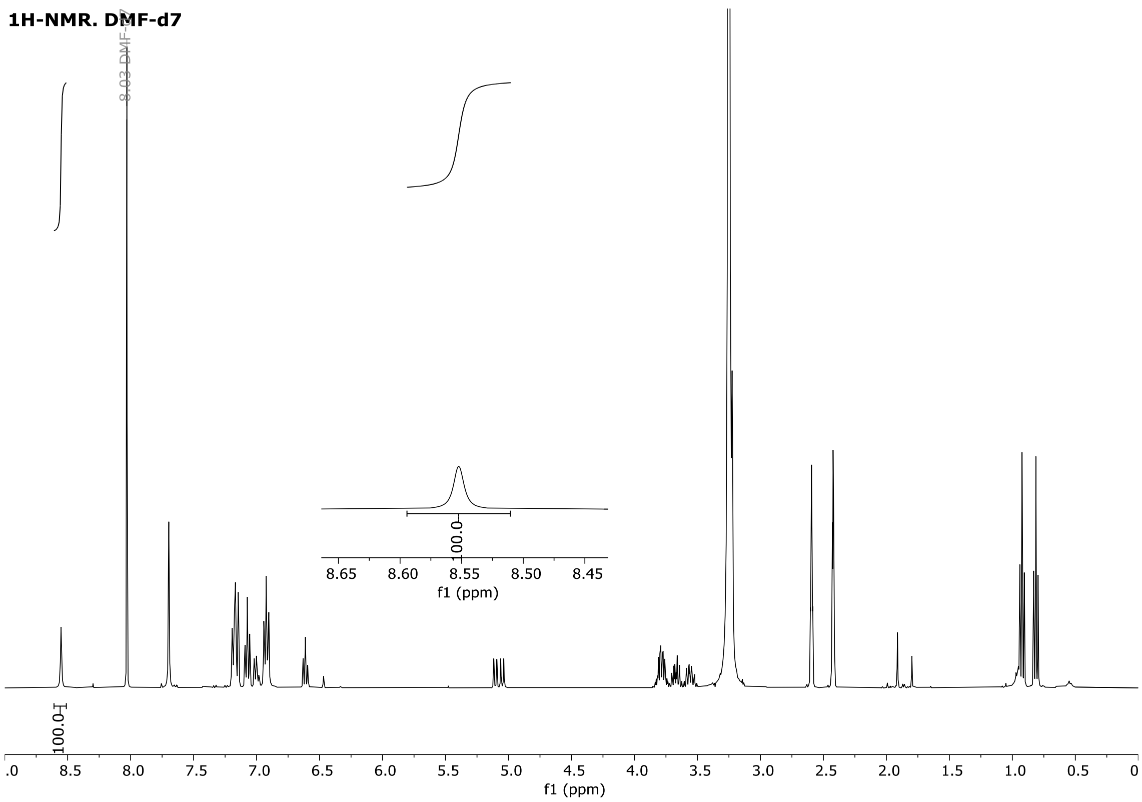

Figure S13 ^{1}H-NMR spectrum of **3** in DMF-d_7.

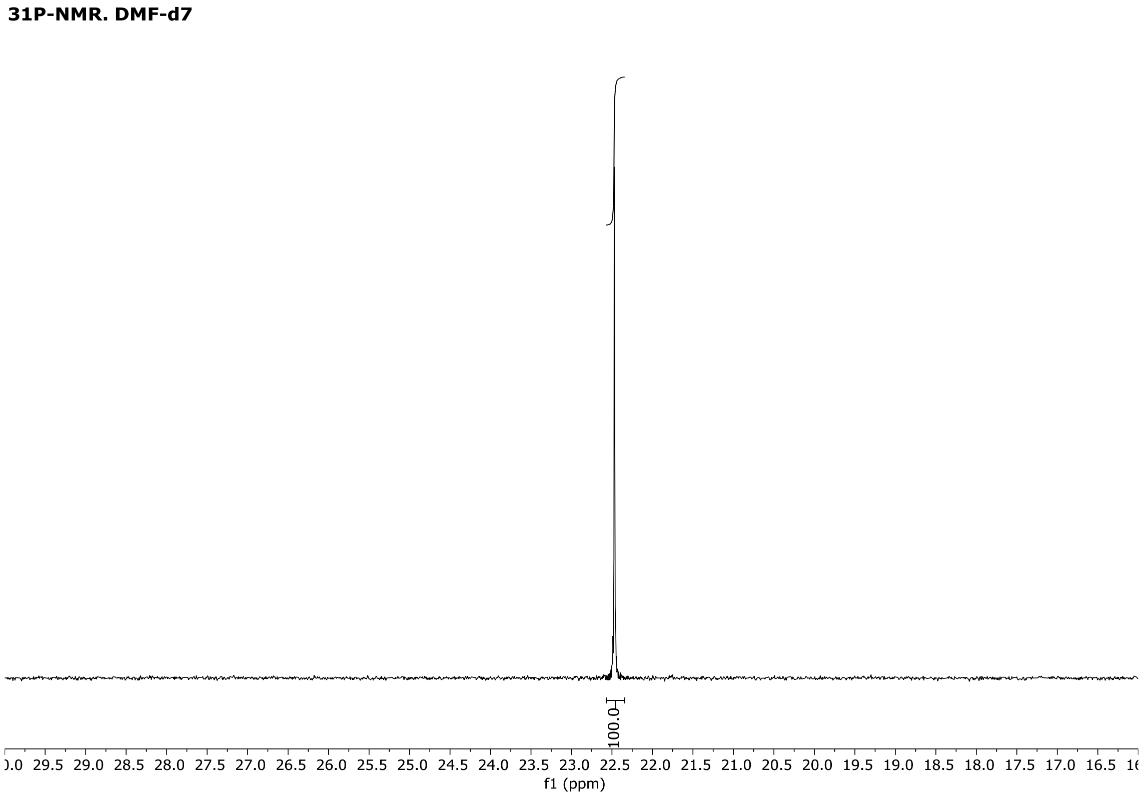

Figure S14 $^{31}P\{^{1}H\}$-NMR spectrum of **3** in DMF-d_7.

3.3. Studies on the SIDA effect. NMR spectra of 3 with varying enantiomeric ratios. Comparison with chiral HPLC

As reported previously,[16] the magnitude of the SIDA effect depends on the enantiomeric purity of the compound. Consequently, we investigated a range of enantiomeric ratios of **3** by ^{1}H-NMR (Scheme S1), ^{31}P{^{1}H}-NMR (Scheme S2) and enantioselective HPLC (Table S1). Clearly, racemic **3** and (*S*)-/(*R*)- enantioenriched **3** showed different NMR spectra, indicating a SIDA effect. Table S1 demonstrates that the measurement of the enantiomeric ratio by NMR correlates well with the enantiomeric ratio obtained by enantioselective HPLC analysis.[17] For enantiomeric purities <55:45 *e.r.*, the SIDA effect becomes negligible, obtaining a single set of peaks.

The different ratios of enantiomers were obtained by mixing the required amounts of the (*S*)-**3** (0.05 M stock solution in $CDCl_3$, 91:9 *e.r.*) and (*R*)-**3** (0.05 M stock solution in $CDCl_3$, 93:7 *e.r.*).[18]

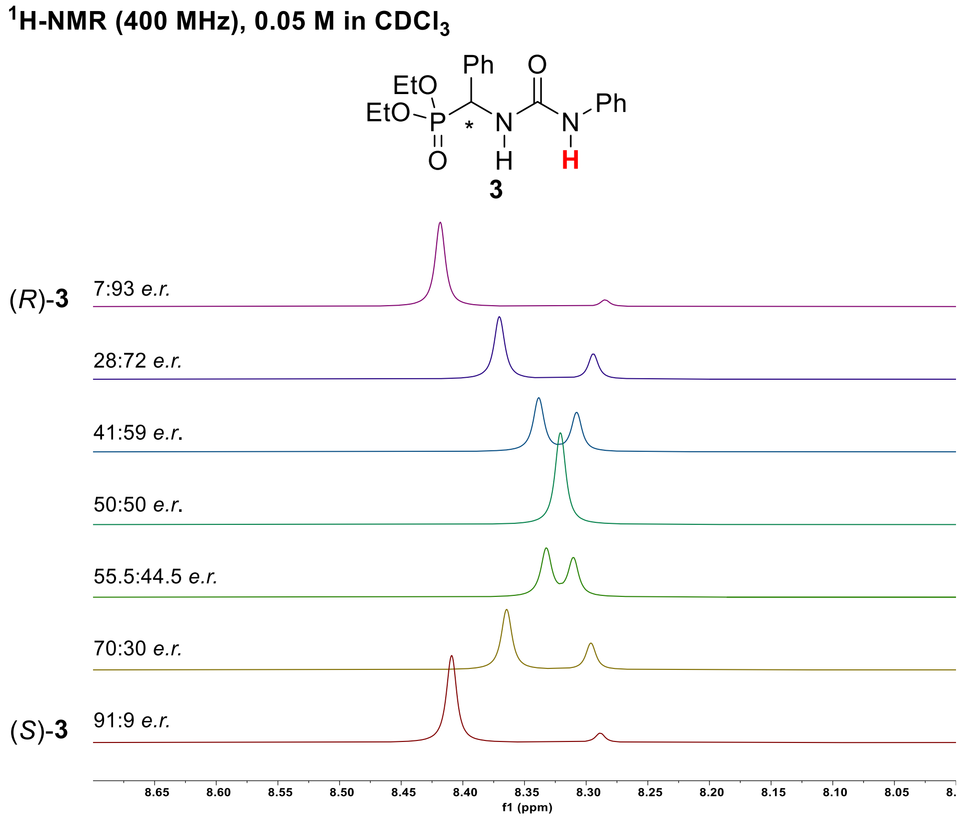

Scheme S1 Trancing the N-H signal (highlighted in red) of **3** by ^{1}H-NMR with different enantiomeric purities.

[16] Szakács, Z.; Sánta, Z.; Lomoschitz, A.; Szántay, C. *Trends Anal. Chem.* **2018**, *109*, 180.
[17] Chiralpak ID, Lux Cellulose-4, 85:15 heptane/^{i}PrOH, 1 ml/min, 25 min, λ_{abs} = 240 nm.
[18] NOTE: (*R*)-**3** was prepared through enantioselective hydrophosphonylation of arylidene urea **6** with diethyl phosphite using (*R*)-TiPSY as catalyst (see section 7.3).

$^{31}P\{^1H\}$-NMR (162 MHz), 0.05 M in $CDCl_3$

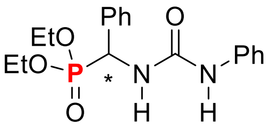

3

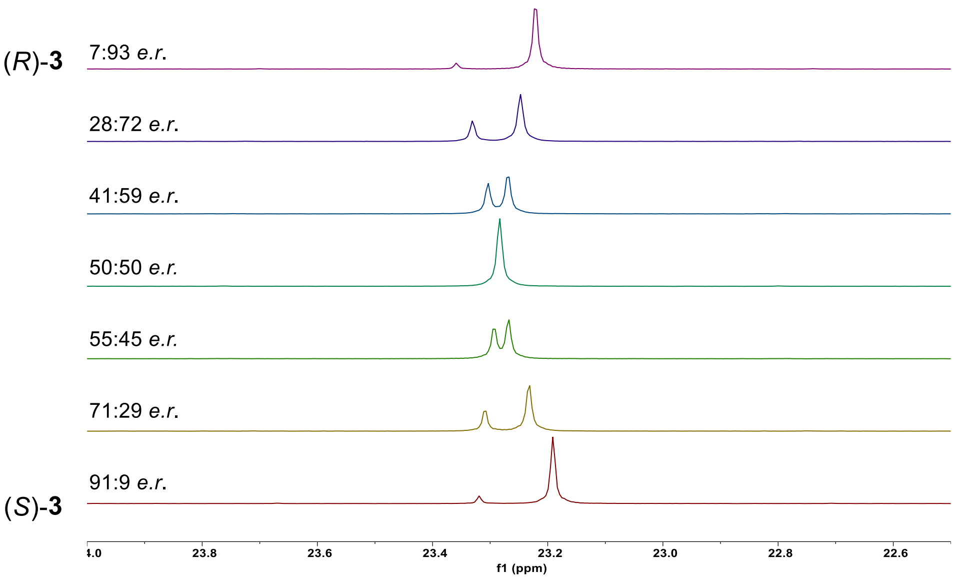

Scheme S2 Trancing the $^{31}P\{^1H\}$-NMR signal (highlighted in red) of **3** with different enantiomeric purities.

Table S1 Comparison of enantiomeric ratios obtained by ^1H-NMR and $^{31}P\{^1H\}$-NMR (in $CDCl_3$) thanks to the SIDA effect and the enantiomeric ratios obtained by enantioselective HPLC analysis of **3**.

**Entry**	***e.r.* by ^{1}H-NMR**	***e.r.* by $^{31}P\{^1H\}$-NMR**	***e.r.* by chiral HPLC**
**1**	91:9	91:9	91:9
**2**	70:30	71:29	70:30
**3**	55.5:44.5	55:45	55:45
**4**	-	-	49:51
**5**	41:59	41:59	41:59
**6**	28:72	28:72	27:73
**7**	7:93	7:93	7:93

3.4. 2D-DOSY NMR of 5 in $CDCl_3$ and DMSO-d_6

This study shows how the nature of the solvent influences the dimer-monomer equilibrium of α-ureidophosphonates, leading to a change in the diffusion coefficients. The value of the diffusion coefficients were obtained by 2D-DOSY NMR in a 500 MHz instrument at 25 °C. The values obtained in $CDCl_3$ and DMSO-d_6 for both enantioenriched and racemic compound **5** were related to the estimated molecular weight using the Stokes-Einstein Gierer-Wirtz estimation, as described in the literature.[19] The estimated molecular weight was obtained by using the spreadsheet provided by the authors of ref. 19, which can be downloaded in (https://www.nmr.chemistry.manchester.ac.uk/). Alternatively, the MestReNova v14.02 software used to process the NMR spectra of this work also enables the estimation of the molecular weight based on the DOSY experiment, providing analogous results.

NOTE: It is important to note that we never observed the dimer-monomer equilibrium when using $CDCl_3$ as solvent throughout a wide range of concentrations and temperatures, which probably indicates a high association constant. Thus, **5**[20] only exists as dimer in $CDCl_3$.

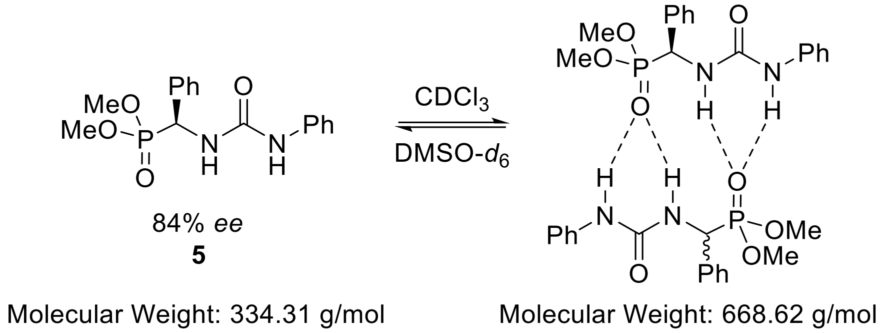

The 2D-DOSY NMR experiments were performed by dissolving 8-10 mg of **5** (batch with a 92:8 *e.r.* for scalemic material or racemic material) in the specified solvent (0.6 mL).

[19] Evans, R.; Dal Poggetto, G.; Nilsson, M.; Morris, G. A. *Anal. Chem.* **2018**, *90*, 3987-3994.

[20] **5** was prepared through enantioselective hydrophosphonylation of arylidene urea **6** with dimethyl phosphite using (*S*)-TiPSY as catalyst (see section 7.3).

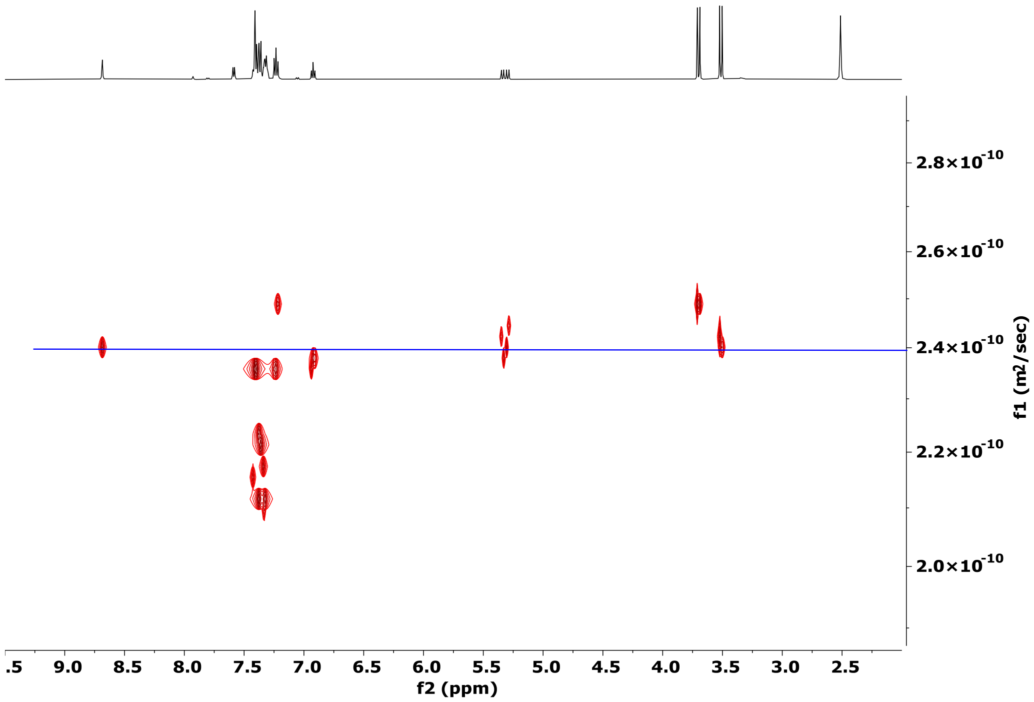

D = 2.4 x 10^{-10} m^2/s → M_W(estimated) = 390 g/mol

Figure S15 2D-DOSY NMR in DMSO-d_6 of scalemic **5**

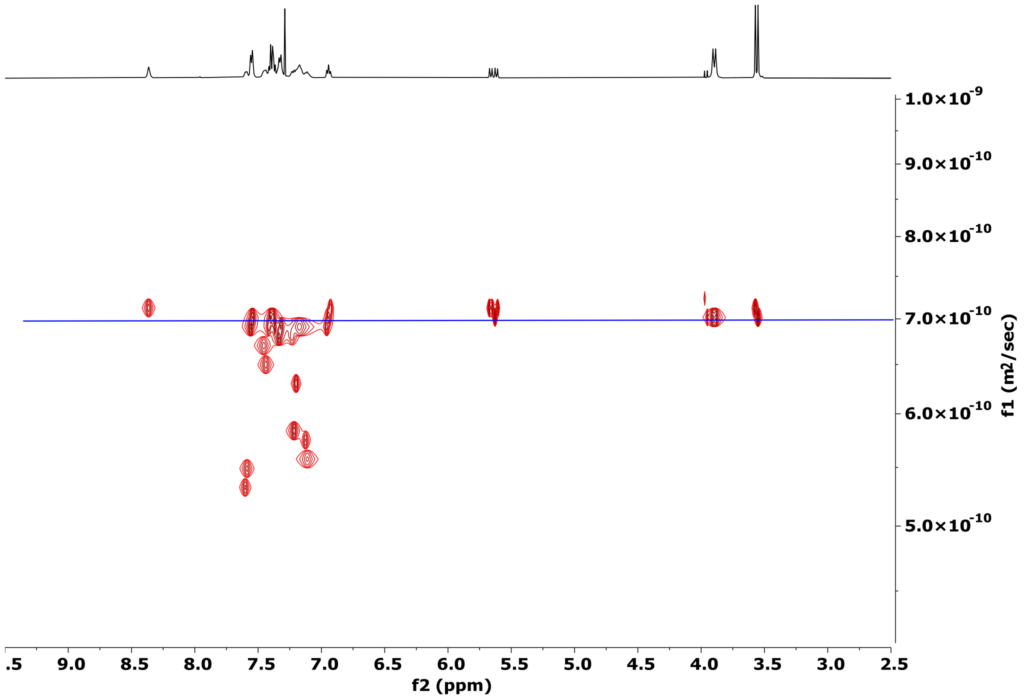

D = 7.0 x 10^{-10} m^2/s → M_W(estimated) = 865 g/mol

Figure S16 2D-DOSY NMR in $CDCl_3$ of scalemic **5**

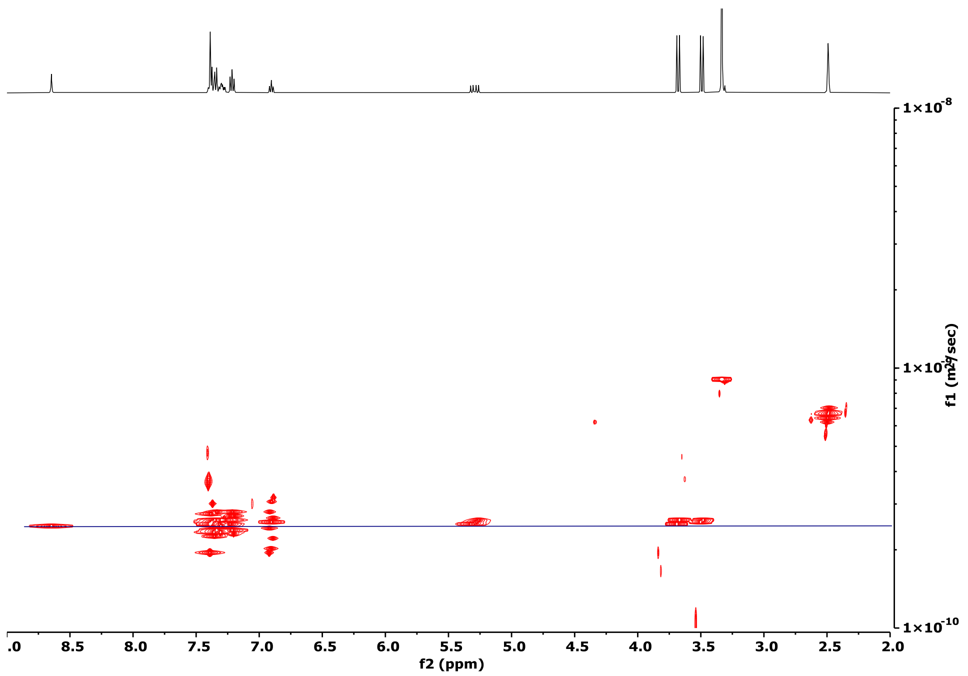

D = 2.4 x 10^{-10} m^2/s $\rightarrow$ M_W(estimated) = 383 g/mol

Figure S17 2D-DOSY NMR in DMSO-d_6 of racemic **5**

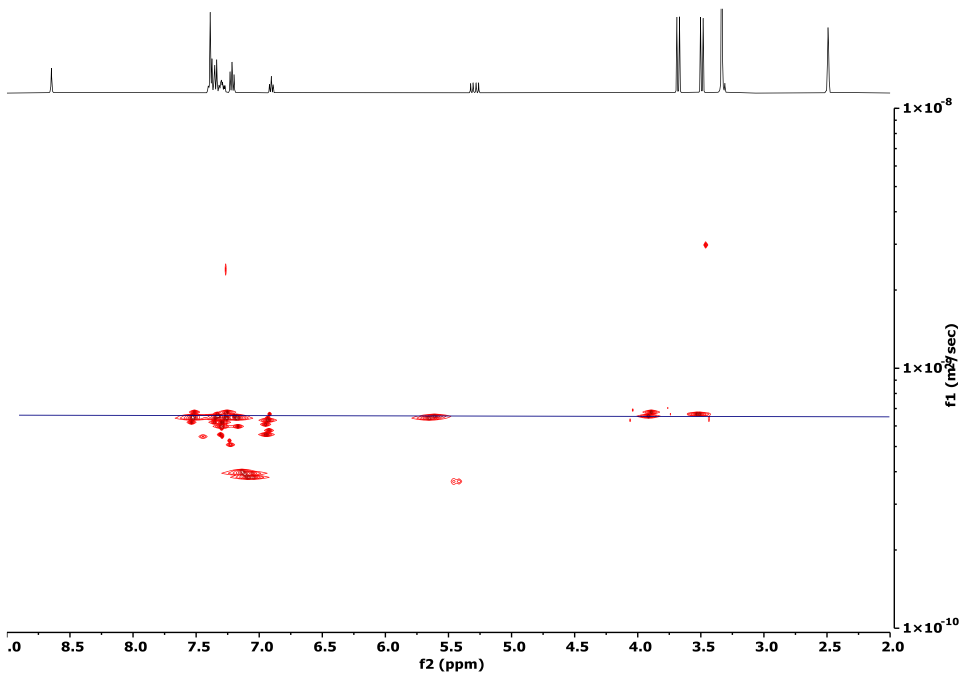

D = 6.6 x 10^{-10} m^2/s $\rightarrow$ M_W(estimated) = 985 g/mol

Figure S18 2D-DOSY NMR in $CDCl_3$ of racemic **5**

3.5. Determination of association constants

The association constant K_a of both racemic and enantioenriched **5** (97% *ee*) were determined by NMR titration following previously described methods.[21] Non-linear regression analysis allowed us to obtain the binding isotherm using platform http://app.supramolecular.org/bindfit/.[22]

According to the literature, the chemical shift of an specific signal can be related to the association constant as follows:

For an equilibrium monomer (M) - dimer (D), the association constant (K_a) can be described as:

$$2\,\mathrm{M} \rightleftarrows \mathrm{D}$$

$$(\mathrm{eq.}\,1)\quad \mathrm{K_a} = \frac{[\mathrm{D}]}{[\mathrm{M}]^2}$$

The initial concentration ($[M]_0$) being defined as:

$$(\mathrm{eq.}\,2)\quad [\mathrm{M}]_0 = [\mathrm{M}] + 2[\mathrm{D}]$$

In such scenario, the observed chemical shift of NMR signal (δ_{obs}) is a linear combination of the chemical shift of the monomeric species (δ_M) and the chemical shift of the dimeric species (δ_D) and directly proportional to the molar fraction of the dimer.

$$(\mathrm{eq.}\,3)\quad \delta_{obs} = \delta_M + (\delta_D - \delta_M)\left(\frac{2[\mathrm{D}]}{[\mathrm{M}]_0}\right) \quad \text{or} \quad \delta_{obs} = \delta_M + (\delta_D - \delta_M)\left(1 - \frac{[\mathrm{M}]}{[\mathrm{M}]_0}\right)$$

Combination of eq.1 and eq. 3, we can relate the observed chemical shift with the association constant:

$$\mathrm{K_a} = \frac{(\delta_D - \delta_M)(\delta_{obs} - \delta_M)}{2(\delta_D - \delta_{obs})^2[\mathrm{M}]_0}$$

In order to compute K_a, the values for δ_M and δ_D must be known. In the case of large values of K_a, the δ_D can be easily extrapolated, but the determination of δ_M will require very high dilutions, where the sensitivity of the instrument might be the limiting factor. Modern NMR instruments allow the calculation of association constants as high as 10^5 M^{-1}.[23] Non-linear regression analysis of the curves obtained by NMR titration (binding isotherm) enables the extrapolation of these values and direct determination of K_a. In our case, the fitting of the data was carried out with the open access tool BindFit (http://app.supramolecular.org/bindfit/). For a comprehensive review on this methodology, we refer to ref. 23.

Procedure: For the studies, samples with various concentrations (~0.5 – 80 mM) were prepared in freshly distilled $CDCl_3$. The ^{1}H-NMR spectra of **5** were recorded in a 500 MHz NMR instrument at 25 °C following the proton signal highlighted in red. The measurements were independently performed twice. All data regarding the experimental binding isotherms and the fitting analysis can be obtained via the following links (open access).

NOTE: In our experience, for concentrations <0.5 mM, significant peak broadening of the N-H signal precluded the accurate determination of the chemical shift. This might contribute to a worse extrapolation of δ_M and, eventually, affect to the calculation of K_a. However, our errors are comparable to those reported in the literature.[21]

---

[21] a) Nogales, D. F.; Ma, J.-S.; Lightner, D. A. *Tetrahedron* **1993**, *49*, 2361–2372; b) Ford, D. D.; Lehnherr, D.; Kennedy, C. R; Jacobsen, E. N. *J. Am. Chem. Soc.* **2016**, *138*, 7860-7863.

[22] a) P. Thordarson, *Chem. Soc. Rev.* **2011**, *40*, 1305-1323; b) D. Brynn Hibbert, Pall Thordarson, *Chem. Commun.* **2016**, *52*, 12792-12805.

[23] Thordarson, P. *Chem. Soc. Rev.* **2011**, *40*, 1305-1323.

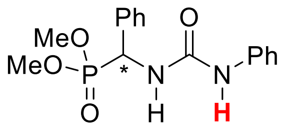

5

a) Racemic **5**

- Measurement 1 (racemic **5**):

http://app.supramolecular.org/bindfit/view/5fa2a37d-acfe-4e03-9f46-0a4376b5ce20

$K_a^1 = 8.5 \times 10^3\ M^{-1} \pm 0.2894\%$

- Measurement 2 (racemic **5**):

http://app.supramolecular.org/bindfit/view/476a4700-8340-4545-8df6-b566a5def1a7

$K_a^2 = 8.2 \times 10^3\ M^{-1} \pm 2.0252\%$

K_a(racemic **5**) = $(8.3 \pm 0.2) \times 10^3\ M^{-1}$

b) Enantioenriched **5** (97% *ee*)

- Measurement 1 (enantioenriched **5**):

http://app.supramolecular.org/bindfit/view/810d5d1a-623b-4b7e-89f2-ac5787990ada

$K_a^1 = 2.7 \times 10^3\ M^{-1} \pm 3.4197\%$

- Measurement 2 (enantioenriched **5**):

http://app.supramolecular.org/bindfit/view/c9d0d58a-c0d1-4b03-a425-11da9332575d

$K_a^2 = 3.9 \times 10^3\ M^{-1} \pm 0.9734\%$

K_a(enantioenriched **5**) = $(3.3 \pm 0.8) \times 10^3\ M^{-1}$

4. Optimization of the enantioselective hydrophosphonylation of arylidene ureas

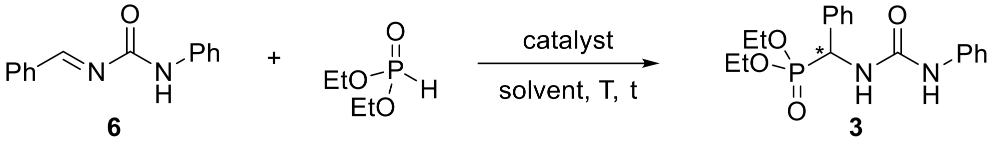

NOTE: The quality of the alkylidene urea was crucial for the reproducibility of the conversion and enantioselectivity. At late stages of the optimization studies, we discovered that using deoxygenated toluene significantly improved the conversion.[24] The quality of the solvent did not have a major impact on the enantioselectivity, though (see Table S3). For the sake of reproducibility and coherence, the whole optimization studies were conducted with the same toluene grade.

Conversion was determined by $^{31}P\{^1H\}$-NMR. The enantiomeric ratio (*e.r.*) of the crude reaction mixture was determined by enantioselective HPLC analysis and by ^1H-NMR and/or $^{31}P\{^1H\}$-NMR from a sample of reaction mixture (0.1 mL) and diluted with $CDCl_3$ (0.4 mL).

Chiralpak AD-H, *n*-heptane/^{i}PrOH 95/5, 40 °C, 1 mL/min, λ_{abs} = 240 nm $t_R(R)$ = 43.6 min, $t_R(S)$ = 48.4 min.

Lux Cellulose-4, *n*-heptane/^{i}PrOH 85/15, 40 °C, 1 mL/min, λ_{abs} = 240 nm $t_R(S)$ = 7.9 min, $t_R(R)$ = 17.6 min.

Catalyst screening

Freshly prepared alkylidene urea **6** (44.9 mg, 0.2 mmol, 1.00 equiv.) and the specified catalyst (10 mol% unless otherwise noted) were dissolved in anhydrous toluene (0.7 mL). A stock solution of diethyl phosphite (0.03 mL, 0.24 mmol, 1.2 equiv.) in toluene (0.3 mL) was added dropwise over period of 1 min to the reaction mixture. The solution was stirred at room temperature for 18 h.

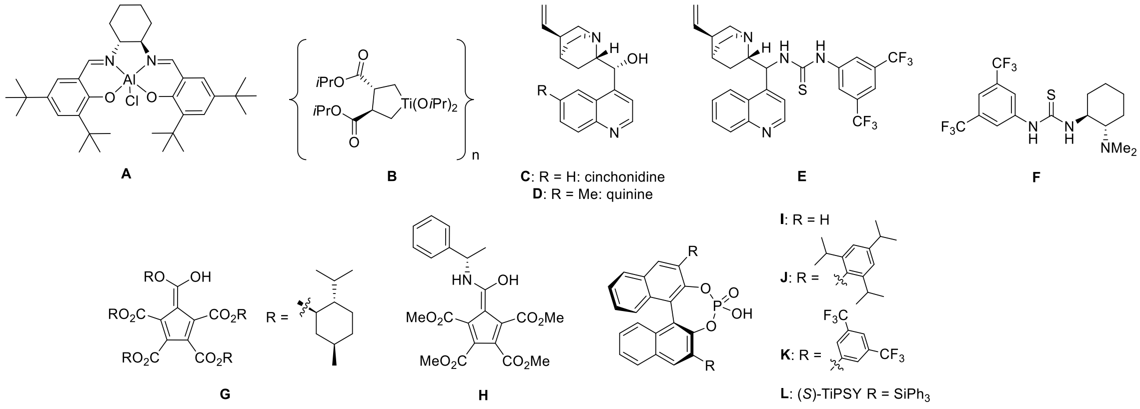

Chart S1

[24] For a similar observation see: Cheng, X.; Goddard, R.; Buth, G.; List, B. *Angew. Chem. Int. Ed.* **2008**, *47*, 5079-5081.

Table S2 Catalyst screening.

**Entry**	**Catalyst**	**Conversion [%]**	***e.r.* by NMR (HPLC)**
1	**-**	28	-
2	**C**	31	(52:48)
3	**D**	40	(52:48)
4	**E**	<1	(63:37)
5	**F**	9	(56:44)
6	**G**	40	(49:51)
7	**H**	47	(46:54)
8	**A**	89	(49:51)
9	**B**	26	37:63 (36:64)
10	**I**	83	(63:37)
11	**J**	61	77:23 (78:22)
12	**K**	83	72:28 (77:23)
13	**L**, (*S*)-TIPSY (10 mol%)	79	87:13 (87:13)
14	**L**, (*S*)-TIPSY (5 mol%) commercial	66	85:15 (86:14)
15	**L**, (*S*)-TIPSY (2.5 mol%)	59	82.5:17.5 (82:18)
16	**L**, (*S*)-TIPSY (5 mol%) home-made	58	88:12 (89:11)
17	(*R*)-TIPSY (5 mol%) home-made	73	84:16 (85:15)

Solvent screening

Freshly prepared alkylidene urea **6** (44.9 mg, 0.2 mmol, 1.00 equiv.) and (*S*)-TiPSY (8.7 mg, 0.01 mmol, 5.0 mol%) were dissolved in the specified anhydrous solvent (0.7 mL). A stock solution of diethyl phosphite (0.03 mL, 0.24 mmol, 1.2 equiv.) in the specified anhydrous solvent (0.3 mL) was added dropwise over period of 1 min to the reaction mixture. The solution was stirred at room temperature for 17 h.

Table S3 Solvent screening.

Entry	Solvent	Conversion [%]	*e.r.* by NMR (HPLC)
1	Et_2O	70	80:20 (79:21)
2	THF	39	70:30 (68:32)
3	*o*-xylene	73	81:21 (82:18)
4	cyHex	94	70:30 (73:27)
5	PhMe:cyHex (2:1)	71	81:19 (81:19)
6	DCM	29	79:21 (79:21)
7	PhMe:DCM (1:1)	50	81:19 (81:19)
8	PhMe	64	87:13 (87:13)
9[a)]	PhMe	54	93:7 (93:7)
10[a)]	Freshly opened bottle of PhMe	76	92:8 (93:7)
11[a)]	PhMe from SPS	76	93:7 (94:6)
12[a)]	Deoxygenated PhMe	88	92:8 (94:6)

a) Reaction concentration 0.1 M, SPS: solvent purifying system

Concentration

Freshly prepared alkylidene urea **6** (44.9 mg, 0.2 mmol, 1.00 equiv.) and (*S*)-TiPSY (8.7 mg, 0.01 mmol, 5.0 mol%) were dissolved in anhydrous toluene. A stock solution of diethyl phosphite (0.03 mL, 0.24 mmol, 1.2 equiv.) in anhydrous toluene (0.3 mL) was added dropwise over period of 1 min to the reaction mixture. The solution was stirred at room temperature for 19 h.

Table S4 Influence of reaction concentration.

Entry	Concentration [M]	Conversion [%]	*e.r.* by NMR (HPLC)
1	0.025	19	(97:3)
2	0.05	36	94:6 (95:5)
3	0.1	54	93:7 (93:7)
4	0.2	64	87:13 (87:13)
5[a)]	0.5	72	79:21 (80:20)

a) (*S*)-TIPSY (5 mol%) commercial

Stoichiometry

Freshly prepared alkylidene urea **6** (44.9 mg, 0.2 mmol, 1.00 equiv.) and (*S*)-TiPSY (8.7 mg, 0.01 mmol, 5.0 mol%) were dissolved in anhydrous toluene (0.7 mL). A stock solution of diethyl phosphite in anhydrous toluene (0.3 mL) was added dropwise over period of 1 min to the reaction mixture. The solution was stirred at room temperature for 17 h.

Table S5 Influence of arylidene urea **6**:phosphite ratio.

Entry	Ratio arylidene urea 6 :phosphite	Conversion [%]	*e.r.* by NMR (HPLC)
1	1:1.2	54	93:7 (93:7)
2	1:2	67	87:13 (87:13)
3	1:2, addition of phosphite over 1 day	45	92:8 (93:7)

Temperature

Freshly prepared alkylidene urea **6** (44.9 mg, 0.2 mmol, 1.00 equiv.) and (*S*)-TiPSY (8.7 mg, 0.01 mmol, 5.0 mol%) were dissolved in anhydrous toluene (0.7 mL). A stock solution of diethyl phosphite (0.03 mL, 0.24 mmol, 1.2 equiv.) in anhydrous toluene (0.3 mL) was added dropwise over period of 1 min to the reaction mixture. The solution was stirred at the specified temperature for 19 h.

Table S6 Influence of reaction temperature.

Entry	Temperature [°C]	Conversion [%]	*e.r.* by NMR (HPLC)
1	7	54	81:19 (82:18)
2	25	64	87:13 (87:13)

Additives

Freshly prepared alkylidene urea **6** (44.9 mg, 0.2 mmol, 1.00 equiv.) and specified catalyst were dissolved in anhydrous toluene. A stock solution of diethyl phosphite (0.03 mL, 0.24 mmol, 1.2 equiv.) in anhydrous toluene (0.3 mL) was added dropwise over period of 1 min to the reaction mixture. The solution was stirred at room temperature for 19 h.

Table S7 Influence of additives.

Entry	Additive	Conversion [%]	*e.r.* by NMR (HPLC)
1[a]	3 Å MS (300 mg)	46	(72:28)
2[a]	4 Å MS (300 mg)	33	73:27 (70:30)
3[a]	4 Å MS (powder, (300 mg))	37	70:30 (72:28)
4[b]	5 Å MS (300 mg)	38	(91:9)
5[b]	$MgSO_4$ (5 equiv.)	15	(92:8)

a) Using catalyst (*S*)-**K** (3.9 mg, 0.005 mmol, 2.5 mol%) in toluene (1 mL, total volume). b) Using (*S*)-TiPSY (8.7 mg, 0.01 mmol, 5.0 mol%) in toluene (2 mL, total volume). MS: molecular sieves.

5. Determination of the absolute configuration of 3

α-Aminophosphonate **3**, prepared from the multi-step route shown in section 3.1, is known to have an (*S*) configuration.[3] The enantioselective HPLC chromatogram of its corresponding α-ureidophosphonate **3**, was compared to the chiral HPLC chromatogram of **3** obtained through (*S*)-TiPSY catalyzed hydrophosphonylation of **6**, which allowed the assignment of the absolute configuration to be (*S*) (Scheme S3).

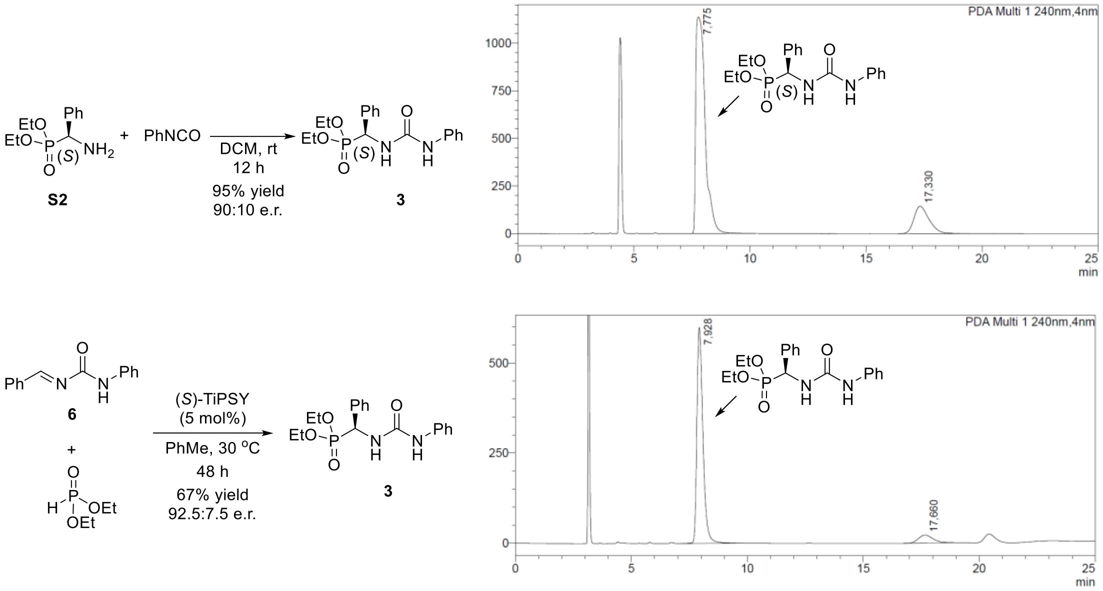

Scheme S3 Determination of the absolute configuration of α-ureidophosphonate **3**. HPLC conditions: Chiralpak ID, OX-H, 85:15 heptane/^{i}PrOH, 1 mL/min, 25 min, λ_{abs} = 240 nm.

By analogy, we assigned the same (*S*)-configuration to other α-ureidophosphonates synthesized through (*S*)-TiPSY catalyzed hydrophosphonylation of arylidene ureas.

6. Increasing optical purity by Self-Disproportionation of Enantiomers (SDE)

6.1. Achiral column chromatography

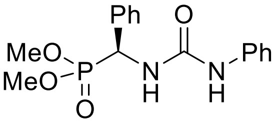

5

The crude material of α-ureidophosphonate **5**, prepared according to the general procedure D, was purified by column chromatography under following conditions

e.r. of crude material: 92:8 ($^{31}P\{^{1}H\}$-NMR in $CDCl_3$)

Reaction scale: 1.68 mmol

Column: Manual column, medium pressure (Ø = 3 cm, L (silica) = 14 cm)

Silica: 45 g, SilicaFlash® P60, 230-400 mesh

Particle diameter: 40-63 µm

Eluent: hexane/^{i}PrOH 90:10

Fraction	*e.r.* ($^{31}P\{^{1}H\}$-NMR in $CDCl_3$)	*e.r.* (chiral SFC)[a]	Yield [%]
1	98:2	99:1	31
2	91:9	92:8	48
3	58:42	59:41	9
4	-[b]	52:48	8
Combined		88:12	96

a) SFC Chiralpak ID, ^{i}PrOH 1-40%, 20 min, 4 mL/min, λ_{abs} = 240 nm, $t_R(R)$ = 13.8 min, $t_R(S)$ = 14.9 min; b) not possible to determine by NMR because of insufficient peak separation

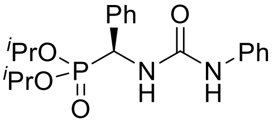

7

The crude material of α-ureidophosphonate **7** prepared according to the general procedure C was purified by column chromatography under following conditions

e.r. of crude material: 77:23 ($^{31}P\{^{1}H\}$-NMR in $CDCl_3$)

Reaction scale: 0.2 mmol

Column: Autocolumn Biotage® Selekt

Silica: 5 g, spherical silica (Biotage® Sfär Silica HC D)

Average particle diameter: 24 µm

Eluent: pentane/EtOAc 90:10

Fraction	*e.r.* ($^{31}P\{^1H\}$-NMR in $CDCl_3$)	*e.r.* (HPLC)[a]	Yield [%]
1	51:49	51:49	3
2	61:39	60:40	32
3	95:5	95:5	37
4	98:2	98:2	4
Combined	79:21	79:21	76

a) **HPLC** Lux Cellulose-4, *n*-heptane/^{i}PrOH 90:10, 40 °C, 1 mL/min, λ_{abs} = 240 nm, $t_R(S)$ = 7.9 min, $t_R(R)$ = 10.5 min.

6.2. Preferential dissolution of the homochiral dimer

During our studies on the characterization of the homo- and heterochiral dimers we observed a remarkable difference in the solubility between highly enantioenriched and nearly racemic samples, the former are significantly more soluble in a wide range of solvents than the latter. Based on our experience on harnessing the different solubility of racemic and enantiopure phosphoramidates in water,[25] we explored the preferential dissolution of the homochiral dimer in a scalemic mixture of **3**.

In a typical experiment, in a vial containing **3** (135.5 mg), with an initial 79:21 *e.r.*, the minimal amount of hexane:^{i}PrOH (8:2) was added to dissolve the compound (14 mL, slight warming of the solution was necessary), obtaining a clear and colorless solution. The vial, at room temperature, was covered with aluminum foil and introduced in the fridge (at 2 ºC) for 40 h. After that time, the formation of a semicrystalline white solid in the bottom of the vial was observed. At this point, the supernatant was transferred to another vial and both fractions were analyzed by enantioselective HPLC (NOTE: The solid fraction was fully dissolved in DCM for accurate determination of the enantiomeric purity). The results are depicted in Table S8.

Table S8 Separation of enantiomers by preferential dissolution of the homochiral dimers.

	Mass (mg)	*e.r.*
**Initial**	135.5	79:21
Solid fraction	42.2	53:47
Soluble fraction	92.1	92.5:7.5

[25] Dašková, V.; Buter, J; Schoonen, A. K.; Lutz, M.; de Vries, F.; Feringa, B. L. *Angew. Chem. Int. Ed.* **2021**, *60*, 11120-11126.

7. Synthetic procedures and spectral data

7.1. Synthesis and characterization of α-ureidosulfones

General method A for the preparation of α-ureidosulfones[26]

In an open round bottom flask, the corresponding urea (20.0 mmol, 1.00 equiv.) and *p*-toluenesulfinic acid sodium salt (40.0 mmol, 2.00 equiv.) were suspended in H_2O/MeOH mixture (2:1, 54 mL). The corresponding aldehyde (30.0 mmol, 1.50 equiv.) was added, followed by formic acid (95%, 1.5 mL). The reaction mixture was stirred for 1 d at room temperature. The resulting precipitate was filtered, washed with H_2O and pentane successively and dried under vacuum. The resulting white solid was used without further purification for preparation of imines.

NOTE: Several α-ureidosulfones proved to be unstable in solution.

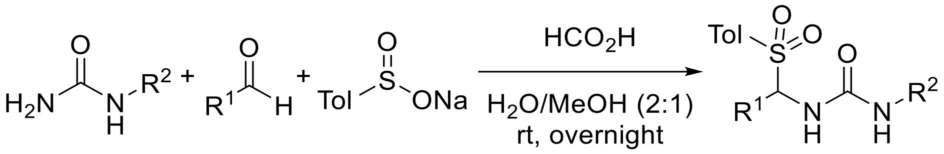

Scheme S4 Preparation of α-ureidosulfones.

1-phenyl-3-(phenyl(tosyl)methyl)urea (S3)

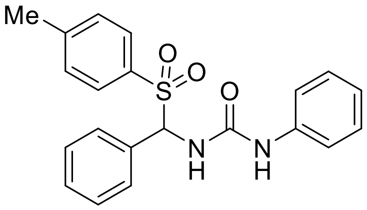

Prepared according to the general procedure A starting from *N*-phenylurea (2.72 g, 20.0 mmol, 1.00 equiv.) and benzaldehyde (3.0 mL, 30.0 mmol, 1.50 equiv.). Product **S3** was obtained as a white solid (7.06 g, 18.6 mmol, 93%).

^{1}H NMR (600 MHz, DMSO-*d*$_6$) δ 8.53 (s, 1H), 7.87 (d, *J* = 10.7 Hz, 1H), 7.74 (d, *J* = 8.3 Hz, 2H), 7.52 – 7.47 (m, 2H), 7.44 (dd, *J* = 5.0, 1.9 Hz, 3H), 7.38 (d, *J* = 7.9 Hz, 2H), 7.24 – 7.17 (m, 4H), 6.95 – 6.90 (m, 1H), 6.23 (d, *J* = 10.6 Hz, 1H), 2.34 (s, 3H); **^{13}C NMR** (151 MHz, DMSO-*d*$_6$) δ 153.0, 144.6, 139.2, 133.9, 131.2, 129.6, 129.3, 129.1, 129.0, 128.7, 128.3, 122.0, 118.1, 72.8, 21.1; **HRMS** (ESI+, *m*/*z*): calculated for $C_{21}H_{20}N_2O_3SNa^+$ [M + Na]$^+$: 403.1087, found: 403.1087.

[26] a) Lillo, V. J.; Mansilla, J.; Saá, J. M. *Org. Biomol. Chem.*, **2018**, *16*, 4527-4536. b) C. T. Mbofana, S. J. Miller, *J. Am. Chem. Soc.*, **2014**, *136*, 3285-3292.

1-((4-chlorophenyl)(tosyl)methyl)-3-phenylurea (S4)

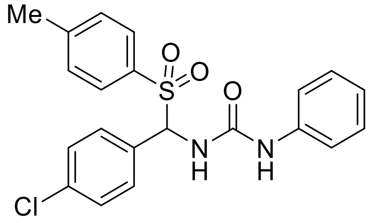

Prepared according to the general procedure A starting from *N*-phenylurea (2.72 g, 20.0 mmol, 1.00 equiv.) and 4-chlorobenzaldehyde (3.2 mL, 30.0 mmol, 1.50 equiv.). Product **S4** was obtained as a white solid (6.91 g, 16.7 mmol, 83%).

^{1}H NMR (600 MHz, DMSO-d_6) δ 8.50 (s, 1H), 7.88 (d, *J* = 10.6 Hz, 1H), 7.76 (d, *J* = 8.2 Hz, 2H), 7.58 – 7.50 (m, 4H), 7.39 (d, *J* = 8.0 Hz, 2H), 7.25 – 7.14 (m, 4H), 6.98 – 6.86 (m, 1H), 6.29 (d, *J* = 10.6 Hz, 1H), 2.35 (s, 3H); **^{13}C NMR** (151 MHz, DMSO-d_6) δ 152.9, 144.8, 139.1, 134.2, 133.7, 130.8, 130.2, 129.6, 129.2, 128.7, 128.4, 122.0, 118.1, 72.0, 21.1; **HRMS** (ESI+, *m*/*z*): calculated for $C_{21}H_{19}ClN_2O_3SNa^+$ [M + Na]$^+$: 437.0697, found: 437.0696.

1-((4-fluorophenyl)(tosyl)methyl)-3-phenylurea (S5)

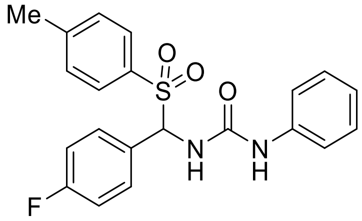

Prepared according to the general procedure A starting from *N*-phenylurea (2.72 g, 20.0 mmol, 1.00 equiv.) and 4-fluorobenzaldehyde (3.2 mL, 30.0 mmol, 1.50 equiv.). Product **S5** was obtained as a white solid (7.83 g, 19.7 mmol, 98%).

^{1}H NMR (600 MHz, DMSO-d_6) δ 8.51 (s, 1H), 7.87 (d, *J* = 10.6 Hz, 1H), 7.75 (d, *J* = 8.3 Hz, 2H), 7.61 – 7.52 (m, 2H), 7.38 (d, *J* = 8.0 Hz, 2H), 7.30 (t, *J* = 8.8 Hz, 2H), 7.24 – 7.15 (m, 4H), 6.96 – 6.86 (m, 1H), 6.29 (d, *J* = 10.6 Hz, 1H), 2.34 (s, 3H); **^{13}C NMR** (151 MHz, DMSO-d_6) δ 163.5, 161.9, 152.9, 144.7, 139.2, 133.8, 131.2 (d, *J* = 8.5 Hz), 129.6, 129.2, 128.7, 127.5 (d, *J* = 3.1 Hz), 122.0, 118.1, 115.4, 115.3, 72.0, 21.1; **^{19}F NMR** (565 MHz, DMSO-d_6) δ -112.07 (tt, *J* = 9.2, 4.7 Hz); **HRMS** (ESI+, *m*/*z*): calculated for $C_{21}H_{19}FN_2O_3SNa^+$ [M + Na]$^+$: 421.0993, found: 421.0995.

1-phenyl-3-(p-tolyl(tosyl)methyl)urea (S6)

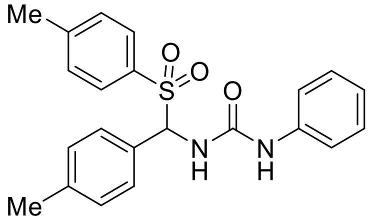

Prepared according to the general procedure A starting from *N*-phenylurea (2.04 g, 15.0 mmol, 1.00 equiv.) and 4-methylbenzaldehyde (2.7 mL, 22.5 mmol, 1.50 equiv.). Product **S6** was obtained as a white solid (5.40 g, 13.7 mmol, 91%).

^{1}H NMR (600 MHz, DMSO-*d*$_6$) δ 8.51 (s, 1H), 7.81 (d, *J* = 10.5 Hz, 1H), 7.73 (d, *J* = 7.9 Hz, 2H), 7.39 – 7.35 (m, 4H), 7.25 (d, *J* = 7.8 Hz, 2H), 7.22 – 7.18 (m, 4H), 6.92 (tt, *J* = 5.9, 2.5 Hz, 1H), 6.16 (d, *J* = 10.6 Hz, 1H), 2.34 (s, 3H), 2.33 (s, 3H); **^{13}C NMR** (151 MHz, DMSO-*d*$_6$) δ 153.0, 144.6, 139.2, 138.9, 134.0, 129.6, 129.1, 128.9, 128.9, 128.7, 128.1, 122.0, 118.0, 72.6, 21.1, 20.8; **HRMS** (ESI+, *m*/*z*): calculated for $C_{22}H_{22}N_2O_3SNa^+$ $[M + Na]^+$: 417.1243, found: 417.1243.

1-((4-methoxyphenyl)(tosyl)methyl)-3-phenylurea (S7)

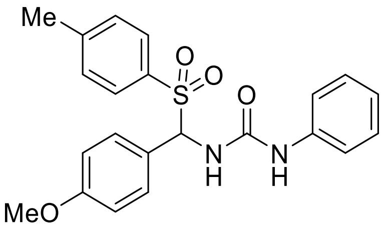

Prepared according to the general procedure A starting from *N*-phenylurea (2.72 g, 20.0 mmol, 1.00 equiv.) and 4-methoxybenzaldehyde (3.7 mL, 30.0 mmol, 1.50 equiv.). Product **S7** was obtained as a white solid (8.13 g, 20 mmol, quantitative).

^{1}H NMR (600 MHz, DMSO-*d*$_6$) δ 8.51 (s, 1H), 7.80 (d, *J* = 10.6 Hz, 1H), 7.73 (d, *J* = 7.9 Hz, 2H), 7.41 (d, *J* = 8.3 Hz, 2H), 7.37 (d, *J* = 8.0 Hz, 2H), 7.26 – 7.11 (m, 4H), 7.01 (d, *J* = 8.3 Hz, 2H), 6.93 (t, *J* = 7.1 Hz, 1H), 6.14 (d, *J* = 10.5 Hz, 1H), 3.78 (s, 3H), 2.34 (s, 3H); **^{13}C NMR** (151 MHz, DMSO-*d*$_6$) δ 160.2, 153.1, 144.7, 139.3, 134.1, 130.5, 129.7, 129.6, 129.2, 128.8, 122.1, 118.2, 113.9, 72.5, 55.4, 21.2. HRMS analysis of this compound failed to provide the expected molecular weight using different ionization techniques, most likely, because of decomposition.

1-((2-hydroxyphenyl)(tosyl)methyl)-3-phenylurea (S8)

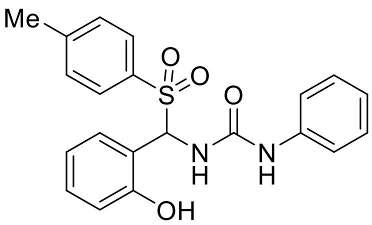

Prepared according to the general procedure A starting from *N*-phenylurea (2.72 g, 20.0 mmol, 1.00 equiv.) and 2-hydroxybenzaldehyde (3.2 mL, 30.0 mmol, 1.50 equiv.). Product **S8** was obtained as a white solid (7.06 g, 17.8 mmol, 89%).

^{1}H NMR (600 MHz, DMSO-*d*$_6$) δ 9.98 (s, 1H), 8.70 (s, 1H), 7.75 (d, *J* = 10.7 Hz, 1H), 7.64 (d, *J* = 7.9 Hz, 2H), 7.35 (d, *J* = 8.0 Hz, 2H), 7.33 (d, *J* = 7.6 Hz, 1H), 7.29 – 7.18 (m, 5H), 6.98 – 6.87 (m, 2H), 6.85 (d, *J* = 8.1 Hz, 1H), 6.58 (d, *J* = 10.4 Hz, 1H), 2.34 (s, 3H); **^{13}C NMR** (151 MHz, DMSO-*d*$_6$) δ 155.8, 153.1, 144.3, 139.4, 134.7, 130.5, 129.5, 129.3, 129.0, 128.7, 121.9, 119.0, 118.0, 118.0, 115.5, 67.8, 21.1. HRMS analysis of this compound failed to provide the expected molecular weight using different ionization techniques, most likely, because of decomposition.

1-(furan-2-yl(tosyl)methyl)-3-phenylurea (S9)

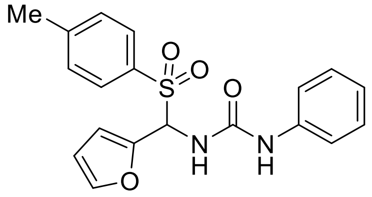

Prepared according to the general procedure A starting from *N*-phenylurea (2.72 g, 20.0 mmol, 1.00 equiv.) and furfural (2.5 mL, 30.0 mmol, 1.50 equiv.). Product **S9** was obtained as a light brown solid (4.28 g, 11.6 mmol, 58%).

^{1}**H NMR** (600 MHz, DMSO-d_6) δ 8.67 (s, 1H), 7.80 – 7.73 (m, 2H), 7.67 (d, *J* = 7.9 Hz, 2H), 7.39 (d, *J* = 8.0 Hz, 2H), 7.29 – 7.17 (m, 4H), 6.99 – 6.85 (m, 1H), 6.63 (d, *J* = 3.4 Hz, 1H), 6.57 – 6.50 (m, 1H), 6.33 – 6.24 (m, 1H), 2.36 (s, 3H); ^{13}**C NMR** (151 MHz, DMSO-d_6) δ 152.9, 144.9, 144.6, 144.5, 139.2, 133.6, 129.7, 129.1, 128.8, 122.1, 118.1, 112.0, 111.2, 68.1, 21.1; **HRMS** (ESI+, *m*/*z*): calculated for $C_{19}H_{18}N_2O_4SNa^+$ $[M + Na]^+$: 393.0880, found 393.0883.

1-(4-chlorophenyl)-3-(phenyl(tosyl)methyl)urea (S10)

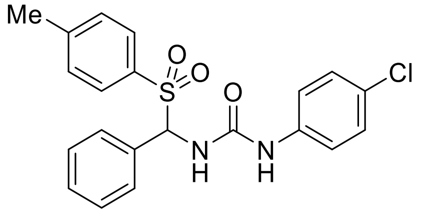

The general procedure A was followed using 4-chlorophenylurea (1.71 g, 10 mmol), *p*-toluenesulfinate (3.56 g, 20 mmol, 2 equiv.), benzaldehyde (1.5 mL, 15 mmol, 1.5 equiv.), formic acid (0.76 mL, 23 mmol, 2 equiv.) in H_2O:MeOH (2:1, 30 mL) at room temperature for 2 days. Product **S10** was obtained as a white solid (3.35 g, 8.08 mmol, 81%).

^{1}**H-NMR** (400 MHz, DMSO-d_6) δ 8.64 (s broad, 1H), 7.89 (d, J = 10.6 Hz, 1H), 7.72 (d, J = 7.9 Hz, 2H), 7.49 – 7.42 (m, 5H), 7.42 – 7.35 (m, 2H), 7.28 – 7.18 (m, 4H), 6.19 (d, J = 10.6 Hz, 1H), 2.35 (s, 3H). ^{13}**C-NMR** (101 MHz, DMSO-d_6) δ 152.9, 144.7, 138.1, 133.8, 131.0, 129.5, 129.4, 129.1, 129.0, 128.5, 128.3, 125.5, 119.6, 72.7, 21.1. **HRMS (ESI)** calculated for $C_{21}H_{19}ClN_2O_3SNa$: 437.06971, found: 437.06802.

1-(4-fluorophenyl)-3-(phenyl(tosyl)methyl)urea (S11)

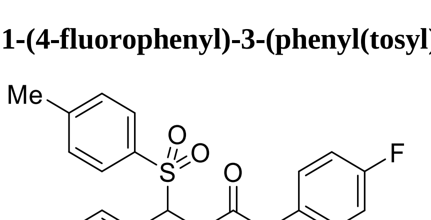

The general procedure A was followed using 4-fluorophenylurea (1.54 g, 10 mmol), *p*-toluenesulfinate (3.56 g, 20 mmol, 2 equiv.), benzaldehyde (1.5 mL, 15 mmol, 1.5 equiv.), formic acid (0.76 mL, 20 mmol, 2 equiv.) in H_2O:MeOH (2:1, 30 mL) at room temperature for 2 days. Product **S11** was obtained as a white solid (3.98 g, 10 mmol, quantitative).

^{1}**H-NMR** (400 MHz, DMSO-d_6) δ 8.53 (s, 1H), 7.84 (d, J = 10.6 Hz, 1H), 7.73 (d, J = 8.0 Hz, 2H), 7.52 – 7.41 (m, 5H), 7.38 (d, J = 7.9 Hz, 2H), 7.24 – 7.14 (m, 2H), 7.09 – 7.01 (m, 2H), 6.20

(d, J = 10.6 Hz, 1H), 2.36 (s, 3H). **^{13}C-NMR** (101 MHz, DMSO-*d*$_6$) δ 158.6, 156.2, 153.0, 144.6, 135.5, 135.5, 133.9, 131.1, 129.5, 129.3, 129.1, 129.1, 129.0, 128.3, 119.9, 119.8, 115.3, 115.1, 72.8, 21.1. **^{19}F-NMR** (376 MHz, DMSO-*d*$_6$) δ -121.27 (tt, J = 8.9, 4.9 Hz). HRMS analysis of this compound failed to provide the expected molecular weight using different ionization techniques, most likely, because of decomposition.

1-(3,5-bis(trifluoromethyl)phenyl)-3-(phenyl(tosyl)methyl)urea (S12)

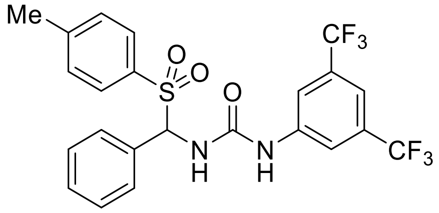

The general procedure A was followed using 3,5-bis(trifluoromethyl)phenylurea (600 mg, 2.2 mmol), *p*-toluenesulfinate (786 mg, 4.4 mmol, 2 equiv.), benzaldehyde (0.33 mL, 3.3 mmol, 1.5 equiv.), formic acid (0.17 mL, 4.4 mmol, 2 equiv.) in H_2O:MeOH (2:1, 6.6 mL) at room temperature for 48 h. Product **S12** was obtained as a white solid (1.02 g, 1.97 mmol, 89%).

^{1}H-NMR (600 MHz, DMSO-*d*$_6$) δ 9.12 (s, 1H), 8.27 (d, *J* = 10.1 Hz, 1H), 7.83 (s, 2H), 7.76 (d, *J* = 7.8 Hz, 2H), 7.66 – 7.28 (m, 8H), 6.23 (d, *J* = 9.8 Hz, 1H), 2.32 (s, 3H). **^{13}C-NMR** (151 MHz, DMSO-*d*$_6$) δ153.0, 144.7, 141.2, 133.9, 130.7 (q, *J* = 32.6 Hz), 130.6, 129.5, 129.5, 129.3, 129.2, 128.4, 123.2 (q, *J* = 272.8 Hz), 118.1, 114.7, 72.8, 20.9. **^{19}F NMR** (565 MHz, DMSO-*d*$_6$) δ -61.79. **HRMS (ESI)** calculated for $C_{23}H_{18}F_6N_2O_3SNa$: 539.08345, found: 539.08156.

1-(phenyl(tosyl)methyl)-3-(*p*-tolyl)urea (S13)

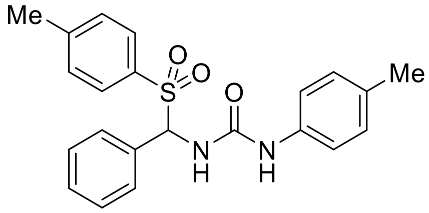

The general procedure A was followed using *p*-tolylurea (1.5 g, 10 mmol), *p*-toluenesulfinate (4.28 g, 24 mmol, 2.4 equiv.), benzaldehyde (1.1 mL, 11 mmol, 1.1 equiv.), formic acid (0.75 mL, 20 mmol, 2 equiv.) in H_2O:MeOH (2:1, 30 mL) at room temperature for 48 h. Product **S13** was obtained as a white solid (3.05 g, 7.73 mmol, 77%).

NOTE: Excess of benzaldehyde is very difficult to remove from the crude mixture by filtration. In this case, the product was dissolved in the minimal amount of DCM (WARNING: we noticed that α-ureidosulfones are very unstable in solution, leading to colored solutions) and rapidly precipitated by addition of an excess of hexane. The white solid formed was filtered and washed with hexane. The resulting white powder was dried under vacuum and used in the next step without further purification.

^{1}H-NMR (400 MHz, DMSO-*d*$_6$) δ 8.41 (s, 1H), 7.80 (d, J = 10.7 Hz, 1H), 7.72 (d, J = 8.3 Hz, 2H), 7.49 – 7.41 (m, 5H), 7.38 (d, J = 8.1 Hz, 2H), 7.07 (d, J = 8.5 Hz, 2H), 7.01 (d, J = 8.4 Hz, 2H), 6.19 (d, J = 10.6 Hz, 1H), 2.36 (s, 3H), 2.20 (s, 3H). **^{13}C-NMR** (101 MHz, DMSO-*d*$_6$) δ 153.0, 144.6, 136.6, 133.9, 131.2, 130.8, 129.5, 129.3, 129.10, 129.07, 129.0, 128.3, 118.2, 72.8, 21.1, 20.3. **HRMS (ESI)** calculated for $C_{22}H_{22}N_2O_3SNa$: 417.12433, found:417.12367.

1-(4-methoxyphenyl)-3-(phenyl(tosyl)methyl)urea (S14)

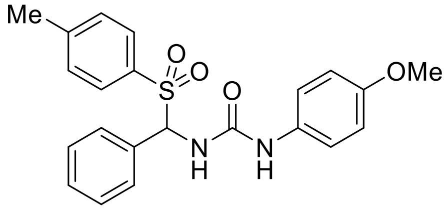

The general procedure A was followed using 4-methoxyphenylurea (1.99 g, 12 mmol), *p*-toluenesulfinate (5.13 g, 28.8 mmol, 2.4 equiv.), benzaldehyde (2.4 mL, 24 mmol, 2 equiv.), formic acid (0.91 mL, 24 mmol, 2 equiv.) in H_2O:MeOH (2:1, 36 mL) at room temperature for 24 h. Product **S14** was obtained as a white solid (4.37 g, 10.6 mmol, 88%).

^{1}H-NMR (600 MHz, DMSO-*d*$_6$) δ 8.31 (s, 1H), 7.75 (d, J = 10.7 Hz, 1H), 7.72 (d, J = 8.3 Hz, 2H), 7.49 – 7.45 (m, 2H), 7.45 – 7.42 (m, 3H), 7.39 (d, J = 8.0 Hz, 2H), 7.08 (d, J = 9.0 Hz, 2H), 6.79 (d, J = 9.0 Hz, 2H), 6.18 (d, J = 10.7 Hz, 1H), 3.68 (s, 3H), 2.37 (s, 3H). **^{13}C-NMR** (151 MHz, DMSO-*d*$_6$) δ 155.0, 153.6, 145.1, 134.5, 132.7, 131.7, 130.0, 129.8, 129.6, 129.5, 128.8, 120.4, 114.4, 73.3, 55.6, 21.6. **HRMS (ESI)** calculated for $C_{22}H_{22}N_2O_4SNa$: 433.11925, found: 433.11825.

1-mesityl-3-(phenyl(tosyl)methyl)urea (S15)

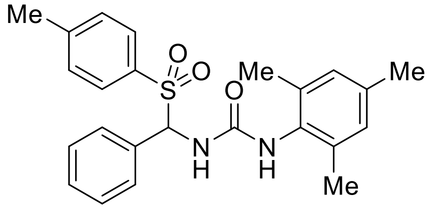

The general procedure A was followed using mesitylurea (2.05 g, 11.5 mmol), *p*-toluenesulfinate (4.1 g, 23 mmol, 2 equiv.), benzaldehyde (1.7 mL, 7.4 mmol, 1.5 equiv.), formic acid (0.87 mL, 23 mmol, 2 equiv.) in H_2O:MeOH (2:1, 24.5 mL) at room temperature for 10 days (shorter times led to incomplete consumption of the starting material). Product **S15** was obtained as a white solid (3.16 g, 7.47 mmol, 65%).

^{1}H-NMR (400 MHz, DMSO-*d*$_6$) δ 7.99 – 7.94 (m, 1H), 7.82 – 7.77 (m, 2H), 7.59 – 7.43 (m, 8H), 6.76 (s, 2H), 6.26 (s, 1H), 2.40 (s, 3H), 2.16 (s, 3H), 1.84 (s, 6H). **^{13}C-NMR** (101 MHz, , DMSO-*d*$_6$) δ 153.4, 144.5, 134.9, 134.8, 134.4, 132.3, 131.3, 129.6, 129.22, 129.17, 129.0, 128.3, 128.1, 72.7, 21.1, 20.4, 17.8. **HRMS (ESI)** calculated for $C_{24}H_{26}N_2O_3SNa$: 445.15563, found: 445.15362.

NOTE: This sulfone proved to be particularly insoluble in a number of solvents. Furthermore, the ^{1}H-NMR signals were broad, most likely, due to rotamers in solution.

1-(naphthalen-2-yl)-3-(phenyl(tosyl)methyl)urea (S16)

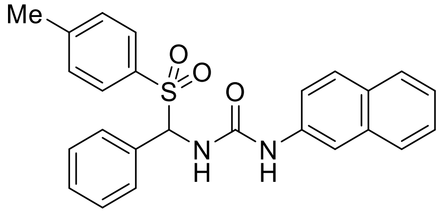

The general procedure A was followed using 2-naphthylurea (920 mg, 4.9 mmol), *p*-toluenesulfinate (1.76 g, 9.9 mmol, 2 equiv.), benzaldehyde (0.75 mL, 7.4 mmol, 1.5 equiv.), formic acid (0.37 mL, 9.9 mmol, 2 equiv.) in H_2O:MeOH (2:1, 15 mL) at room temperature for 10 days (shorter times led to incomplete consumption of the starting material). Product **S16** was obtained as a white solid (1.66 g, 3.86 mmol, 78%).

^{1}H-NMR (400 MHz, DMSO-d_6) δ 8.74 (s, 1H), 8.01 – 7.91 (m, 1H), 7.90 – 7.64 (m, 6H), 7.57 – 7.21 (m, 10H), 6.24 (d, J = 10.6 Hz, 1H), 2.33 (s, 3H). **^{13}C-NMR** (101 MHz, DMSO-d_6) δ 153.1, 144.7, 136.8, 133.9, 133.5, 131.1, 129.6, 129.4, 129.1, 129.0, 128.4, 127.4, 126.8, 126.3, 124.0, 119.5, 113.4, 72.8, 21.1. **HRMS (ESI)** calculated for $C_{25}H_{22}N_2O_3SNa$: 453.12433, found: 453.12224.

7.2. Synthesis and characterization of (hetero)arylideneureas

General method B for the preparation of (hetero)arylideneureas[27]

The corresponding α-ureidosulfone (1 mmol) and pre-dried Cs_2CO_3 (4.5 mmol) were suspended in DCM (24 mL) and stirred 30 min-3 h at room temperature. The reaction mixture was filtered through a Celite® plug (NOTE: Do not rinse the filter cake with more solvent, as this reduces the purity of the final product) and the solvent was removed in vacuo (Note: temperature of water bath above 30ºC should be avoided because of possible imine decomposition). Freshly prepared products were used without further purification for following hydrophosphonylation reactions. Arylidene- and heteroarylideneureas were found to be stable for up to 7 d when stored under high vacuum or inert atmosphere.

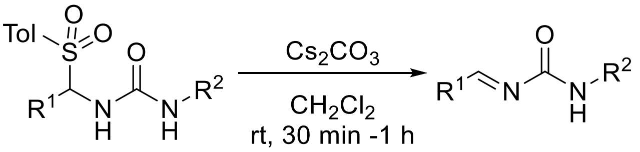

Scheme S5 Preparation of (hetero)arylideneureas.

(*E*)-1-benzylidene-3-phenylurea (6)[28]

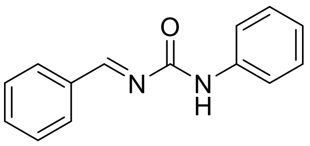

Prepared according to the general procedure B from *α*-ureidosulfone **S3** (1.30 g, 3.42 mmol, 1.00 equiv.). Product **6** was obtained as a white solid (669 mg, 2.98 mmol, 87%).

^{1}H NMR (600 MHz, $CDCl_3$) δ 9.28 (s, 1H), 7.96 (d, J = 7.3 Hz, 2H), 7.82 (s, 1H), 7.65 (d, J = 8.1 Hz, 2H), 7.58 (t, J = 7.4 Hz, 1H), 7.50 (t, J = 7.6 Hz, 2H), 7.37 (t, J = 7.9 Hz, 2H), 7.14 (t, J = 7.4 Hz, 1H); **^{13}C NMR** (151 MHz, $CDCl_3$) δ 170.40, 160.0, 138.0, 134.3, 133.7, 130.6, 129.3, 129.1, 124.5, 119.4; **HRMS** (ESI+, *m*/*z*): calculated for $C_{14}H_{13}N_2O^+$ $[M + H]^+$: 225.1022, found: 225.1019.

---

[27] V. J. Lillo, J. Mansilla, J. M. Saá, *Angew. Chem. Int. Ed.* **2016**, *55*, 4312–4316.

[28] M. Yusuf, S. Thakur, *J. Heterocycl. Chem.* **2019**, *56*, 3403–3413.

(*E*)-1-(4-chlorobenzylidene)-3-phenylurea (S17)

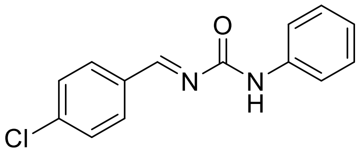

Prepared according to the general procedure B from *α*-ureidosulfone **S4** (539 mg, 1.30 mmol, 1.00 equiv.). Product **S17** was obtained as a white solid (269 mg, 1.04 mmol, 80%).

^{1}H NMR (600 MHz, $CDCl_3$) δ 9.22 (s, 1H), 7.88 (d, *J* = 8.5 Hz, 2H), 7.81 (s, 1H), 7.64 (d, *J* = 7.4 Hz, 2H), 7.46 (d, *J* = 8.5 Hz, 2H), 7.37 (t, *J* = 7.9 Hz, 2H), 7.15 (t, *J* = 7.4 Hz, 1H); **^{13}C NMR** (151 MHz, $CDCl_3$) δ 168.7, 159.5, 139.8, 137.7, 132.5, 131.4, 129.3, 129.1, 124.4, 119.2; **HRMS** (ESI+, *m*/*z*): calculated for $C_{14}H_{12}ClN_2O^+$ $[M + H]^+$: 259.0633, found: 259.0634.

(*E*)-1-(4-fluorobenzylidene)-3-phenylurea (S18)

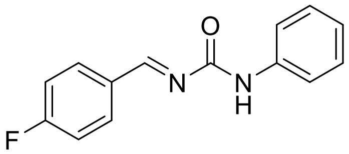

Prepared according to the general procedure B from *α*-ureidosulfone **S5** (996 mg, 2.50 mmol, 1.00 equiv.). Product **S18** was obtained as a white solid (422 mg, 1.74 mmol, 70%).

^{1}H NMR (600 MHz, $CDCl_3$) δ 9.24 (s, 1H), 7.98 (dd, *J* = 8.5, 5.6 Hz, 2H), 7.73 (s, 1H), 7.64 (d, *J* = 8.0 Hz, 2H), 7.37 (t, *J* = 7.8 Hz, 2H), 7.19 (t, *J* = 8.5 Hz, 2H), 7.15 (t, *J* = 7.4 Hz, 1H); **^{13}C NMR** (151 MHz, $CDCl_3$) δ 168.9, 166.2 (d, *J* = 256.0 Hz), 159.8, 138.0, 132.9 (d, *J* = 9.3 Hz), 130.6 (d, *J* = 3.0 Hz), 129.3, 124.5, 119.4, 116.5 (d, *J* = 22.2 Hz); **^{19}F NMR** (565 MHz, $CDCl_3$) δ -103.90 – -103.99 (m); **HRMS** (ESI+, *m*/*z*): calculated for $C_{14}H_{12}FN_2O^+$ $[M + H]^+$: 243.0928, found: 243.0928.

(*E*)-1-(4-methylbenzylidene)-3-phenylurea (S19)[10]

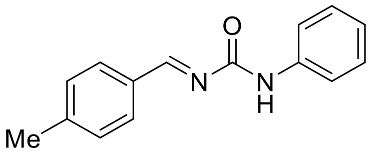

Prepared according to the general procedure B from *α*-ureidosulfone **S6** (986 mg, 2.50 mmol, 1.00 equiv.). Product **S19** was obtained as a white solid (441 mg, 1.85 mmol, 74%).

^{1}H NMR (600 MHz, $CDCl_3$) δ 9.24 (s, 1H), 7.86 (d, *J* = 7.9 Hz, 2H), 7.71 (s, 1H), 7.64 (d, *J* = 8.0 Hz, 2H), 7.37 (t, *J* = 7.9 Hz, 2H), 7.31 (d, *J* = 7.8 Hz, 2H), 7.14 (t, *J* = 7.4 Hz, 1H), 2.45 (s, 3H); **^{13}C NMR** (151 MHz, $CDCl_3$) δ 170.3, 160.2, 144.8, 138.1, 131.7, 130.7, 129.9, 129.3, 124.4, 119.4, 22.0; **HRMS** (ESI+, *m*/*z*): calculated for $C_{15}H_{15}N_2O^+$ $[M + H]^+$: 239.1179, found: 239.1177.

(*E*)-1-(4-methoxylbenzylidene)-3-phenylurea (S20)[10]

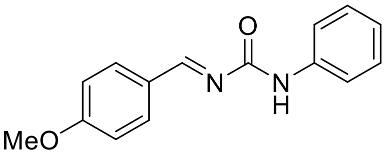

Prepared according to the general procedure B from *α*-ureidosulfone **S7** (411 mg, 1.00 mmol, 1.00 equiv.). Product **S20** was obtained as a white solid (226 mg, 0.89 mmol, 89%).

^{1}H NMR (600 MHz, $CDCl_3$) δ 9.22 (s, 1H), 7.91 (s, 1H), 7.90 (d, *J* = 8.7 Hz, 2H), 7.64 (d, *J* = 7.9 Hz, 2H), 7.35 (t, *J* = 8.0 Hz, 2H), 7.12 (t, *J* = 7.4 Hz, 1H), 6.97 (d, *J* = 8.7 Hz, 2H), 3.87 (s, 3H); **^{13}C NMR** (151 MHz, $CDCl_3$) δ 169.5, 164.2, 160.4, 138.3, 132.7, 129.2, 127.1, 124.2, 119.3, 114.6, 55.6; **HRMS** (ESI+, *m*/*z*): calculated for $C_{15}H_{15}N_2O_2^+$ [M + H]$^+$: 255.1128, found: 255.1127.

(*E*)-1-(2-hydroxylbenzylidene)-3-phenylurea (S21)[10]

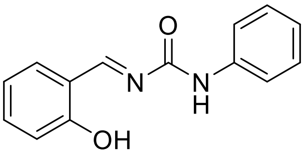

Prepared according to the general procedure B from *α*-ureidosulfone **S8** (515 mg, 1.30 mmol, 1.00 equiv.). Product **S21** was obtained as a yellow solid (244 mg, 1.02 mmol, 78%).

^{1}H NMR (600 MHz, $CDCl_3$) δ 12.32 (s, 1H), 9.38 (s, 1H), 7.64 (d, *J* = 8.5 Hz, 2H), 7.61 (s, 1H), 7.54 (dd, *J* = 7.7, 1.7 Hz, 1H), 7.51 – 7.47 (m, 1H), 7.39 (dd, *J* = 8.5, 7.4 Hz, 2H), 7.17 (tt, *J* = 7.4, 1.1 Hz, 1H), 7.05 (dd, *J* = 8.4, 1.0 Hz, 1H), 7.01 (td, *J* = 7.5, 1.1 Hz, 1H); **^{13}C NMR** (151 MHz, $CDCl_3$) δ 172.6, 162.6, 157.4, 137.6, 136.0, 135.2, 129.4, 124.9, 120.1, 119.6, 117.8, 117.6; **HRMS** (ESI+, *m*/*z*): calculated for $C_{14}H_{13}N_2O_2^+$ [M + H]$^+$: 241.0972, found: 241.0973.

(*E*)-1-(furan-2-ylmethylene)-3-phenylurea (S22)

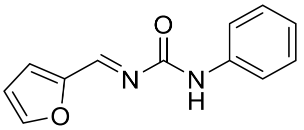

Prepared according to the general procedure B from *α*-ureidosulfone **S9** (370 mg, 1.00 mmol, 1.00 equiv.). Product **S22** was obtained as a white solid (168 mg, 0.78 mmol, 78%).

^{1}H NMR (600 MHz, $CDCl_3$) δ 9.07 (s, 1H), 7.88 (s, 1H), 7.71 (dd, *J* = 1.7, 0.8 Hz, 1H), 7.61 (d, *J* = 8.5 Hz, 2H), 7.36 (dd, *J* = 8.6, 7.4 Hz, 2H), 7.23 (dd, *J* = 3.5, 0.8 Hz, 1H), 7.13 (tt, *J* = 7.4, 1.1 Hz, 1H), 6.64 (dd, *J* = 3.5, 1.7 Hz, 1H); **^{13}C NMR** (151 MHz, $CDCl_3$) δ 159.3, 156.3, 150.7, 148.1, 137.8, 129.1, 124.3, 122.9, 119.1, 113.1; **HRMS** (ESI+, *m*/*z*): calculated for $C_{12}H_{11}N_2O_2^+$ [M + H]$^+$: 215.0815, found: 215.0810.

(*E*)-1-benzylidene-3-(4-chlorophenyl)urea (S23)

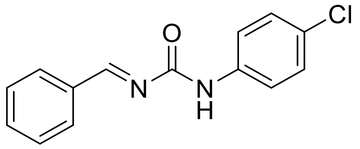

The general procedure B was followed using sulfone **S10** (830 mg, 2 mmol), Cs_2CO_3 (2.93 g, 9 mmol, 4.5 equiv.) in DCM (40 mL) at room temperature for 1 h. Product **S23** was obtained as a white foam (397 mg, 1.535 mmol, 77%).

^{1}H-NMR (400 MHz, $CDCl_3$) δ 9.27 (s, 1H), 7.96 (d, J = 7.3 Hz, 2H), 7.80 (s broad, 1H), 7.64 – 7.56 (m, 3H), 7.50 (t, J = 7.6 Hz, 2H), 7.32 (d, J = 8.7 Hz, 2H). **^{13}C-NMR** (151 MHz, CDCl3) δ 170.8, 160.0, 136.6, 134.1, 133.9, 130.6, 129.4, 129.3, 129.2, 120.6. **HRMS (ESI)** calculated for $C_{14}H_{11}N_2OClNa$: 281.04521, found: 281.04440.

(*E*)-1-benzylidene-3-(4-fluorophenyl)urea (S24)

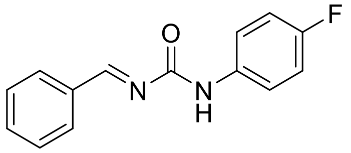

The general procedure B was followed using sulfone **S11** (797 mg, 2 mmol), Cs_2CO_3 (2.93 g, 9 mmol, 4.5 equiv.) in DCM (40 mL) at room temperature for 1 h. Product **S24** was obtained as a white solid (195 mg, 0.81 mmol, 40%).

^{1}H-NMR (400 MHz, $CDCl_3$) δ 9.27 (s, 1H), 7.96 (d, J = 6.9 Hz, 2H), 7.77 (s broad, 1H), 7.64 – 7.56 (m, 3H), 7.50 (apparent t, J = 7.4 Hz, 2H), 7.06 (apparent t, J = 8.7 Hz, 2H). **^{13}C-NMR** (101 MHz, $CDCl_3$) δ 170.6, 160.1, 159.5 (d, J = 243.6 Hz), 134.2, 134.1 (d, J = 2.6 Hz), 133.8, 130.6, 129.1, 121.1 (d, J = 7.8 Hz), 115.9 (d, J = 22.5 Hz). **^{19}F NMR** (376 MHz, $CDCl_3$) δ -118.04 (tt, J = 8.8, 4.8 Hz). **HRMS (ESI)** calculated for $C_{14}H_{10}N_2OF$: 242.08162, found: 242.08127.

(*E*)-1-benzylidene-3-(3,5-bis(trifluoromethyl)phenyl)urea (S25)

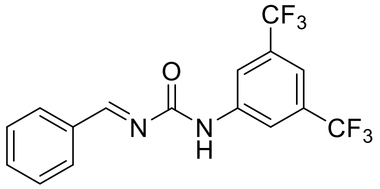

The general procedure B was followed using sulfone **S12** (517 mg, 1 mmol), Cs_2CO_3 (1.47 g, 4.5 mmol, 4.5 equiv.) in DCM (20 mL) at room temperature for 1 h. Product **S25** was obtained as a pale yellow solid (250 mg, 0.69 mmol, 69%).

^{1}H-NMR (600 MHz, $CDCl_3$) δ 9.28 (s, 1H), 8.15 (s, 2H), 8.07 (s, 1H), 7.97 (d, J = 7.2 Hz, 2H), 7.64 – 7.60 (m, 2H), 7.52 (t, J = 7.7 Hz, 2H). **^{13}C-NMR** (151 MHz, $CDCl_3$) δ 171.9, 160.3, 139.6, 134.3, 133.9, 132.7 (q, J = 33.5 Hz), 130.9, 129.3, 123.2 (q, J = 272.8 Hz), 119.0 (d, J = 4.1 Hz), 117.7 (p, J = 3.7 Hz). **^{19}F NMR** (565 MHz, $CDCl_3$) δ -63.05. **HRMS (ESI)** calculated $C_{16}H_{11}F_6N_2O^+$ $[M + H]^+$: 371.0770, found:371.0770.

(*E*)-1-benzylidene-3-(p-tolyl)urea (S26)

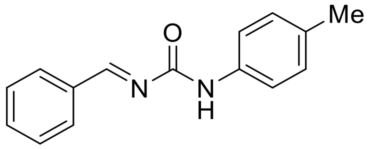

The general procedure B was followed using sulfone **S13** (789 mg, 2 mmol), Cs_2CO_3 (2.93 g, 9 mmol, 4.5 equiv.) in DCM (40 mL) at room temperature for 1 h. Product **S26** was obtained as a pale yellow solid (218 mg, 0.91 mmol, 46%).

^{1}H-NMR (400 MHz, $CDCl_3$) δ 9.27 (s, 1H), 7.96 (d, J = 8.5 Hz, 2H), 7.72 (s broad, 1H), 7.61 – 7.46 (m, 5H), 7.17 (d, J = 8.1 Hz, 2H), 2.34 (s, 3H). **^{13}C-NMR** (101 MHz, $CDCl_3$) δ 170.2, 159.9, 135.5, 134.3, 134.1, 133.6, 130.5, 129.8, 129.1, 119.4, 21.0. **HRMS (ESI)** calculated for $C_{15}H_{14}N_2ONa$: 261.09983, found: 261.09924.

(*E*)-1-benzylidene-3-(4-methoxyphenyl)urea (S27)

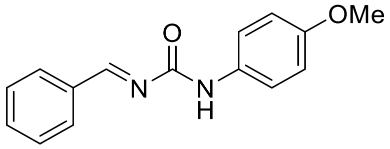

The general procedure B was followed using sulfone **S14** (821 mg, 2 mmol), Cs_2CO_3 (2.93 g, 9 mmol, 4.5 equiv.) in DCM (40 mL) at room temperature for 1 h. Product **S27** was obtained as a yellow solid (378 mg, 1.49 mmol, 74%).

^{1}H-NMR (600 MHz, $CDCl_3$) δ 9.27 (s, 1H), 7.98 – 7.92 (m, 2H), 7.68 (s, 1H), 7.60 – 7.54 (m, 3H), 7.50 (t, J = 7.7 Hz, 2H), 6.93 – 6.89 (m, 2H), 3.81 (s, 3H). **^{13}C-NMR** (151 MHz, $CDCl_3$) δ 170.0, 159.9, 156.6, 134.4, 133.6, 131.2, 130.5, 129.1, 121.1, 114.5, 55.6. **HRMS (ESI)** calculated for $C_{15}H_{15}N_2O_2$ [M^+ + 1]: 255.11280, found: 255.11250.

(*E*)-1-benzylidene-3-mesitylurea (S28)

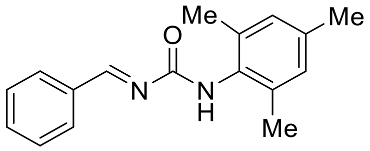

The general procedure B was followed using sulfone **S15** (423 mg, 1 mmol), Cs_2CO_3 (1.47 g, 4.5 mmol, 4.5 equiv.) in DCM (20 mL) at room temperature for 1.5 h. Product **S28** was obtained as a white solid (123 mg, 0.46 mmol, 46%).

^{1}H-NMR (400 MHz, $CDCl_3$) δ 9.28 (s, 1H), 7.98 (d, J = 7.4 Hz, 2H), 7.62 – 7.56 (m, 1H), 7.51 (t, J = 7.5 Hz, 2H), 7.08 (s broad, 1H), 6.95 (s, 2H), 2.29 (s, 3H), 2.28 (s, 6H). **^{13}C-NMR** (151 MHz, $CDCl_3$) δ 170.2, 161.1, 137.3, 135.4, 134.4, 133.5, 131.0, 130.5, 129.14, 129.07, 21.1, 18.5. **HRMS (ESI)** calculated for $C_{17}H_{19}N_2O$ [M^+ + 1]: 267.14919, found: 267.14906.

(*E*)-1-benzylidene-3-(naphthalen-2-yl)urea (S29)

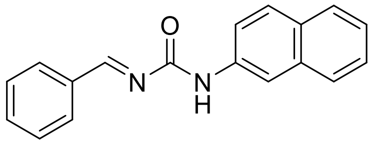

The general procedure B was followed using sulfone **S16** (431 mg, 1 mmol), Cs_2CO_3 (1.47 g, 4.5 mmol, 4.5 equiv.) in DCM (20 mL) at room temperature for 1 h. Product **S29** was obtained as a yellow solid (183 mg, 0.66 mmol, 66%).

^{1}H-NMR (400 MHz, $CDCl_3$) δ 9.33 (s, 1H), 8.34 (d, J = 2.2 Hz, 1H), 7.99 (d, J = 6.8 Hz, 2H), 7.91 (s broad, 1H), 7.86 – 7.76 (m, 3H), 7.64 – 7.56 (m, 2H), 7.55 – 7.45 (m, 3H), 7.46 – 7.38 (m, 1H). **^{13}C-NMR** (151 MHz, $CDCl_3$) δ 170.5, 160.2, 135.4, 134.3, 134.0, 133.8, 130.8, 130.6, 129.2, 129.1, 127.9, 127.7, 126.7, 125.2, 119.5, 116.1. **HRMS (ESI)** calculated for $C_{18}H_{14}N_2ONa$: 297.09983, found: 297.09897.

7.3. Synthesis and characterization of α-ureidophosphonates

General procedure C for the enantioselective hydrophosphonylation of (hetero)arylidene ureas

Freshly prepared arylidene urea (0.2 mmol, 1.00 equiv.) and TiPSY catalyst (8.7 mg, 0.01 mmol, 5 mol%) were dissolved in anhydrous, deoxygenated toluene (1.7 mL). At this point, a pale yellow to intense yellow solution was usually obtained. A stock solution of the corresponding phosphite (0.24 mmol, 1.2 equiv.) in toluene (0.3 mL) was added dropwise over period of 1 min to the reaction mixture. The solution was stirred at 6 °C or 30 °C for the specified time. After full conversion of imine, a thick white suspension was often obtained. The *e.r.* was determined at this point by taking an aliquot (100 µL) of the reaction mixture and diluting it with $CDCl_3$ (0.4-0.5 mL) and analyzing it by ^{31}P{^{1}H}-NMR and/or ^{1}H-NMR. After reaction completion, the solvent was removed in vacuo and the crude material was purified by column chromatography using the specified eluent.

NOTE: As precaution, the enantiomeric purity (*e.r.*) was determined before and after the purification by column chromatography.

General procedure D for the 2 mmol-scale preparation of 5

Starting from α-ureidosulfone **S3** (2.00 mmol), arylidene urea **6** was prepared according to general procedure B. The resulting arylidene urea **6** (1.00 equiv.) and (*S*)-TiPSY catalyst (2 mol%) were dissolved in anhydrous, deoxygenated toluene (17 mL). A stock solution of dimethyl phosphite (1.1 equiv.) in toluene (3.0 mL) was added dropwise over period of 1 min to the reaction mixture. The yellow solution was stirred at 30 °C for 2 d. After reaction completion, a white suspension was obtained and the solvent was removed in vacuo and the crude material was purified by column chromatography using the specified eluent.

Diethyl (*S*)-(phenyl(3-phenylureido)methyl)phosphonate ((*S*)-3)

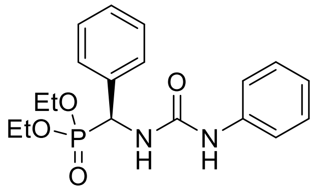

The general procedure C was followed using arylidene urea **6** (44.9 mg, 0.2 mmol), diethyl phosphite (30 µL, 0.233 mmol, 1.2 equiv.), *S*-TiPSY (8.7 mg, 0.01 mmol, 0.05 equiv.) in PhMe (2 mL) at 30 °C for 48 h. Purification by flash column chromatography using hexane/^{i}PrOH 9:1. Product (*S*)**-3** was obtained as a white solid (46 mg, 63%).

^{1}H-NMR (400 MHz, DMSO-*d*$_6$) δ 8.69 (s, 1H), 7.42 – 7.33 (m, 6H), 7.35 – 7.26 (m, 1H), 7.28 – 7.18 (m, 3H), 6.94 – 6.87 (m, 1H), 5.23 (dd, J = 21.9, 9.7 Hz, 1H), 4.13 – 3.97 (m, 2H), 3.95 – 3.84 (m, 1H), 3.83 – 3.69 (m, 1H), 1.21 (t, J = 7.0 Hz, 3H), 1.06 (t, J = 7.0 Hz, 3H). **^{13}C-NMR** (151 MHz, DMSO-*d*$_6$) δ 154.1 (d, J = 10.6 Hz), 139.9, 136.7, 128.8, 128.2 (d, J = 2.1 Hz), 127.7 (d, J = 5.4 Hz), 127.6 (d, J = 2.7 Hz), 121.5, 117.5, 62.5 (d, J = 7.0 Hz), 62.4 (d, J = 7.0 Hz), 50.0 (d, J = 153.2 Hz), 16.3 (d, J = 5.4 Hz), 16.0 (d, J = 5.9 Hz). **^{31}P{^{1}H}-NMR** (162 MHz, DMSO-*d*$_6$) δ 22.43. **HRMS (ESI)** calculated for $C_{18}H_{23}N_2O_4PNa$: 385.12876, found: 385.12787.

Enantiomeric purity: 94:6 *e.r.* (crude, ^{31}P{^{1}H}-NMR in $CDCl_3$) 92.5:7.5 *e.r.* (after purification, ^{31}P{^{1}H}-NMR in $CDCl_3$). 92.5:7.5 *e.r.* (by chiral HPLC, Lux Cellulose-4, 85:15 heptane/^{i}PrOH, 1 mL/min, 25 min, λ_{abs} = 240 nm, t_R(*S*) = 7.906 min, t_R(*R*) = 17.429 min).

$[\boldsymbol{\alpha}]_{\boldsymbol{D}}^{\mathbf{20}}$ = –5.4 (c = 2.08, DMSO)

Dibenzyl (*S*)-(phenyl(3-phenylureido)methyl)phosphonate ((*S*)-4)

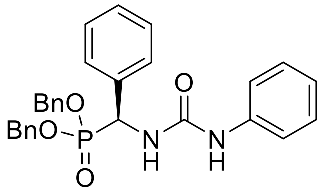

The general procedure C was followed using arylidene urea **6** (44.9 mg, 0.20 mmol, 1.00 equiv.), dibenzyl phosphite (62.9 mg, 0.24 mmol, 1.20 equiv.) and (*S*)-TiPSY catalyst (8.7 mg, 0.01 mmol, 5 mol%) in PhMe (2 mL) at 30 °C for 72 h. The crude material was purified by column chromatography (hexane/^{i}PrOH 95:5). Product (*S*)**-4** was obtained as a white solid (67.0 mg, 0.14 mmol, 69%).

^{1}H NMR (600 MHz, DMSO-*d*$_6$) δ 8.68 (s, 1H), 7.44 (d, *J* = 7.6 Hz, 2H), 7.41 – 7.33 (m, 5H), 7.35 – 7.25 (m, 9H), 7.23 (t, *J* = 7.8 Hz, 2H), 7.20 – 7.15 (m, 2H), 6.92 (t, *J* = 7.4 Hz, 1H), 5.45 (dd, *J* = 21.8, 9.6 Hz, 1H), 5.04 (d, *J* = 7.7 Hz, 2H), 4.92 (dd, *J* = 12.1, 6.8 Hz, 1H), 4.78 (dd, *J* = 12.0, 8.0 Hz, 1H); **^{13}C NMR** (151 MHz, DMSO-*d*$_6$) δ 154.1 (d, *J* = 10.2 Hz), 139.8, 136.32, 136.28, 136.2 (d, *J* = 6.5 Hz), 128.8, 128.37, 128.36 (d, *J* = 7.5 Hz), 128.2 (d, *J* = 10.9 Hz), 127.9 (d, *J* = 5.6 Hz), 127.8, 127.7, 127.5, 121.6, 117.6, 67.7 (d, *J* = 6.7 Hz), 67.4 (d, *J* = 6.7 Hz), 50.1 (d, *J* = 153.1 Hz); **^{31}P NMR** (162 MHz, DMSO-*d*$_6$) δ 23.31; **HRMS** (ESI+, *m*/*z*): calculated for $C_{28}H_{28}N_2O_4P^+$ [M + H]$^+$: 487.1781, found: 487.1769.

Enantiomeric purity: 74:26 (crude, ^{31}P{^{1}H}-NMR in $CDCl_3$), 75:25 (after purification, ^{31}P{^{1}H}-NMR in $CDCl_3$).

$[\boldsymbol{\alpha}]_{\boldsymbol{D}}^{\mathbf{20}}$ = 6.0 (c = 0.30, DMSO).

Dimethyl (*S*)-(phenyl(3-phenylureido)methyl)phosphonate ((*S*)-5)

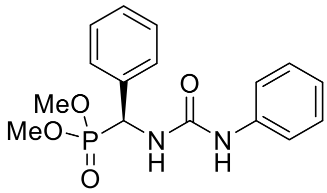

The general procedure C was followed using arylidene urea **6** (44.9 mg, 0.20 mmol, 1.00 equiv.), dimethyl phosphite (22 µL, 0.24 mmol, 1.20 equiv.) and (*S*)-TiPSY catalyst (8.7 mg, 0.01 mmol, 5 mol%) in PhMe (2 mL) at 30 °C for 32 h. The crude material was purified by column chromatography (hexane/^{i}PrOH 90:10). Product (*S*)-**5** was obtained as a white solid (59.0 mg, 0.18 mmol, 88%).

2 mmol-scale: Prepared according to the general procedure D from α-ureidosulfone **S3** (2.00 mmol). Resulting arylidene urea **6** (376 mg, 1.68 mmol, 1.00 equiv.) was used in the subsequent reaction with dimethyl phosphite (170 µL, 1.85 mmol, 1.1 equiv.) in presence of (*S*)-TiPSY catalyst (29 mg, 0.034 mmol, 2 mol%). The crude material was purified by column chromatography (hexane/^{i}PrOH 90:10). Product (*S*)-**5** was obtained as a white solid (540 mg, 1.62 mmol, 81% over two steps) For *e.r.* see section SDE on achiral column chromatography.

^{1}H NMR (600 MHz, DMSO-d_6) δ 8.66 (s, 1H), 7.43 – 7.33 (m, 6H), 7.34 – 7.27 (m, 2H), 7.25 – 7.18 (m, 2H), 6.91 (tt, *J* = 7.3, 1.2 Hz, 1H), 5.31 (dd, *J* = 21.8, 9.7 Hz, 1H), 3.69 (d, *J* = 10.6 Hz, 3H), 3.50 (d, *J* = 10.5 Hz, 3H); **^{13}C NMR** (151 MHz, DMSO-d_6) δ 154.1 (d, *J* = 10.4 Hz), 139.8, 136.5, 128.8, 128.4 (d, *J* = 2.1 Hz), 127.72, 127.68, 121.6, 117.6, 53.4 (d, *J* = 7.0 Hz), 53.2 (d, *J* = 7.0 Hz), 49.4 (d, *J* = 153.3 Hz); **^{31}P{^{1}H}-NMR** (162 MHz, DMSO-d_6) δ 24.70; **HRMS** (ESI+, *m*/*z*): calculated for $C_{16}H_{19}N_2O_4PNa^+$ [M + Na]$^+$: 357.0975, found: 357.0979.

Enantiomeric purity: 93:7 *e.r.* (crude, ^{31}P{^{1}H}-NMR in $CDCl_3$), 94:6 *e.r.* (after purification, ^{31}P{^{1}H}-NMR in $CDCl_3$), 92:8 (SFC Chiralpak ID, ^{i}PrOH 1-40%, 20 min, 4 mL/min, λ_{abs} = 240 nm, t_R(*R*) = 13.8 min, t_R(*S*) = 14.9 min).

$[\alpha]_D^{20}$ = 16.2 (c = 0.72, DMSO).

Diisopropyl (*S*)-(phenyl(3-phenylureido)methyl)phosphonate ((*S*)-7)

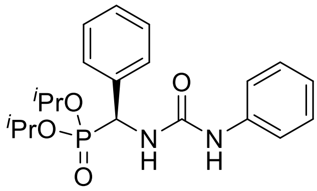

The general procedure C was followed using arylidene urea **6** (44.9 mg, 0.20 mmol, 1.00 equiv.), diisopropyl phosphite (40 µL, 0.24 mmol, 1.20 equiv.) and (*S*)-TiPSY catalyst (8.7 mg, 0.01 mmol, 5 mol%) in PhMe (1 mL) at 30 °C for 96 h. The crude material was purified by column chromatography (hexane/^{i}PrOH 95:5). Product (*S*)-**7** was obtained as a white solid (56.0 mg, 0.14 mmol, 72%).

^{1}H NMR (600 MHz, MeOD-d_4) δ 7.46 (dd, *J* = 7.6, 2.1 Hz, 2H), 7.38 (t, *J* = 7.6 Hz, 2H), 7.37 – 7.30 (m, 3H), 7.25 (t, *J* = 7.9 Hz, 2H), 6.99 (d, *J* = 7.3 Hz, 1H), 5.32 (d, *J* = 22.5 Hz, 1H), 4.74 – 4.63 (m, 1H), 4.58 – 4.48 (m, 1H), 1.35 (d, *J* = 6.1 Hz, 3H), 1.29 (d, *J* = 6.2 Hz, 3H), 1.27 (d, *J* = 6.2 Hz, 3H), 1.09 (d, *J* = 6.2 Hz, 3H); **^{13}C NMR** (151 MHz, MeOD-d_4) 156.8 (d, *J* = 9.6 Hz), 140.5, 137.3, 129.9, 129.5 (d, *J* = 2.2 Hz), 129.2 (d, *J* = 5.8 Hz), 129.1 (d, *J* = 3.0 Hz), 123.7, 120.0, 73.8 (d, *J* = 7.6 Hz), 73.7 (d, *J* = 7.3 Hz), 52.5 (d, *J* = 157.5 Hz), 24.43 (d, *J* = 5.9 Hz),

24.39 (d, J = 5.2 Hz), 24.0 (d, J = 5.3 Hz), 23.8 (d, J = 5.4 Hz); **^{31}P NMR** (162 MHz, MeOD-d_4) δ 23.38; **HRMS** (ESI+, *m/z*): calculated for $C_{20}H_{28}N_2O_4P^+$ $[M + H]^+$: 391.1781, found: 391.1777.

Enantiomeric purity: 77:23 (crude, ^{31}P{^{1}H}-NMR in $CDCl_3$), 79.5:20.5 (after purification, ^{31}P{^{1}H}-NMR in $CDCl_3$).

HPLC Lux Cellulose-4, *n*-heptane/^{i}PrOH 90:10, 40 °C, 1 mL/min, λ_{abs} = 240 nm, $t_R(S)$ = 7.9 min, $t_R(R)$ = 10.5 min.

$[\boldsymbol{\alpha}]_D^{20}$ = –8.3 (c = 1.40, MeOH).

Didodecyl (*S*)-(phenyl(3-phenylureido)methyl)phosphonate ((*S*)-8)

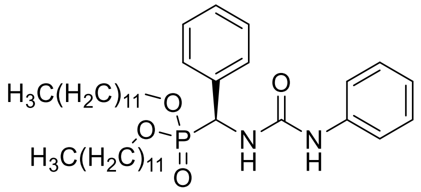

The general procedure C was followed using arylidene urea **6** (44.9 mg, 0.2 mmol), didodecyl phosphite (100.5 mg, 0.233 mmol, 1.2 equiv.), *S*-TiPSY (8.7 mg, 0.01 mmol, 0.05 equiv.) in PhMe (2 mL) at 30 °C for 63 h. Purification by flash column chromatography using hexane/^{i}PrOH 98:2 afforded product (*S*)-**8** as a tan solid (42.3 mg, 0.066 mmol, 33%).

^{1}H-NMR (600 MHz, DMSO) δ 8.66 (s, 1H), 7.42 – 7.32 (m, 6H), 7.31 – 7.27 (m, 1H), 7.26 – 7.18 (m, 3H), 6.92 – 6.86 (m, 1H), 5.24 (dd, J = 21.9, 9.5 Hz, 1H), 4.03 – 3.91 (m, 2H), 3.86 – 3.78 (m, 1H), 3.70 – 3.62 (m, 1H), 1.26 – 1.13 (m, 46H). **^{13}C-NMR** (151 MHz, DMSO) δ 154.0 (d, J = 10.3 Hz), 139.9, 136.7, 128.7, 128.2, 127.7 (d, J = 5.4 Hz), 127.5, 124.3 (d, J = 11.9 Hz), 121.4, 118.4, 117.5, 66.4 (d, J = 7.1 Hz), 66.1 (d, J = 7.0 Hz), 49.9 (d, J = 153.5 Hz), 34.5, 34.3, 31.3, 31.1, 30.3, 30.0, 30.0, 29.8, 29.8, 29.05, 29.02, 9.0, 28.95, 28.92, 28.7, 28.6, 28.5, 25.0, 24.8, 22.1, 13.9. **^{31}P{^{1}H}-NMR** (243 MHz, DMSO-d_6) δ 22.45. **HRMS (ESI)** calculated for $C_{38}H_{64}N_2O_4P$ $[M^+ + 1]$: 643.45982, found: 643.45915.

Enantiomeric purity: 66:34 *e.r.* (crude, ^{31}P{^{1}H}-NMR in $CDCl_3$) 65:35 *e.r.* (after purification, ^{31}P{^{1}H}-NMR in $CDCl_3$).

NOTE: Specific optical rotation could not be determined for this compound due to solubility problems under the conditions required for the measurement.

(*S*)-1-((5,5-dimethyl-2-oxido-1,3,2-dioxaphosphinan-2-yl)(phenyl)methyl)-3-phenylurea ((*S*)-9)

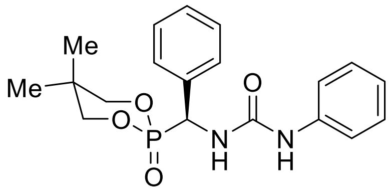

The general procedure C was followed using arylidene urea **6** (44.9 mg, 0.20 mmol, 1.00 equiv.), neopentylene phosphite (36.0 mg, 0.24 mmol, 1.20 equiv.) and (*S*)-TiPSY catalyst (8.7 mg, 0.01 mmol, 5 mol%) in PhMe (2 mL) at 6 °C for 10 h. The crude material was purified by column chromatography (hexane/^{i}PrOH 95:5). Product (*S*)-**9** was obtained as a white solid (60.5 mg, 0.16 mmol, 81%).

^{1}H NMR (600 MHz, DMSO-d_6) δ 8.67 (s, 1H), 7.52 – 7.47 (m, 2H), 7.41 – 7.34 (m, 4H), 7.34 – 7.27 (m, 2H), 7.23 (dd, J = 8.6, 7.3 Hz, 2H), 6.92 (tt, J = 7.4, 1.2 Hz, 1H), 5.71 (dd, J = 17.4, 9.7

Hz, 1H), 4.45 (dd, J = 11.0, 2.8 Hz, 1H), 4.37 (dd, J = 11.1, 3.1 Hz, 1H), 3.96 (ddd, J = 19.4, 11.1, 2.7 Hz, 1H), 3.87 (ddd, J = 19.0, 10.9, 2.7 Hz, 1H), 1.13 (s, 3H), 0.88 (s, 3H); **^{13}C NMR** (151 MHz, DMSO-d_6) δ 154.2 (d, J = 8.8 Hz), 139.9, 136.5, 128.8, 128.4, 127.9 (d, J = 5.4 Hz), 127.7 (d, J = 2.5 Hz), 121.6, 117.6, 76.4 (t, J = 6.1 Hz), 46.4 (d, J = 143.0 Hz), 40.1, 32.0 (d, J = 7.0 Hz), 21.3, 19.6; **^{31}P{^{1}H}-NMR** (162 MHz, DMSO-d_6) δ 15.46; **HRMS** (ESI+, m/z): calculated for $C_{19}H_{24}N_2O_4P^+$ [M + H]$^+$: 375.1468, found: 375.1469.

Enantiomeric purity: 87:13 *e.r.* (crude, ^{31}P{^{1}H}-NMR in $CDCl_3$), 87:13 *e.r.* (after purification, ^{31}P{^{1}H}-NMR in $CDCl_3$).

$[\alpha]_D^{20}$ = 8.3 (c = 0.34, DMSO).

Diphenyl (*S*)-(phenyl(3-phenylureido)methyl)phosphonate ((*S*)-10)

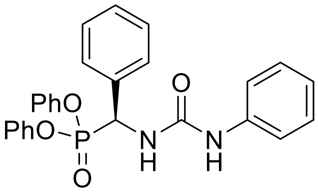

The general procedure C was followed using arylidene urea **6** (44.9 mg, 0.20 mmol, 1.00 equiv.), diphenyl phosphite (46 µL, 0.24 mmol, 1.20 equiv.) and (*S*)-TiPSY catalyst (8.7 mg, 0.01 mmol, 5 mol%) in PhMe (2 mL) at 6 °C for 6 h. The crude material was purified by column chromatography (hexane/^{i}PrOH 95:5). Product (*S*)-**10** was obtained as a white solid (80.0 mg, 0.17 mmol, 87%).

^{1}H NMR (600 MHz, DMSO-d_6) δ 8.68 (s, 1H), 7.66 (dd, J = 10.0, 3.4 Hz, 1H), 7.54 (dd, J = 7.7, 1.8 Hz, 2H), 7.44 (t, J = 7.6 Hz, 2H), 7.41 – 7.33 (m, 5H), 7.33 (dd, J = 8.5, 7.4 Hz, 2H), 7.27 – 7.23 (m, 2H), 7.21 (t, J = 7.4 Hz, 1H), 7.18 (t, J = 7.5 Hz, 1H), 7.09 (d, J = 8.0 Hz, 2H), 6.97 – 6.89 (m, 3H), 5.76 (dd, J = 22.4, 10.0 Hz, 1H); **^{13}C NMR** (151 MHz, DMSO-d_6) δ 154.1 (d, J = 10.4 Hz), 150.0 (d, J = 10.0 Hz), 149.8 (d, J = 9.8 Hz), 139.7, 135.2, 129.92, 129.90, 129.4, 128.8, 128.6 (d, J = 2.2 Hz), 128.2 (d, J = 2.7 Hz), 128.1 (d, J = 5.9 Hz), 125.4 (d, J = 6.3 Hz), 121.8, 120.4 (d, J = 3.8 Hz), 120.3 (d, J = 3.8 Hz), 117.8, 115.2, 50.6 (d, J = 157.2 Hz); **^{31}P NMR** (162 MHz, DMSO-d_6) δ 15.90; **HRMS** (ESI+, m/z): calculated for $C_{26}H_{24}N_2O_4P^+$ [M + H]$^+$: 459.1468, found: 459.1473.

Enantiomeric purity: 73:27 (crude, ^{31}P{^{1}H}-NMR in $CDCl_3$), 76:24 (after purification, ^{31}P {^{1}H}-NMR in $CDCl_3$).

$[\alpha]_D^{20}$ = 5.7 (c = 0.32, DMSO).

Isopropyl methyl ((*S*)-phenyl(3-phenylureido)methyl)phosphonate ((*S*)-11)

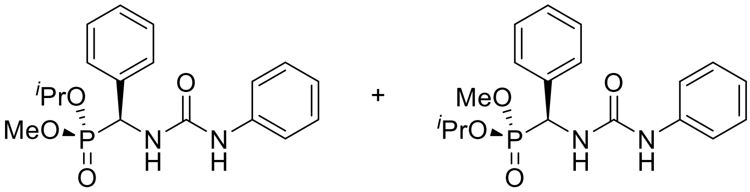

The general procedure C was followed using arylidene urea **6** (44.9 mg, 0.2 mmol), isopropyl methyl phosphite (32.2 mg, 0.233 mmol, 1.2 equiv.), (*S*)-TiPSY (8.7 mg, 0.01 mmol, 0.05 equiv.) in PhMe (2 mL) at 30 °C for 40 h. Purification by flash column chromatography using hexane/^{i}PrOH 95:5 afforded product (*S*)-**11** as a white semisolid (34.1 mg, 0.094 mmol, 47%).

NOTE: Characterization is provided for both indistinguishable diastereoisomers (1:1 mixture).

^{1}H-NMR (600 MHz, DMSO) δ 8.69 (s, 1H), 8.67 (s, 1H), 7.43 – 7.33 (m, 12H), 7.33 – 7.27 (m, 2H), 7.27 – 7.19 (m, 6H), 6.92 – 6.89 (m, 2H), 5.27 – 5.18 (m, 2H), 4.65 – 4.57 (m, 1H), 4.42 – 4.34 (m, 1H), 3.66 (d, *J* = 10.7 Hz, 3H), 3.48 (d, *J* = 10.7 Hz, 3H), 1.27 (d, *J* = 6.2 Hz, 3H), 1.21 – 1.17 (m, 6H), 0.93 (d, *J* = 6.2 Hz, 3H). **^{13}C-NMR** (151 MHz, DMSO) δ 154.14 (d, *J* = 3.5 Hz), 154.07 (d, *J* = 3.5 Hz), 139.87, 136.70, 136.64, 128.78, 128.28 (d, *J* = 2.1 Hz), 128.23 (d, *J* = 2.4 Hz), 127.87 (d, *J* = 5.6 Hz), 127.69 (d, *J* = 5.5 Hz), 127.61 (t, *J* = 3.4 Hz), 121.52, 117.55, 117.54, 71.41 (t, *J* = 6.6 Hz), 52.91 (d, *J* = 7.0 Hz), 52.75 (d, *J* = 7.0 Hz), 49.94 (dd, *J* = 154.6, 2.5 Hz), 23.89 (d, *J* = 3.2 Hz), 23.82 (d, *J* = 3.0 Hz), 23.36 (d, *J* = 5.2 Hz), 22.93 (d, *J* = 5.5 Hz). **^{31}P{^{1}H}-NMR** (243 MHz, DMSO-*d*$_6$) δ 22.76, 22.75. **HRMS (ESI)** calculated for $C_{18}H_{24}N_2O_4P$ [M$^+$ + 1]: 363.14682, found: 363.14644.

Enantiomeric purity: 67:33 *e.r.*, 1:1 *d.r.* (crude, ^{31}P{^{1}H}-NMR in $CDCl_3$), 67:33 *e.r.*, 1:1 *d.r.* (after purification, ^{31}P{^{1}H}-NMR in $CDCl_3$).

$[\alpha]_D^{20}$ = –1.9 (1.051, DMSO).

Diethyl (*S*)-((4-chlorophenyl)(3-phenylureido)methyl)phosphonate ((*S*)-12)

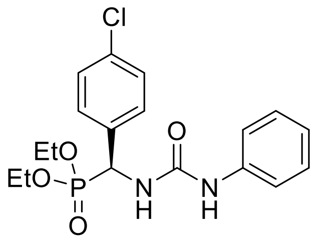

The general procedure C was followed using arylidene urea **S17** (51.7 mg, 0.20 mmol, 1.00 equiv.), diethyl phosphite (30.0 µL, 0.24 mmol, 1.20 equiv.) and (*S*)-TiPSY catalyst (8.7 mg, 0.01 mmol, 5 mol%) in PhMe (2 mL) at 30 °C for 48 h. The crude material was purified by column chromatography (hexane/^{i}PrOH 92:8). Product (*S*)-**12** was obtained as a white solid (59.0 mg, 0.15 mmol, 74%).

^{1}H NMR (600 MHz, DMSO-*d*$_6$) δ 8.69 (s, 1H), 7.45 (d, *J* = 8.6 Hz, 2H), 7.42 (dd, *J* = 8.6, 2.0 Hz, 2H), 7.36 (d, *J* = 7.6 Hz, 2H), 7.26 (dd, *J* = 9.5, 4.7 Hz, 1H), 7.22 (dd, *J* = 8.6, 7.3 Hz, 2H), 6.91 (tt, *J* = 7.3, 1.2 Hz, 1H), 5.26 (dd, *J* = 22.1, 9.5 Hz, 1H), 4.11 – 3.98 (m, 2H), 3.98 – 3.88 (m, 1H), 3.87 – 3.78 (m, 1H), 1.21 (t, *J* = 7.0 Hz, 3H), 1.09 (t, *J* = 7.0 Hz, 3H); **^{13}C NMR** (151 MHz, DMSO-*d*$_6$) δ 154.1 (d, *J* = 10.8 Hz), 139.8, 135.9, 132.3 (d, *J* = 3.3 Hz), 129.5 (d, *J* = 5.4 Hz), 128.8, 128.3 (d, *J* = 2.1 Hz), 121.6, 117.6, 62.7 (d, *J* = 7.0 Hz), 62.6 (d, *J* = 6.8 Hz), 49.5 (d, *J* = 153.5 Hz), 16.3 (d, *J* = 5.2 Hz), 16.1 (d, *J* = 5.4 Hz); **^{31}P{^{1}H}-NMR** (162 MHz, DMSO-*d*$_6$) δ 21.90; **HRMS** (ESI+, *m*/*z*): calculated for $C_{18}H_{21}ClN_2O_4PNa^+$ [M + Na]$^+$: 419.0898, found: 419.0899.

Enantiomeric purity: 91:9 *e.r.* (crude, ^{31}P{^{1}H}-NMR in $CDCl_3$), 89:11 *e.r.* (after purification, ^{31}P{^{1}H}-NMR in $CDCl_3$).

$[\alpha]_D^{20}$ = –25.7 (c = 0.63, MeOH).

Dimethyl (*S*)-((4-chlorophenyl)(3-phenylureido)methyl)phosphonate ((*S*)-13)

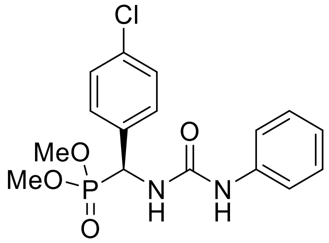

The general procedure C was followed using arylidene urea **S17** (51.7 mg, 0.20 mmol, 1.00 equiv.), dimethyl phosphite (21.0 µL, 0.24 mmol, 1.20 equiv.) and (*S*)-TiPSY catalyst (8.7 mg, 0.01 mmol, 5 mol%) in PhMe (2 mL) at 30 °C for 17 h. The crude material was purified by column chromatography (hexane/^{i}PrOH 92:8). Product (*S*)**-13** was obtained as a white solid (55.0 mg, 0.15 mmol, 75%).

^{1}H NMR (600 MHz, DMSO-d_6) δ 8.66 (s, 1H), 7.46 (d, *J* = 8.6 Hz, 2H), 7.43 (dd, *J* = 8.6, 2.0 Hz, 2H), 7.37 – 7.36 (dd, *J* = 8.6, 1.3 Hz, 2H), 7.32 (dd, *J* = 9.6, 4.6 Hz, 1H), 7.23 (dd, *J* = 8.6, 7.3 Hz, 2H), 6.92 (tt, *J* = 7.3, 1.2 Hz, 1H), 5.34 (dd, *J* = 22.0, 9.5 Hz, 1H), 3.70 (d, *J* = 10.6 Hz, 3H), 3.54 (d, *J* = 10.6 Hz, 3H); **^{13}C NMR** (151 MHz, DMSO-d_6) δ 154.1 (d, *J* = 10.8 Hz), 139.8, 135.7, 132.4 (d, *J* = 3.3 Hz), 129.5 (d, *J* = 5.4 Hz), 128.8, 128.4 (d, *J* = 2.1 Hz), 121.6, 117.6, 53.5 (d, *J* = 7.0 Hz), 53.3 (d, *J* = 6.7 Hz), 48.9 (d, *J* = 153.1 Hz; **^{31}P{^{1}H}-NMR** (162 MHz, DMSO-d_6) δ 24.20; **HRMS** (ESI+, *m*/*z*): calculated for $C_{16}H_{18}ClN_2O_4PNa^+$ [M + Na]$^+$: 391.0585, found: 391.0585.

Enantiomeric purity: 94:6 *e.r.* (crude, ^{31}P{^{1}H}-NMR in $CDCl_3$), 95:5 *e.r.* (after purification, ^{31}P{^{1}H}-NMR in $CDCl_3$).

$[\alpha]_D^{20}$ = –16.2 (c = 0.32, DMSO).

Dimethyl (*R*)-((4-chlorophenyl)(3-phenylureido)methyl)phosphonate ((*R*)-13)

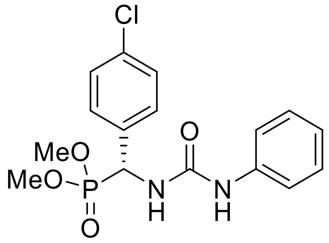

The general procedure C was followed using arylidene urea **S17** (51.7 mg, 0.20 mmol, 1.00 equiv.), dimethyl phosphite (21.0 µL, 0.24 mmol, 1.20 equiv.) and (*R*)-TiPSY catalyst (8.7 mg, 0.01 mmol, 5 mol%) in PhMe (2 mL) at 30 °C for 18 h. The crude material was purified by column chromatography (hexane/^{i}PrOH 92:8). Product (*R*)**-13** was obtained as a white solid (61.0 mg, 0.17 mmol, 83%). NMR and HRMS analysis is in agreement with data obtained for product (*S*)**-13**.

Enantiomeric purity: 92:8 *e.r.* (crude, ^{31}P{^{1}H}-NMR in $CDCl_3$), 94:6 *e.r.* (after purification, ^{31}P{^{1}H}-NMR in $CDCl_3$).

$[\alpha]_D^{20}$ = 17.9 (c = 0.38, DMSO).

(*S*)-1-((4-chlorophenyl)(5,5-dimethyl-2-oxido-1,3,2-dioxaphosphinan-2-yl)methyl)-3-phenylurea ((*S*)-14)

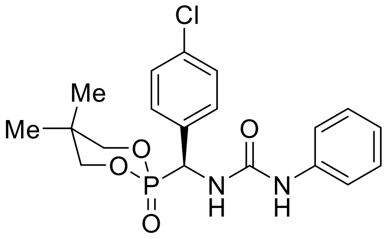

The general procedure C was followed using arylidene urea **S17** (51.7 mg, 0.20 mmol, 1.00 equiv.), neopentylene phosphite (36.0 mg, 0.24 mmol, 1.20 equiv.) and (*S*)-TiPSY catalyst (8.7 mg, 0.01 mmol, 5 mol%) in PhMe (2 mL) at 6 °C for 6 h. The crude material was purified by column chromatography (hexane/^{i}PrOH 96:4). Product (*S*)**-14** was obtained as a white solid (78.0 mg, 0.19 mmol, 95%).

^{1}H NMR (600 MHz, DMSO-d_6) δ 8.66 (s, 1H), 7.52 (dd, *J* = 8.6, 1.9 Hz, 2H), 7.46 (d, *J* = 8.5 Hz, 2H), 7.36 (d, *J* = 7.4 Hz, 2H), 7.32 (dd, *J* = 9.6, 4.0 Hz, 1H), 7.23 (dd, *J* = 8.6, 7.3 Hz, 2H), 6.92 (tt, *J* = 7.3, 1.2 Hz, 1H), 5.75 (dd, *J* = 17.6, 9.6 Hz, 1H), 4.49 (dd, *J* = 11.0, 2.6 Hz, 1H), 4.38 (dd, *J* = 11.2, 2.9 Hz, 1H), 3.96 (ddd, *J* = 19.7, 11.0, 2.6 Hz, 1H), 3.86 (ddd, *J* = 19.2, 10.9, 2.7 Hz, 1H), 1.13 (s, 3H), 0.88 (s, 3H); **^{13}C NMR** (151 MHz, DMSO-d_6) δ 154.1 (d, *J* = 9.4 Hz), 139.8, 135.7, 132.4 (d, *J* = 3.0 Hz), 129.6 (d, *J* = 5.4 Hz), 128.8, 128.4 (d, *J* = 1.0 Hz), 121.6, 117.6, 76.5 (dd, *J* = 5.9, 3.8 Hz), 45.9 (d, *J* = 142.8 Hz), 40.1, 32.0 (d, *J* = 1.1 Hz), 21.3, 19.5; **^{31}P{^{1}H}-NMR** (162 MHz, DMSO-d_6) δ 15.05; **HRMS** (ESI+, *m*/*z*): calculated for $C_{19}H_{23}ClN_2O_4P^+$ [M + H]$^+$: 409.1079, found: 409.1082.

Enantiomeric purity: 86:14 *e.r.* (crude, ^{31}P{^{1}H}-NMR in $CDCl_3$), 86.5:13.5 *e.r.* (after purification, ^{31}P{^{1}H}-NMR in $CDCl_3$).

$[\alpha]_D^{20}$ = 12.0 (c = 0.44, DMSO).

(*R*)-1-((4-chlorophenyl)(5,5-dimethyl-2-oxido-1,3,2-dioxaphosphinan-2-yl)methyl)-3-phenylurea ((*R*)-14)

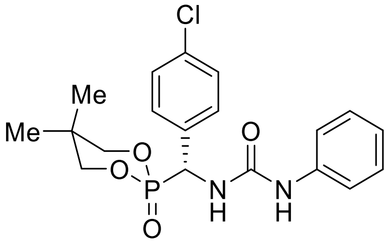

The general procedure C was followed using arylidene urea **S17** (51.7 mg, 0.20 mmol, 1.00 equiv.), neopentylene phosphite (36.0 mg, 0.24 mmol, 1.20 equiv.) and (*R*)-TiPSY catalyst (8.7 mg, 0.01 mmol, 5 mol%) in PhMe (2 mL) at 6 °C for 6 h. The crude material was purified by column chromatography (hexane/^{i}PrOH 96:4). Product (*R*)**-14** was obtained as a white solid (71.0 mg, 0.17 mmol, 87%). NMR and HRMS analysis is in agreement with data obtained for product (*S*)**-14**.

Enantiomeric purity: 85:15 *e.r.* (crude, ^{31}P{^{1}H}-NMR in $CDCl_3$), 88:12 *e.r.* (after purification, ^{31}P{^{1}H}-NMR in $CDCl_3$).

$[\alpha]_D^{20}$ = –14.8 (c = 0.55, DMSO).

Dimethyl (*S*)-((4-fluorophenyl)(3-phenylureido)methyl)phosphonate ((*S*)-15)

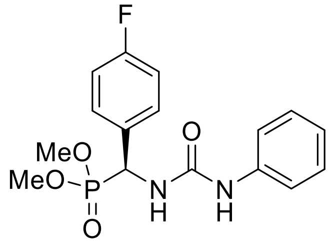

The general procedure C was followed using arylidene urea **S18** (48.5 mg, 0.20 mmol, 1.00 equiv.), dimethyl phosphite (21.0 µL, 0.24 mmol, 1.20 equiv.) and (*S*)-TiPSY catalyst (8.7 mg, 0.01 mmol, 5 mol%) in PhMe (2 mL) at 30 °C for 48 h. The crude material was purified by column chromatography (hexane/^{i}PrOH 95:5). Product (*S*)**-15** was obtained as a white solid (56.0 mg, 0.16 mmol, 80%).

^{1}H NMR (600 MHz, DMSO-d_6) δ 8.64 (s, 1H), 7.47 – 7.41 (m, 2H), 7.36 (d, *J* = 7.3 Hz, 2H), 7.30 (dd, *J* = 9.6, 4.4 Hz, 1H), 7.23 (d, *J* = 7.7 Hz, 2H), 7.22 (d, *J* = 7.7 Hz, 2H), 6.92 (tt, *J* = 7.3, 1.2 Hz, 1H), 5.33 (dd, *J* = 21.7, 9.6 Hz, 1H), 3.70 (d, *J* = 10.6 Hz, 3H), 3.52 (d, *J* = 10.6 Hz, 3H); **^{13}C NMR** (151 MHz, DMSO-d_6) δ 161.4 (dd, *J* = 243.8, 2.8 Hz), 154.0 (d, *J* = 10.5 Hz), 139.8, 132.8 (d, *J* = 2.8 Hz), 129.72 (d, *J* = 8.4 Hz), 129.67 (d, *J* = 8.4 Hz), 128.8, 121.6, 117.6, 115.3 (dd, *J* = 21.7, 2.0 Hz), 53.5 (d, *J* = 7.0 Hz), 53.3 (d, *J* = 6.6 Hz), 48.7 (d, *J* = 154.1 Hz); **^{19}F NMR** (376 MHz, DMSO-d_6) δ -114.68 – -114.85 (m); **^{31}P{^{1}H}-NMR** (162 MHz, DMSO-d_6) δ 24.51 (d, *J* = 4.5 Hz); **HRMS** (ESI+, *m*/*z*): calculated for $C_{16}H_{18}FN_2O_4PNa^+$ [M + Na]$^+$: 375.0880, found: 375.0881.

Enantiomeric purity: 93.3:6.7 *e.r.* (crude, ^{31}P{^{1}H}-NMR in $CDCl_3$), 93.6:6.4 *e.r.* (after purification, ^{31}P{^{1}H}-NMR in $CDCl_3$).

$[\alpha]_D^{20}$ = –9.2 (c = 0.30, DMSO).

Dimethyl (*S*)-((3-phenylureido)(p-tolyl)methyl)phosphonate ((*S*)-16)

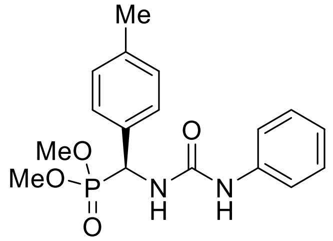

The general procedure C was followed using arylidene urea **S19** (47.7 mg, 0.20 mmol, 1.00 equiv.), dimethyl phosphite (21.0 µL, 0.24 mmol, 1.20 equiv.) and (*S*)-TiPSY catalyst (8.7 mg, 0.01 mmol, 5 mol%) in PhMe (2 mL) at 30 °C for 18 h. The crude material was purified by column chromatography (hexane/^{i}PrOH 93:7). Product (*S*)**-16** was obtained as a white solid (63.0 mg, 0.18 mmol, 90%).

^{1}H NMR (600 MHz, DMSO-d_6) δ 8.64 (s, 1H), 7.38 – 7.32 (m, 2H), 7.28 (dd, *J* = 8.2, 2.0 Hz, 2H), 7.25 – 7.20 (m, 3H), 7.19 (d, *J* = 7.9 Hz, 2H), 6.91 (tt, *J* = 7.3, 1.2 Hz, 1H), 5.25 (dd, *J* = 21.5, 9.7 Hz, 1H), 3.68 (d, *J* = 10.6 Hz, 3H), 3.50 (d, *J* = 10.5 Hz, 3H), 2.29 (s, 1H); **^{13}C NMR** (151 MHz, DMSO-d_6) δ 154.1 (d, *J* = 10.4 Hz), 139.9, 137.0 (d, *J* = 2.8 Hz), 133.41, 129.0 (d, *J* = 2.1 Hz), 128.8, 127.6 (d, *J* = 5.5 Hz), 121.5, 117.6, 53.4 (d, *J* = 6.9 Hz), 53.2 (d, *J* = 6.8 Hz), 49.1 (d, *J* = 154.1 Hz), 20.7; **^{31}P{^{1}H}-NMR** (162 MHz, DMSO-d_6) δ 24.86 (d, *J* = 4.5 Hz); **HRMS** (ESI+, *m*/*z*): calculated for $C_{17}H_{21}N_2O_4PNa^+$ [M + Na]$^+$: 371.1131, found: 371.1129.

Enantiomeric purity: 96:4 *e.r.* (crude, $^{31}P\{^1H\}$-NMR in $CDCl_3$), 96.5:3.5 *e.r.* (after purification, $^{31}P\{^1H\}$-NMR in $CDCl_3$).

$[\alpha]_D^{20}$ = –21.2 (c = 0.29, DMSO).

Dimethyl (*S*)-((4-methoxyphenyl)(3-phenylureido)methyl)phosphonate ((*S*)-17)

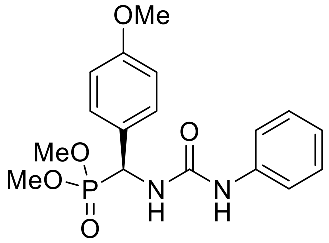

The general procedure C was followed using arylidene urea **S20** (50.9 mg, 0.20 mmol, 1.00 equiv.), dimethyl phosphite (21.0 µL, 0.24 mmol, 1.20 equiv.) and (*S*)-TiPSY catalyst (8.7 mg, 0.01 mmol, 5 mol%) in PhMe (2 mL) at 30 °C for 18 h. The crude material was purified by column chromatography (hexane/^{i}PrOH 93:7). Product (*S*)**-17** was obtained as a white solid (67.0 mg, 0.18 mmol, 92%).

^{1}H NMR (600 MHz, DMSO-*d*$_6$) δ 8.62 (s, 1H), 7.35 (dd, *J* = 8.7, 1.2 Hz, 2H), 7.32 (dd, *J* = 8.8, 2.0 Hz, 2H), 7.25 – 7.17 (m, 3H), 6.94 (d, *J* = 8.6 Hz, 2H), 6.91 (tt, *J* = 7.3, 1.2 Hz, 1H), 5.24 (dd, *J* = 21.3, 9.7 Hz, 1H), 3.74 (s, 3H), 3.68 (d, *J* = 10.5 Hz, 3H), 3.49 (d, *J* = 10.5 Hz, 3H); **^{13}C NMR** (151 MHz, DMSO-*d*$_6$) δ 158.8 (d, *J* = 2.6 Hz), 154.0 (d, *J* = 10.6 Hz), 139.9, 128.9 (d, *J* = 5.7 Hz), 128.8, 128.3, 121.5, 117.6, 113.8 (d, *J* = 1.8 Hz), 55.1, 53.3 (d, *J* = 7.0 Hz), 53.2 (d, *J* = 6.9 Hz), 48.7 (d, *J* = 155.1 Hz); **^{31}P{^{1}H}-NMR** (162 MHz, DMSO-*d*$_6$) δ 24.99; **HRMS** (ESI+, *m*/*z*): calculated for $C_{17}H_{21}N_2O_5PNa^+$ $[M + Na]^+$: 387.1080, found: 387.1081.

Enantiomeric purity: 95:5 *e.r.* (crude, $^{31}P\{^1H\}$-NMR in $CDCl_3$), 94:6 *e.r.* (after purification, $^{31}P\{^1H\}$-NMR in $CDCl_3$).

$[\alpha]_D^{20}$ = –8.1 (c = 0.45, DMSO).

Dimethyl (*S*)-((2-hydroxyphenyl)(3-phenylureido)methyl)phosphonate ((*S*)-18)

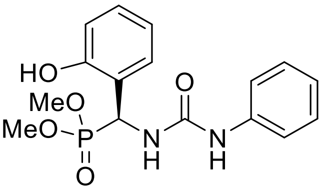

The general procedure C was followed using arylidene urea **S21** (48.1 mg, 0.20 mmol, 1.00 equiv.), dimethyl phosphite (21.0 µL, 0.24 mmol, 1.20 equiv.) and (*S*)-TiPSY catalyst (8.7 mg, 0.01 mmol, 5 mol%) in PhMe (2 mL) at 30 °C for 18 h. The crude material was purified by column chromatography (DCM/MeOH 97:3). Product (*S*)**-18** was obtained as a white solid (43.0 mg, 0.12 mmol, 61%).

NOTE: Product (*S*)**-18** was obtained with 85% NMR-purity.

^{1}H NMR (400 MHz, MeOD-*d*$_4$) δ δ 7.65 – 7.57 (m, 1H), 7.37 – 7.27 (m, 4H), 7.25 – 7.20 (m, 2H), 7.15 (tt, *J* = 7.7, 1.8 Hz, 1H), 6.96 (tt, *J* = 7.3, 1.2 Hz, 1H), 6.89 – 6.80 (m, 2H), 5.89 (d, *J* = 21.3 Hz, 1H), 3.79 (d, *J* = 10.6 Hz, 3H), 3.61 (d, *J* = 10.6 Hz, 3H); **^{13}C NMR** (151 MHz, DMSO-*d*$_6$) δ 155.1 (d, *J* = 6.0 Hz), 154.4 (d, *J* = 9.1 Hz), 140.5, 136.8, 129.23, 129.17, 129.1, 123.3, 121.9, 119.5, 118.2, 118.0, 115.72, 53.6 (d, *J* = 6.7 Hz), 53.5 (d, *J* = 6.6 Hz), 43.6 (d, *J* = 156.0

Hz); **^{31}P NMR** (162 MHz, DMSO-*d*$_6$) δ 25.52; **HRMS** (ESI+, *m*/*z*): calculated for $C_{16}H_{20}N_2O_5P^+$ $[M + H]^+$: 351.1104, found: 351.1001.

Enantiomeric purity: 81:19 *e.r.* (crude, SFC), 79.5:20.5 *e.r.* (after purification, ^{31}P NMR in $(CD_3)_2CO$); 79:21 *e.r.* (**SFC** Trefoil AMY1, CO_2:MeOH 97:3 → 50:50% in 4.5 min, 6 min, 1.8 mL/min, λ_{abs} = 238 nm, $t_R(R)$ = 2.9 min, $t_R(S)$ = 3.1 min).

NOTE: *e.r.* of the crude mixture was not possible to be determined using NMR-analysis because of its poor solubility in deuterated solvents allowing dimer formation.

$[\alpha]_D^{20}$ = 13.3 (c = 0.61, DMSO).

Dimethyl (*S*)-(furan-2-yl(3-phenylureido)methyl)phosphonate ((*S*)-19)

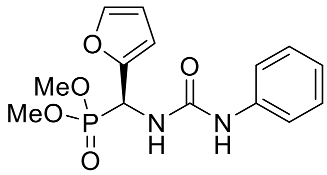

The general procedure C was followed using arylidene urea **S22** (42.8 mg, 0.20 mmol, 1.00 equiv.), dimethyl phosphite (21.0 µL, 0.24 mmol, 1.20 equiv.) and (*S*)-TiPSY catalyst (8.7 mg, 0.01 mmol, 5 mol%) in PhMe (2 mL) at 30 °C for 78 h. The crude material was purified by column chromatography (pentane /EtOAc 70:30). Product (*S*)**-19** was obtained as a light brown solid (43.0 mg, 0.13 mmol, 66%).

^{1}H NMR (600 MHz, DMSO-*d*$_6$) 8.65 (s, 1H), 7.68 (s, 1H), 7.36 (d, *J* = 8.0 Hz, 2H), 7.24 (t, *J* = 7.9 Hz, 2H), 7.06 (dd, *J* = 9.8, 2.4 Hz, 1H), 6.93 (t, *J* = 7.4 Hz, 1H), 6.49 – 6.42 (m, 2H), 5.43 (dd, *J* = 21.5, 9.7 Hz, 1H), 3.71 (d, *J* = 10.7 Hz, 3H), 3.60 (d, *J* = 10.6 Hz, 3H); **^{13}C NMR** (151 MHz, DMSO-*d*$_6$) δ 153.9 (d, *J* = 8.2 Hz), 149.1, 143.1 (d, *J* = 2.5 Hz), 139.7, 136.5, 128.8, 127.4, 121.7, 117.7, 110.8 (d, *J* = 1.9 Hz), 108.4 (d, *J* = 6.9 Hz), 53.4 (d, *J* = 6.8 Hz), 53.3 (d, *J* = 6.6 Hz), 43.7 (d, *J* = 160.7 Hz); **^{31}P NMR** (243 MHz, DMSO-*d*$_6$) δ 22.08; **HRMS** (ESI+, *m*/*z*): calculated for $C_{14}H_{18}N_2O_5P^+$ $[M + H]^+$: 325.0948, found: 325.0943.

Enantiomeric purity: 96:4 *e.r.* (crude, ^{31}P{^{1}H}-NMR in $CDCl_3$), 96:4 *e.r.* (after purification, ^{31}P{^{1}H}-NMR in $CDCl_3$).

$[\alpha]_D^{20}$ = –42.9 (c = 0.21, DMSO).

dimethyl (*S*)-((3-(4-chlorophenyl)ureido)(phenyl)methyl)phosphonate ((*S*)-20)

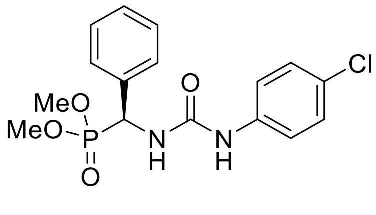

The general procedure C was followed using arylidene urea **S23** (51.7 mg, 0.2 mmol), dimethyl phosphite (21 µL, 0.233 mmol, 1.2 equiv.), (*S*)-TiPSY (8.7 mg, 0.01 mmol, 0.05 equiv.) in PhMe (2 mL) at 30 °C for 63 h. Purification by flash column chromatography using hexane/^{i}PrOH 90:10 as eluent. Product (*S*)**-20** was obtained as a white solid (49 mg, 0.133 mmol, 66%).

^{1}H-NMR (600 MHz, DMSO-*d*$_6$) δ 8.78 (s, 1H), 7.43 – 7.37 (m, 6H), 7.35 – 7.29 (m, 2H), 7.27 (d, J = 8.9 Hz, 2H), 5.29 (dd, J = 21.9, 9.6 Hz, 1H), 3.69 (d, J = 10.6 Hz, 3H), 3.49 (d, J = 10.6 Hz, 3H). **^{13}C-NMR** (151 MHz, DMSO-*d*$_6$) δ 153.9 (d, J = 10.4 Hz), 138.8, 136.3, 128.6, 128.4

(d, J = 1.4 Hz), 127.71, 127.70 (d, J = 5.2 Hz), 125.0, 119.1, 53.4 (d, J = 7.1 Hz), 53.2 (d, J = 6.7 Hz), 49.4 (d, J = 153.3 Hz). **^{31}P{^{1}H}-NMR** (162 MHz, DMSO-*d*$_6$) δ 24.59. **HRMS (ESI)** calculated for: $C_{16}H_{18}ClN_2O_4PNa$: 391.05849, found: 391.05772.

Enantiomeric purity: 86:14 *e.r.* (crude, by ^{31}P{^{1}H}-NMR in $CDCl_3$), 86:14 *e.r.* (by ^{31}P{^{1}H}-NMR in $CDCl_3$).

$[\alpha]_D^{20}$ = –14.2 (0.69, DMSO)

Dimethyl (*S*)-((3-(4-fluorophenyl)ureido)(phenyl)methyl)phosphonate ((*S*)-21)

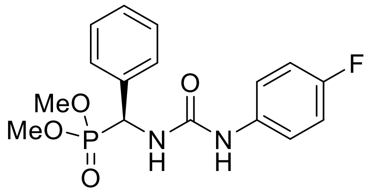

The general procedure C was followed using arylidene urea **S24** (48.5 mg, 0.2 mmol), dimethyl phosphite (21 µL, 0.233 mmol, 1.2 equiv.), (*S*)-TiPSY (8.7 mg, 0.01 mmol, 0.05 equiv.) in PhMe (2 mL) at 30 °C for 20 h. Purification by flash column chromatography using hexane/^{i}PrOH 95:5 as eluent. Product (*S*)-**21** was obtained as a white solid (47.5 mg, 0.135 mmol, 67%).

^{1}H-NMR (600 MHz, DMSO-*d*$_6$) δ 8.69 (s, 1H), 7.42 – 7.34 (m, 6H), 7.33 – 7.26 (m, 2H), 7.06 (apparent t, J = 8.9 Hz, 2H), 5.30 (dd, J = 21.6, 9.3 Hz, 1H), 3.69 (d, J = 10.6 Hz, 3H), 3.50 (d, J = 10.5 Hz, 3H). **^{13}C-NMR** (151 MHz, DMSO-*d*$_6$) δ 157.9 (d, J = 1.6 Hz), 156.3, 154.1 (d, J = 10.7 Hz), 136.4, 136.2 (d, J = 2.5 Hz), 128.4 (d, J = 1.7 Hz), 127.7 (d, J = 5.2 Hz), 119.2 (d, J = 7.6 Hz), 115.3 (d, J = 22.3 Hz), 53.4 (d, J = 7.0 Hz), 53.2 (d, J = 6.9 Hz), 49.4 (d, J = 153.3 Hz). **^{19}F NMR** (565 MHz, DMSO) δ -121.87 (tt, J = 9.4, 4.6 Hz). **^{31}P{^{1}H}-NMR** (162 MHz, DMSO-*d*$_6$) δ 24.69. **HRMS (ESI)** calculated for: $C_{16}H_{18}FN_2O_4PNa$: 375.08804, found: 375.08665.

Enantiomeric purity: 85:15 *e.r.* (crude, by ^{31}P{^{1}H}-NMR in $CDCl_3$), 91:9 *e.r.* (by ^{31}P{^{1}H}-NMR in $CDCl_3$).

$[\alpha]_D^{20}$ = –6.7 (1.04, DMSO)

Dimethyl (*S*)-((3-(3,5-bis(trifluoromethyl)phenyl)ureido)(phenyl)methyl)phosphonate ((*S*)-22)

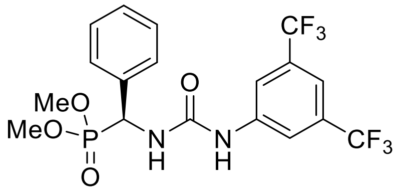

The general procedure C was followed using arylidene urea **S25** (72.1 mg, 0.2 mmol), dimethyl phosphite (21 µL, 0.233 mmol, 1.2 equiv.), (*S*)-TiPSY (8.7 mg, 0.01 mmol, 0.05 equiv.) in PhMe (2 mL) at 30 °C for 32 h. Purification by flash column chromatography using hexane/^{i}PrOH 90:10 as eluent. Product (*S*)-**22** was obtained as a white solid (51 mg, 0.108 mmol, 54%).

^{1}H-NMR (600 MHz, DMSO-*d*$_6$) δ 9.33 (s, 1H), 8.02 (s, 2H), 7.66 (dd, J = 9.5, 4.2 Hz, 1H), 7.58 (s, 1H), 7.45 – 7.41 (m, 2H), 7.39 (dd, J = 8.5, 6.8 Hz, 2H), 7.35 – 7.29 (m, 1H), 5.32 (dd, J = 21.5, 9.3 Hz, 1H), 3.70 (d, J = 10.6 Hz, 3H), 3.50 (d, J = 10.6 Hz, 3H). **^{13}C-NMR** (151 MHz, DMSO-*d*$_6$) δ 153.9 (d, J = 10.9 Hz), 141.8, 136.0, 130.7 (q, J = 32.6 Hz), 128.4, 127.8, 127.8 (d, J = 5.6 Hz), 123.3 (q, J = 272.7 Hz), 117.4, 114.2, 53.4 (d, J = 7.0 Hz), 53.3 (d, J = 6.8 Hz), 49.6

(d, J = 153.6 Hz). **^{19}F NMR** (565 MHz, DMSO-*d*$_6$) δ -61.74. **^{31}P{^{1}H}-NMR** (162 MHz, DMSO-*d*$_6$) δ 24.24. **HRMS (ESI)** calculated for: $C_{18}H_{17}F_6N_2O_4PNa$: 493.07223, found: 493.07068.

Enantiomeric purity: 85:15 *e.r.* (crude, by ^{31}P{^{1}H}-NMR in $CDCl_3$), 85.5:14.5 *e.r.* (by ^{31}P{^{1}H}-NMR in $CDCl_3$).

$[\boldsymbol{\alpha}]_{\boldsymbol{D}}^{\mathbf{20}}$ = –14.2 (0.687, DMSO)

Dimethyl (*S*)-(phenyl(3-(p-tolyl)ureido)methyl)phosphonate ((*S*)-23)

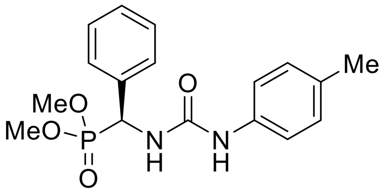

The general procedure C was followed using arylidene urea **S26** (47.7 mg, 0.2 mmol), dimethyl phosphite (21 µL, 0.233 mmol, 1.2 equiv.), (*S*)-TiPSY (8.7 mg, 0.01 mmol, 0.05 equiv.) in PhMe (2 mL) at 30 °C for 48 h. Purification by flash column chromatography using hexane/^{i}PrOH 90:10 as eluent. Product (*S*)**-23** was obtained as a pale yellow solid (47.6 mg, 0.137 mmol, 68%).

^{1}H-NMR (600 MHz, DMSO-*d*$_6$) δ 8.55 (s, 1H), 7.42 – 7.35 (m, 4H), 7.34 – 7.28 (m, 1H), 7.27 – 7.22 (m, 3H), 7.03 (d, J = 8.1 Hz, 2H), 5.29 (dd, J = 22.0, 9.6 Hz, 1H), 3.68 (d, J = 10.6 Hz, 3H), 3.50 (d, J = 10.6 Hz, 3H), 2.20 (s, 3H). **^{13}C-NMR** (101 MHz, DMSO-*d*$_6$) δ 154.1 (d, J = 10.4 Hz), 137.3, 136.5, 130.3, 129.1, 128.4 (d, J = 2.1 Hz), 127.7, 127.7, 117.6, 53.4 (d, J = 6.9 Hz), 53.2 (d, J = 6.9 Hz), 49.4 (d, J = 153.3 Hz), 20.3. **^{31}P{^{1}H}-NMR** (162 MHz, DMSO-*d*$_6$) δ 24.79. **HRMS (ESI)** calculated for: $C_{17}H_{21}N_2O_4PNa$: 371.11311, found: 371.11236.

Enantiomeric purity: 92:8 *e.r.* (crude, by ^{31}P{^{1}H}-NMR in $CDCl_3$), 95:6 *e.r.* (after purification, by ^{31}P{^{1}H}-NMR in $CDCl_3$).

$[\boldsymbol{\alpha}]_{\boldsymbol{D}}^{\mathbf{20}}$ = –12.4 (1.206, DMSO)

Diethyl (*S*)-((3-(4-methoxyphenyl)ureido)(phenyl)methyl)phosphonate ((*S*)-24)

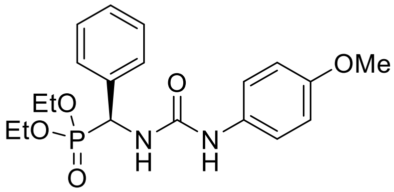

The general procedure C was followed using arylidene urea **S27** (50.9 mg, 0.2 mmol), diethyl phosphite (30 µL, 0.233 mmol, 1.2 equiv.), (*S*)-TiPSY (8.7 mg, 0.01 mmol, 0.05 equiv.) in PhMe (2 mL) at 30 °C for 96 h. Purification by flash column chromatography using hexane/^{i}PrOH 9:1 as eluent. Product (*S*)**-24** was obtained as a white to tan solid (52.5 mg, 0.134 mmol, 67%).

^{1}H-NMR (400 MHz, DMSO-*d*$_6$) δ 8.51 (s, 1H), 7.44 – 7.33 (m, 4H), 7.32 – 7.24 (m, 3H), 7.14 (dd, J = 9.9, 4.3 Hz, 1H), 6.81 (d, J = 9.0 Hz, 2H), 5.23 (dd, J = 12.2, 9.7 Hz, 1H), 4.11 – 3.98 (m, 2H), 3.97 – 3.82 (m, 1H), 3.82 – 3.72 (m, 1H), 3.68 (s, 3H), 1.21 (t, J = 7.1 Hz, 3H), 1.06 (t, J = 7.0 Hz, 3H). **^{13}C-NMR** (101 MHz, DMSO-*d*$_6$) δ 154.3 (d, J = 10.3 Hz), 154.2, 136.8, 133.0, 128.2 (d, J = 2.1 Hz), 127.7 (d, J = 5.5 Hz), 127.6 (d, J = 2.8 Hz), 119.2, 114.0, 62.5 (d, J = 7.0 Hz), 62.4 (d, J = 7.0 Hz), 55.1, 50.0 (d, J = 153.3 Hz), 16.2 (d, J = 5.3 Hz), 16.0 (d, J = 5.5 Hz). **^{31}P{^{1}H}-NMR** (162 MHz, DMSO-*d*$_6$) δ 22.55. **HRMS (ESI)** calculated for: $C_{19}H_{25}N_2O_5PNa$: 415.13933, found: 415.13832.

Enantiomeric purity: 90:10 *e.r.* (crude, by $^{31}P\{^1H\}$-NMR in $CDCl_3$) 92:8 *e.r.* (after purification, by $^{31}P\{^1H\}$-NMR in $CDCl_3$), 92.5:7.5 *e.r.* (HPLC Chiralpak AY-H Lux 5µm Amylose-2, *n*-heptane/^{i}PrOH 90:10, 40 °C, 1 mL/min, λ_{abs} = 244 nm t_R(major) = 18.0 min, t_R(minor) = 28.9 min).

$[\alpha]_D^{20}$ = –7.6 (4.136, DMSO)

Dimethyl (*S*)-((3-(4-methoxyphenyl)ureido)(phenyl)methyl)phosphonate ((*S*)-25)

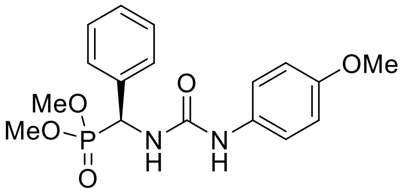

The general procedure C was followed using arylidene urea **S27** (50.9 mg, 0.2 mmol), dimethyl phosphite (21 µL, 0.233 mmol, 1.2 equiv.), (*S*)-TiPSY (8.7 mg, 0.01 mmol, 0.05 equiv.) in PhMe (2 mL) at 30 °C for 48 h. Purification by flash column chromatography using hexane/^{i}PrOH 85:15 as eluent. Product (*S*)**-25** was obtained as a white to tan solid (50.3 mg, 0.138 mmol, 69%).

^{1}H-NMR (400 MHz, DMSO-*d*$_6$) δ 8.46 (s, 1H), 7.41 – 7.32 (m, 5H), 7.26 (d, J = 9.0 Hz, 2H), 7.18 (dd, J = 9.8, 4.2 Hz, 1H), 6.81 (d, J = 9.0 Hz, 2H), 5.35 – 5.20 (m, 1H), 3.68 (t, J = 5.3 Hz, 6H), 3.50 (d, J = 10.6 Hz, 3H). **^{13}C-NMR** (101 MHz, DMSO-*d*$_6$) δ 154.7 (d, J = 10.5 Hz), 154.6, 137.0, 136.9, 133.4, 128.8 (d, J = 2.1 Hz), 128.1 (d, J = 2.9 Hz), 119.7, 114.4, 55.6, 53.8 (d, J = 7.0 Hz), 53.6 (d, J = 6.8 Hz), 49.9 (d, J = 153.2 Hz). **^{31}P{^{1}H}-NMR** (162 MHz, DMSO-*d*$_6$) δ 24.80. **HRMS (ESI)** calculated for: $C_{17}H_{21}N_2O_5PNa$: 387.10803, found: 387.10702.

Enantiomeric purity: 92:8 *e.r.* (crude, by $^{31}P\{^1H\}$-NMR in $CDCl_3$), 92:8 *e.r.* (after purification, by $^{31}P\{^1H\}$-NMR in $CDCl_3$).

$[\alpha]_D^{20}$ = 6.3 (0.572, DMSO)

(*R*)-1-((5,5-dimethyl-2-oxido-1,3,2-dioxaphosphinan-2-yl)(phenyl)methyl)-3-(4-methoxyphenyl)urea ((*R*)-26)

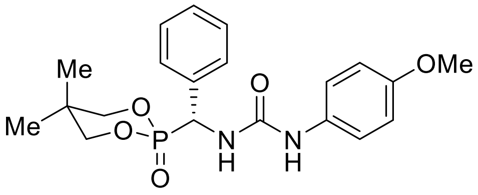

The general procedure C was followed using arylidene urea **S27** (50.9 mg, 0.2 mmol), neopentylene phosphite (36.4 mg, 0.233 mmol, 1.2 equiv.), (*R*)-TiPSY (8.7 mg, 0.01 mmol, 0.05 equiv.) in PhMe (2 mL) at 6 °C for 24 h. Purification by flash column chromatography using hexane/^{i}PrOH 85:15 as eluent. Product (*R*)**-26** was obtained as a white solid (57.7 mg, 0.143 mmol, 71%).

^{1}H-NMR (400 MHz, DMSO-*d*$_6$) δ 8.48 (s, 1H), 7.52 – 7.44 (m, 2H), 7.42 – 7.28 (m, 4H), 7.27 (d, *J* = 9.0 Hz, 2H), 6.82 (d, *J* = 9.0 Hz, 2H), 5.68 (dd, *J* = 17.5, 9.7 Hz, 1H), 4.44 (dd, *J* = 11.0, 3.2 Hz, 1H), 4.36 (dd, *J* = 11.1, 3.4 Hz, 1H), 4.02 – 3.89 (m, 1H), 3.93 – 3.78 (m, 1H), 3.68 (s, 3H), 1.13 (s, 3H), 0.88 (s, 3H). **^{13}C-NMR** (101 MHz, DMSO-*d*$_6$) δ 154.8 (d, J = 8.8 Hz), 154.6, 137.0, 133.5, 128.8 (d, J = 1.9 Hz), 128.3 (d, J = 5.5 Hz), 128.0 (d, J = 2.6 Hz), 119.7, 114.4, 76.9 (d, J = 4.1 Hz), 76.9 (d, J = 4.0 Hz), 55.6, 46.9 (d, J = 143.3 Hz), 32.4 (d, J = 7.0 Hz), 21.7, 20.0. **^{31}P{^{1}H}-NMR** (162 MHz, DMSO-*d*$_6$) δ 15.55. **HRMS (ESI)** calculated for: $C_{20}H_{25}N_2O_5PNa$: 427.13933, found: 427.13808.

Enantiomeric purity: 82:18 *e.r.* (crude, by $^{31}P\{^{1}H\}$-NMR in $CDCl_3$), 87:13 *e.r.* (after purification, by $^{31}P\{^{1}H\}$-NMR in $CDCl_3$).

$[\alpha]_D^{20}$ = –22.6 (0.61, DMSO)

Dimethyl (*S*)-((3-mesitylureido)(phenyl)methyl)phosphonate ((*S*)-27)

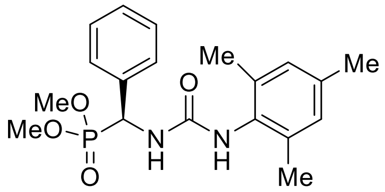

The general procedure C was followed using arylidene urea **S28** (44 mg, 0.165 mmol), dimethyl phosphite (18 µL, 0.192 mmol, 1.2 equiv.), (*S*)-TiPSY (7.1 mg, 0.01 mmol, 0.05 equiv.) in PhMe (1.7 mL) at 30 °C for 40 h. Purification by flash column chromatography using hexane/^{i}PrOH 90:10 as eluent. Product (*S*)**-27** was obtained as a white solid (34 mg, 0.09 mmol, 55%).

^{1}H-NMR (600 MHz, DMSO-*d*$_6$) δ 7.62 – 7.55 (m, 1H), 7.44 – 7.35 (m, 4H), 7.35 – 7.28 (m, 2H), 6.83 (s, 2H), 5.30 (dd, J = 21.5, 10.3 Hz, 1H), 3.70 (d, J = 10.6 Hz, 3H), 3.51 (d, J = 10.5 Hz, 3H), 2.19 (s, 3H), 2.07 (s, 6H). **^{13}C-NMR** (151 MHz, DMSO-*d*$_6$) δ 155.5 (d, J = 9.3 Hz), 137.1, 136.9, 135.5, 135.2, 133.4, 128.8 (d, J = 1.7 Hz), 128.7, 128.1 (d, J = 5.4 Hz), 53.9 (d, J = 6.8 Hz), 53.5 (d, J = 6.9 Hz), 50.2 (d, J = 152.2 Hz), 20.9, 18.5. **^{31}P{^{1}H}-NMR** (162 MHz, DMSO-*d*$_6$) δ 25.06. **HRMS (ESI)** calculated for: $C_{19}H_{26}N_2O_4P$ [M^+ + 1]: 377.16247, found: 377.16200.

Enantiomeric purity: 85:15 *e.r.* (by ^{1}H-NMR in CD_2Cl_2). NOTE: The presence of rotamers in solution made the determination of e.r. difficult by NMR (broad signals). However, the ^{1}H-NMR signal in CD_2Cl_2 (600 MHz) at 3.49 ppm, which corresponds to one of the methyl groups of the phosphonate moiety, was clearly resolved, which allowed the determination of the enantiomeric purity using the SIDA effect.

$[\alpha]_D^{20}$ = –6.1 (1.32, DMSO)

Dimethyl (*S*)-((3-(naphthalen-2-yl)ureido)(phenyl)methyl)phosphonate ((*S*)-28)

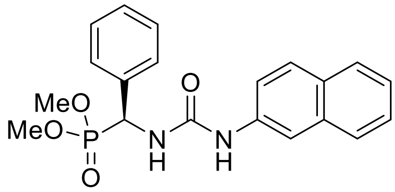

The general procedure C was followed using arylidene urea **S29** (54.9 mg, 0.2 mmol), dimethyl phosphite (21 µL, 0.233 mmol, 1.2 equiv.), (*S*)-TiPSY (8.7 mg, 0.01 mmol, 0.05 equiv.) in PhMe (2 mL) at 30 °C for 40 h. Purification by flash column chromatography using hexane/^{i}PrOH 90:10 as eluent. Product (*S*)**-28** was obtained as a white solid (64 mg, 0.167 mmol, 83%).

^{1}H-NMR (600 MHz, DMSO-*d*$_6$) δ 8.92 (s, 1H), 8.04 (s, 1H), 7.78 (d, J = 8.9 Hz, 2H), 7.75 – 7.70 (m, 1H), 7.47 – 7.35 (m, 7H), 7.35 – 7.29 (m, 2H), 5.37 (dd, J = 21.7, 9.6 Hz, 1H), 3.71 (d, J = 10.6 Hz, 3H), 3.52 (d, J = 10.5 Hz, 3H). **^{13}C-NMR** (151 MHz, DMSO-*d*$_6$) δ 154.2 (d, J = 10.5 Hz), 137.5, 136.4, 136.4, 133.7, 128.9, 128.4, 127.8, 127.7, 127.4, 126.9, 126.3, 123.8, 119.3, 112.7, 53.4 (d, J = 7.0 Hz), 53.2 (d, J = 6.7 Hz), 49.5 (d, J = 153.4 Hz). **^{31}P{^{1}H}-NMR** (162 MHz, DMSO-*d*$_6$) δ 24.70. **HRMS (ESI)** calculated for: $C_{20}H_{22}N_2O_4P$ [M^+ + 1]: 385.13117, found: 385.13064.

Enantiomeric purity: 90.5:9.5 *e.r.* (crude, by $^{31}P\{^{1}H\}$-NMR in $CDCl_3$), 93:7 *e.r.* (by $^{31}P\{^{1}H\}$-NMR in $CDCl_3$).

$[\alpha]_D^{20}$ = –42.7 (3.78, DMSO)

7.4. Unsuccessful arylidene ureas

Arylidene ureas **S30** and **S31**, prepared following literature procedures,[8] did not provide the desired α-ureidophosphonates under the optimized conditions.

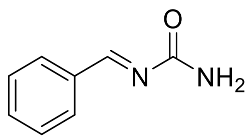

S30

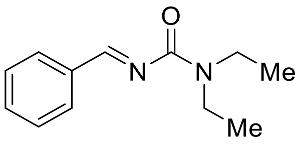

S31

The preparation of a *N*-alkyl substituted arylidene urea **S32** and thiourea derivative **S33**, proved to be unsuccessful. Consequently, these compounds could not be tested in the enantioselective hydrophosphonylation reaction.

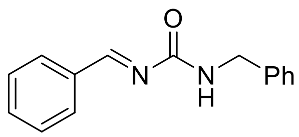

S32

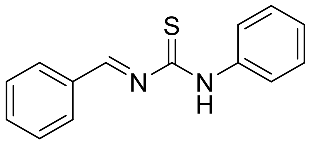

S33

8. X-ray crystallographic analysis of 4

Single crystals of $C_{28}H_{27}N_2O_4P$ of *rac*-**4** were grown from a saturated solution in hexane/^{i}PrOH 95:5 at room temperature. A suitable crystal was selected and analyzed on a 'Bruker APEX-II CCD' diffractometer. The crystal was kept at 100.0 K during data collection. Using Olex2,[29] the structure was solved with the ShelXT[30] structure solution program using Intrinsic Phasing and refined with the ShelXL[31] refinement package using Least Squares minimization.

CCDC 2193600 contains the supplementary crystallographic data for this paper. These data can be obtained free of charge from The Cambridge Crystallographic Data Centre via www.ccdc.cam.ac.uk/data_request/cif.

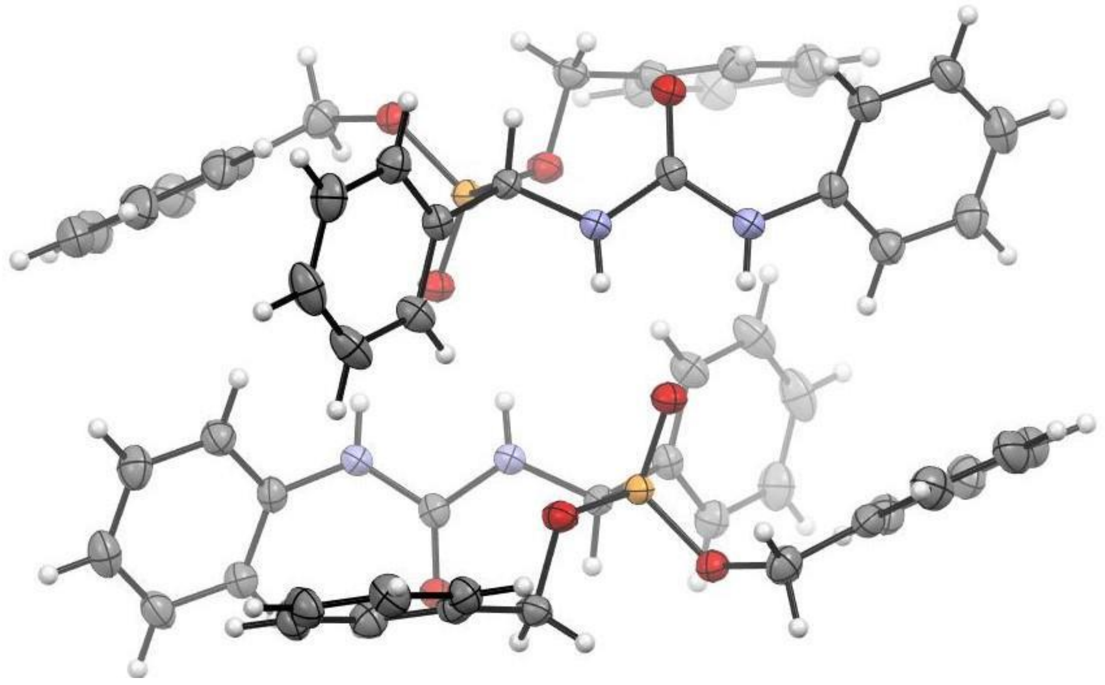

Table S9 Crystal data and structure refinement .

Identification code	mo_DSV598_0m_a
Empirical formula	$C_{28}H_{27}N_2O_4P$
Formula weight	486.48
Temperature/K	100.0
Crystal system	monoclinic
Space group	$P2_1/n$
a/Å	10.2404(7)
b/Å	13.3528(11)
c/Å	18.2585(16)
α/°	90
β/°	103.998(3)

[29] Dolomanov, O.V., Bourhis, L.J., Gildea, R.J, Howard, J.A.K. & Puschmann, H. *J. Appl. Cryst.* **2009**, *42*, 339-341.
[30] Sheldrick, G.M. *Acta Cryst*. **2015**, *A71*, 3-8.
[31] Sheldrick, G.M. *Acta Cryst.* **2015**, *C71*, 3-8.

γ/°	90
Volume/Å^3	2422.5(3)
Z	4
ρ_{calc}g/cm^3	1.334
μ/mm^{-1}	0.152
F(000)	1024.0
Crystal size/mm^3	0.224 × 0.113 × 0.097
Radiation	MoKα (λ = 0.71073)
2Θ range for data collection/°	5.11 to 65.328
Index ranges	-13 ≤ h ≤ 15, -20 ≤ k ≤ 20, -27 ≤ l ≤ 27
Reflections collected	99995
Independent reflections	8862 [R_{int} = 0.1075, R_{sigma} = 0.0536]
Data/restraints/parameters	8862/0/317
Goodness-of-fit on F^2	1.017
Final R indexes [I>=2σ (I)]	R_1 = 0.0484, wR_2 = 0.1056
Final R indexes [all data]	R_1 = 0.0830, wR_2 = 0.1219
Largest diff. peak/hole / e Å^{-3}	0.47/-0.48

Table S10 Fractional Atomic Coordinates (×10^4) and Equivalent Isotropic Displacement Parameters (Å^2×10^3) for mo_DSV598_0m_a. U_{eq} is defined as 1/3 of of the trace of the orthogonalized U_{IJ} tensor.

Atom	*x*	*y*	*z*	U(eq)
P1	7448.1(3)	5554.9(3)	5511.8(2)	14.80(8)
O1	7874.0(9)	4491.8(7)	3694.8(6)	18.3(2)
O3	8758.2(9)	6184.5(7)	5842.0(5)	17.7(2)
O4	6355.1(10)	5657.0(7)	5905.8(6)	18.9(2)
O2	7847.9(10)	4416.2(7)	5535.0(6)	18.9(2)
N1	6138.4(11)	5257.9(9)	4055.9(7)	17.4(2)
N2	5757.5(11)	3784.9(9)	3399.7(7)	19.5(2)
C16	9127.9(13)	2936.3(10)	5462.4(8)	17.2(3)
C23	8722.3(14)	7357.4(11)	6868.1(8)	20.1(3)
C8	6682.2(13)	4503.8(10)	3716.2(7)	15.7(2)
C1	7022.1(13)	5980.0(10)	4532.3(8)	15.4(2)
C15	9161.9(13)	4054.8(10)	5456.5(8)	18.6(3)
C22	9226.9(14)	6370.6(11)	6647.9(8)	21.7(3)
C9	6090.5(13)	2845.4(10)	3138.4(8)	17.1(3)
C2	6438.6(13)	7031.8(10)	4475.4(7)	16.3(2)
C13	5619.3(16)	1077.3(11)	3016.3(9)	23.9(3)
C12	6661.6(16)	943.2(12)	2661.0(9)	25.5(3)
C10	7107.8(14)	2722.7(11)	2757.0(8)	20.2(3)
C19	9102.6(16)	855.0(11)	5456.9(9)	24.8(3)
C21	8889.3(14)	2413.8(11)	4784.0(8)	20.8(3)
C14	5333.9(14)	2019.7(11)	3259.1(8)	20.4(3)
C28	8325.8(16)	8128.5(11)	6349.6(9)	24.2(3)
C18	9334.1(15)	1368.4(11)	6136.8(9)	23.8(3)
C17	9343.0(14)	2408.7(11)	6140.2(8)	19.9(3)
C11	7399.6(15)	1770.0(12)	2531.4(8)	23.2(3)
C7	5213.0(14)	7231.0(11)	4656.7(8)	20.6(3)
C24	8698.2(16)	7511.5(14)	7619.0(9)	28.6(3)
C3	7151.4(14)	7818.6(11)	4255.5(8)	20.4(3)
C20	8875.9(16)	1375.5(11)	4780.3(9)	24.5(3)
C27	7925.5(17)	9045.0(12)	6584.7(10)	29.4(3)
C4	6648.3(17)	8790.0(11)	4213.4(9)	25.6(3)

C6	4711.5(16)	8202.8(12)	4615.8(9)	26.0(3)
C5	5433.7(18)	8982.7(12)	4396.2(9)	28.0(3)
C26	7943.2(17)	9202.8(13)	7335.9(10)	31.6(4)
C25	8317.2(17)	8435.0(14)	7851.4(10)	32.7(4)

Table S11 Anisotropic Displacement Parameters (Å^2×10^3) for mo_DSV598_0m_a. The Anisotropic displacement factor exponent takes the form: $-2\pi^2[h^2a^{*2}U_{11}+2hka^*b^*U_{12}+\ldots]$.

Atom	U_{11}	U_{22}	U_{33}	U_{23}	U_{13}	U_{12}
P1	13.73(14)	13.28(15)	18.06(16)	-0.62(12)	5.17(12)	-0.27(12)
O1	16.6(4)	18.8(5)	21.2(5)	-2.3(4)	7.8(4)	-0.5(4)
O3	16.8(4)	19.6(5)	17.7(5)	-2.4(4)	5.9(4)	-3.0(4)
O4	17.2(4)	19.8(5)	21.7(5)	-0.8(4)	8.4(4)	-1.0(4)
O2	16.1(4)	14.7(4)	27.2(5)	1.0(4)	8.2(4)	0.7(4)
N1	13.8(5)	17.0(5)	21.8(6)	-4.6(4)	4.9(4)	-0.3(4)
N2	15.3(5)	18.4(5)	26.2(6)	-6.5(5)	7.8(4)	-0.9(4)
C16	14.5(5)	15.5(6)	22.1(7)	1.2(5)	5.3(5)	1.4(5)
C23	17.1(6)	23.4(7)	20.0(7)	-4.1(5)	5.0(5)	-3.9(5)
C8	17.7(6)	14.8(6)	14.8(6)	0.3(5)	4.2(5)	0.9(5)
C1	14.8(5)	13.7(6)	18.4(6)	-1.9(5)	5.5(5)	-1.7(4)
C15	16.0(6)	16.6(6)	24.3(7)	0.7(5)	7.3(5)	1.7(5)
C22	21.1(6)	23.6(7)	18.9(7)	-1.4(5)	2.0(5)	-0.8(5)
C9	19.1(6)	16.0(6)	15.6(6)	-2.9(5)	3.0(5)	0.1(5)
C2	18.5(6)	14.8(6)	15.3(6)	-0.9(5)	3.5(5)	1.3(5)
C13	30.3(7)	17.5(7)	23.9(7)	-0.9(5)	6.9(6)	-2.0(6)
C12	32.5(8)	19.5(7)	23.8(7)	-4.6(6)	5.5(6)	3.8(6)
C10	19.8(6)	21.4(7)	19.6(6)	-3.9(5)	5.5(5)	-0.9(5)
C19	28.6(7)	15.9(6)	32.5(8)	-0.2(6)	12.2(6)	3.0(6)
C21	21.9(6)	20.4(7)	20.7(7)	0.8(5)	6.3(5)	0.2(5)
C14	22.3(6)	19.6(7)	20.4(7)	-1.8(5)	7.5(5)	-1.2(5)
C28	28.8(7)	22.5(7)	24.0(7)	-3.3(6)	11.9(6)	-4.9(6)
C18	27.9(7)	20.1(7)	23.8(7)	5.0(6)	7.3(6)	3.4(6)
C17	21.1(6)	19.1(6)	19.5(6)	0.1(5)	4.9(5)	1.9(5)
C11	23.5(7)	25.9(7)	21.0(7)	-5.9(6)	6.5(5)	4.0(6)
C7	21.5(6)	20.7(7)	20.6(7)	1.4(5)	6.8(5)	4.1(5)
C24	26.8(7)	38.6(9)	19.0(7)	-3.0(6)	3.1(6)	2.9(7)
C3	21.7(6)	18.2(6)	21.0(7)	-0.3(5)	4.4(5)	-0.7(5)
C20	29.1(7)	21.3(7)	24.8(7)	-4.4(6)	9.8(6)	-0.4(6)
C27	33.4(8)	20.4(7)	37.6(9)	-4.0(7)	14.7(7)	-4.0(6)
C4	34.1(8)	16.8(6)	24.9(7)	0.8(6)	5.0(6)	-0.6(6)
C6	29.6(7)	27.6(8)	22.0(7)	1.2(6)	8.4(6)	12.3(6)
C5	41.6(9)	19.8(7)	21.8(7)	0.2(6)	6.3(6)	10.9(6)
C26	26.7(7)	29.9(8)	40.1(10)	-14.5(7)	12.0(7)	-3.4(6)
C25	27.7(8)	45.8(10)	24.7(8)	-13.9(7)	6.5(6)	1.1(7)

Table S12 Bond Lengths.

Atom	Atom	Length/Å		Atom	Atom	Length/Å
P1	O3	1.5744(10)		C9	C14	1.3954(19)
P1	O4	1.4760(10)		C2	C7	1.3987(19)

P1	O2	1.5726(10)		C2	C3	1.3927(19)
P1	C1	1.8258(14)		C13	C12	1.388(2)
O1	C8	1.2305(16)		C13	C14	1.389(2)
O3	C22	1.4546(17)		C12	C11	1.390(2)
O2	C15	1.4688(16)		C10	C11	1.392(2)
N1	C8	1.3696(17)		C19	C18	1.387(2)
N1	C1	1.4576(17)		C19	C20	1.387(2)
N2	C8	1.3739(17)		C21	C20	1.387(2)
N2	C9	1.4129(17)		C28	C27	1.391(2)
C16	C15	1.4940(19)		C18	C17	1.389(2)
C16	C21	1.391(2)		C7	C6	1.391(2)
C16	C17	1.395(2)		C24	C25	1.390(2)
C23	C22	1.505(2)		C3	C4	1.391(2)
C23	C28	1.391(2)		C27	C26	1.383(2)
C23	C24	1.393(2)		C4	C5	1.388(2)
C1	C2	1.5199(18)		C6	C5	1.391(2)
C9	C10	1.3956(19)		C26	C25	1.382(3)

Table S13 Bond angles.

Atom	Atom	Atom	Angle/°		Atom	Atom	Atom	Angle/°
O3	P1	C1	100.87(6)		C14	C9	N2	117.36(12)
O4	P1	O3	116.13(6)		C14	C9	C10	119.74(13)
O4	P1	O2	107.42(6)		C7	C2	C1	121.33(12)
O4	P1	C1	114.77(6)		C3	C2	C1	119.47(12)
O2	P1	O3	108.29(5)		C3	C2	C7	119.19(13)
O2	P1	C1	109.03(6)		C12	C13	C14	120.74(14)
C22	O3	P1	121.38(9)		C13	C12	C11	119.22(14)
C15	O2	P1	123.62(8)		C11	C10	C9	119.64(14)
C8	N1	C1	119.66(11)		C20	C19	C18	120.32(14)
C8	N2	C9	124.35(11)		C20	C21	C16	120.36(14)
C21	C16	C15	119.63(13)		C13	C14	C9	119.85(13)
C21	C16	C17	119.53(13)		C27	C28	C23	120.02(15)
C17	C16	C15	120.83(13)		C19	C18	C17	119.85(14)
C28	C23	C22	121.92(13)		C18	C17	C16	120.12(14)
C28	C23	C24	119.27(14)		C12	C11	C10	120.73(14)
C24	C23	C22	118.77(14)		C6	C7	C2	120.36(14)
O1	C8	N1	122.61(12)		C25	C24	C23	120.30(16)
O1	C8	N2	124.45(12)		C4	C3	C2	120.43(14)
N1	C8	N2	112.94(11)		C21	C20	C19	119.82(14)
N1	C1	P1	110.22(9)		C26	C27	C28	120.39(16)
N1	C1	C2	113.17(11)		C5	C4	C3	120.09(15)
C2	C1	P1	110.56(9)		C7	C6	C5	119.92(14)
O2	C15	C16	107.71(11)		C4	C5	C6	120.02(14)
O3	C22	C23	111.67(12)		C25	C26	C27	119.82(16)
C10	C9	N2	122.88(13)		C26	C25	C24	120.15(15)

Table S14 Hydrogen Atom Coordinates (Å×10^4) and Isotropic Displacement Parameters (Å^2×10^3).

Atom	*x*	*y*	*z*	U(eq)
H1	5260.01	5306.16	3986.89	21
H2	4900.76	3918.39	3356.9	23
H1A	7876.21	6009.41	4359.31	18
H15A	9886.24	4304.01	5879.38	22
H15B	9337.42	4298.73	4977.25	22
H22A	10223.14	6369.7	6789.09	26
H22B	8912.23	5824.51	6929.48	26
H13	5095.46	518.1	3094.21	29
H12	6868.18	294.04	2508.23	31
H10	7598.47	3286.49	2652.05	24
H19	9099.39	143.52	5454.71	30
H21	8734.55	2770.29	4320.63	25
H14	4625.05	2102.13	3506.86	24
H28	8328.39	8029.09	5834.72	29
H18	9486.08	1009.34	6598.89	29
H17	9495.99	2761.27	6605.09	24
H11	8111.02	1683.73	2286.07	28
H7	4720.49	6699.58	4808.68	25
H24	8943.24	6983.15	7974.19	34
H3	7986.62	7691.05	4133.4	24
H20	8711.93	1021.45	4315.6	29
H27	7638.22	9565.54	6227.37	35
H4	7136.85	9322.14	4059.31	31
H6	3876.92	8333.84	4737.61	31
H5	5095.22	9647.49	4371.41	34
H26	7698.89	9837.4	7496.86	38
H25	8314.06	8538.45	8365.74	39

9. Computational data

All electronic structure calculations were performed using the Gaussian 09 software package[32] at the CESGA facilities. The geometries of all minima were optimized at the ωb97XD[33] level within the self-consistent reaction field (SCRF) using the SMD model[34] (toluene). The 6-31G(d,p) basis set[35] was employed for all other atoms. Frequency calculations were performed at the same level to evaluate the zero-point vibrational energy and thermal corrections at 298 K and to confirm the nature of the stationary points. Single-point energies were calculated using ωb97XD within the self-consistent reaction field (SCRF) using the SMD model (dichloromethane) at the 6-311++G(d,p) level.[36] The schemes were built up in terms of ΔG_{sol}. All 3D representations were generated using CYLview.[37]

Cartesian coordinates in Å of the calculated structures. 6-31G(d,p)-optimized geometries (BS1) and 6-31G++(d,p) (BS2) electronic energies.

1) **Monomeric α-ureidophosphonate (*S*)-5**

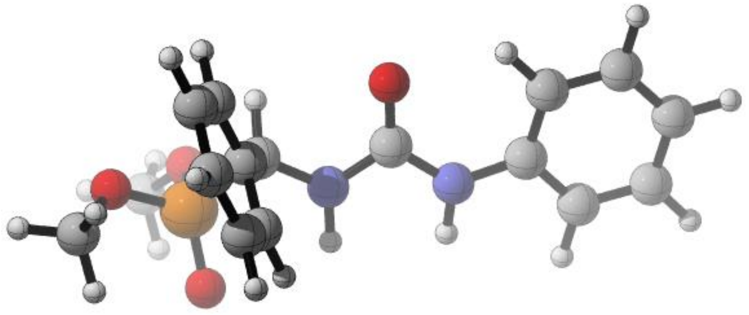

Electronic Energy BS1 = -1372.69706735 Hartree

Electronic Energy BS2 = -1372.95572023 Hartree

Zero-point Energy Correction = 0.339468 Hartree

---

[32] Frisch, M. J.; Trucks, G. W.; Schlegel, H. B.; Scuseria, G. E.; Robb, M. A.; Cheeseman, J. R.; Scalmani, G.; Barone, V.; Mennucci, B.; Petersson, G. A.; Nakatsuji, H.; Caricato, M.; Li, X.; Hratchian, H. P.; Izmaylov, A. F.; Bloino, J.; Zheng, G.; Sonnenberg, J. L.; Hada, M.; Ehara, M.; Toyota, K.; Fukuda, R.; Hasegawa, J.; Ishida, M.; Nakajima, T.; Honda, Y.; Kitao, O.; Nakai, H.; Vreven, T.; Montgomery, J. A., Jr.; Peralta, J. E.; Ogliaro, F.; Bearpark, M.; Heyd, J. J.; Brothers, E.; Kudin, K. N.; Staroverov, V. N.; Kobayashi, R.; Normand, J.; Raghavachari, K.; Rendell, A.; Burant, J. C.; Iyengar, S. S.; Tomasi, J.; Cossi, M.; Rega, N.; Millam, N. J.; Klene, M.; Knox, J. E.; Cross, J. B.; Bakken, V.; Adamo, C.; Jaramillo, J.; Gomperts, R.; Stratmann, R. E.; Yazyev, O.; Austin, A. J.; Cammi, R.; Pomelli, C.; Ochterski, J. W.; Martin, R. L.; Morokuma, K.; Zakrzewski, V. G.; Voth, G. A.; Salvador, P.; Dannenberg, J. J.; Dapprich, S.; Daniels, A. D.; Farkas, Ö.; Foresman, J. B.; Ortiz, J. V.; Cioslowski, J.; Fox, D. J. Gaussian 09, Revision D.01; Gaussian, Inc., Wallingford, CT, 2013.

[33] a) Chai, J.-D.; Head-Gordon, M. *J. Chem. Phys.* **2008**, *128*, 084106. b) Chai, J.-D.; Head-Gordon, M. *Phys. Chem. Chem. Phys.* **2008**, *10*, 6615-6620.

[34] Marenich, A. V.; Cramer, C. J.; Truhlar, D. G. *J. Phys. Chem. B* **2009**, *113*, 6378-6396.

[35] (a) Hariharan, P. C.; Pople, J. A. Theor. Chim. Acta 1973, 28, 213-222. (b) Hehre, W. J.; Ditchfield, R.; Pople, J. A. *J. Chem. Phys.* **1972**, *56*, 2257-2261.

[36] (a) Krishnan, R.; Binkley, J. S.; Seeger, R.; Pople, J. A. *J. Chem. Phys.* **1980**, *72*, 650-654. (b) McLean, A. D.; Chandler, G. S. *J. Chem. Phys.* **1980**, 72, 5639-5648.

[37] Legault, C. Y. CYLview, 1.0b; Université de Sherbrooke: Quebec, Canada, 2009; http://www.cylview.org.

Thermal Correction to Enthalpy = 0.363374 Hartree

Thermal Correction to Free Energy = 0.282918 Hartree

C	-1.056560000	-0.194127000	-0.458734000
C	1.377517000	-0.192721000	-0.329847000
N	2.455394000	-0.530973000	0.461811000
O	1.454091000	0.397345000	-1.396521000
H	-0.937457000	-0.327641000	-1.537486000
H	2.263357000	-1.099401000	1.272418000
N	0.169586000	-0.608003000	0.188800000
H	0.092556000	-0.928322000	1.144477000
P	-2.349909000	-1.357056000	0.118439000
O	-2.245982000	-1.640870000	1.575027000
O	-2.157463000	-2.592483000	-0.876721000
O	-3.765789000	-0.771000000	-0.356695000
C	-2.903782000	-3.795765000	-0.654218000
H	-3.966619000	-3.619420000	-0.841059000
H	-2.520424000	-4.526493000	-1.365289000
H	-2.755070000	-4.153912000	0.367677000
C	-4.548040000	0.075107000	0.500319000
H	-5.589583000	-0.080364000	0.218245000
H	-4.397145000	-0.197240000	1.547393000
H	-4.267615000	1.118847000	0.337640000
C	3.820543000	-0.283737000	0.224040000
C	4.733989000	-0.833608000	1.132745000
C	4.293059000	0.482004000	-0.847033000
C	6.098348000	-0.623274000	0.976906000
H	4.374006000	-1.430142000	1.967997000
C	5.664297000	0.682345000	-0.989357000
H	3.592413000	0.904609000	-1.553144000
C	6.574968000	0.137909000	-0.088330000
H	6.789089000	-1.058506000	1.692360000
H	6.019457000	1.277725000	-1.824923000
H	7.639993000	0.303036000	-0.212987000

C	-1.479643000	1.237322000	-0.173474000
C	-1.959851000	2.038569000	-1.209031000
C	-1.452891000	1.745196000	1.126626000
C	-2.415084000	3.328659000	-0.950071000
H	-1.972717000	1.651417000	-2.223798000
C	-1.903240000	3.036545000	1.387285000
H	-1.090312000	1.125538000	1.941630000
C	-2.390069000	3.829882000	0.350085000
H	-2.781052000	3.945161000	-1.765016000
H	-1.878098000	3.421298000	2.401953000
H	-2.741234000	4.836732000	0.552886000

2) Parallel heterochiral dimer of 5 (A in Figure 5)

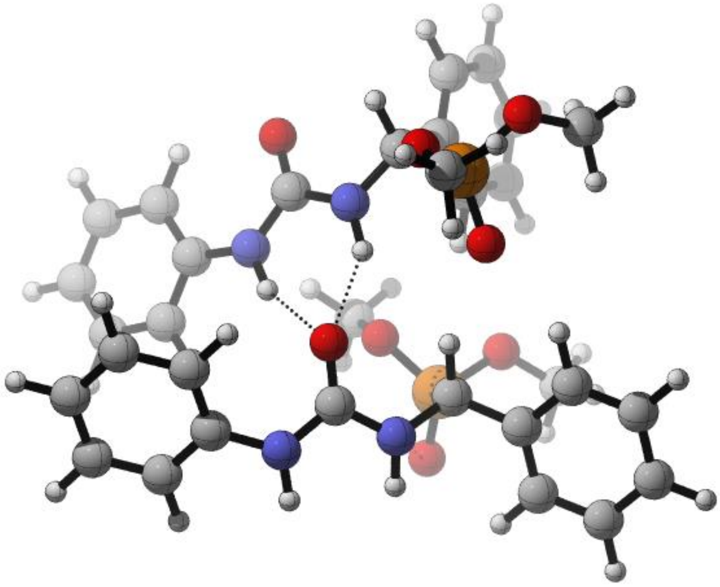

Electronic Energy BS1 = -2745.43658539 Hartree

Electronic Energy BS2 = -2745.94478364 Hartree

Zero-point Energy Correction = 0.681559 Hartree

Thermal Correction to Enthalpy = 0.72965 Hartree

Thermal Correction to Free Energy = 0.594594 Hartree

C	-0.900108000	-2.674840000	0.923942000
C	1.377226000	-2.309196000	0.167617000
N	2.333958000	-1.342884000	-0.053197000
O	1.505056000	-3.501304000	-0.089034000

H	-0.563147000	-3.589144000	1.424094000
H	2.096694000	-0.407226000	0.262591000
N	0.229887000	-1.801288000	0.726830000
H	0.113825000	-0.795319000	0.800099000
P	-2.043485000	-1.821285000	2.066554000
O	-2.305916000	-0.399163000	1.705791000
O	-1.360400000	-2.070588000	3.487755000
O	-3.368718000	-2.722636000	2.163394000
C	-1.811957000	-1.326683000	4.625659000
H	-2.826850000	-1.629944000	4.898043000
H	-1.125693000	-1.567463000	5.436670000
H	-1.782226000	-0.254750000	4.415005000
C	-4.491903000	-2.485563000	1.301430000
H	-5.372699000	-2.833035000	1.842030000
H	-4.583965000	-1.420124000	1.076938000
H	-4.370821000	-3.053049000	0.375331000
C	-1.190858000	2.168696000	-0.066668000
C	1.052819000	2.175032000	0.924728000
N	2.194529000	2.922493000	1.100771000
O	0.989220000	0.980668000	1.210253000
H	-1.297151000	1.237350000	0.497548000
H	2.191331000	3.860858000	0.730111000
N	-0.020126000	2.874330000	0.439917000
H	0.153379000	3.777980000	0.022618000
P	-0.894524000	1.751224000	-1.833519000
O	-0.441568000	2.910050000	-2.647674000
O	0.112124000	0.521080000	-1.687447000
O	-2.228553000	1.030832000	-2.376143000
C	0.622073000	-0.164051000	-2.843963000
H	-0.175623000	-0.754867000	-3.301324000
H	1.409017000	-0.823288000	-2.479774000
H	1.032176000	0.552321000	-3.559902000
C	-3.268813000	1.785530000	-3.012526000
H	-3.806624000	1.088286000	-3.655906000

H	-2.842500000	2.594988000	-3.609675000
H	-3.945014000	2.192073000	-2.255782000
C	3.477982000	2.387567000	1.367280000
C	4.580113000	3.004027000	0.767505000
C	3.668626000	1.281814000	2.199397000
C	5.861816000	2.509596000	0.984147000
H	4.432116000	3.859767000	0.113391000
C	4.954525000	0.783516000	2.388825000
H	2.819091000	0.804128000	2.668005000
C	6.054179000	1.387976000	1.786490000
H	6.708175000	2.993637000	0.507245000
H	5.091409000	-0.092905000	3.013777000
H	7.050124000	0.984786000	1.934580000
C	3.560917000	-1.481111000	-0.716577000
C	4.268069000	-0.303168000	-0.996198000
C	4.100892000	-2.712212000	-1.109556000
C	5.495402000	-0.351893000	-1.643095000
H	3.857564000	0.655144000	-0.691339000
C	5.330005000	-2.742660000	-1.765319000
H	3.557306000	-3.622968000	-0.899699000
C	6.037753000	-1.574360000	-2.035900000
H	6.027160000	0.574654000	-1.837915000
H	5.737461000	-3.703872000	-2.064625000
H	6.995807000	-1.614813000	-2.544130000
C	-2.461479000	2.982837000	0.070059000
C	-2.580976000	4.245654000	-0.519618000
C	-3.550559000	2.438538000	0.754523000
C	-3.770947000	4.960569000	-0.414423000
H	-1.757779000	4.659744000	-1.096290000
C	-4.742470000	3.153767000	0.851028000
H	-3.452931000	1.454820000	1.205511000
C	-4.855716000	4.414878000	0.269754000
H	-3.854102000	5.938916000	-0.877303000
H	-5.584144000	2.723946000	1.385462000

H	-5.785207000	4.970312000	0.347679000
C	-1.649811000	-3.053858000	-0.343916000
C	-2.014369000	-4.378826000	-0.578857000
C	-2.027289000	-2.068860000	-1.255877000
C	-2.756555000	-4.714216000	-1.708951000
H	-1.708595000	-5.151448000	0.120741000
C	-2.766137000	-2.400911000	-2.386911000
H	-1.746725000	-1.037600000	-1.080360000
C	-3.136879000	-3.725162000	-2.615043000
H	-3.029204000	-5.749779000	-1.887200000
H	-3.050312000	-1.618143000	-3.083618000
H	-3.712292000	-3.987701000	-3.497493000

3) Parallel homochiral dimer of 5 (B in Figure 5)

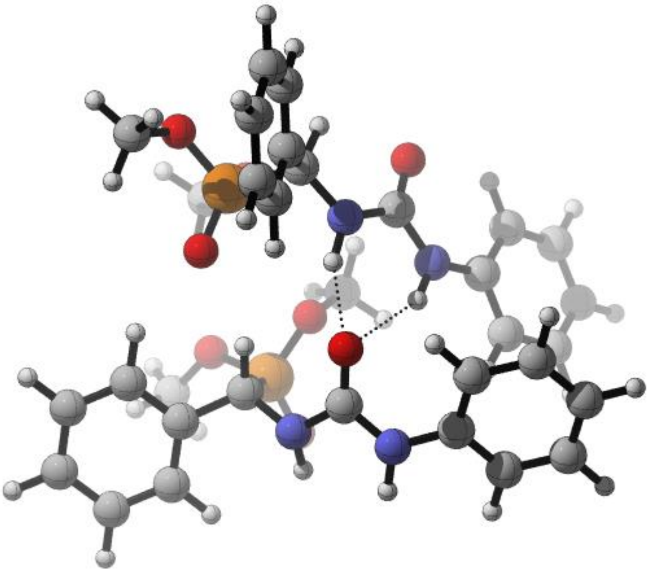

Electronic Energy BS1 = -2745.43756458 Hartree

Electronic Energy BS2 = -2745.94556952 Hartree

Zero-point Energy Correction = 0.682803 Hartree

Thermal Correction to Enthalpy = 0.730365 Hartree

Thermal Correction to Free Energy = 0.596498 Hartree

C	0.925428000	2.809454000	0.448029000
C	-1.293615000	2.004237000	1.069709000

N	-2.265627000	1.089219000	0.731484000
O	-1.369293000	2.785042000	2.012943000
H	0.675066000	3.462080000	1.289975000
H	-2.071426000	0.525553000	-0.087644000
N	-0.216575000	1.963161000	0.217998000
H	-0.125447000	1.171544000	-0.411673000
P	2.343103000	1.777002000	0.982656000
O	2.646970000	0.629998000	0.080334000
O	1.940250000	1.429498000	2.485479000
O	3.595584000	2.771034000	1.185043000
C	2.715134000	0.482528000	3.232200000
H	3.728175000	0.864638000	3.388788000
H	2.212853000	0.377244000	4.193858000
H	2.739259000	-0.479980000	2.716699000
C	4.552322000	2.959874000	0.133813000
H	5.456074000	3.343823000	0.607774000
H	4.761810000	2.011861000	-0.367004000
H	4.174147000	3.686894000	-0.590278000
C	1.149812000	-2.081781000	-0.662314000
C	-0.912902000	-1.411449000	-1.805929000
N	-2.073026000	-1.884525000	-2.372725000
O	-0.707174000	-0.213079000	-1.613645000
H	1.363209000	-1.011561000	-0.720132000
H	-2.159025000	-2.886301000	-2.458356000
N	0.002425000	-2.387081000	-1.507227000
H	-0.379841000	-3.311718000	-1.352508000
P	0.642621000	-2.458488000	1.069051000
O	-0.227065000	-3.665460000	1.132439000
O	0.022601000	-1.065748000	1.530103000
O	1.963443000	-2.554132000	1.984241000
C	-0.628327000	-0.921197000	2.803489000
H	-0.038259000	-1.398134000	3.591145000
H	-0.693467000	0.150413000	2.990404000
H	-1.628526000	-1.357163000	2.759755000

C	2.623906000	-3.809663000	2.197679000
H	3.125513000	-3.734540000	3.163189000
H	1.897288000	-4.625150000	2.215622000
H	3.359906000	-3.975311000	1.406483000
C	-3.290272000	-1.172924000	-2.483282000
C	-3.346763000	0.213028000	-2.651842000
C	-4.476480000	-1.910893000	-2.403887000
C	-4.587359000	0.845314000	-2.691196000
H	-2.434399000	0.787606000	-2.729330000
C	-5.707057000	-1.268218000	-2.466438000
H	-4.435259000	-2.988801000	-2.267680000
C	-5.769313000	0.117344000	-2.596314000
H	-4.621180000	1.924784000	-2.796864000
H	-6.618413000	-1.853093000	-2.394175000
H	-6.728594000	0.622854000	-2.621485000
C	-3.415970000	0.745528000	1.454797000
C	-3.835328000	1.409491000	2.614241000
C	-4.169558000	-0.334349000	0.974331000
C	-4.987915000	0.980606000	3.269838000
H	-3.262110000	2.247435000	2.985630000
C	-5.319261000	-0.744273000	1.635748000
H	-3.856808000	-0.848693000	0.070744000
C	-5.738117000	-0.091246000	2.793871000
H	-5.301273000	1.503921000	4.168400000
H	-5.887490000	-1.580372000	1.239231000
H	-6.635200000	-0.411341000	3.313797000
C	1.327280000	3.638015000	-0.756422000
C	1.645337000	4.987680000	-0.598640000
C	1.439721000	3.052803000	-2.020532000
C	2.072343000	5.745531000	-1.686760000
H	1.561237000	5.446186000	0.382727000
C	1.864048000	3.810078000	-3.108596000
H	1.202259000	2.001394000	-2.155323000
C	2.184808000	5.156899000	-2.944461000

H	2.312261000	6.795659000	-1.552108000
H	1.948031000	3.345710000	-4.086207000
H	2.516082000	5.745645000	-3.794206000
C	2.387226000	-2.874132000	-1.030313000
C	3.637072000	-2.255834000	-0.938523000
C	2.316714000	-4.225256000	-1.377781000
C	4.796995000	-2.981836000	-1.196329000
H	3.685815000	-1.209341000	-0.651170000
C	3.477999000	-4.948427000	-1.639508000
H	1.357605000	-4.731968000	-1.431629000
C	4.722233000	-4.328710000	-1.547190000
H	5.763107000	-2.491656000	-1.124758000
H	3.409514000	-5.997669000	-1.909186000
H	5.627897000	-4.892110000	-1.749239000

4) Antiparallel heterochiral dimer of 5 (C in Figure 5)

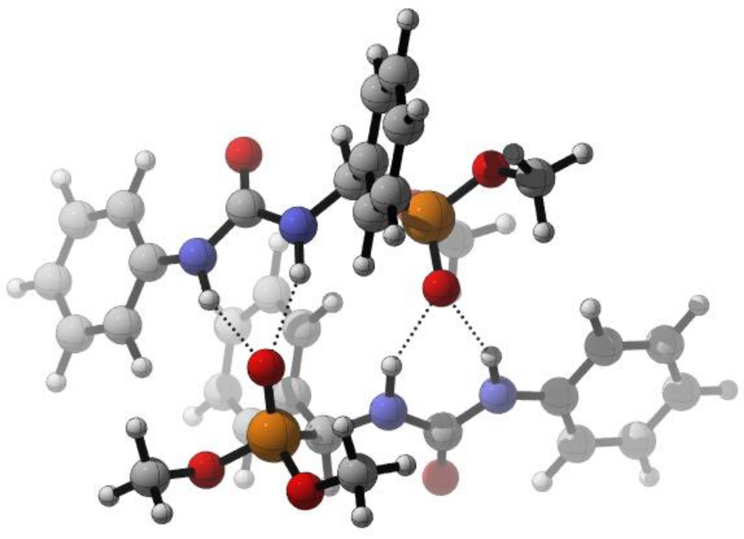

Electronic Energy BS1 = -2745.45455711 Hartree

Electronic Energy BS2 = -2745.96274332 Hartree

Zero-point Energy Correction = 0.682693 Hartree

Thermal Correction to Enthalpy = 0.730199 Hartree

Thermal Correction to Free Energy = 0.596608 Hartree

C	-0.586906000	-2.244017000	1.175154000
C	1.777418000	-2.602028000	0.702362000
N	2.925951000	-1.930163000	0.355975000
O	1.640534000	-3.816587000	0.720062000
H	-0.522836000	-3.247668000	1.607271000
H	2.854563000	-0.915143000	0.356994000
N	0.761535000	-1.731980000	1.068475000
H	0.886016000	-0.752476000	0.820838000
P	-1.383943000	-1.177601000	2.422814000
O	-1.437930000	0.271030000	2.044450000
O	-0.644509000	-1.428670000	3.821186000
O	-2.796907000	-1.880279000	2.646899000
C	0.493723000	-0.640899000	4.203360000
H	0.290161000	0.420589000	4.043462000
H	0.654543000	-0.837419000	5.262900000
H	1.367920000	-0.947722000	3.624995000
C	-3.762695000	-1.285533000	3.526880000
H	-4.672756000	-1.873819000	3.417946000
H	-3.407973000	-1.332461000	4.559324000
H	-3.954215000	-0.248224000	3.239227000
C	0.296181000	2.810811000	-0.695092000
C	-1.982941000	1.954765000	-0.944264000
N	-2.959372000	1.241532000	-0.292193000
O	-2.087613000	2.441022000	-2.063953000
H	0.009379000	3.184133000	-1.683815000
H	-2.672621000	0.843013000	0.597621000
N	-0.835168000	2.082923000	-0.186335000
H	-0.765576000	1.572635000	0.689196000
P	1.681975000	1.641199000	-0.953337000
O	1.962609000	0.784441000	0.246592000
O	1.241498000	0.859235000	-2.265455000
O	2.967526000	2.469531000	-1.434574000
C	1.952786000	-0.315557000	-2.694729000
H	3.003076000	-0.078976000	-2.883150000

H	1.470431000	-0.635395000	-3.616579000
H	1.872445000	-1.105122000	-1.943704000
C	3.937795000	2.953456000	-0.491668000
H	4.855066000	3.116929000	-1.056885000
H	4.105517000	2.215872000	0.296629000
H	3.593367000	3.894344000	-0.055054000
C	-4.096952000	0.655794000	-0.869762000
C	-4.671310000	-0.424547000	-0.188867000
C	-4.666927000	1.093946000	-2.069445000
C	-5.795374000	-1.060569000	-0.697784000
H	-4.205112000	-0.794778000	0.719916000
C	-5.789601000	0.439944000	-2.571939000
H	-4.220946000	1.923593000	-2.600655000
C	-6.363267000	-0.634777000	-1.897042000
H	-6.216113000	-1.906409000	-0.162325000
H	-6.221209000	0.784512000	-3.507053000
H	-7.238679000	-1.134261000	-2.299513000
C	4.104441000	-2.455595000	-0.192155000
C	5.048218000	-1.528914000	-0.658729000
C	4.375798000	-3.824301000	-0.296189000
C	6.243854000	-1.960691000	-1.218941000
H	4.838193000	-0.464805000	-0.575889000
C	5.578679000	-4.240222000	-0.862949000
H	3.648567000	-4.540873000	0.060241000
C	6.518163000	-3.323068000	-1.326653000
H	6.962481000	-1.227879000	-1.573325000
H	5.778895000	-5.304683000	-0.939486000
H	7.450806000	-3.663202000	-1.764503000
C	-1.369696000	-2.300104000	-0.126927000
C	-1.171141000	-1.344753000	-1.122408000
C	-2.313014000	-3.308551000	-0.331473000
C	-1.907150000	-1.386601000	-2.303024000
H	-0.448351000	-0.548718000	-0.981556000
C	-3.047036000	-3.355181000	-1.512644000

H	-2.470450000	-4.060582000	0.436673000
C	-2.849484000	-2.391631000	-2.499454000
H	-1.760488000	-0.613425000	-3.050766000
H	-3.780023000	-4.141984000	-1.660405000
H	-3.439406000	-2.414018000	-3.409824000
C	0.761486000	3.955872000	0.184026000
C	1.062888000	5.192607000	-0.388070000
C	0.952255000	3.773008000	1.556138000
C	1.550778000	6.234164000	0.397793000
H	0.915729000	5.340249000	-1.454237000
C	1.436734000	4.814134000	2.342602000
H	0.728961000	2.812560000	2.011917000
C	1.741037000	6.045843000	1.765001000
H	1.776442000	7.193276000	-0.057477000
H	1.579845000	4.662725000	3.407861000
H	2.119155000	6.856838000	2.379291000

5) Antiparallel homochiral dimer of 5 (D in Figure 5)

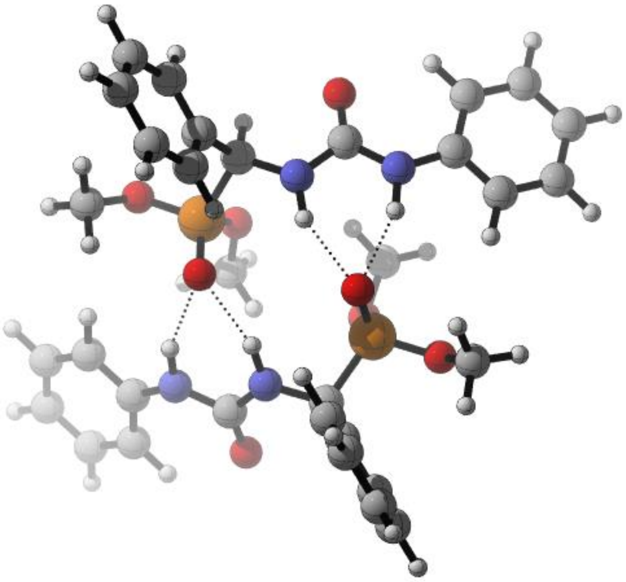

Electronic Energy BS1 = -2745.44820476 Hartree

Electronic Energy BS2 = -2745.95754218 Hartree

Zero-point Energy Correction = 0.681908 Hartree

Thermal Correction to Enthalpy = 0.729669 Hartree

Thermal Correction to Free Energy = 0.593881 Hartree

C	0.445867000	2.912963000	0.189848000
C	2.676222000	2.077736000	-0.320708000
N	3.498298000	0.981277000	-0.193240000
O	2.957252000	3.104078000	-0.927230000
H	0.737877000	3.562810000	-0.642084000
H	3.067574000	0.172893000	0.247536000
N	1.468883000	1.905788000	0.324758000
H	1.227368000	0.980311000	0.668506000
P	-1.109036000	2.092815000	-0.300613000
O	-1.530184000	0.995472000	0.628901000
O	-0.805847000	1.661960000	-1.800918000
O	-2.228007000	3.228057000	-0.464655000
C	-1.665944000	0.737182000	-2.493861000
H	-2.717472000	1.008229000	-2.368455000
H	-1.392582000	0.807309000	-3.545641000
H	-1.486111000	-0.275329000	-2.127231000
C	-3.072245000	3.609987000	0.633801000
H	-3.997202000	3.983398000	0.193881000
H	-3.279517000	2.751058000	1.277011000
H	-2.585552000	4.397869000	1.213461000
C	-0.445847000	-2.912919000	0.189833000
C	-2.676206000	-2.077712000	-0.320704000
N	-3.498297000	-0.981262000	-0.193252000
O	-2.957243000	-3.104084000	-0.927171000
H	-0.737840000	-3.562763000	-0.642108000
H	-3.067558000	-0.172825000	0.247422000
N	-1.468851000	-1.905730000	0.324713000
H	-1.227336000	-0.980249000	0.668444000
P	1.109072000	-2.092769000	-0.300570000
O	1.530138000	-0.995398000	0.628947000
O	0.805957000	-1.661953000	-1.800901000
O	2.228067000	-3.227996000	-0.464529000

C	1.666072000	-0.737192000	-2.493841000
H	2.717573000	-1.008463000	-2.368699000
H	1.392475000	-0.807068000	-3.545576000
H	1.486513000	0.275286000	-2.126984000
C	3.072229000	-3.609923000	0.633988000
H	3.997283000	-3.983183000	0.194144000
H	3.279321000	-2.751030000	1.277302000
H	2.585559000	-4.397915000	1.213516000
C	-4.685359000	-0.722540000	-0.893161000
C	-5.155525000	0.598725000	-0.874630000
C	-5.409958000	-1.697246000	-1.588678000
C	-6.326599000	0.942557000	-1.538523000
H	-4.594673000	1.357776000	-0.335103000
C	-6.580330000	-1.335399000	-2.252003000
H	-5.049688000	-2.716513000	-1.608053000
C	-7.048821000	-0.024052000	-2.235632000
H	-6.672511000	1.971474000	-1.511746000
H	-7.133391000	-2.100157000	-2.788919000
H	-7.962941000	0.241317000	-2.756317000
C	4.685339000	0.722488000	-0.893161000
C	5.155495000	-0.598780000	-0.874524000
C	5.409924000	1.697124000	-1.588790000
C	6.326543000	-0.942684000	-1.538423000
H	4.594652000	-1.357776000	-0.334910000
C	6.580272000	1.335204000	-2.252120000
H	5.049664000	2.716392000	-1.608245000
C	7.048751000	0.023855000	-2.235645000
H	6.672447000	-1.971601000	-1.511563000
H	7.133323000	2.099908000	-2.789124000
H	7.962851000	-0.241571000	-2.756336000
C	0.209301000	3.762734000	1.425629000
C	0.163693000	5.152503000	1.312225000
C	-0.014855000	3.168887000	2.669880000
C	-0.109436000	5.942511000	2.426833000

H	0.347395000	5.617938000	0.347884000
C	-0.285391000	3.957216000	3.784330000
H	0.014545000	2.087365000	2.763020000
C	-0.337846000	5.345454000	3.664559000
H	-0.136326000	7.023190000	2.328948000
H	-0.458723000	3.487326000	4.747341000
H	-0.549101000	5.959193000	4.534557000
C	-0.209317000	-3.762692000	1.425619000
C	-0.163655000	-5.152458000	1.312209000
C	0.014771000	-3.168844000	2.669882000
C	0.109462000	-5.942463000	2.426822000
H	-0.347305000	-5.617893000	0.347858000
C	0.285294000	-3.957169000	3.784337000
H	-0.014673000	-2.087324000	2.763026000
C	0.337805000	-5.345405000	3.664559000
H	0.136391000	-7.023141000	2.328932000
H	0.458572000	-3.487279000	4.747358000
H	0.549049000	-5.959142000	4.534562000

10. NMR spectra

^{1}H-NMR (600 MHz, $CDCl_3$)

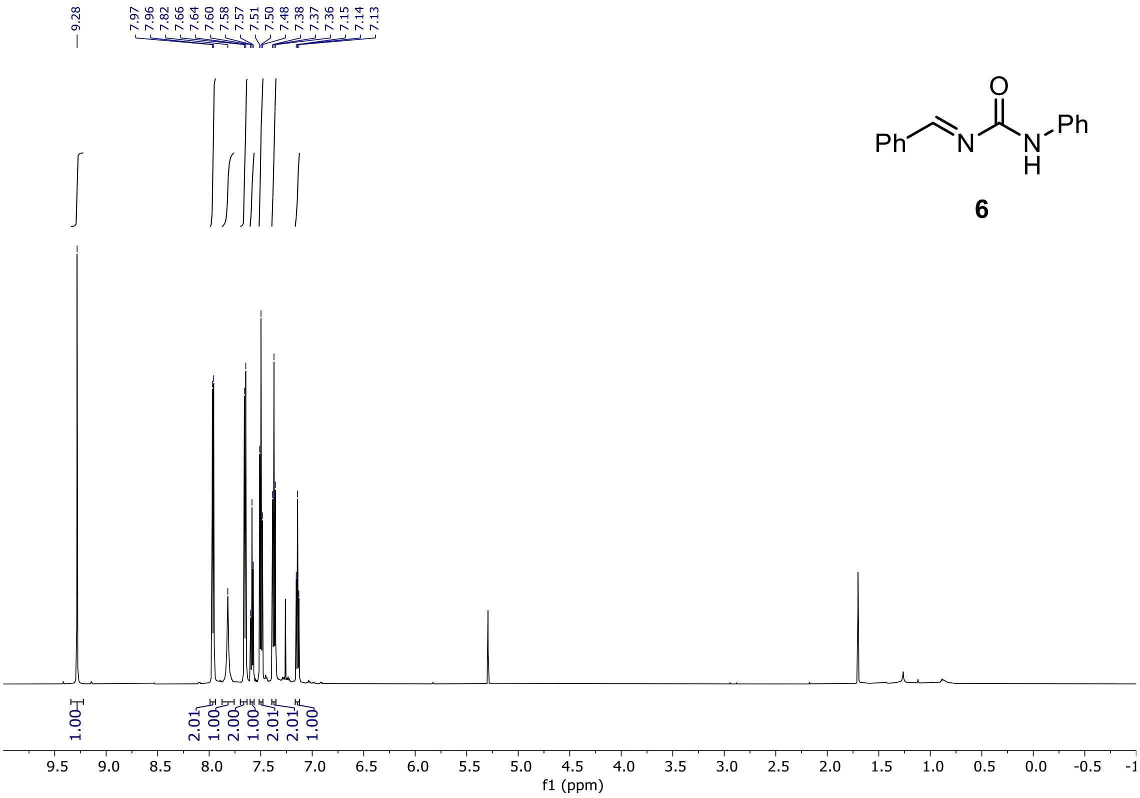

^{13}C-NMR (151 MHz, $CDCl_3$)

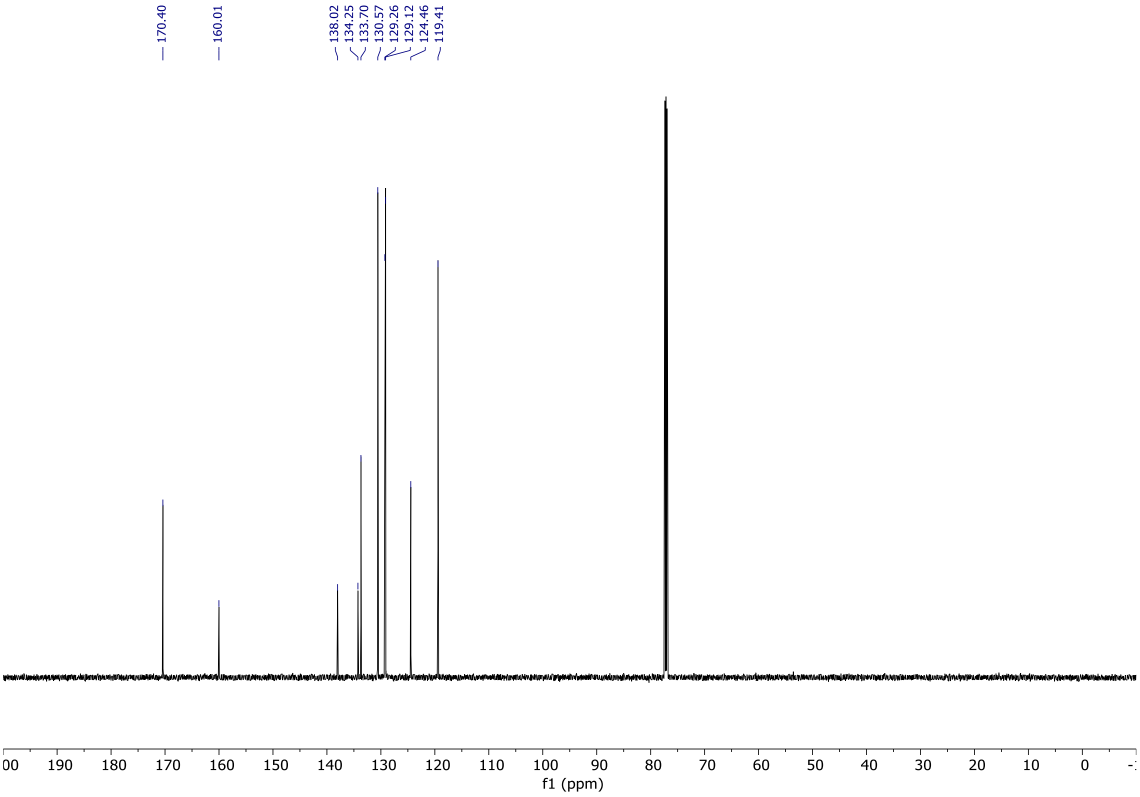

^{1}H-NMR (600 MHz, $CDCl_3$)

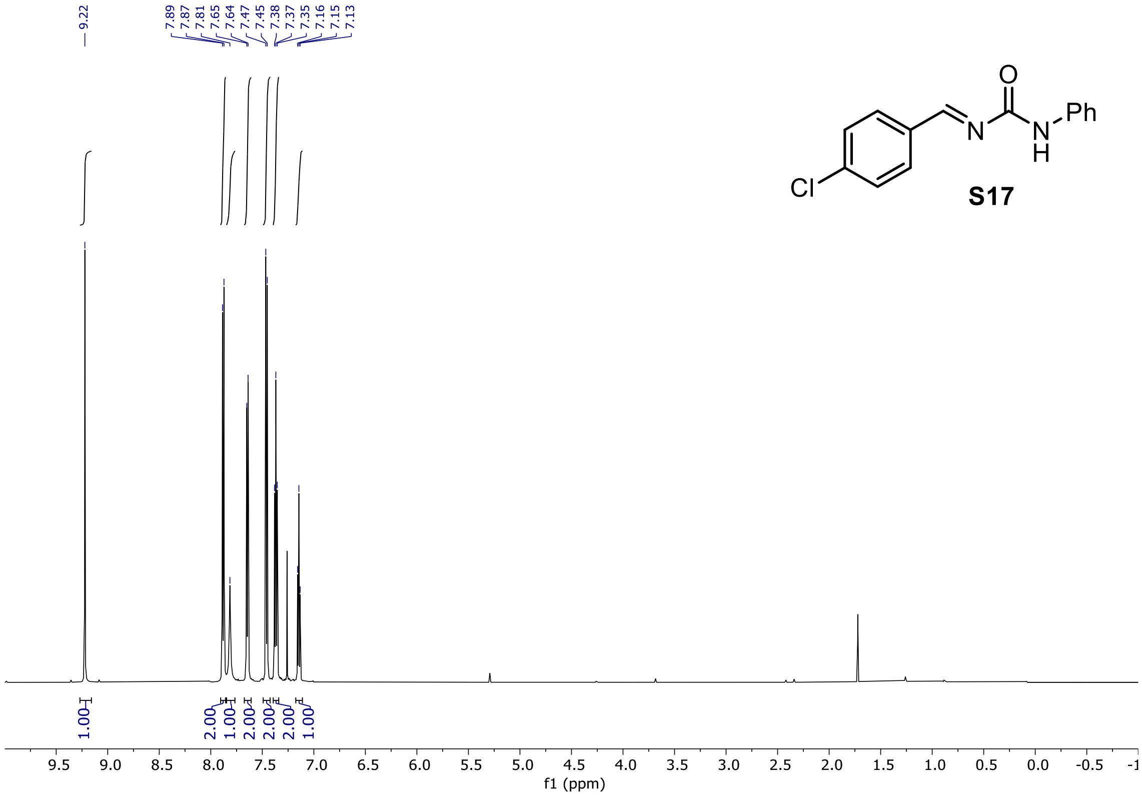

^{13}C-NMR (151 MHz, $CDCl_3$)

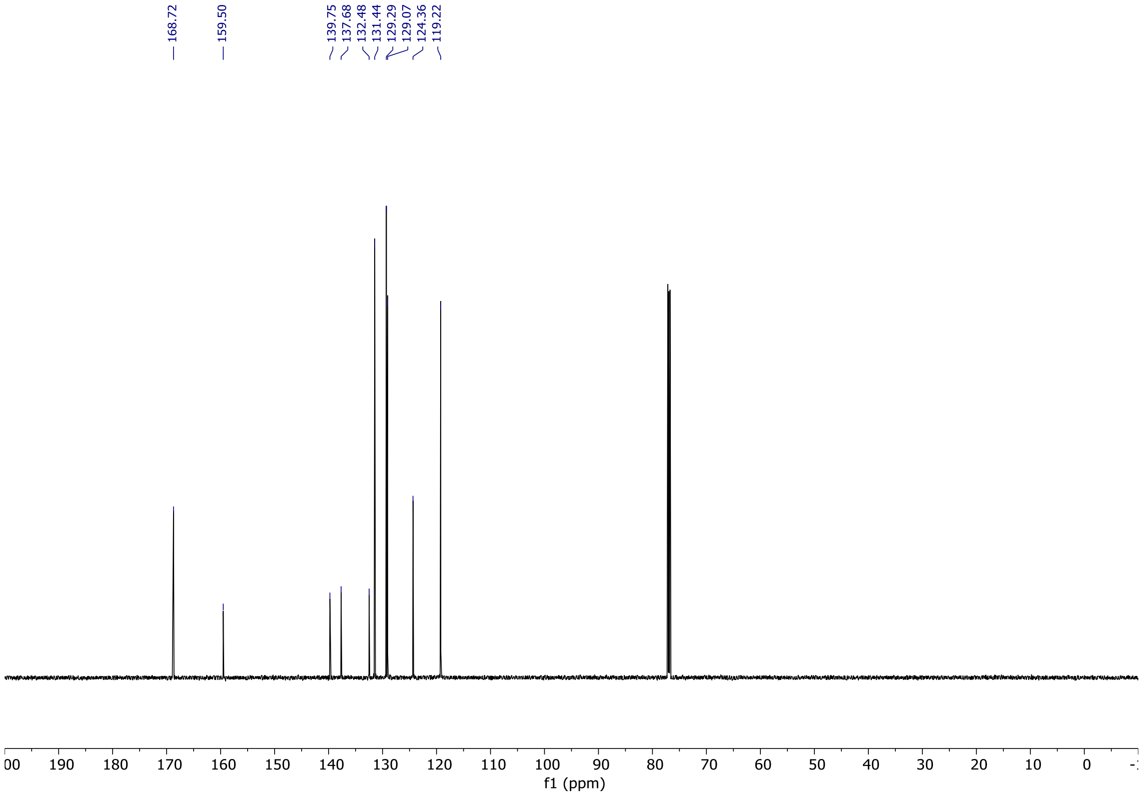

^{1}H-NMR (600 MHz, $CDCl_3$)

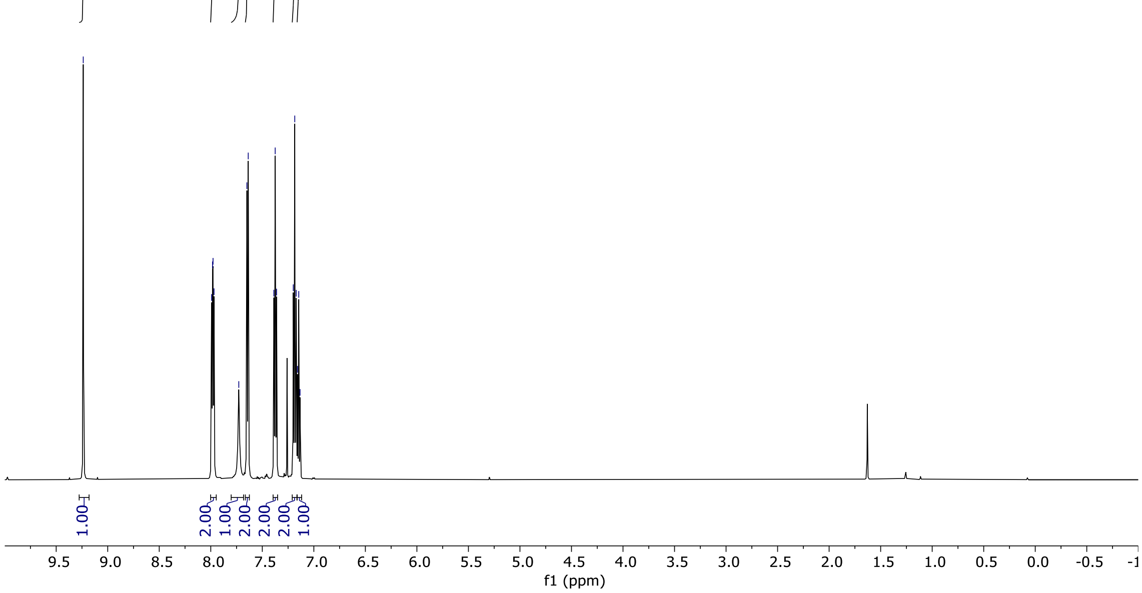

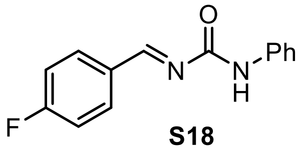

S18

^{13}C-NMR (151 MHz, $CDCl_3$)

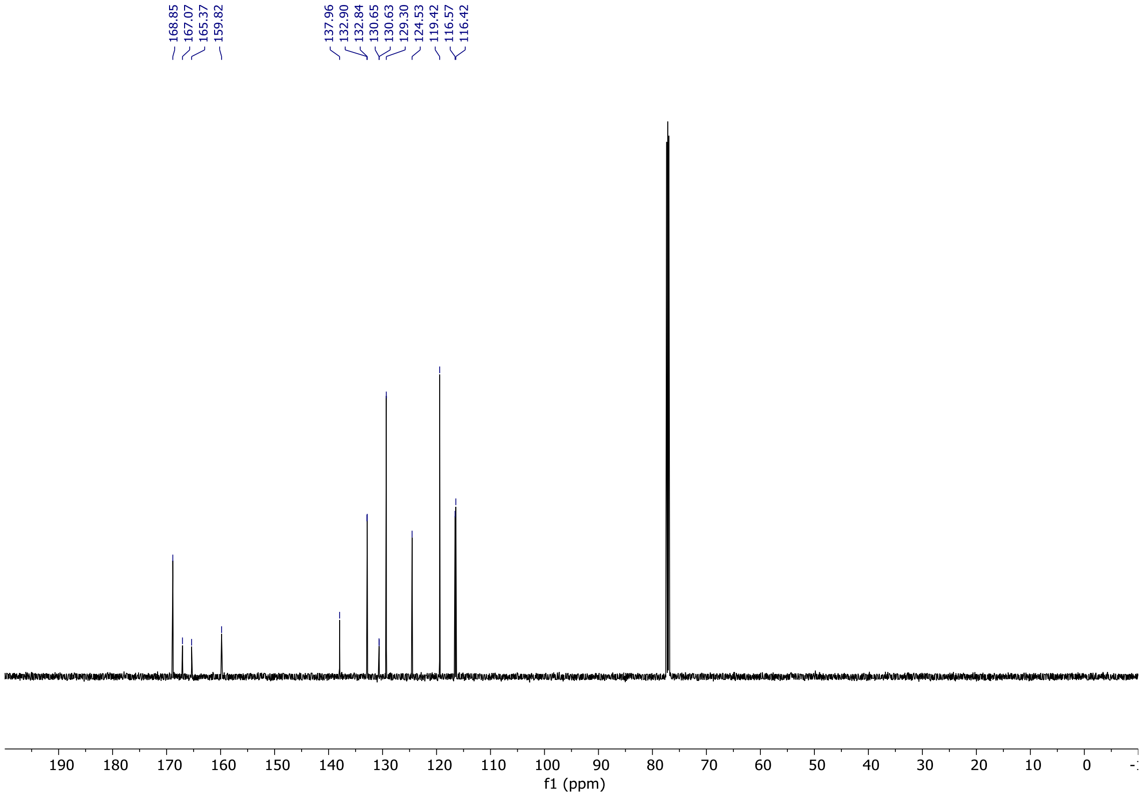

^{19}F-NMR (565 MHz, $CDCl_3$)

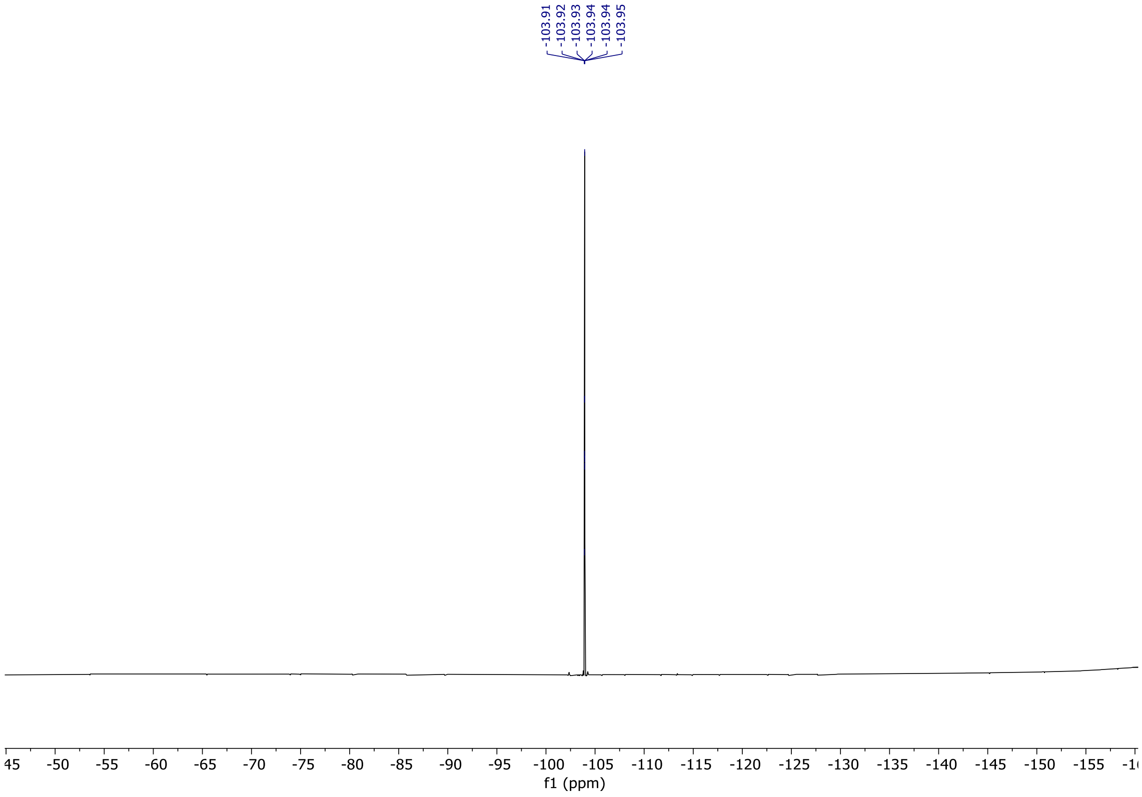

^{1}H-NMR (600 MHz, CDCl$_3$)

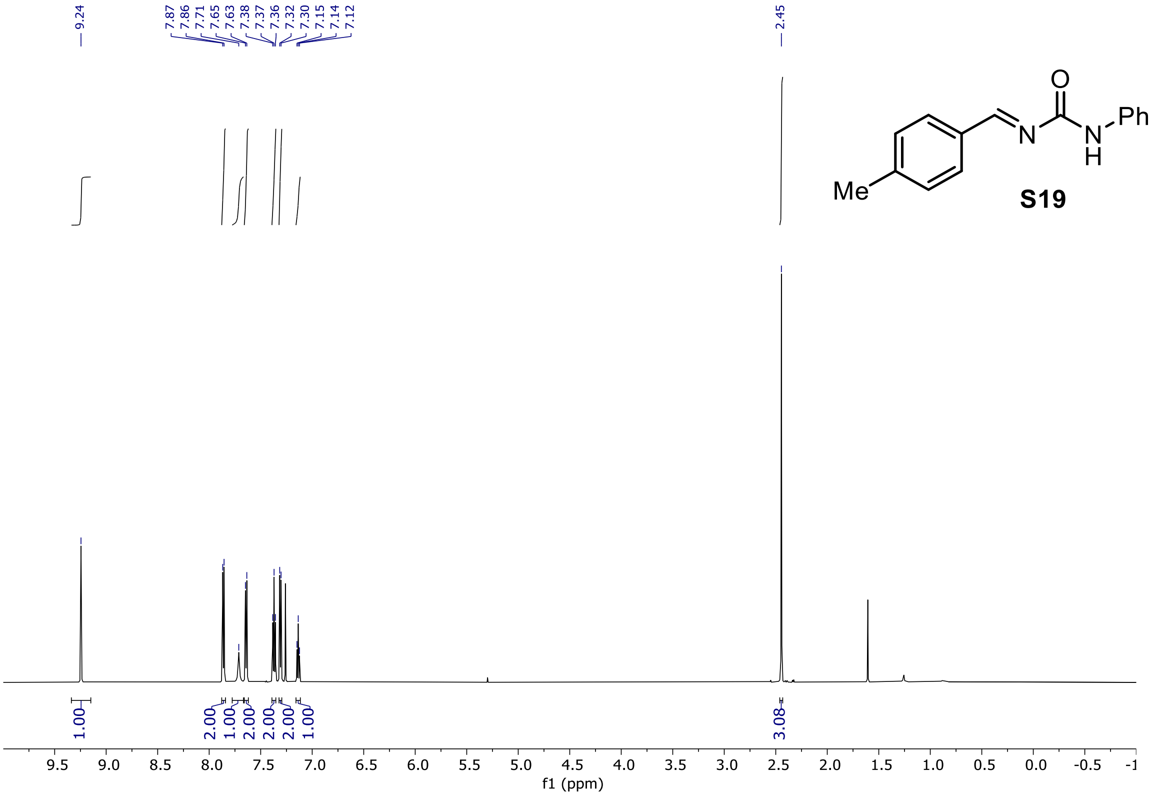

S19

^{13}C-NMR (151 MHz, CDCl$_3$)

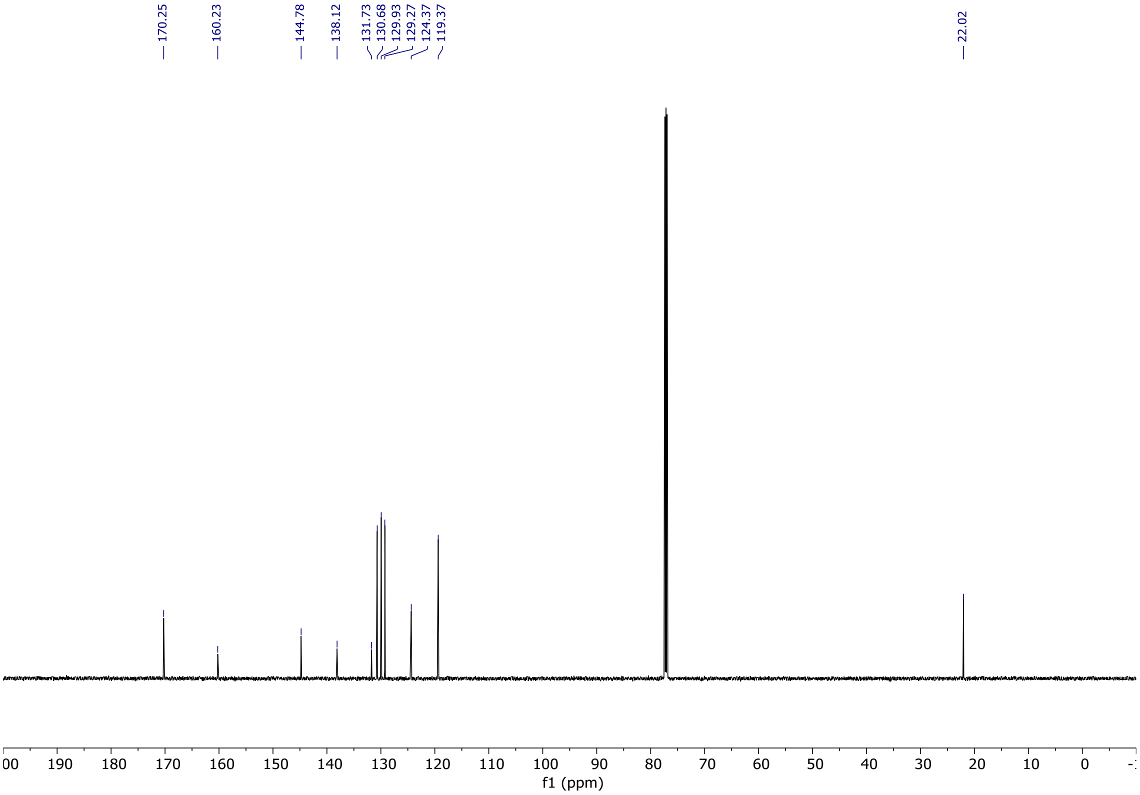

^{1}H-NMR (600 MHz, CDCl₃)

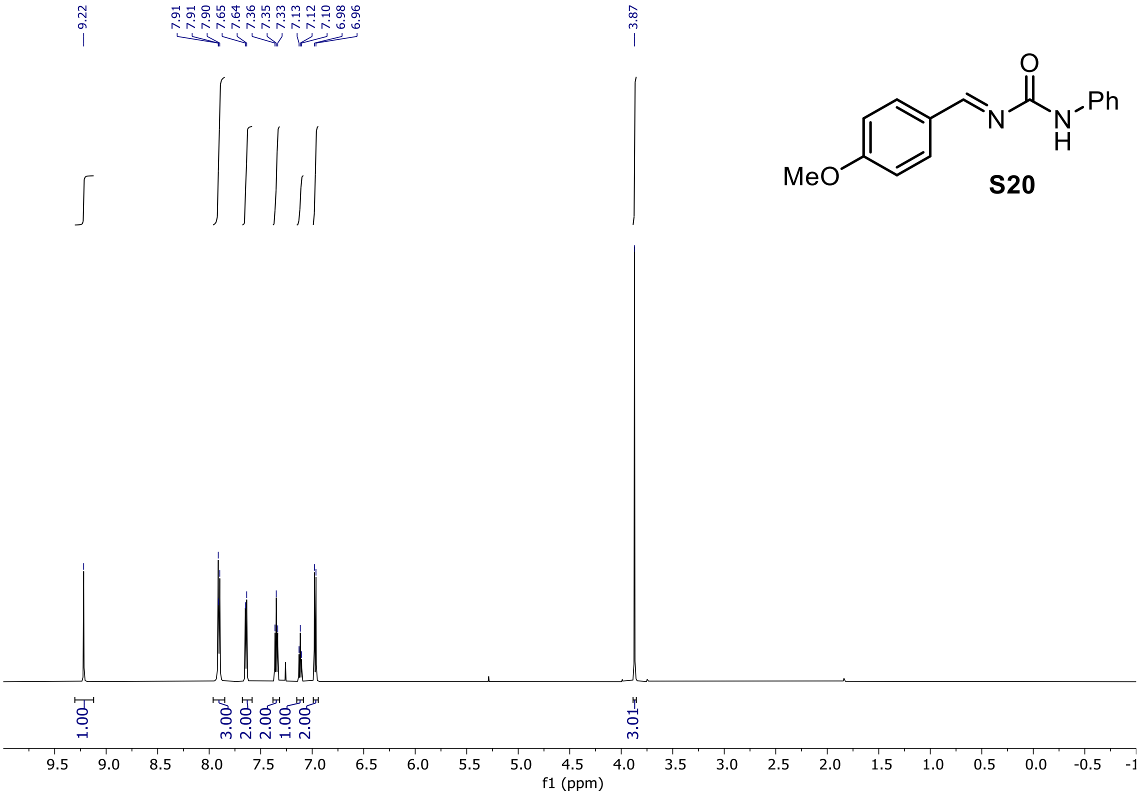

^{13}C-NMR (151 MHz, CDCl₃)

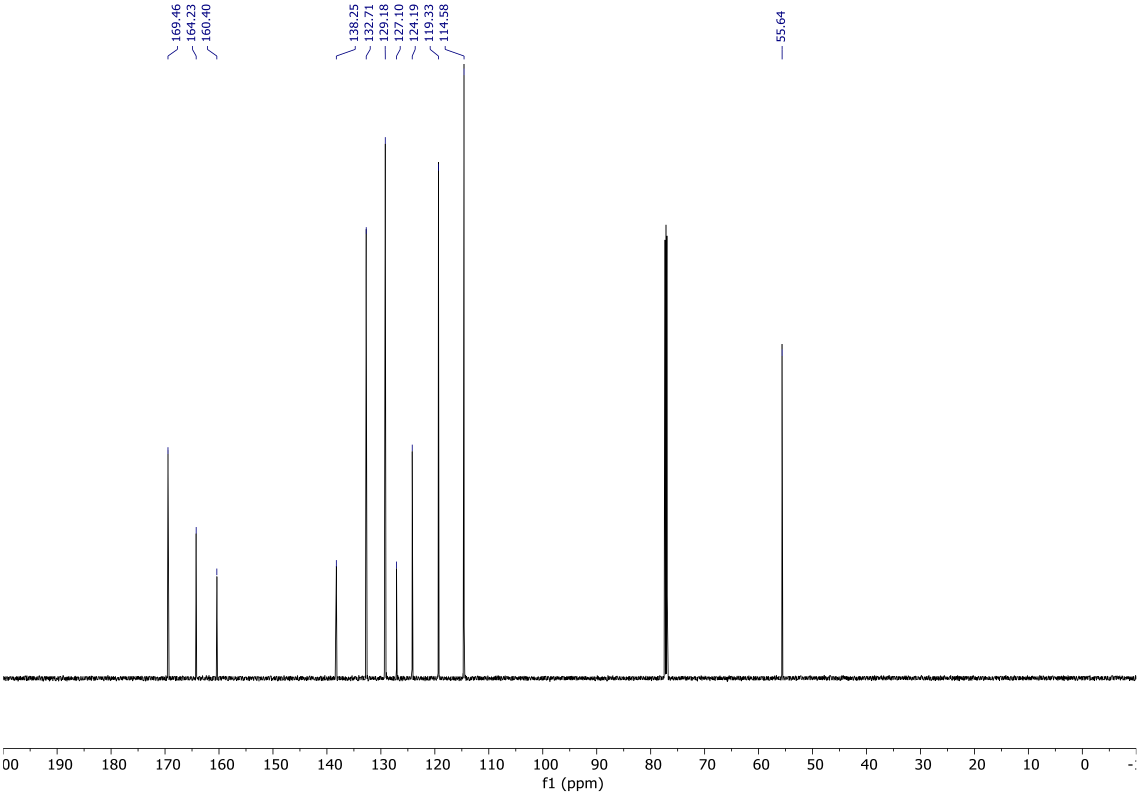

^{1}H-NMR (600 MHz, $CDCl_3$)

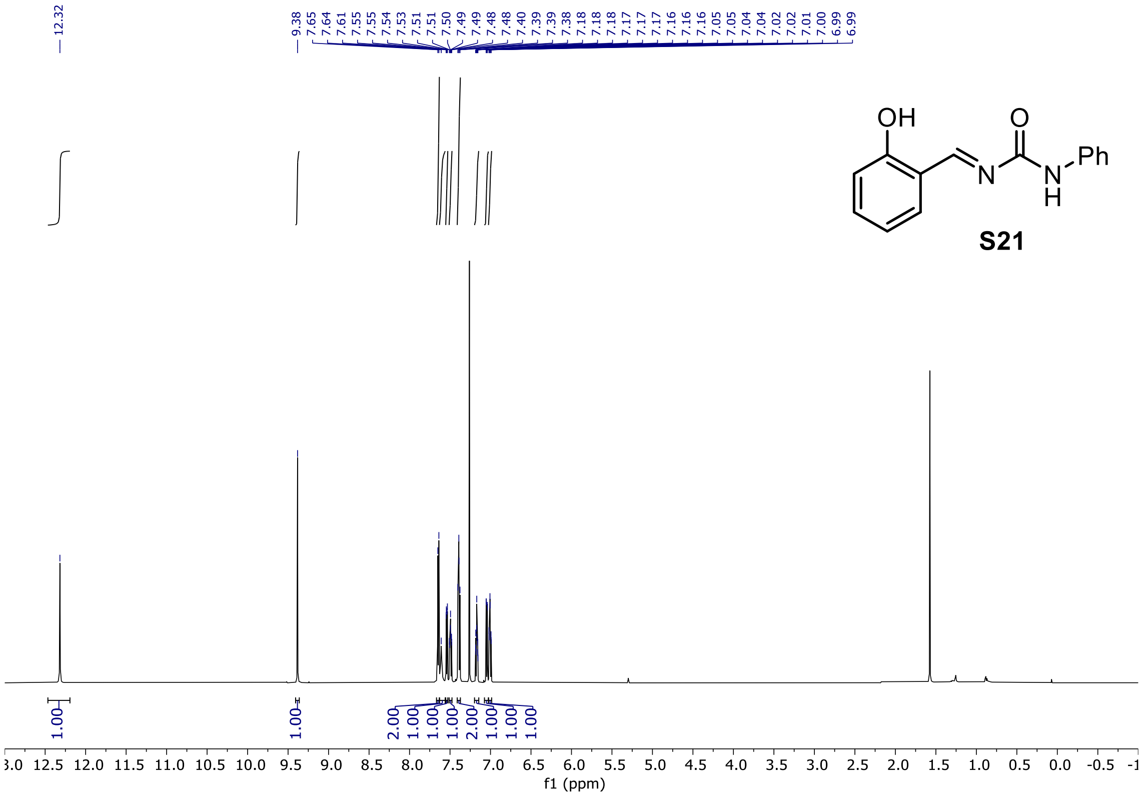

^{13}C-NMR (151 MHz, $CDCl_3$)

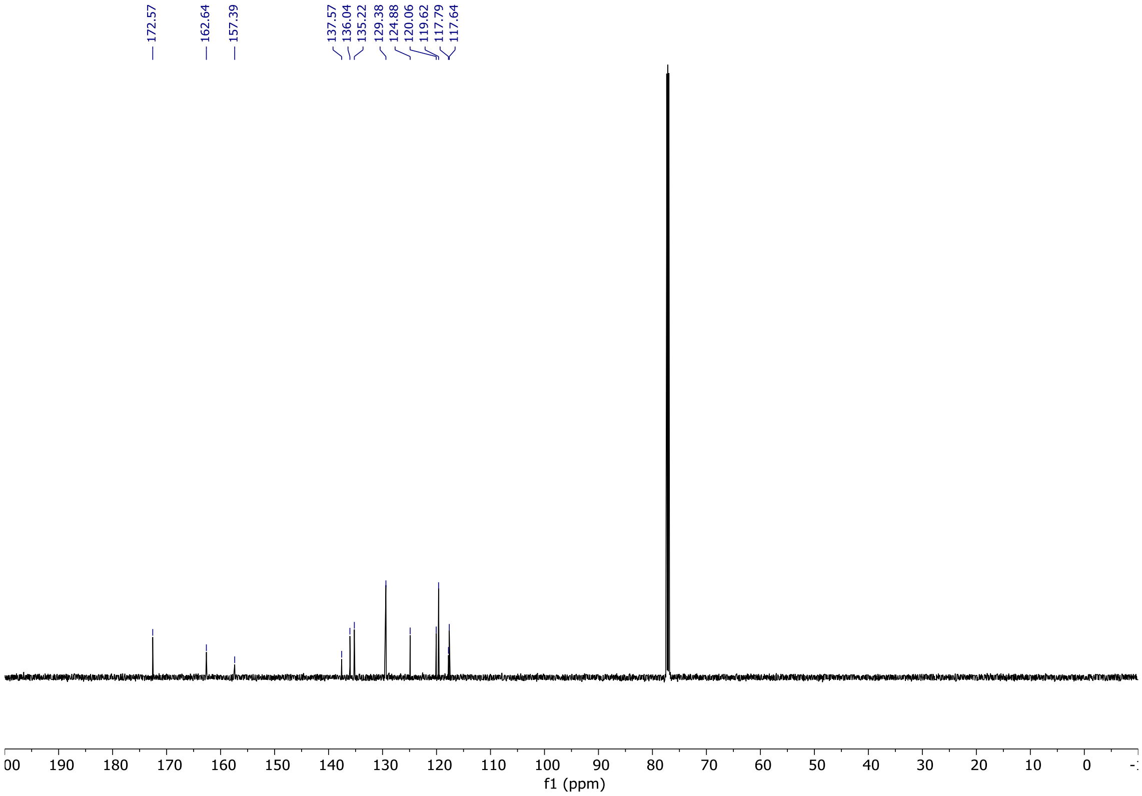

^{1}H-NMR (600 MHz, CDCl$_3$)

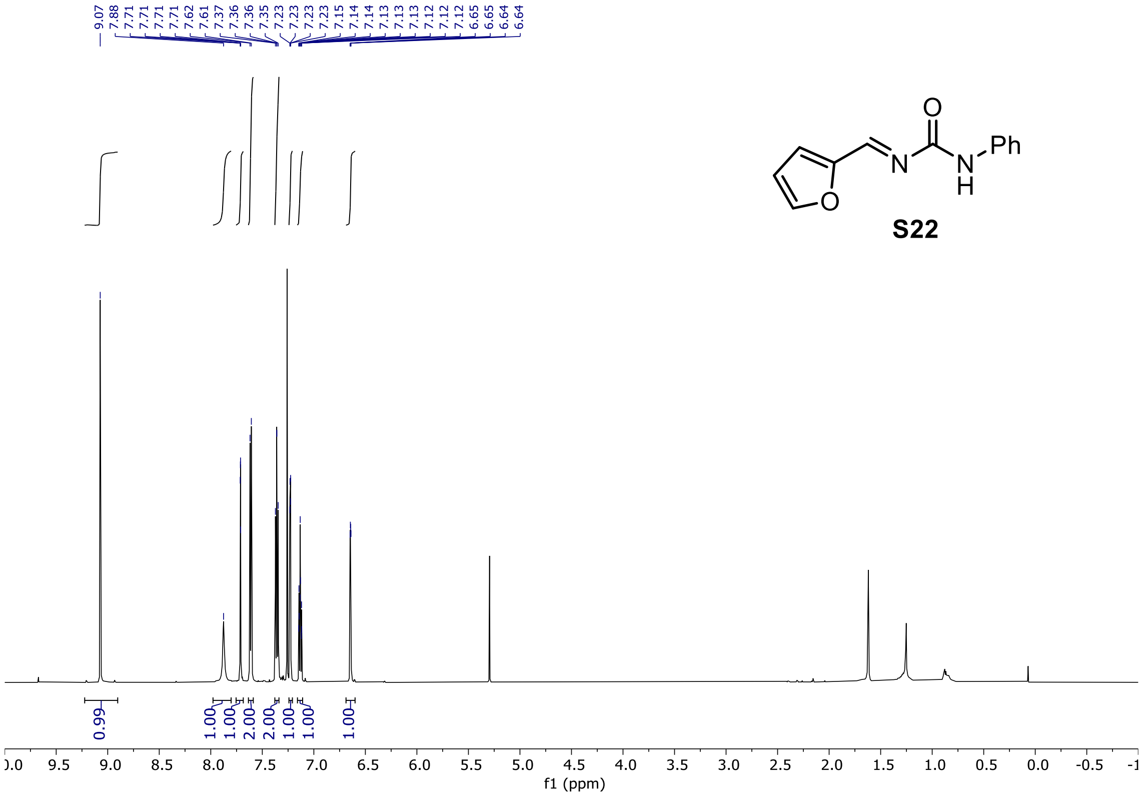

S22

^{13}C-NMR (151 MHz, CDCl$_3$)

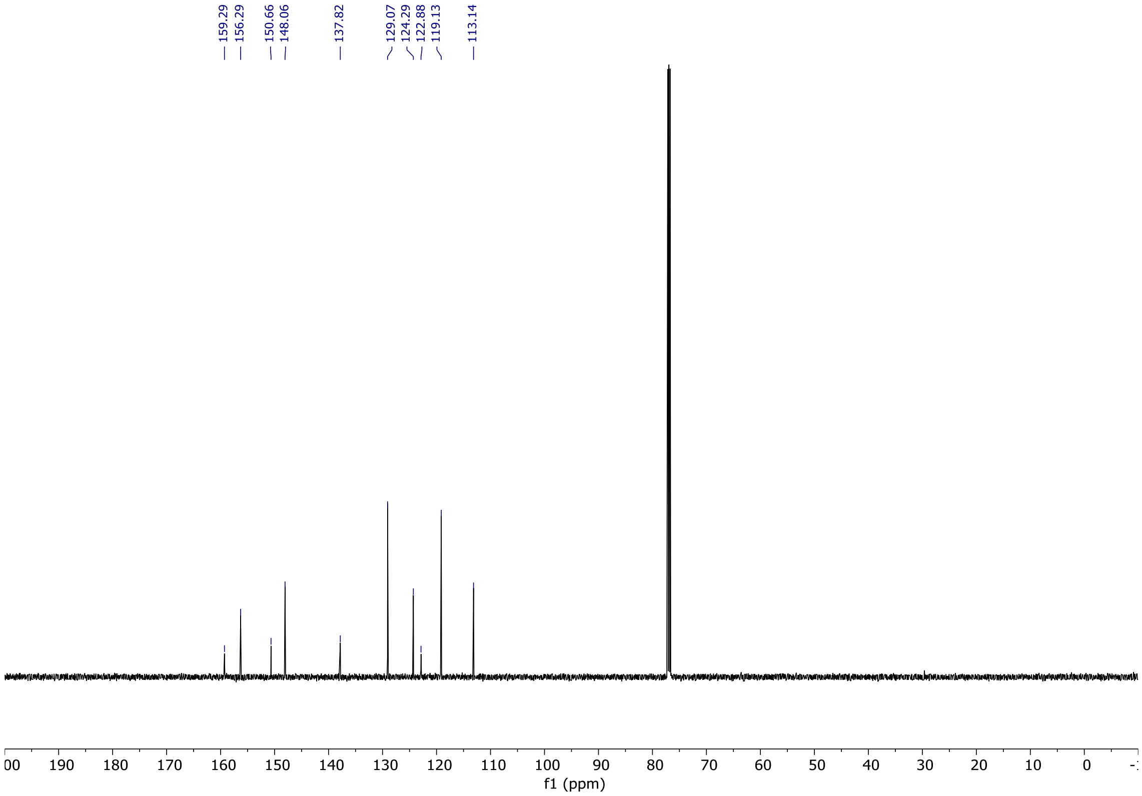

^{1}H-NMR (400 MHz, $CDCl_3$)

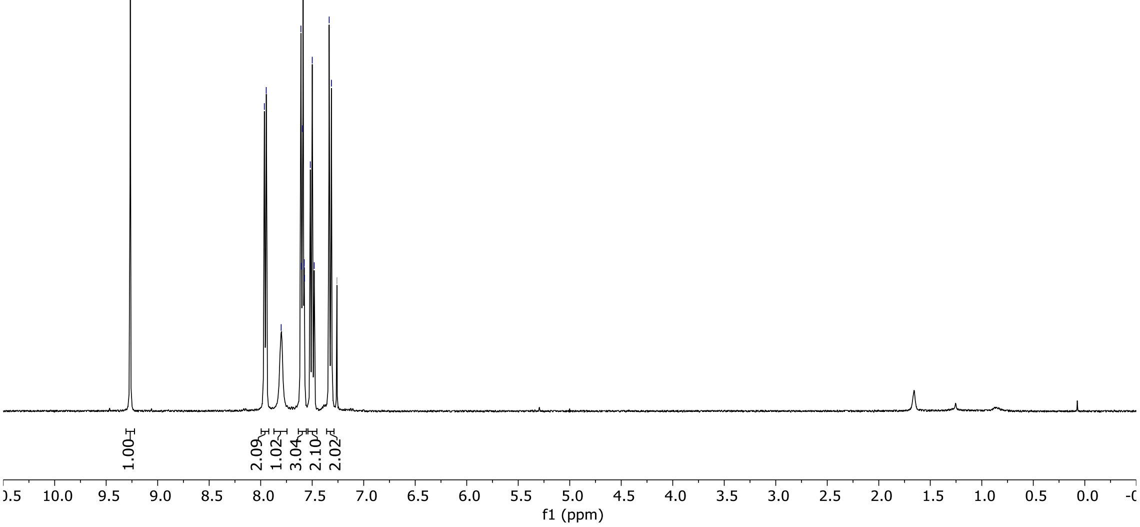

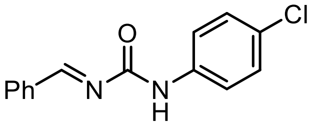

S23

^{13}C-NMR (151 MHz, $CDCl_3$)

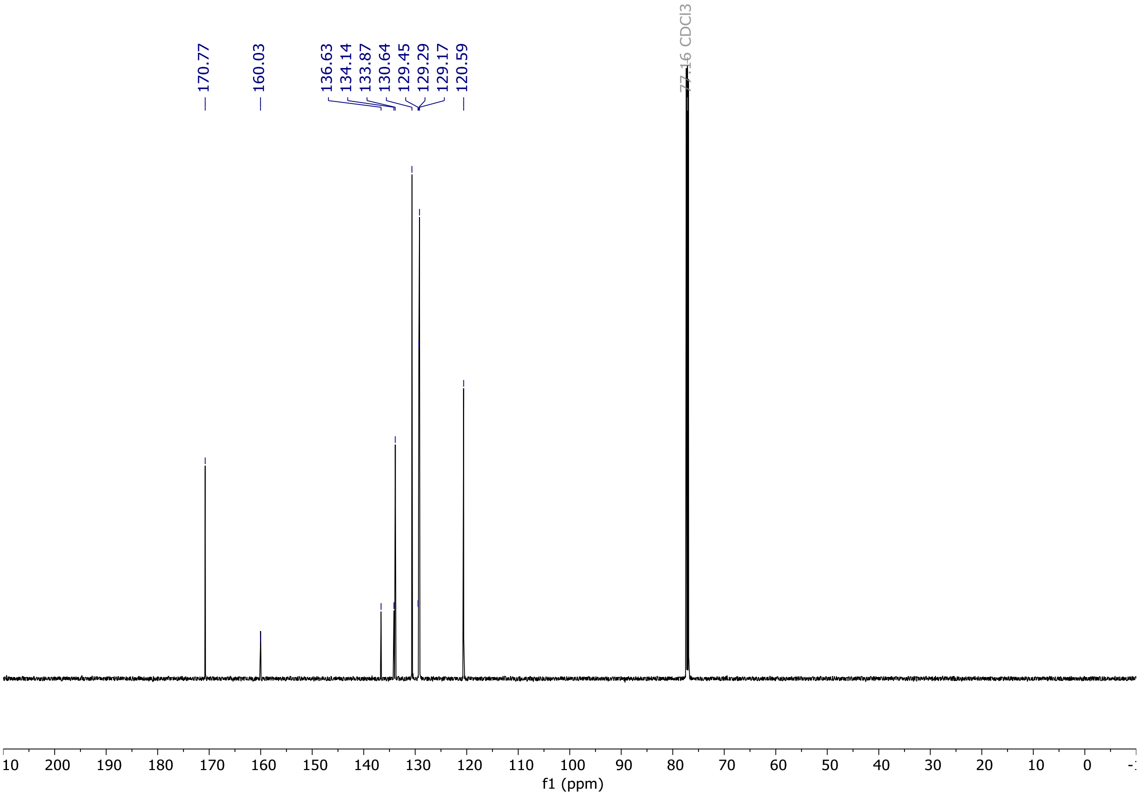

^{1}H-NMR (400 MHz, $CDCl_3$)

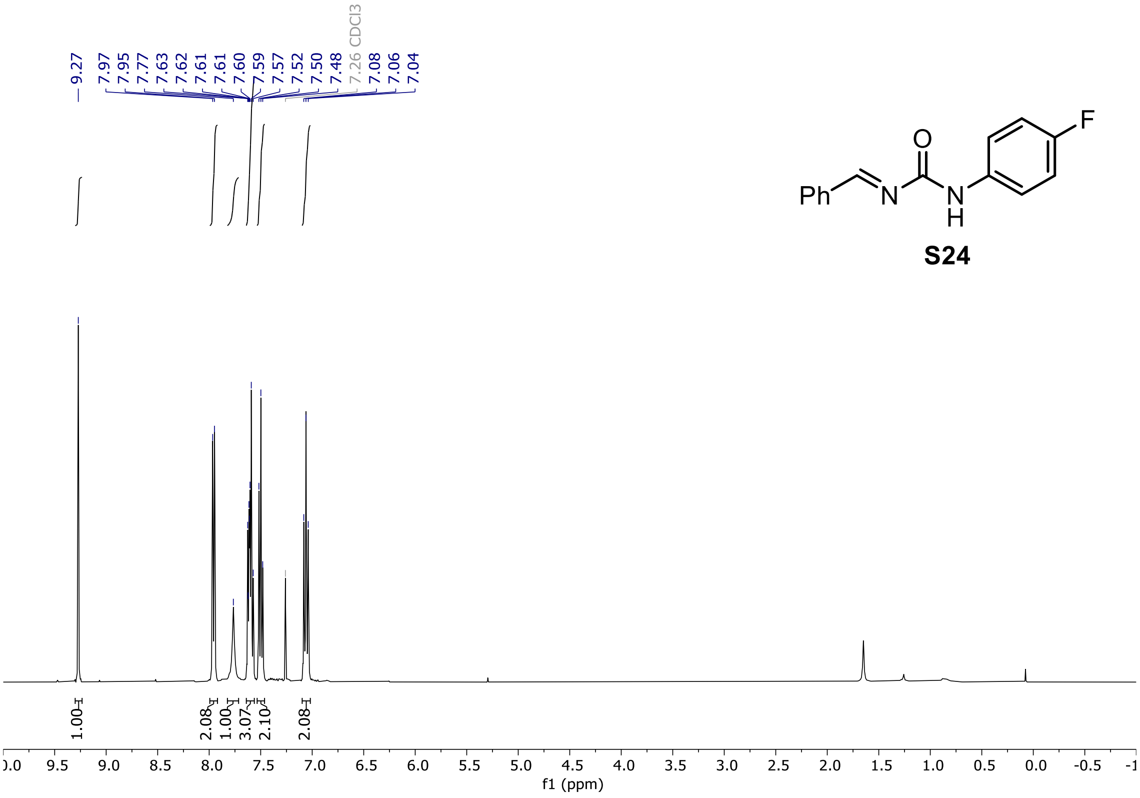

S24

^{13}C-NMR (101 MHz, $CDCl_3$)

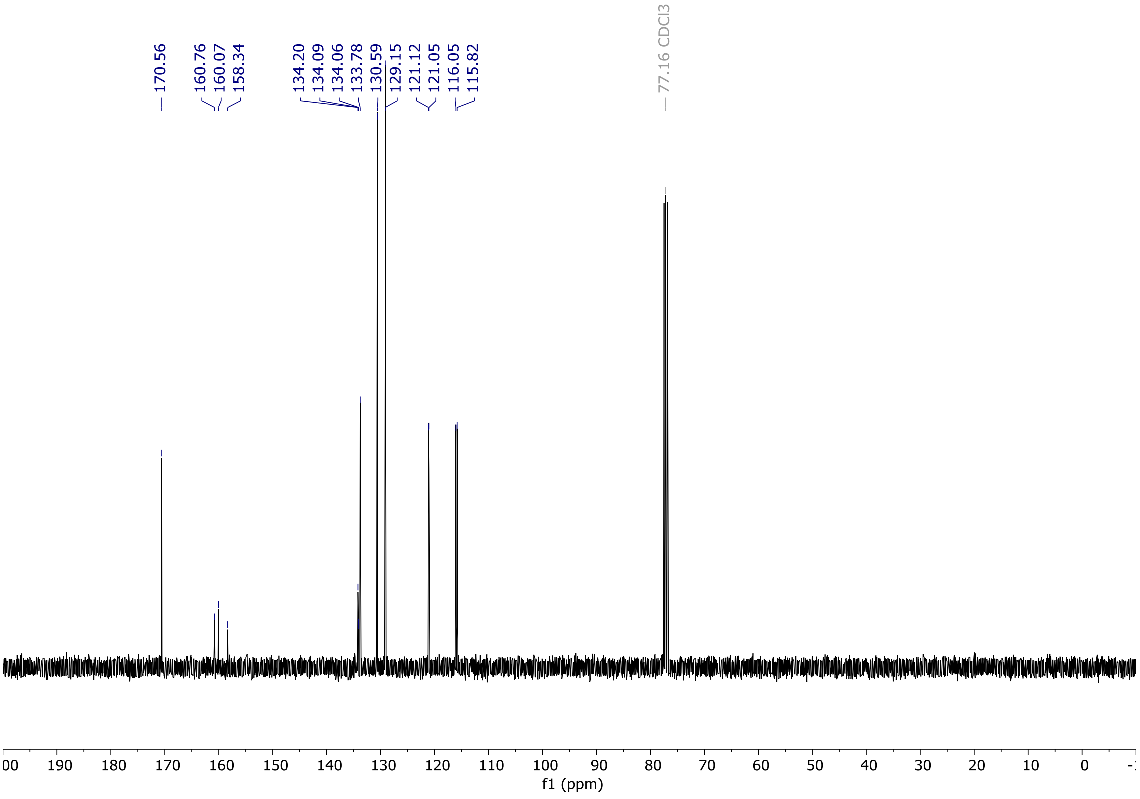

^{19}F-NMR (376 MHz, $CDCl_3$)

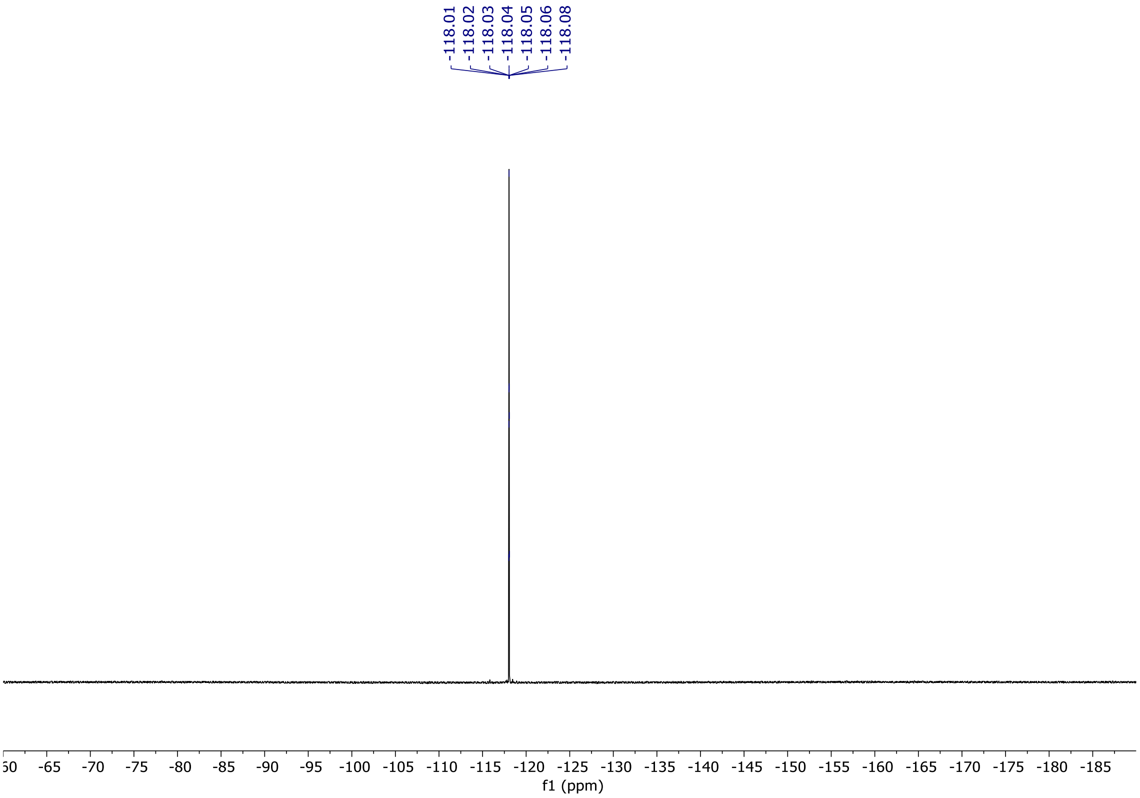

^{1}H-NMR (600 MHz, $CDCl_3$)

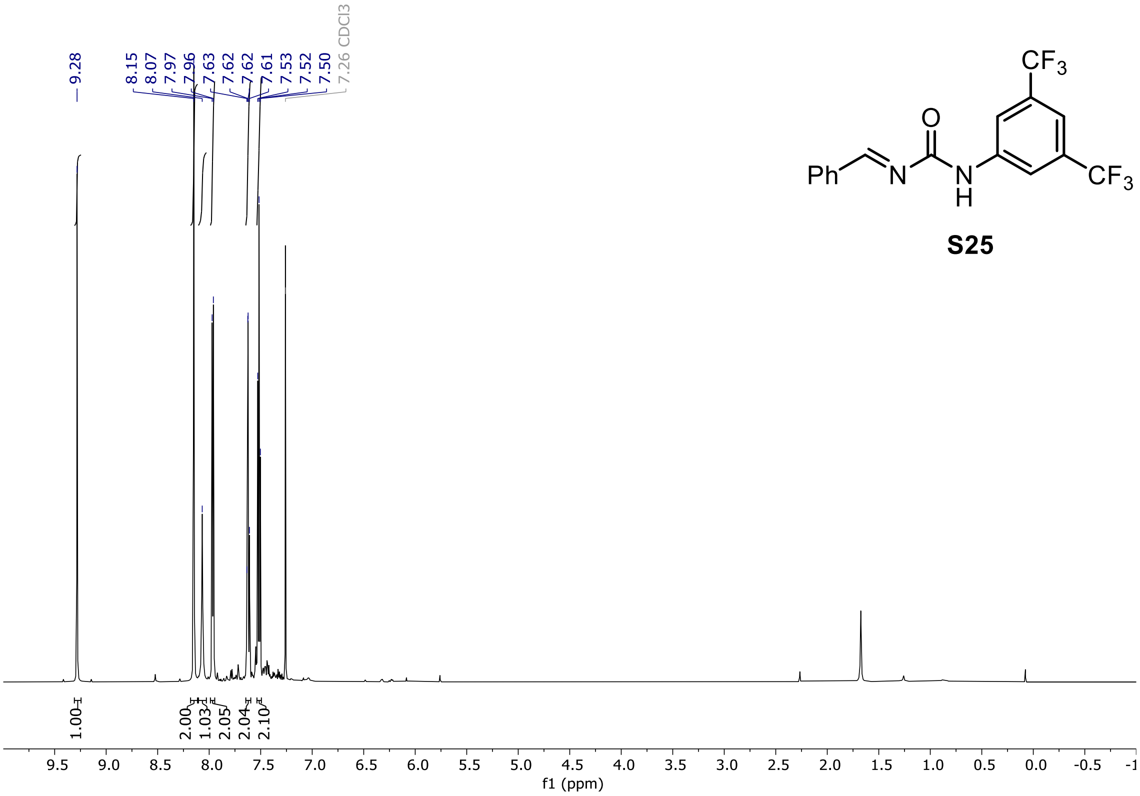

S25

^{13}C-NMR (151 MHz, $CDCl_3$)

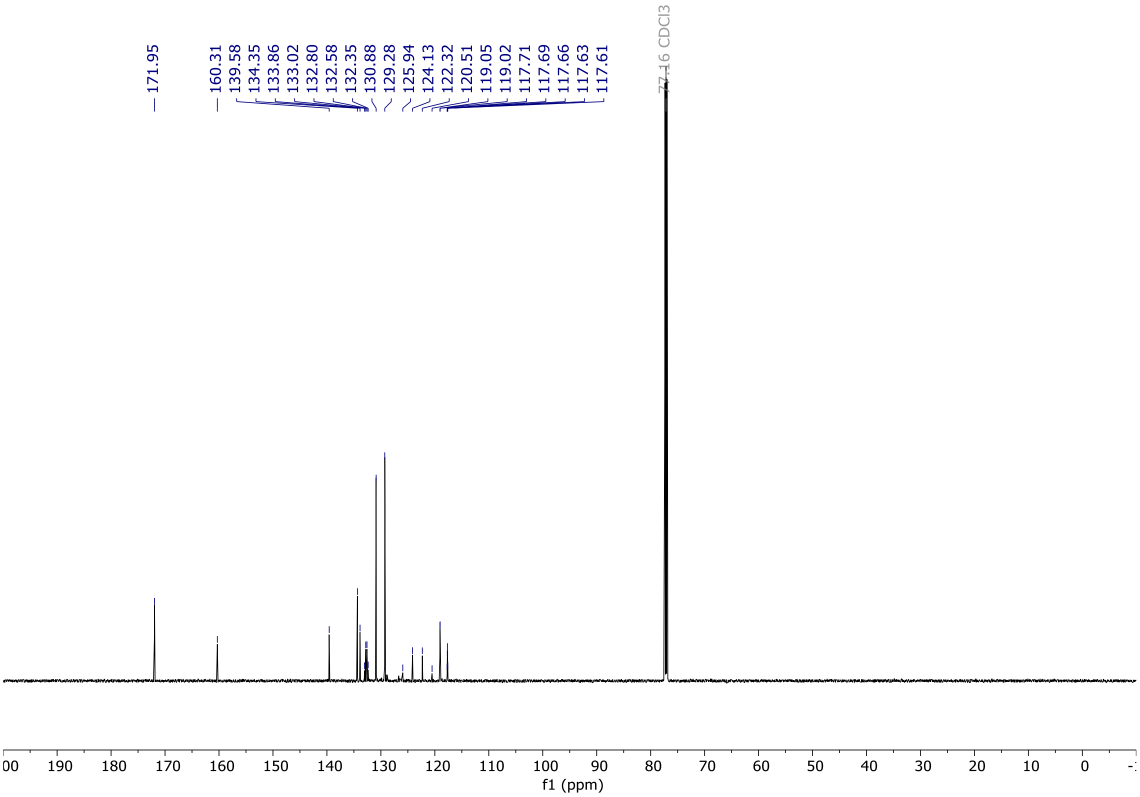

^{19}F-NMR (565 MHz, $CDCl_3$)

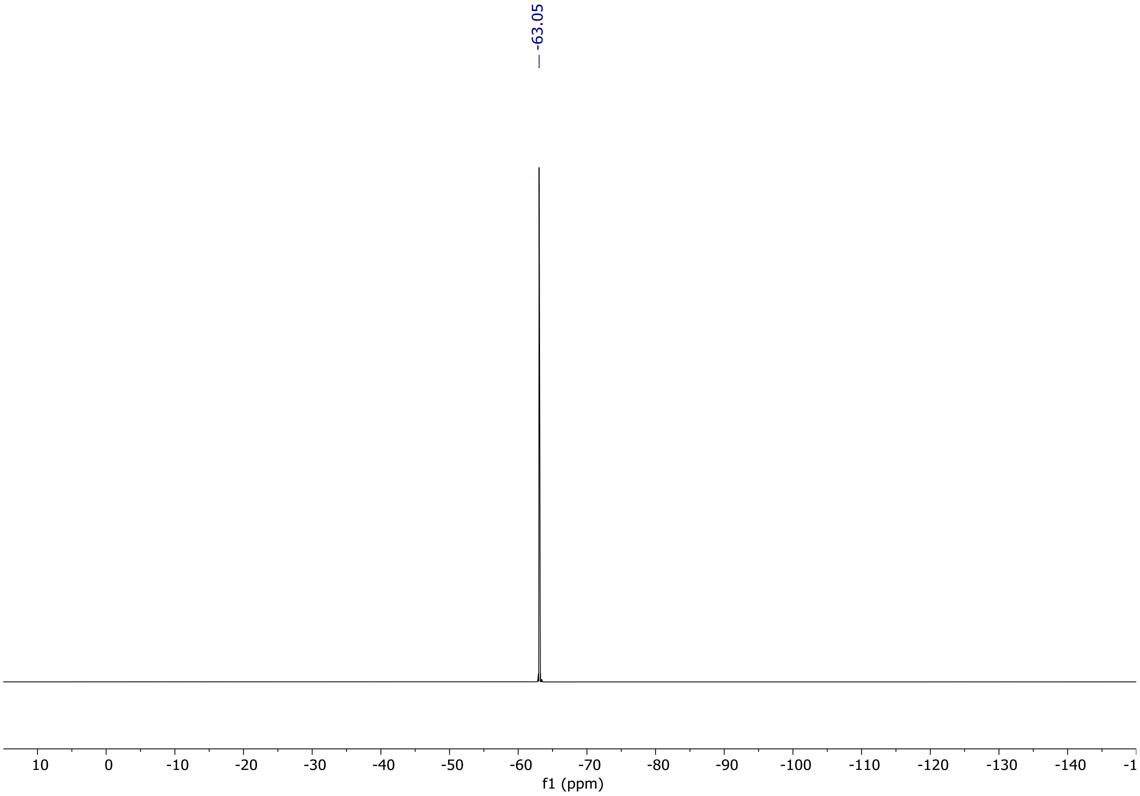

^{1}H-NMR (400 MHz, $CDCl_3$)

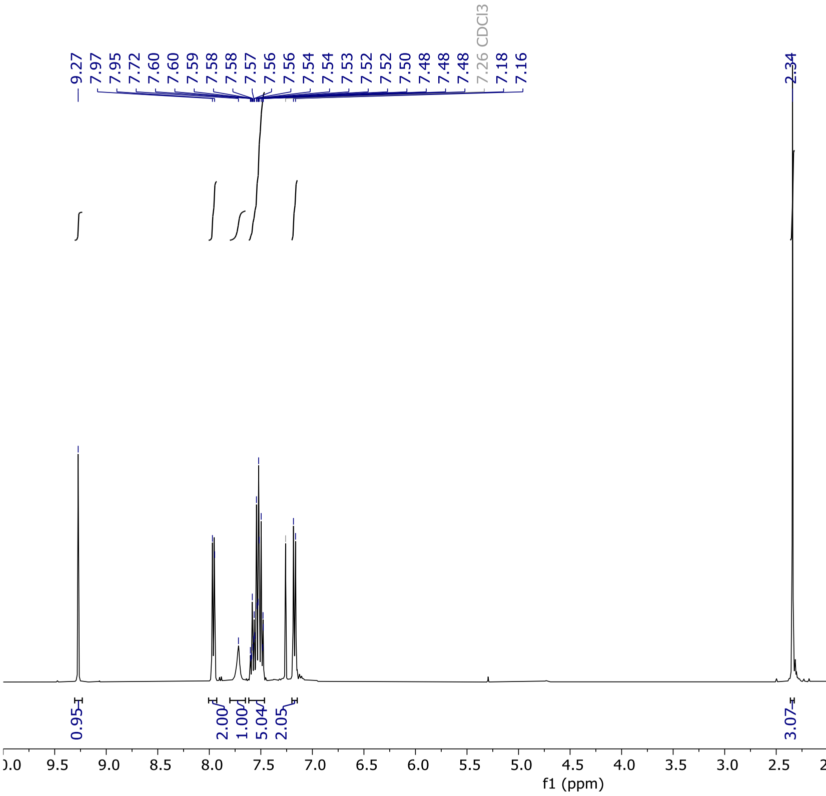

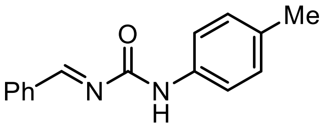

S26

^{13}C-NMR (101 MHz, $CDCl_3$)

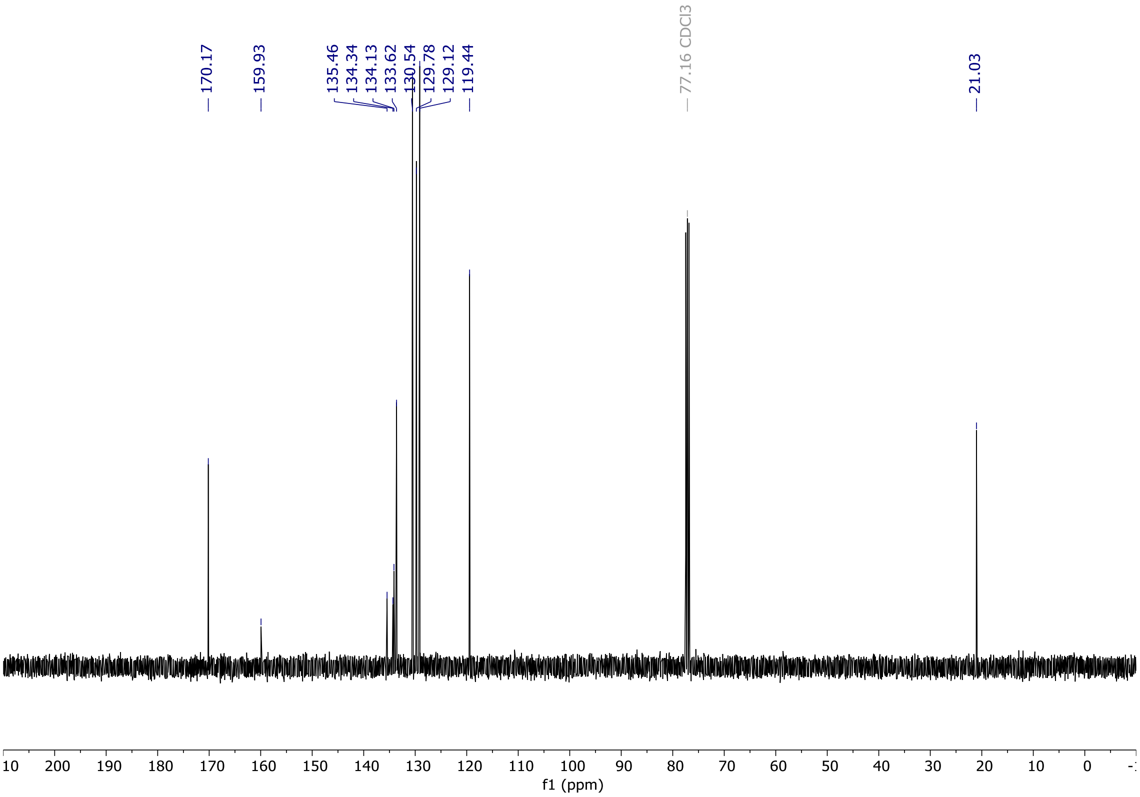

^{1}H-NMR (600 MHz, $CDCl_3$)

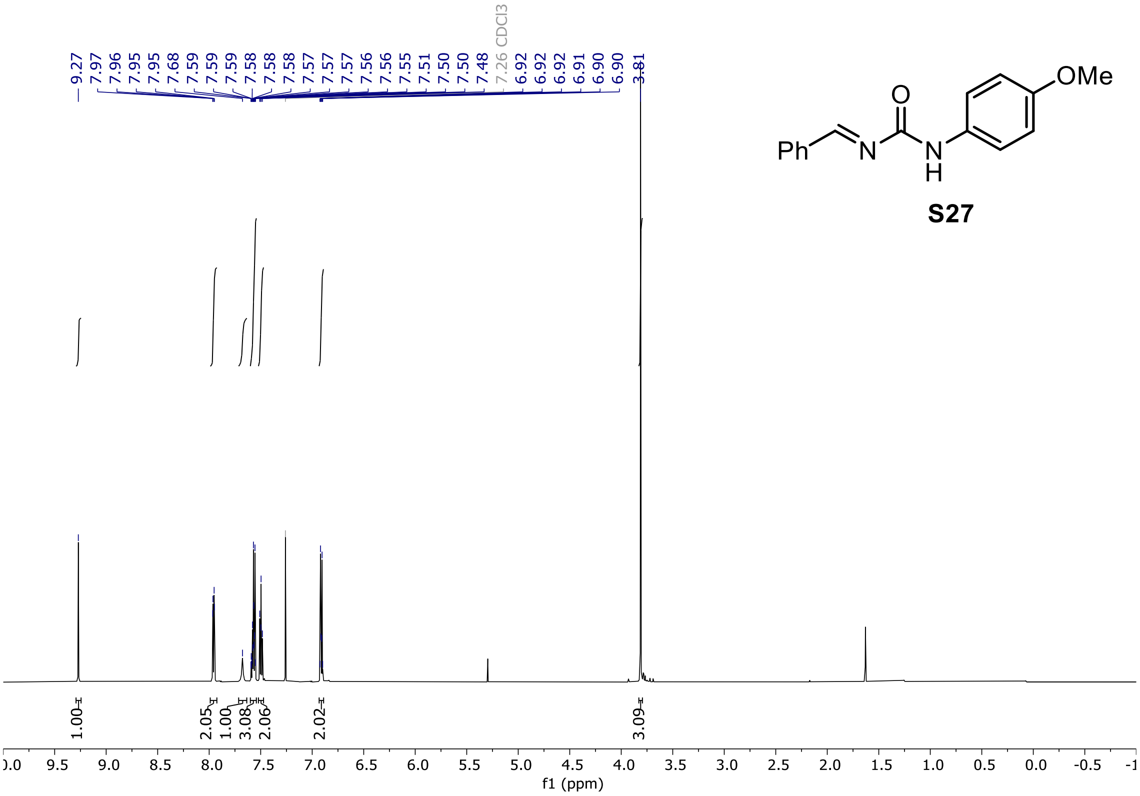

S27

^{13}C-NMR (151 MHz, $CDCl_3$)

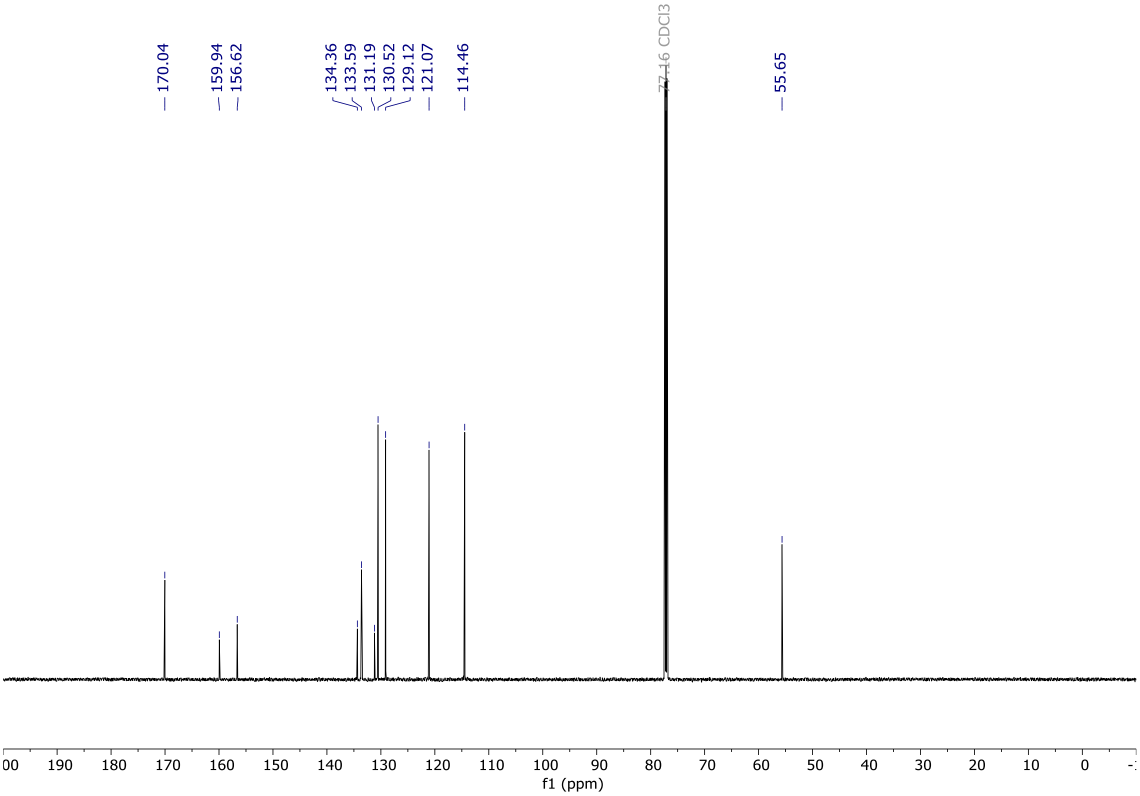

^{1}H-NMR (400 MHz, CDCl$_3$)

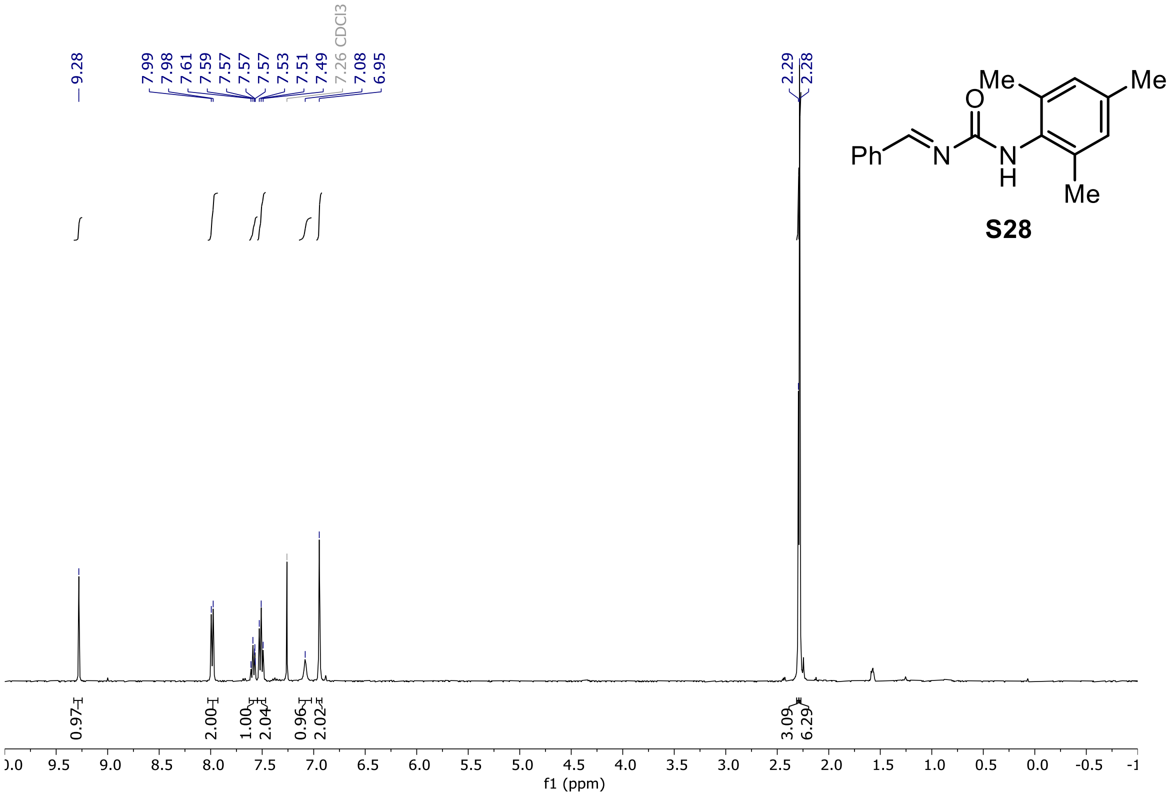

S28

^{13}C-NMR (151 MHz, CDCl$_3$)

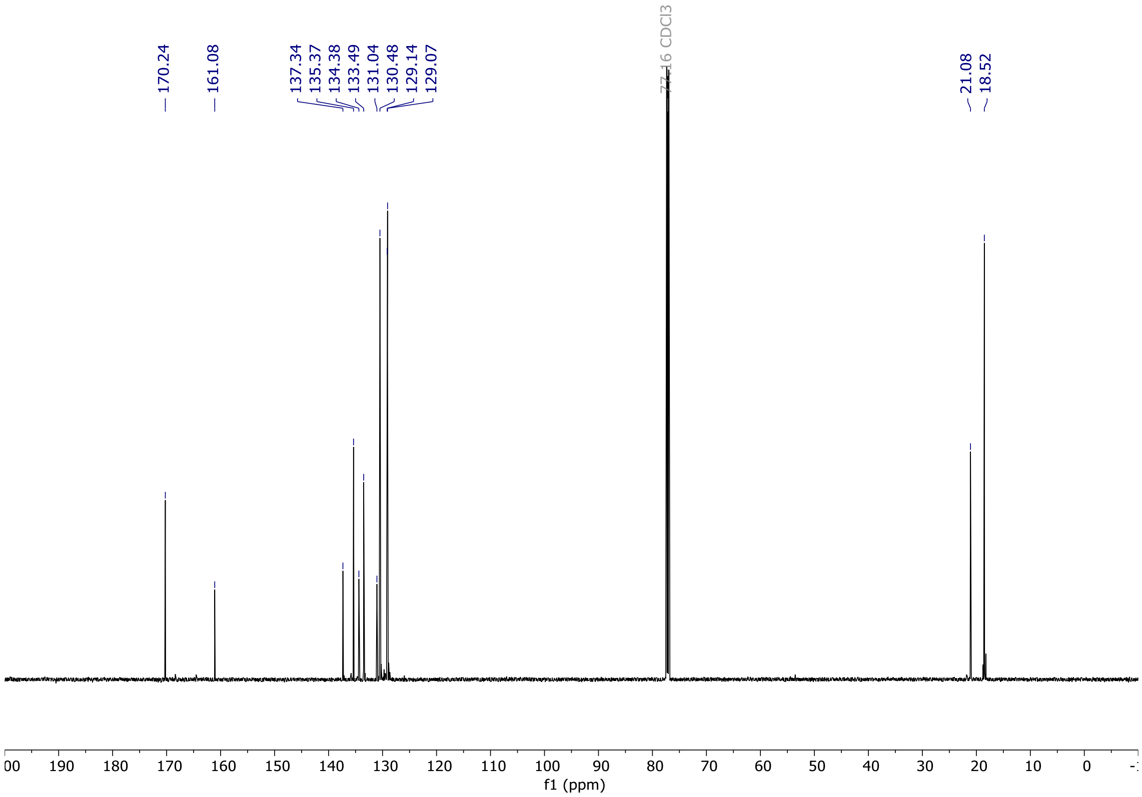

^{1}H-NMR (400 MHz, $CDCl_3$)

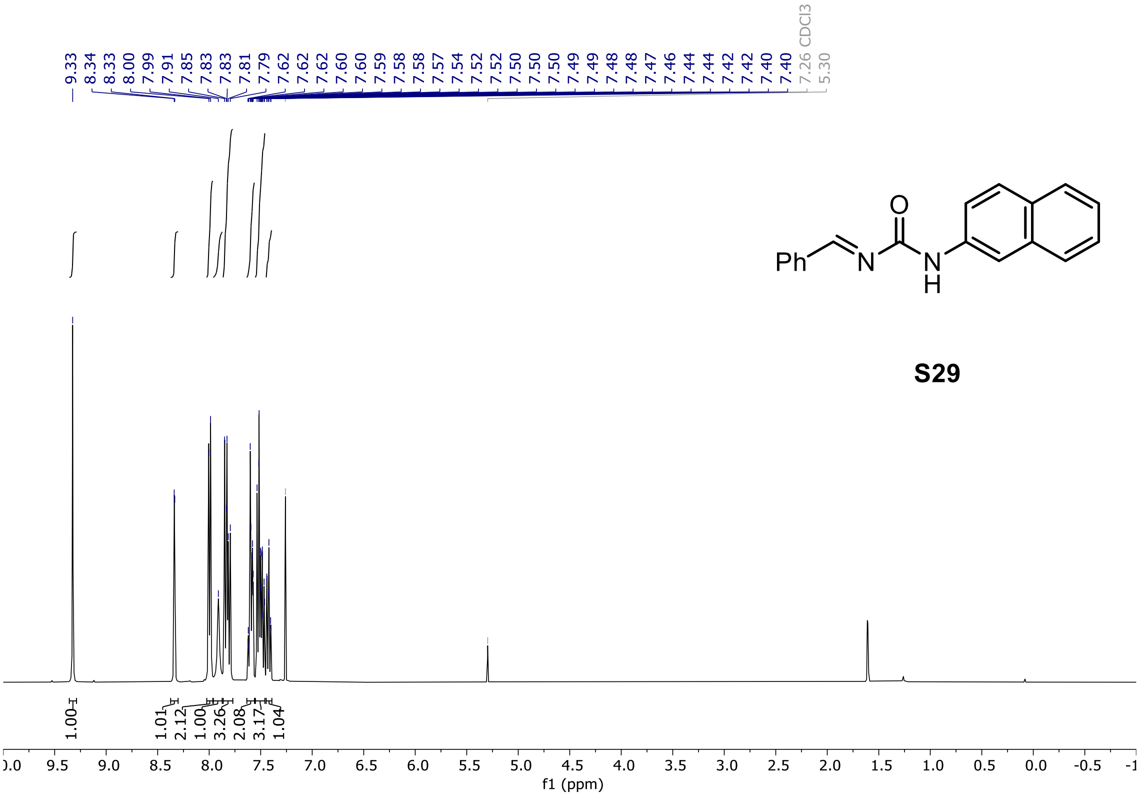

S29

^{13}C-NMR (151 MHz, $CDCl_3$)

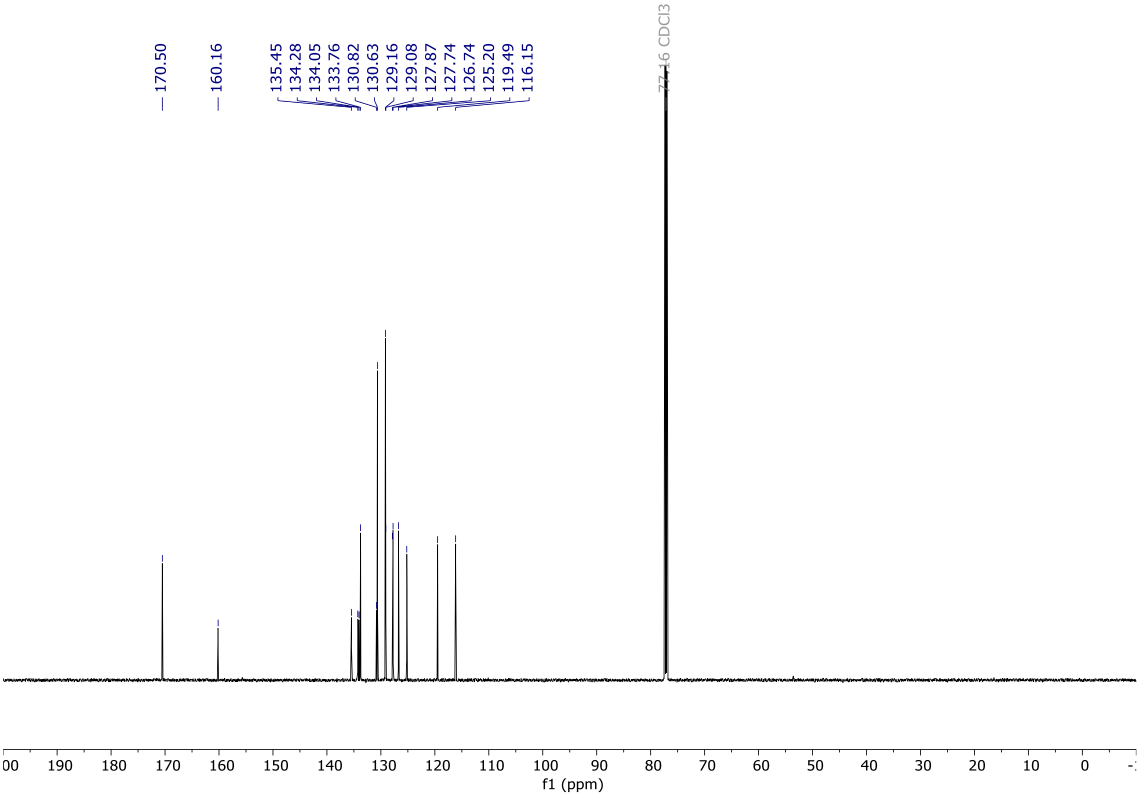

^{1}H-NMR (400 MHz, DMSO-d_6)

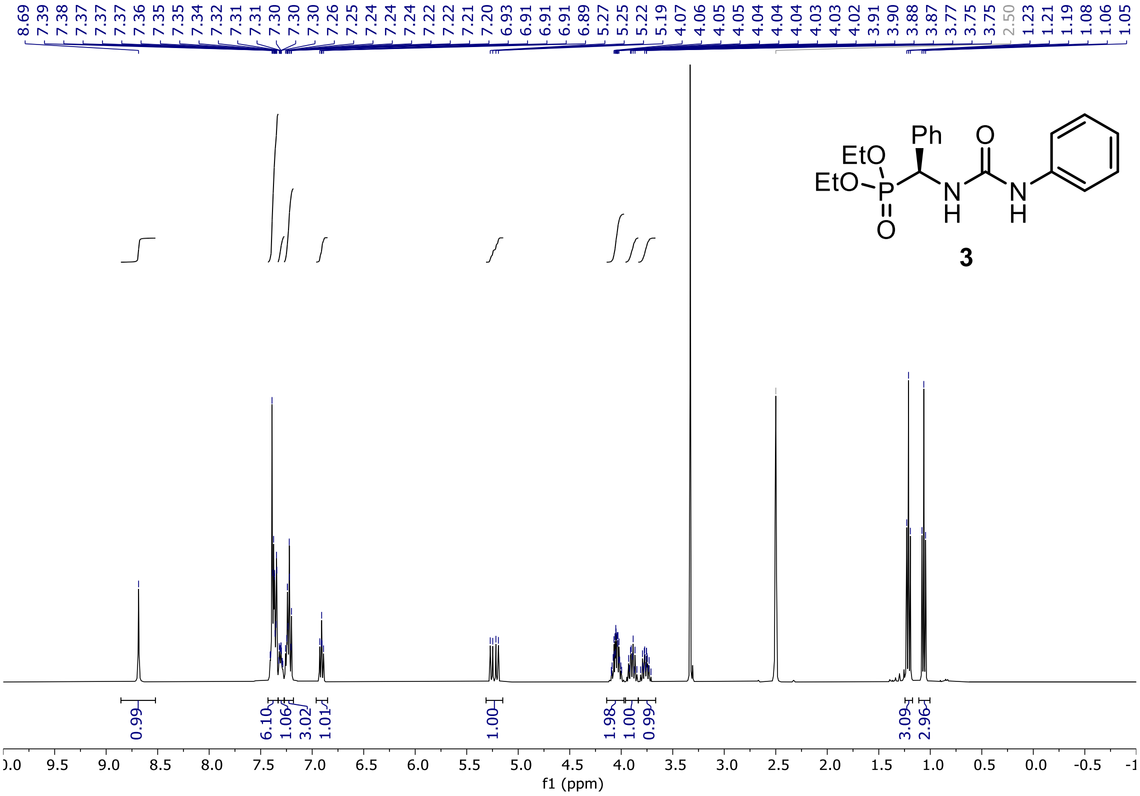

^{13}C-NMR (151 MHz, DMSO-d_6)

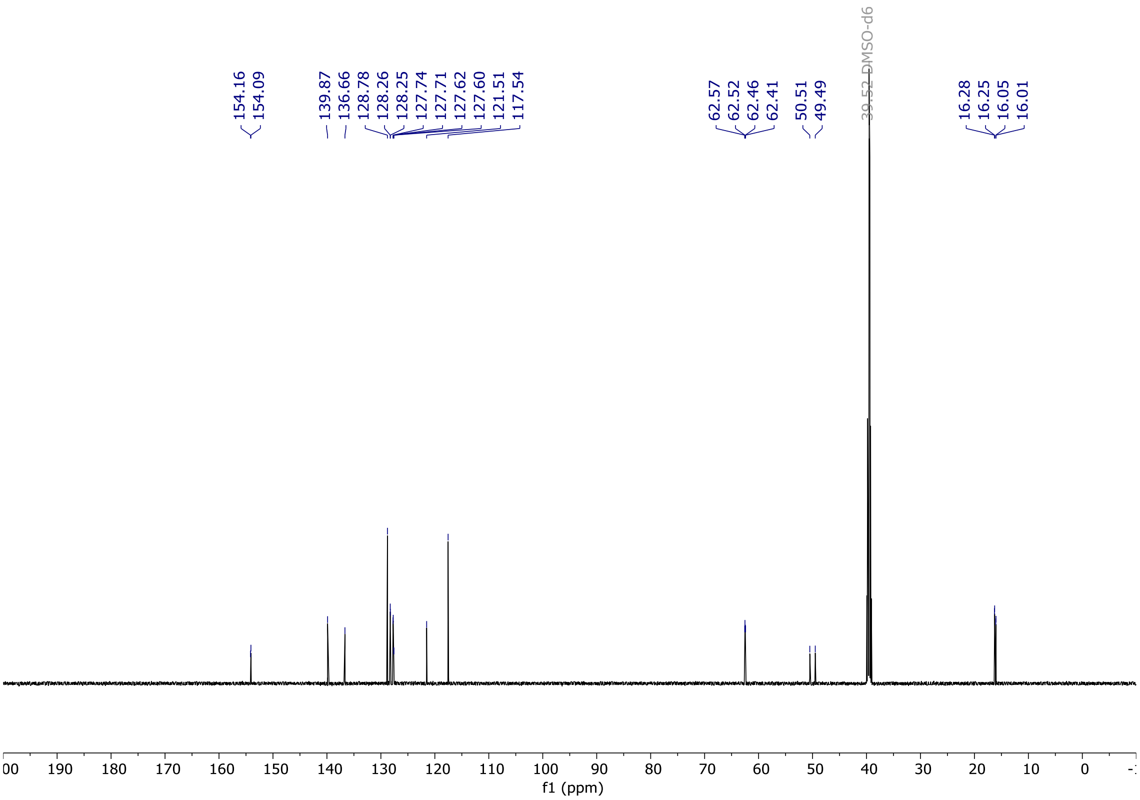

^{31}P-NMR (162 MHz, DMSO-d_6)

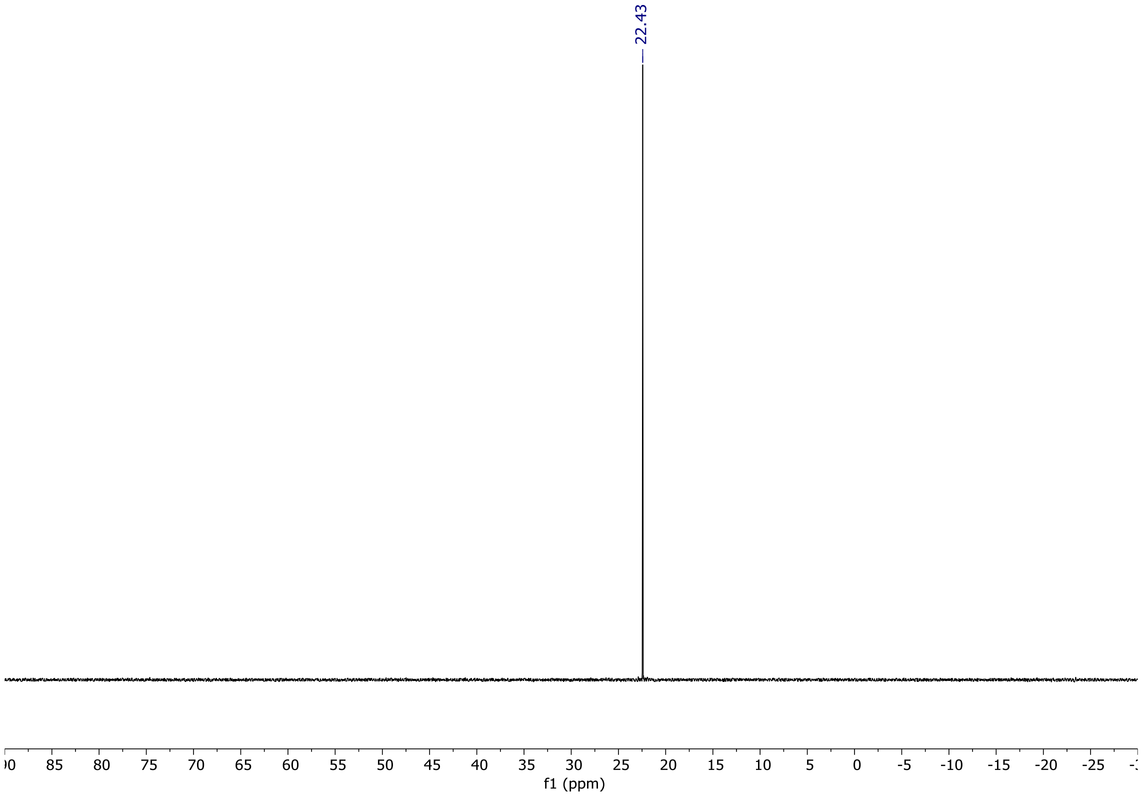

^{1}H-NMR (600 MHz, DMSO- *d*6)

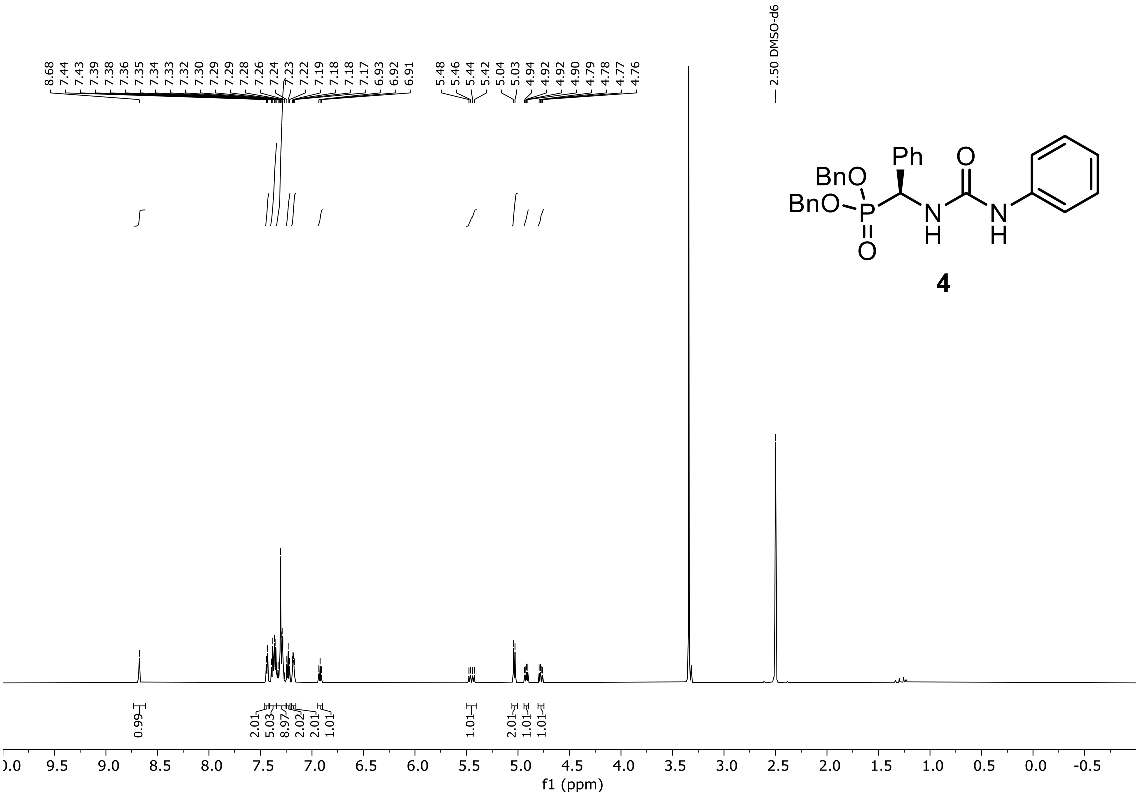

^{13}C-NMR (151 MHz, DMSO- *d*6)

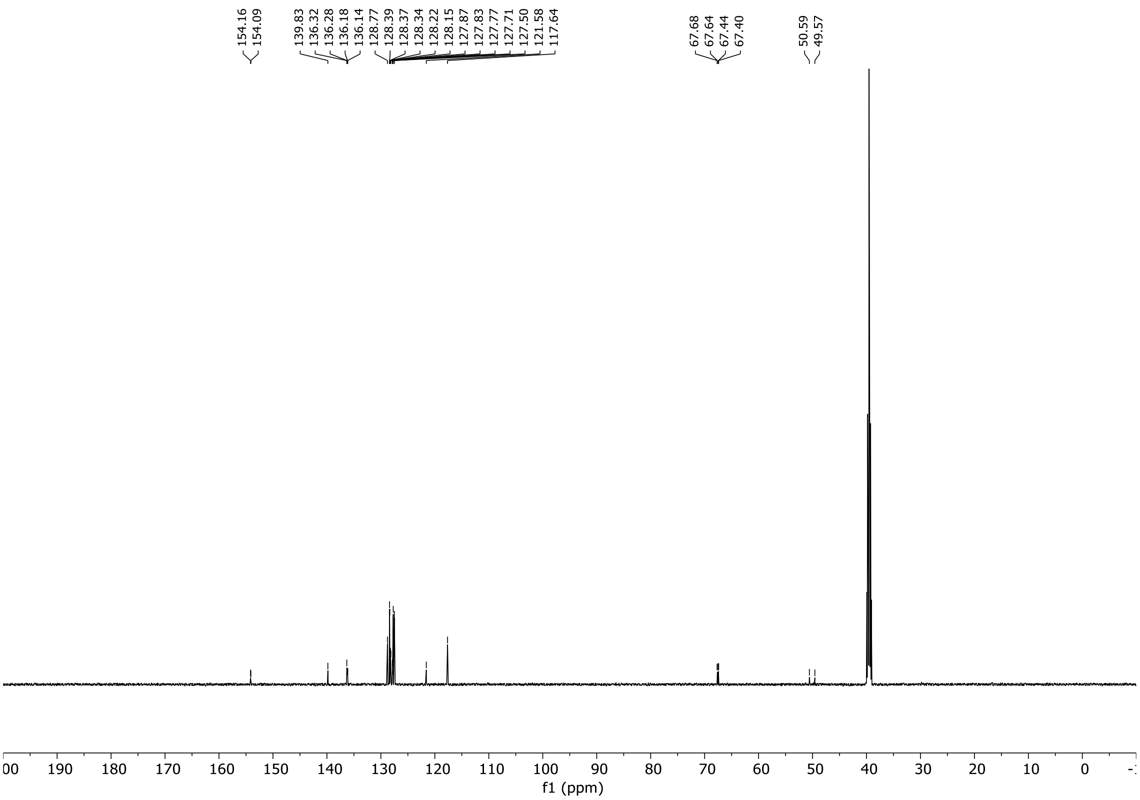

^{31}P-NMR (162 MHz, DMSO-*d*$_6$)

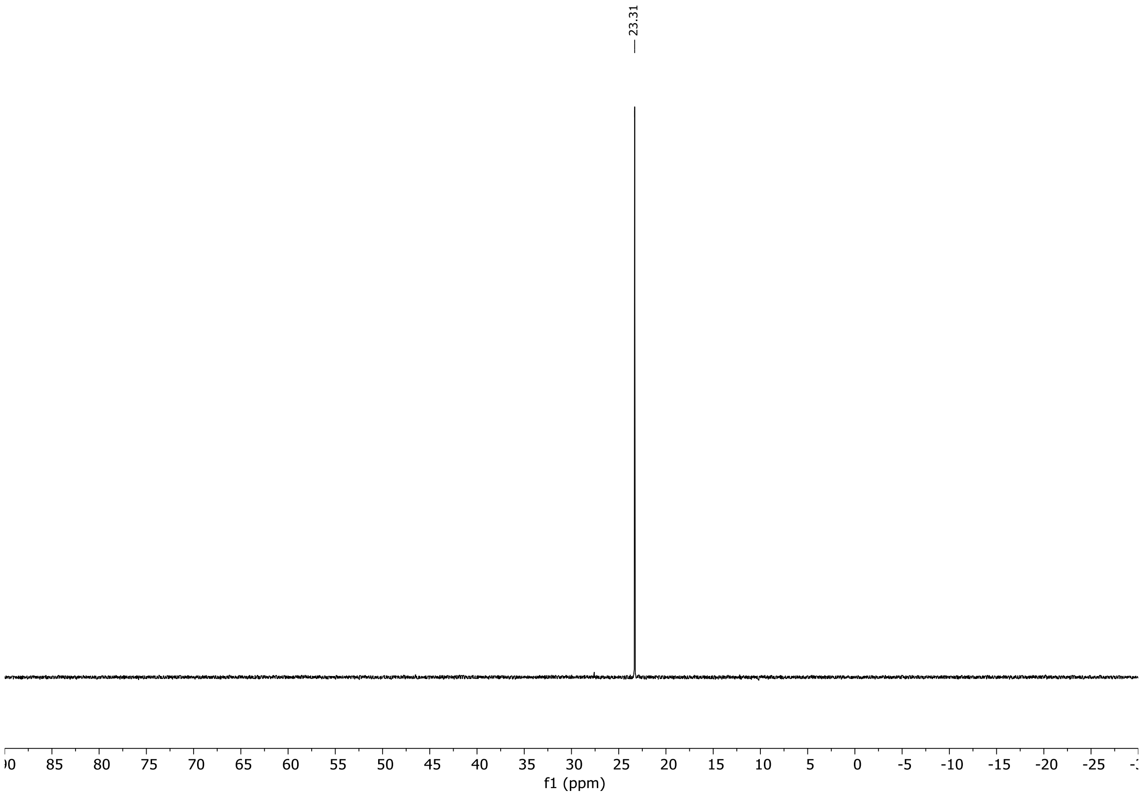

^{1}H-NMR (600 MHz, DMSO- d_6)

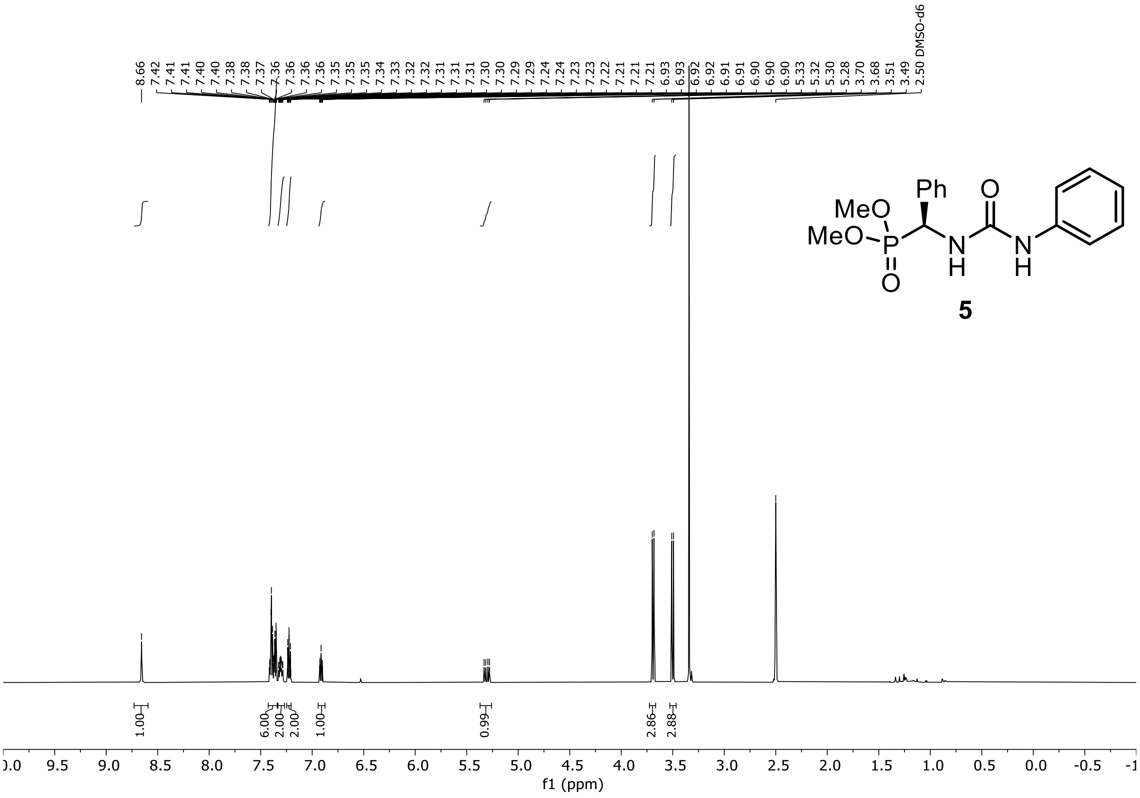

^{13}C-NMR (151 MHz, DMSO- d_6)

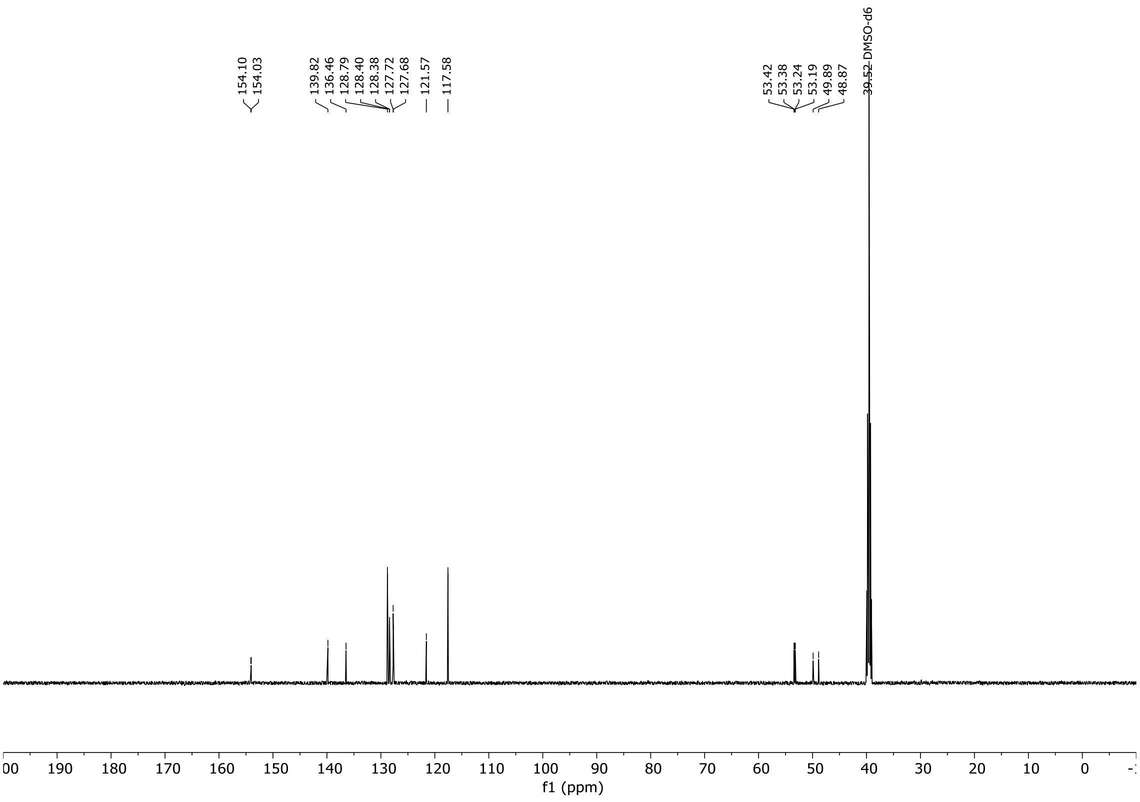

^{31}P-NMR (162 MHz, DMSO-d_6)

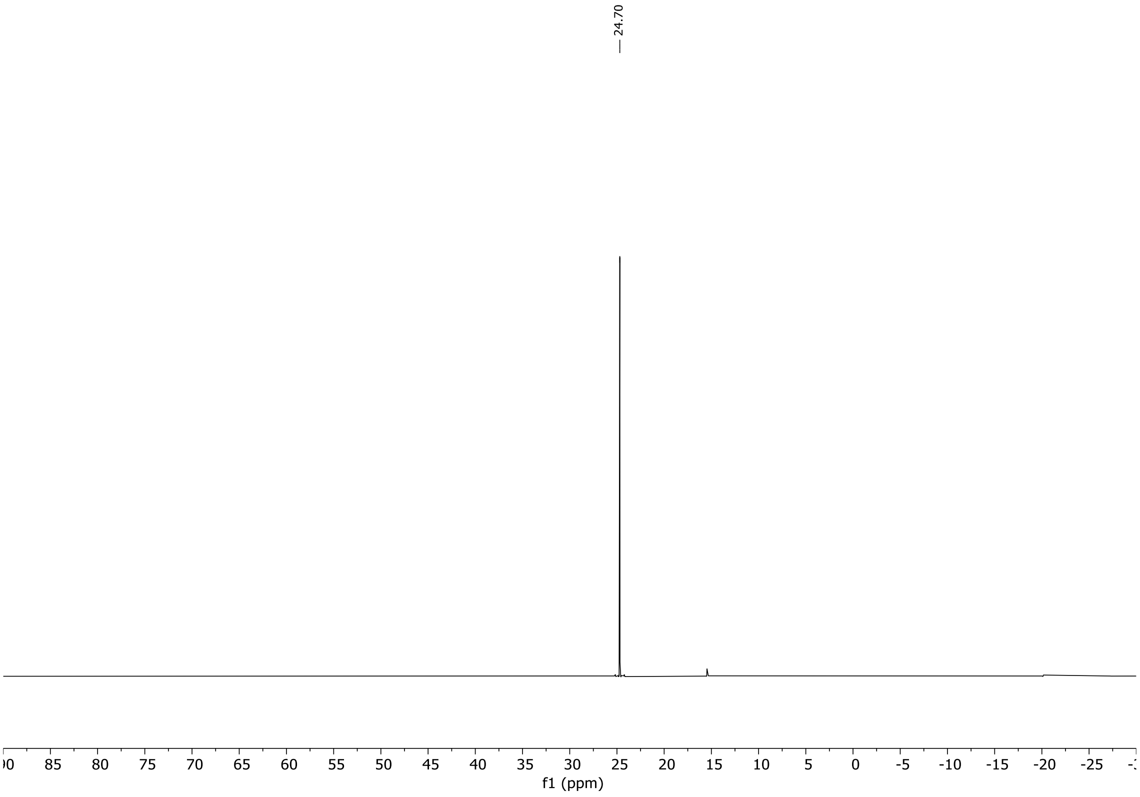

^{1}H-NMR (600 MHz, MeOD-d_4)

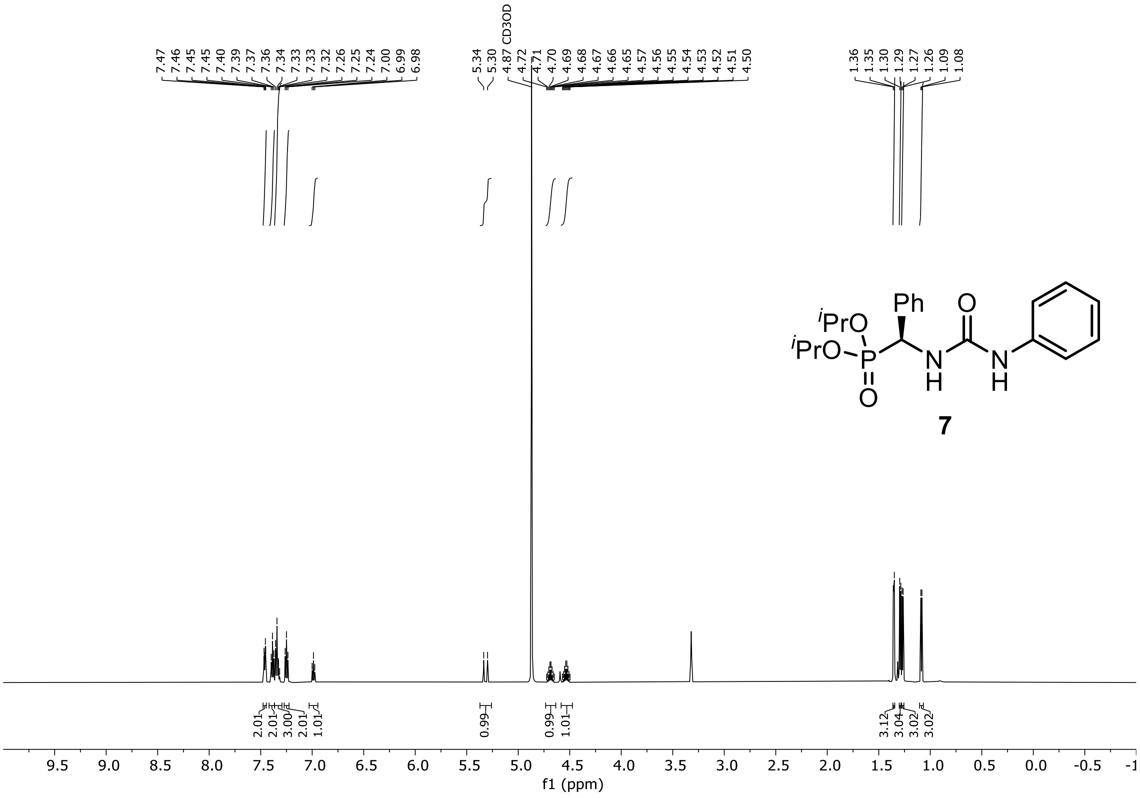

^{13}C-NMR (151 MHz, MeOD-d_4)

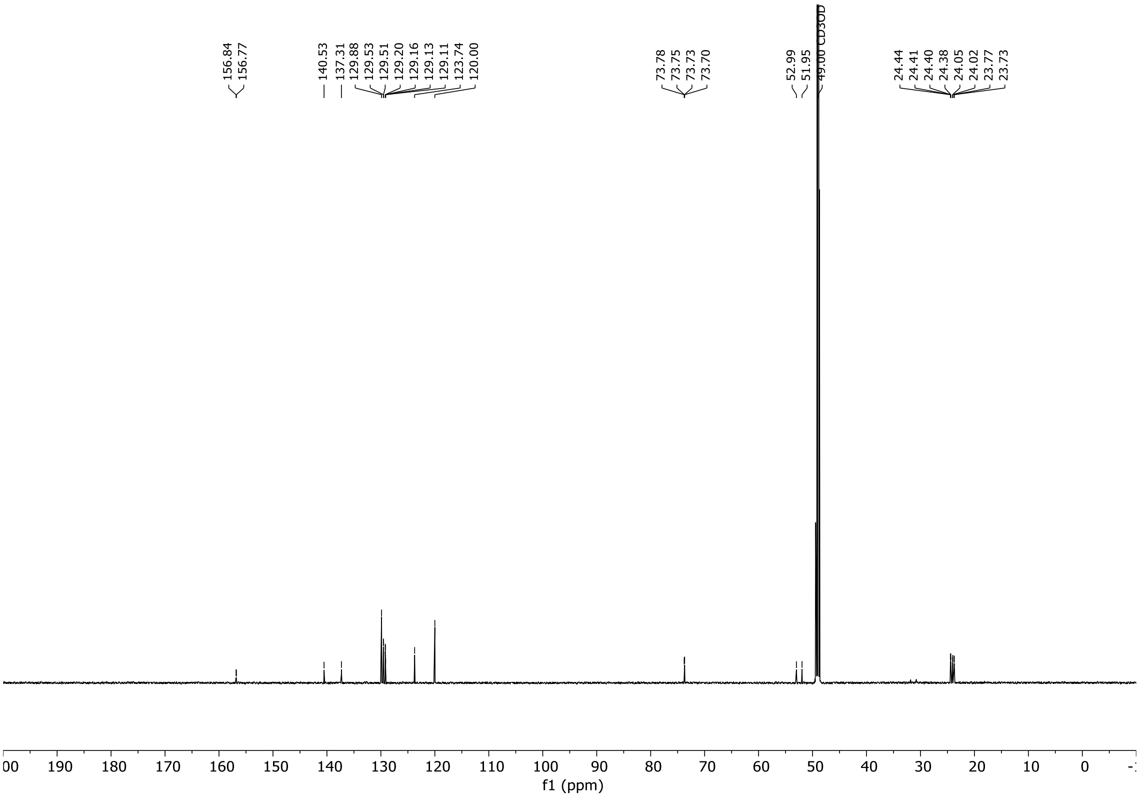

^{31}P-NMR (162 MHz, MeOD-*d*$_4$)

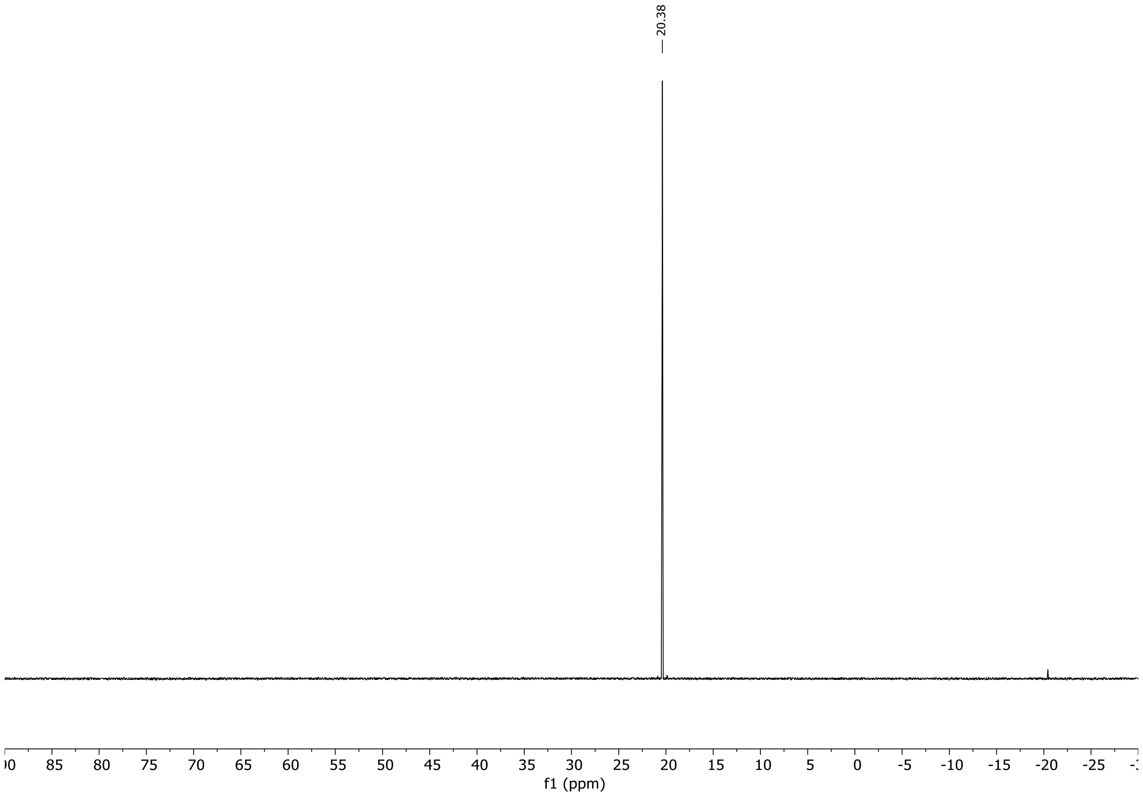

^{1}H-NMR (600 MHz, DMSO-d_6)

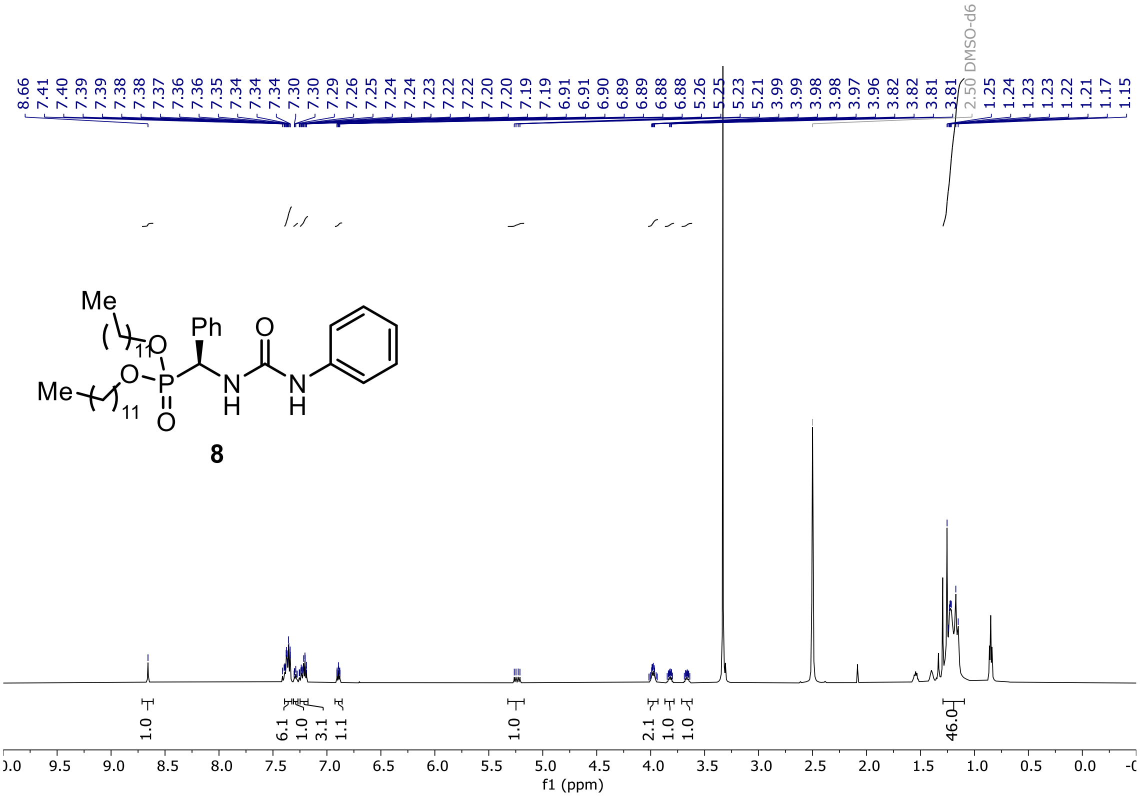

8

^{13}C-NMR (151 MHz, DMSO-d_6)

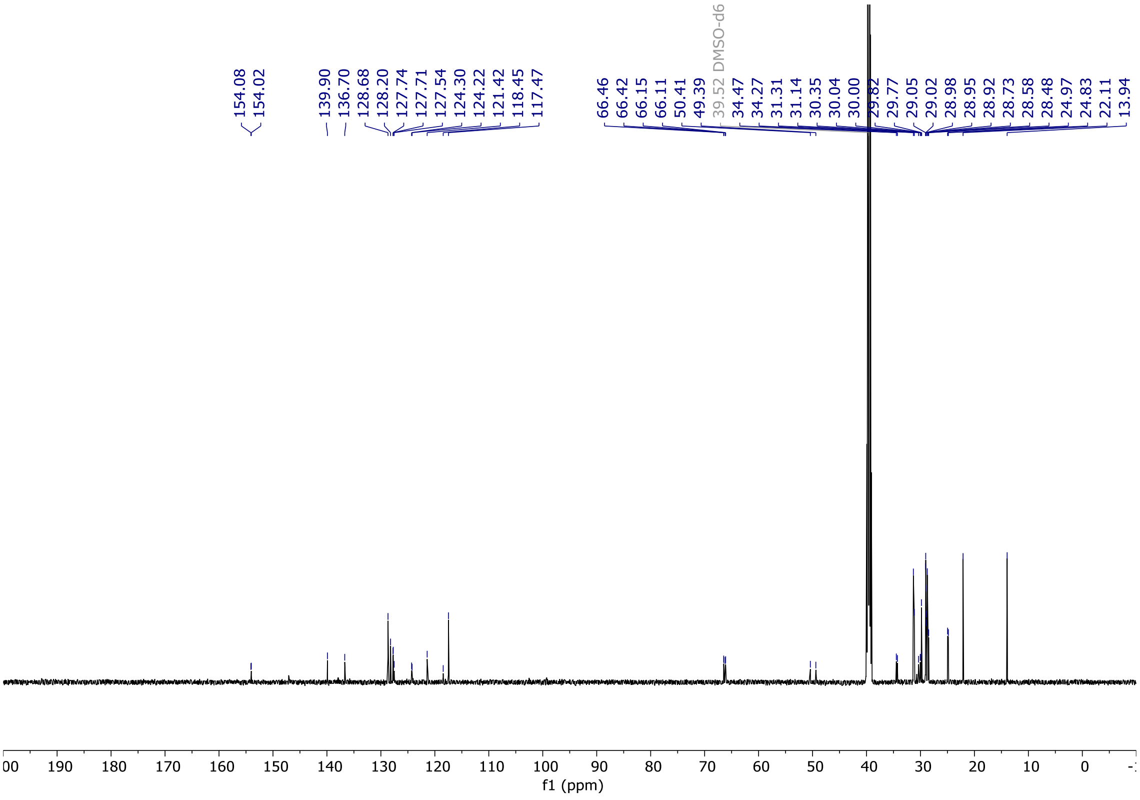

^{31}P-NMR (162 MHz, DMSO-d_6)

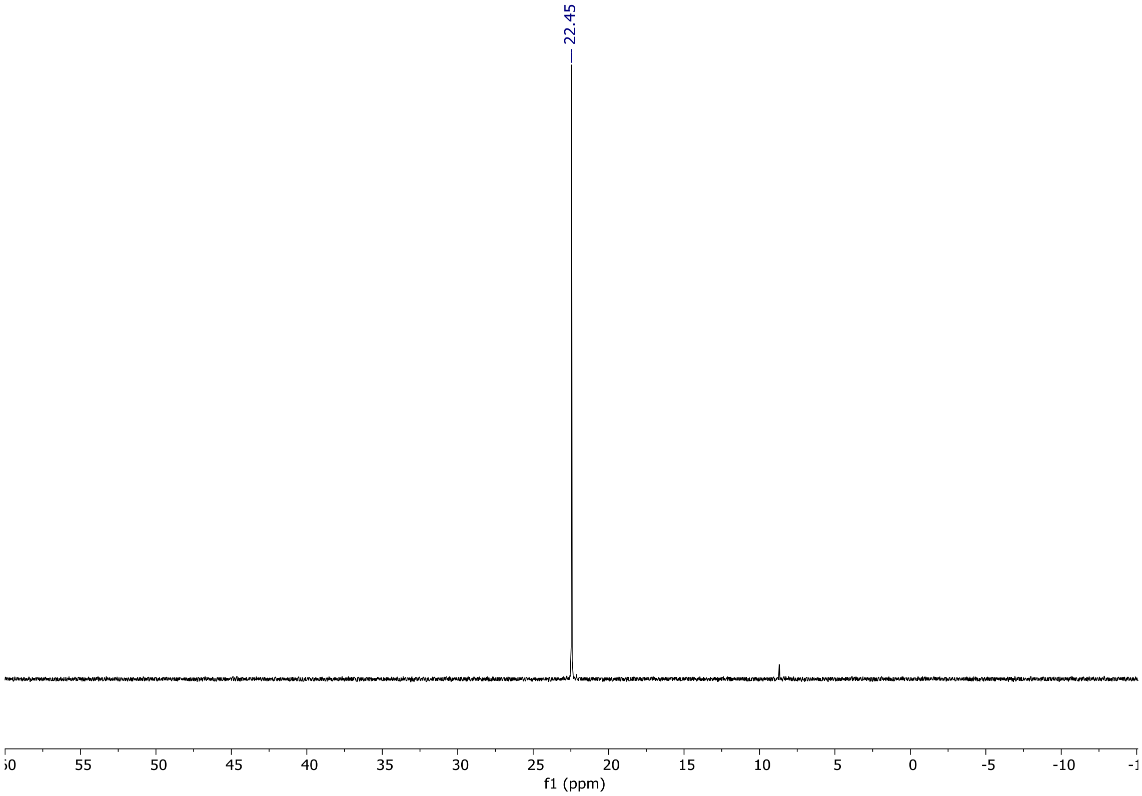

^{1}H-NMR (600 MHz, DMSO-d_6)

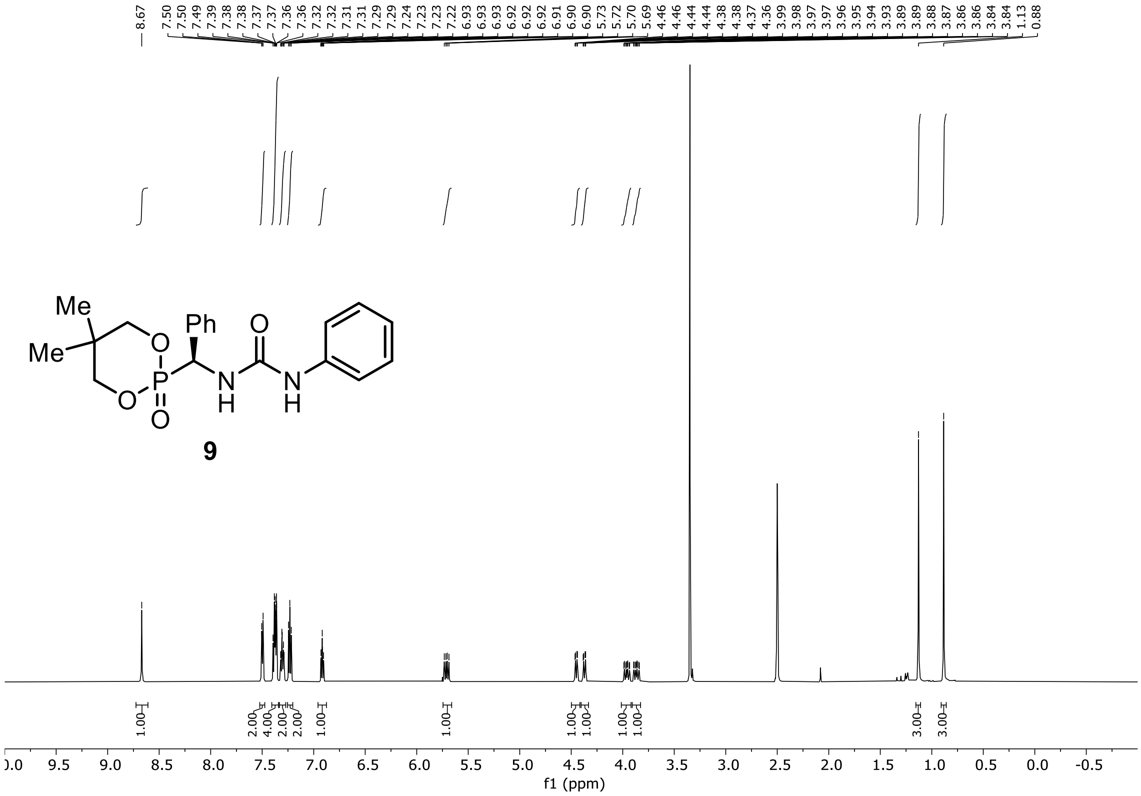

^{13}C-NMR (151 MHz, DMSO-d_6)

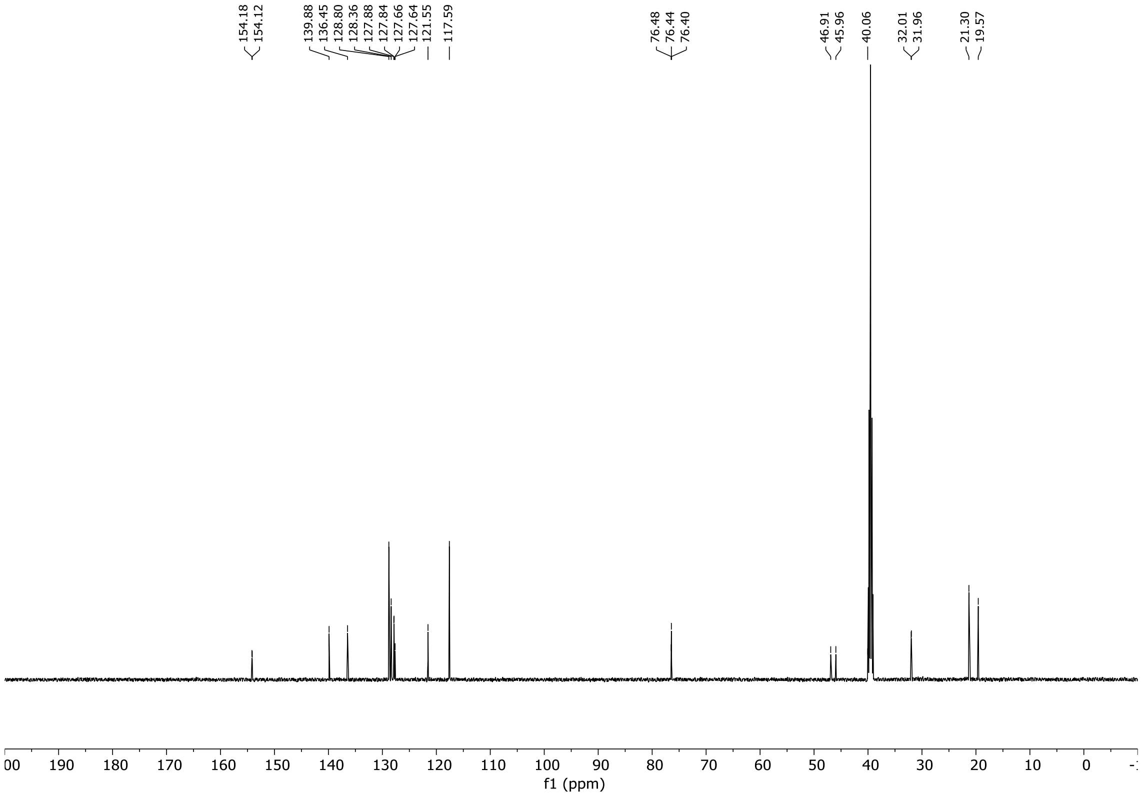

^{31}P-NMR (162 MHz, DMSO-d_6)

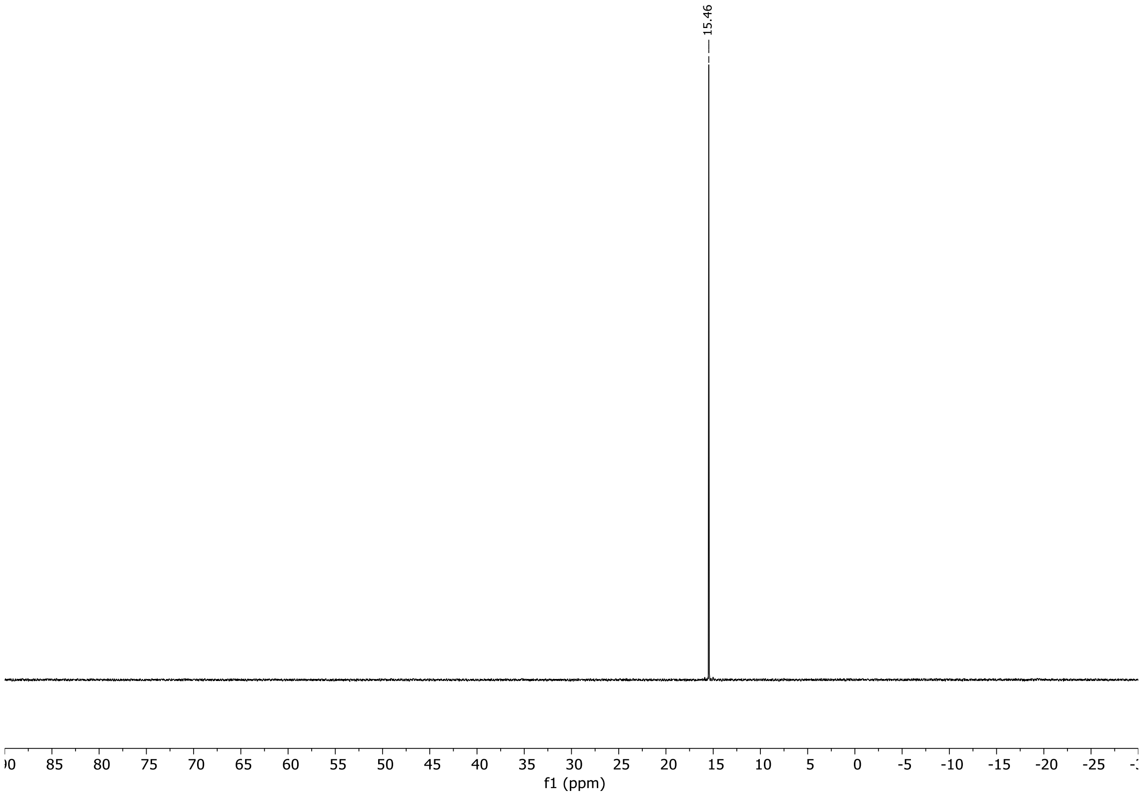

^{1}H-NMR (600 MHz, DMSO-d_6)

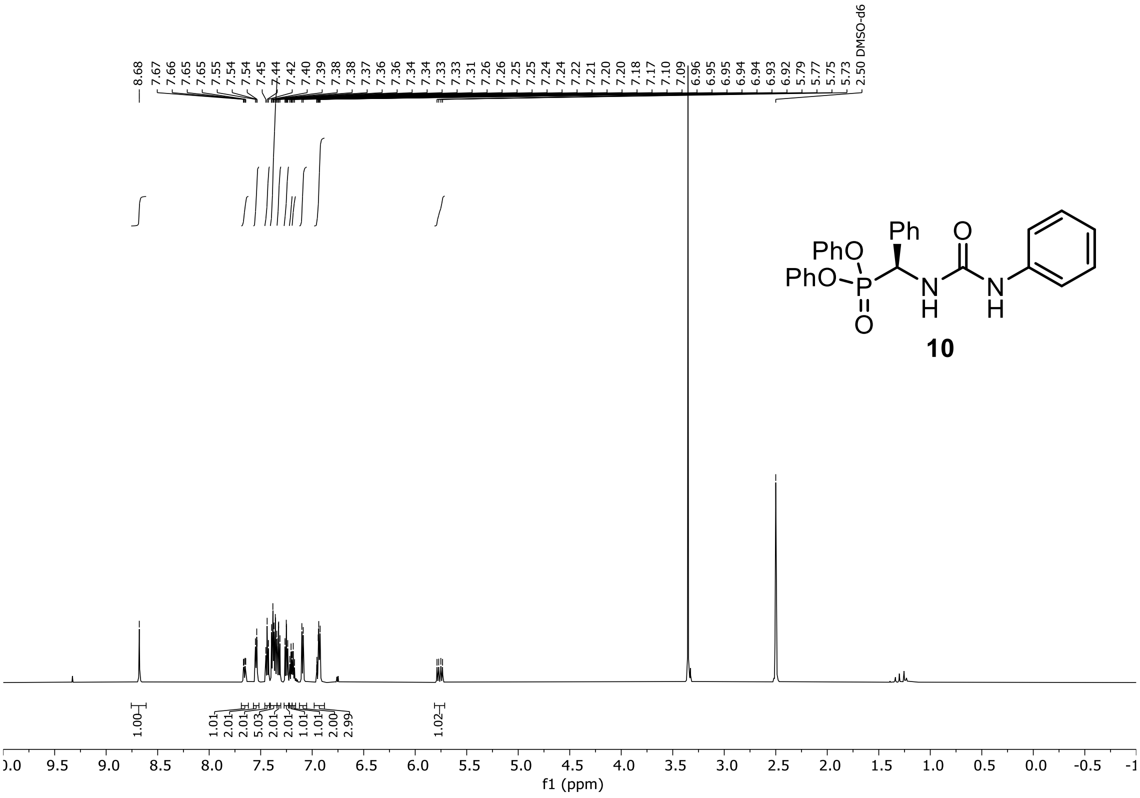

^{13}C-NMR (151 MHz, DMSO-d_6)

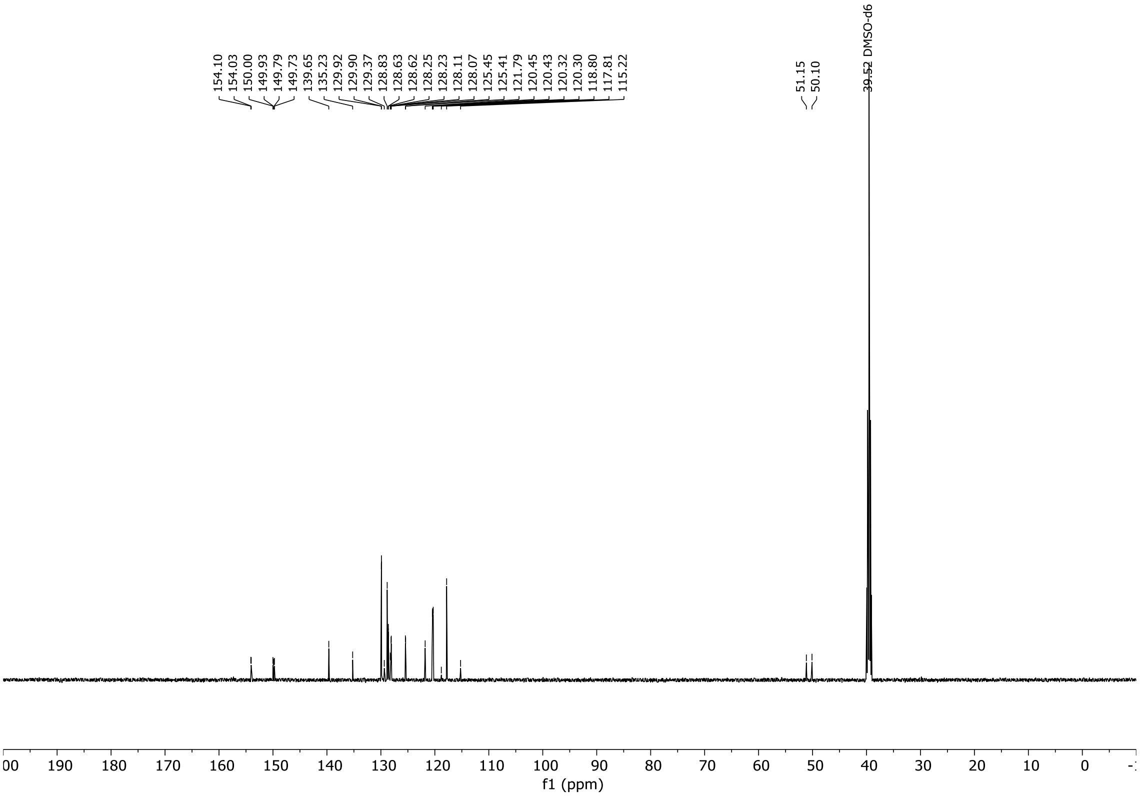

^{31}P-NMR (162 MHz, DMSO-d_6)

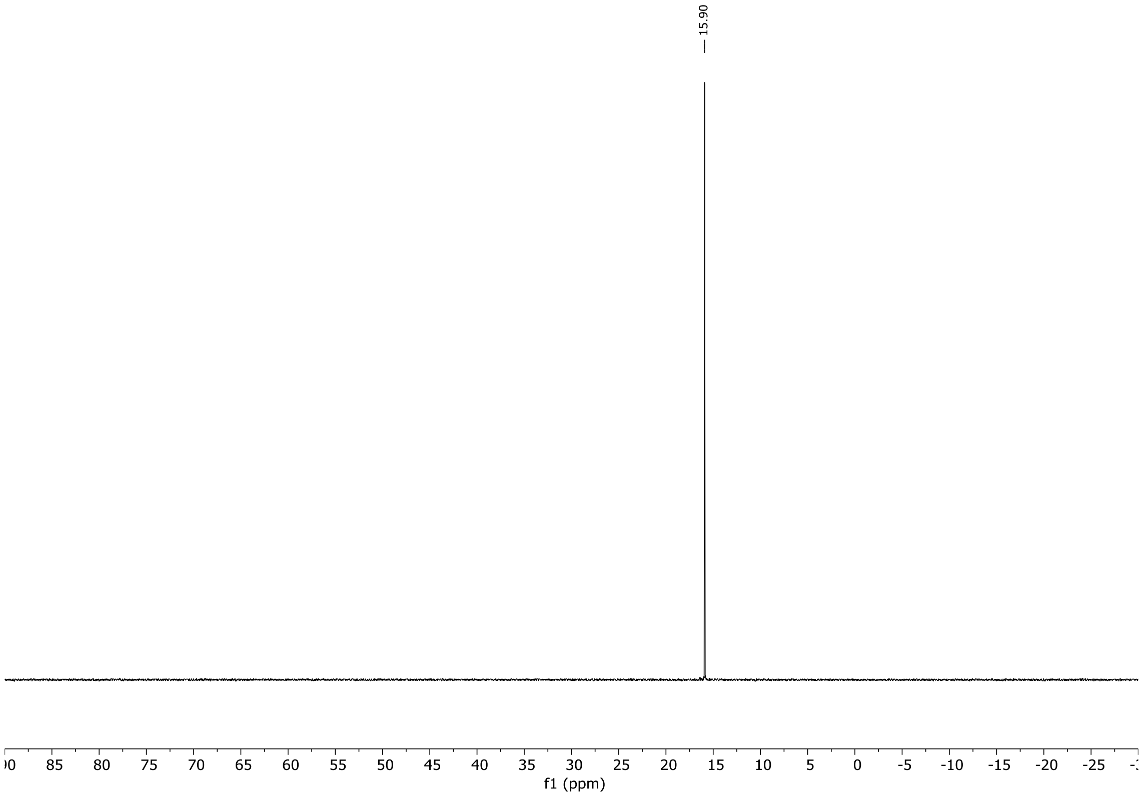

^{1}H-NMR (600 MHz, DMSO-d_6)

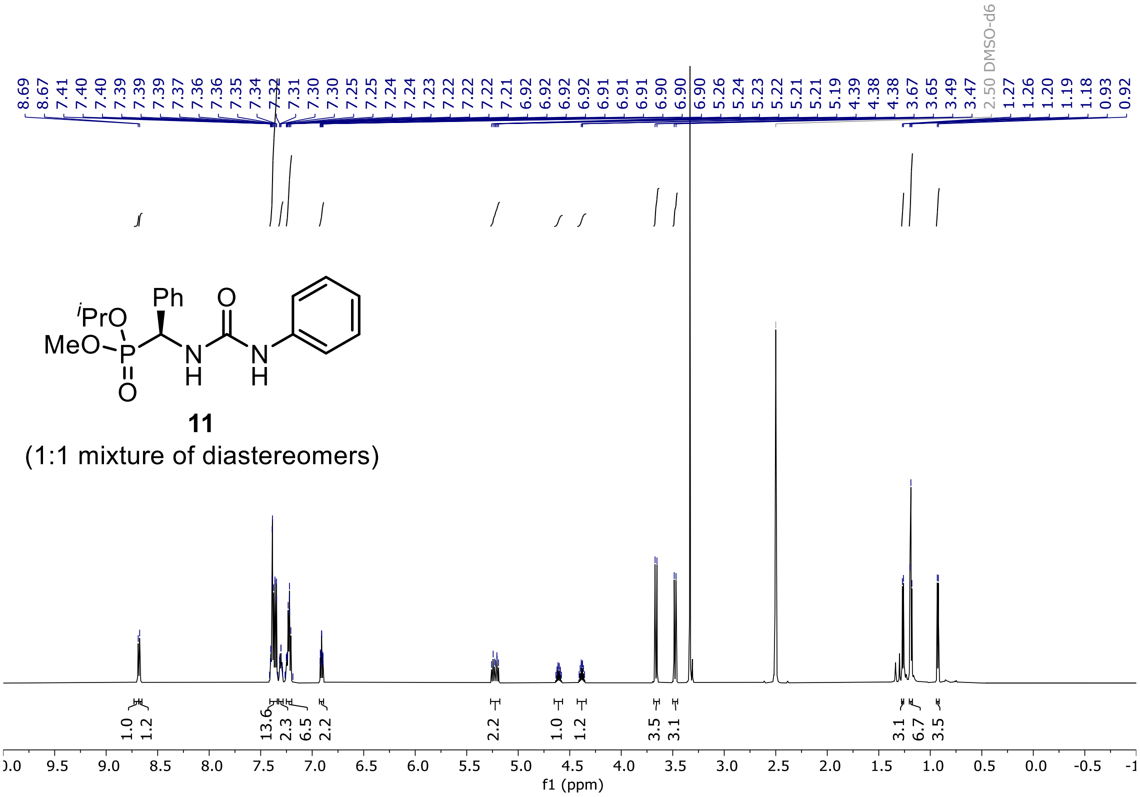

^{13}C-NMR (151 MHz, DMSO-d_6)

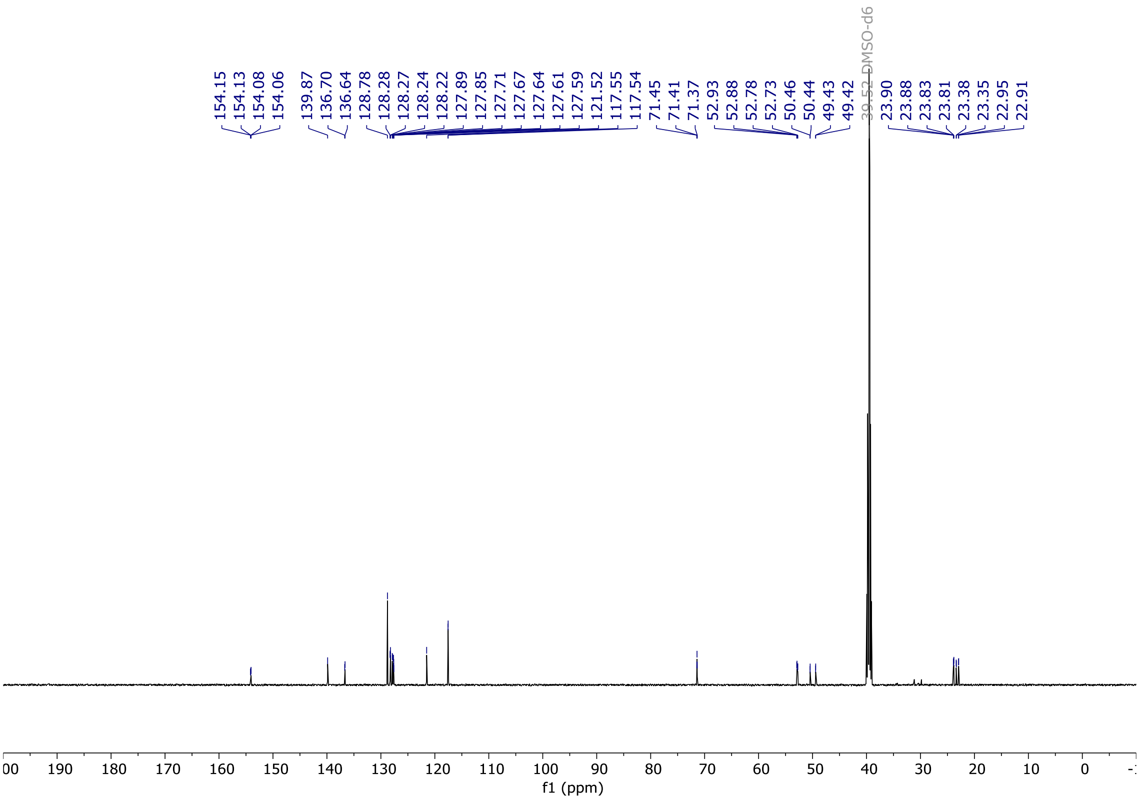

^{31}P-NMR (243 MHz, DMSO-d_6)

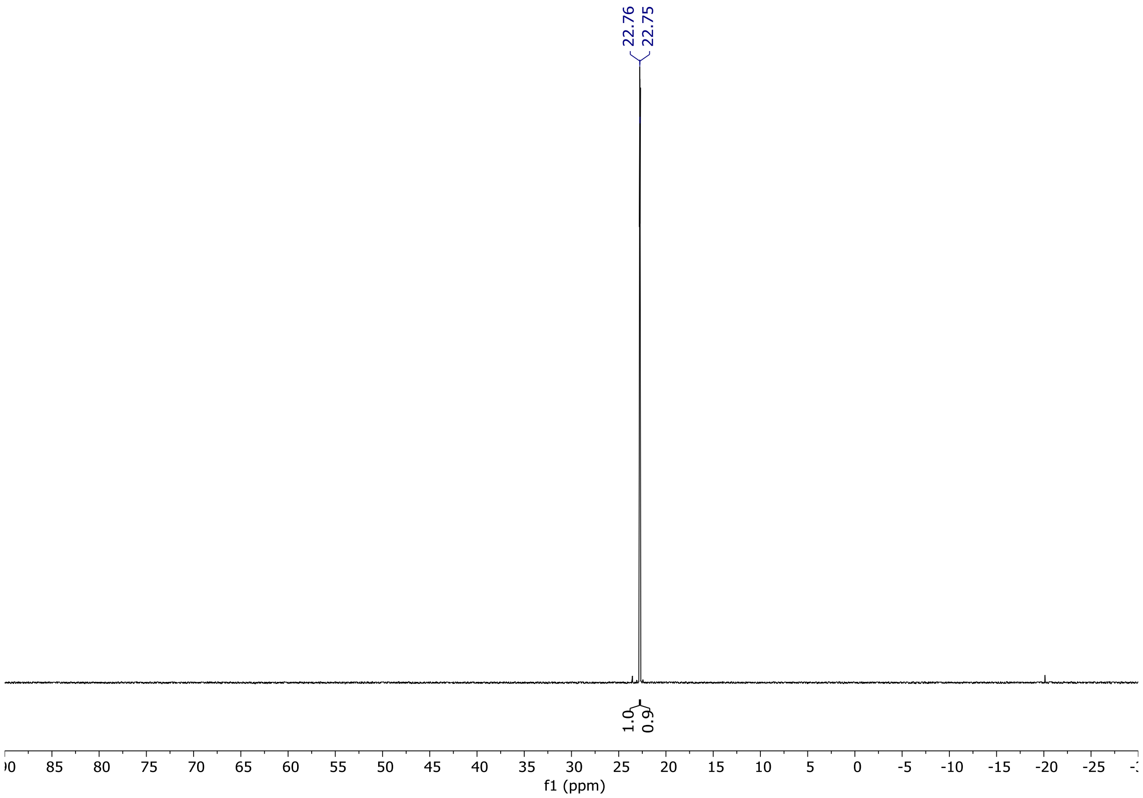

^{1}H-NMR (600 MHz, DMSO-d_6)

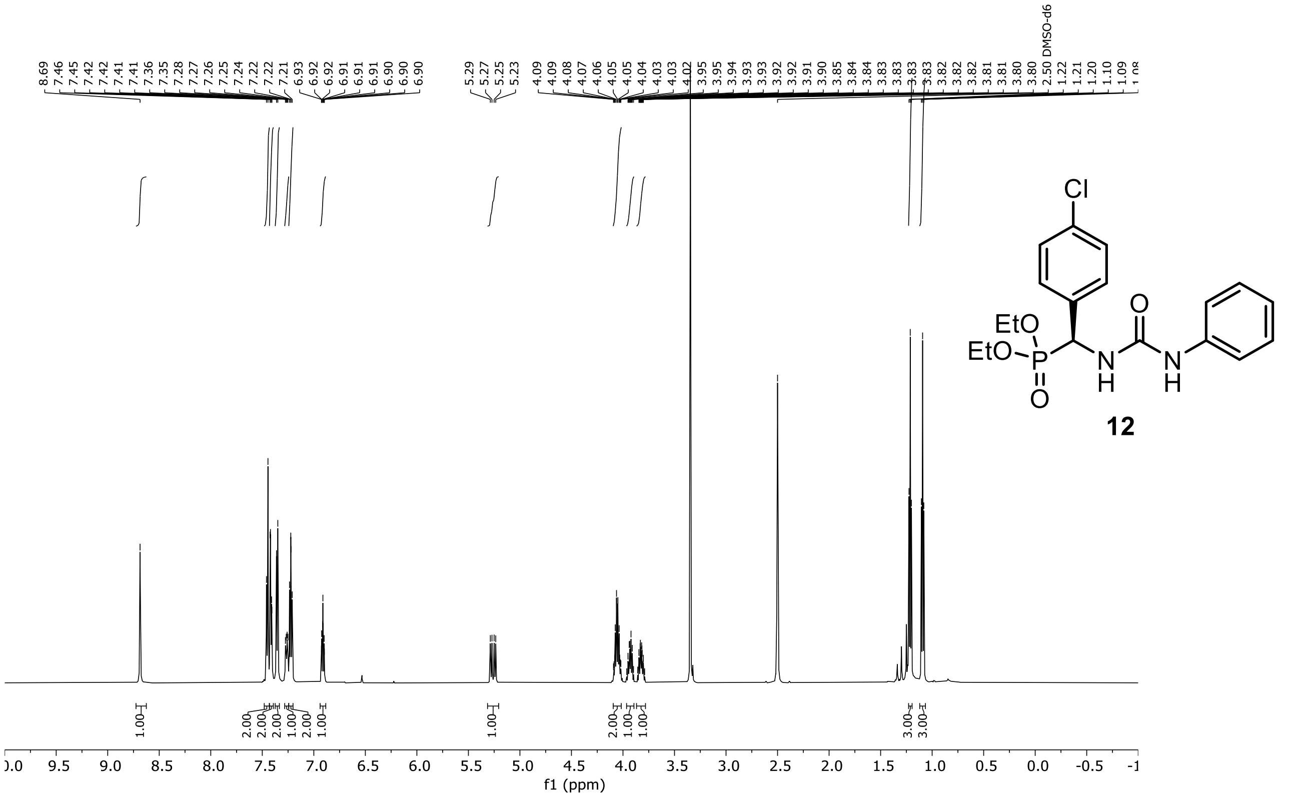

^{13}C-NMR (151 MHz, DMSO-d_6)

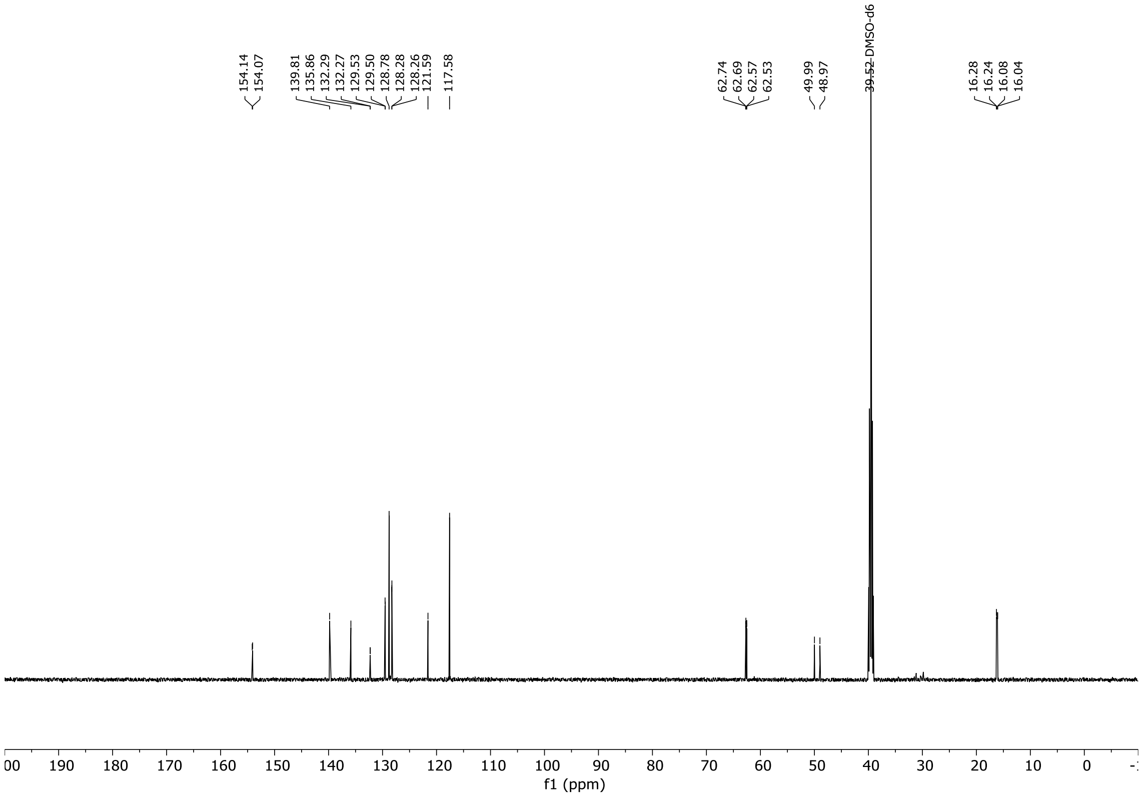

^{31}P-NMR (162 MHz, DMSO-d_6)

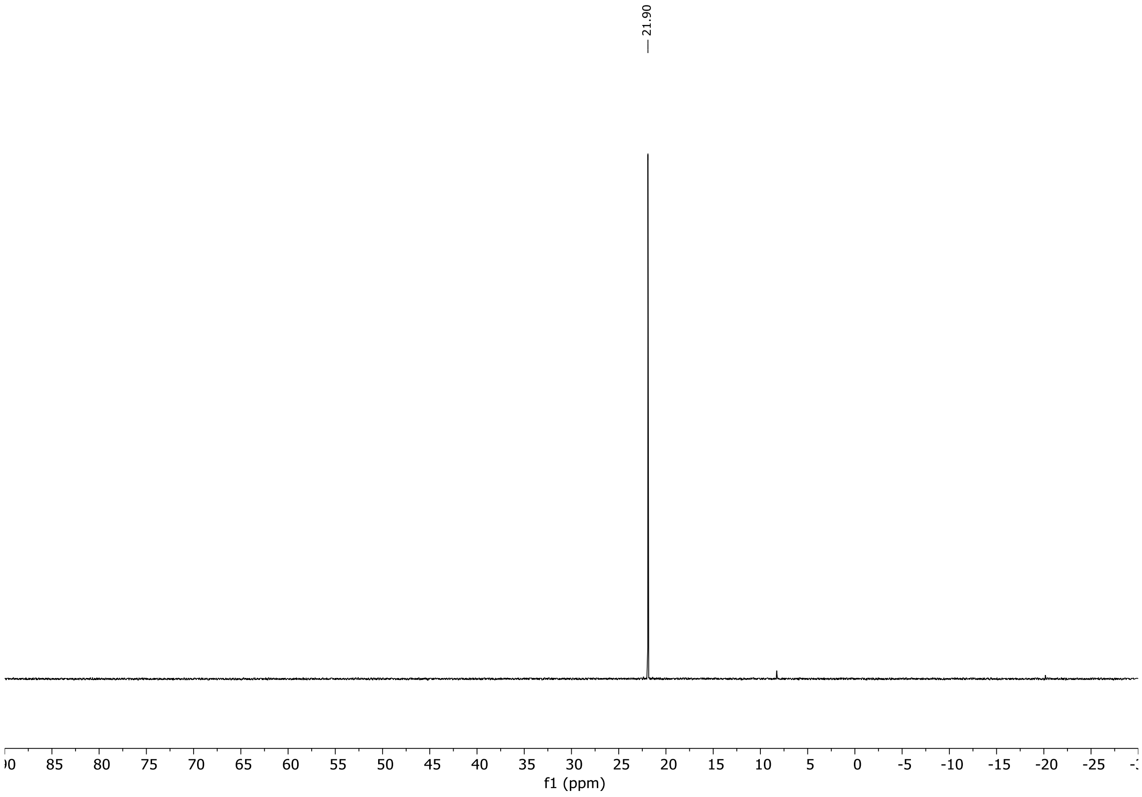

^{1}H-NMR (600 MHz, DMSO-d_6)

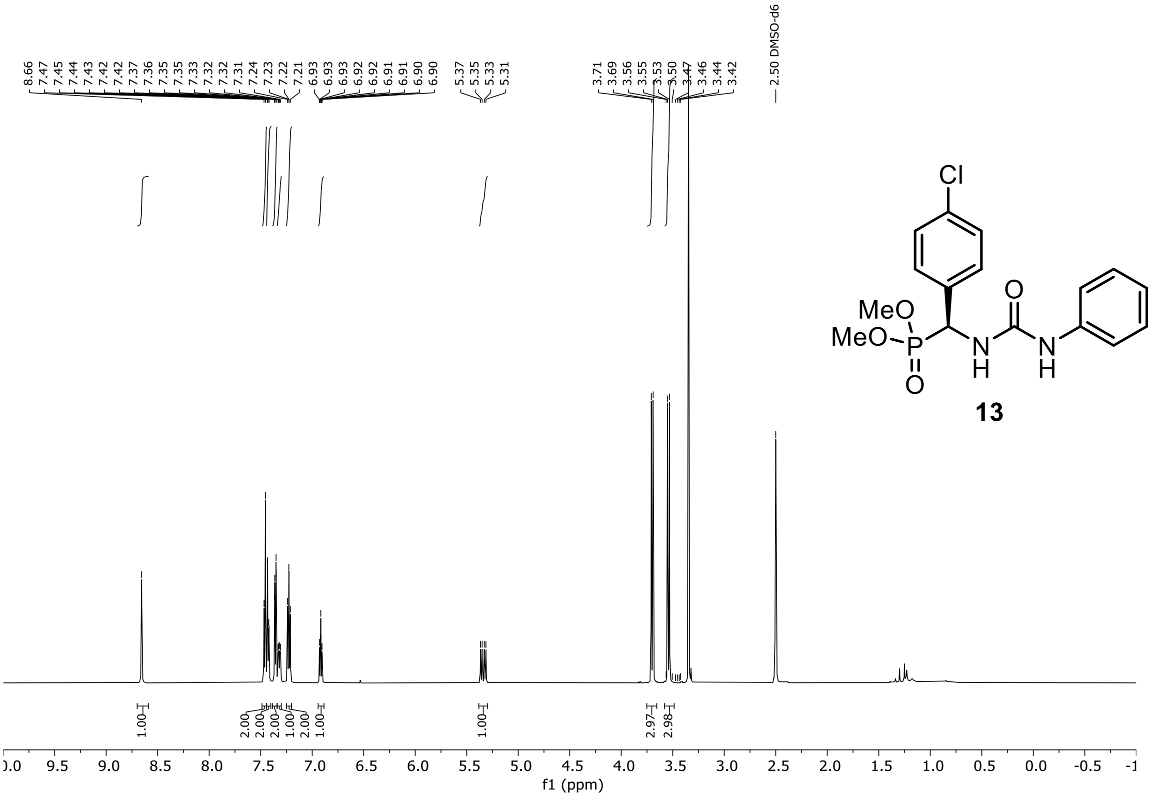

^{13}C-NMR (151 MHz, DMSO-d_6)

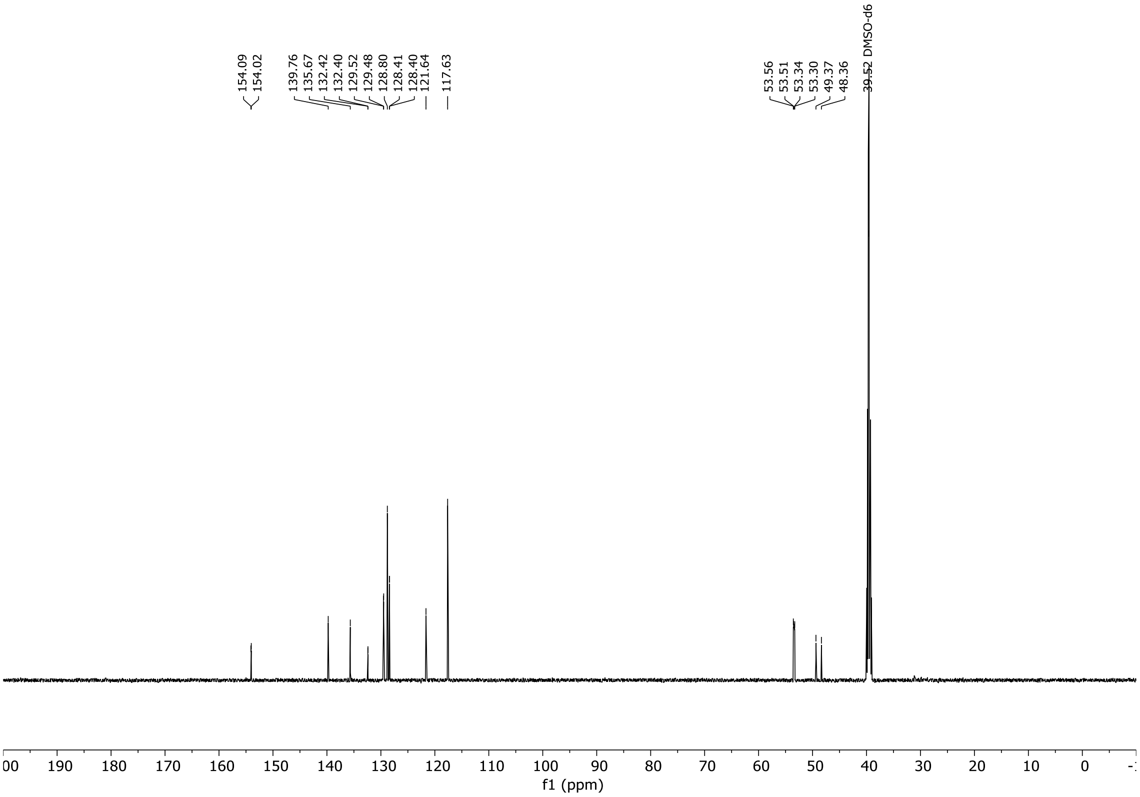

^{31}P-NMR (162 MHz, DMSO-d_6)

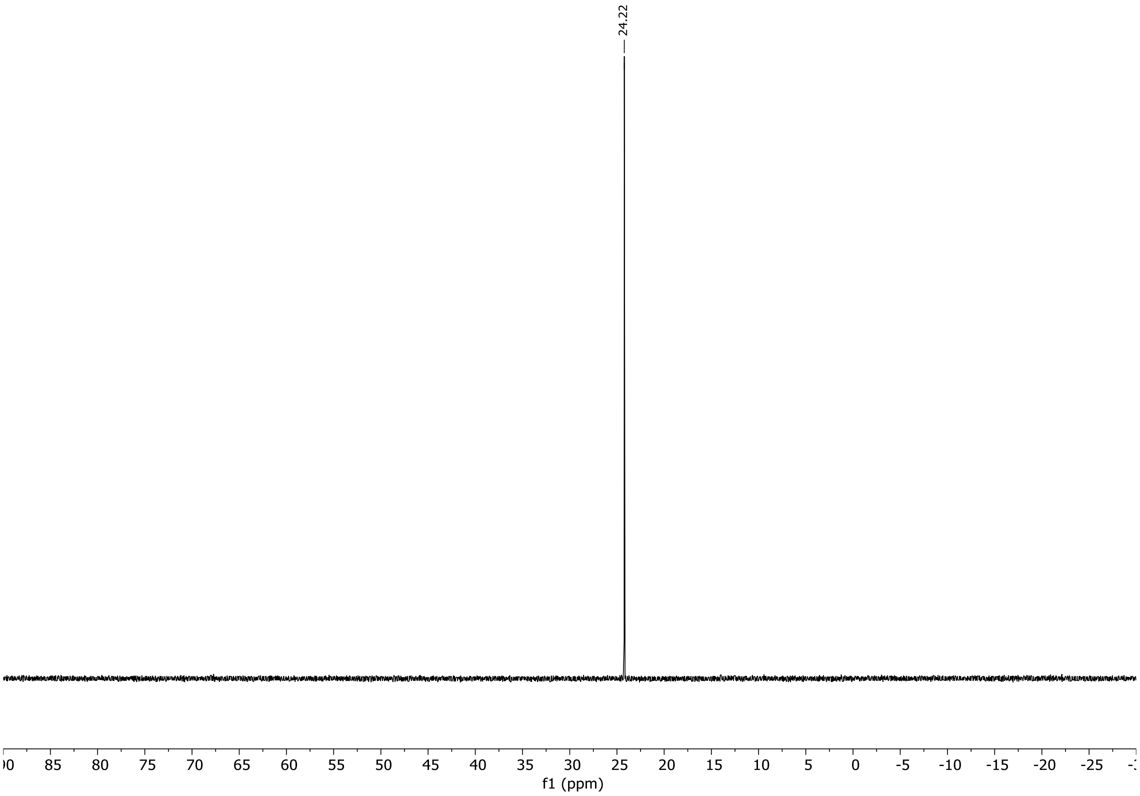

^{1}H-NMR (600 MHz, DMSO-d_6)

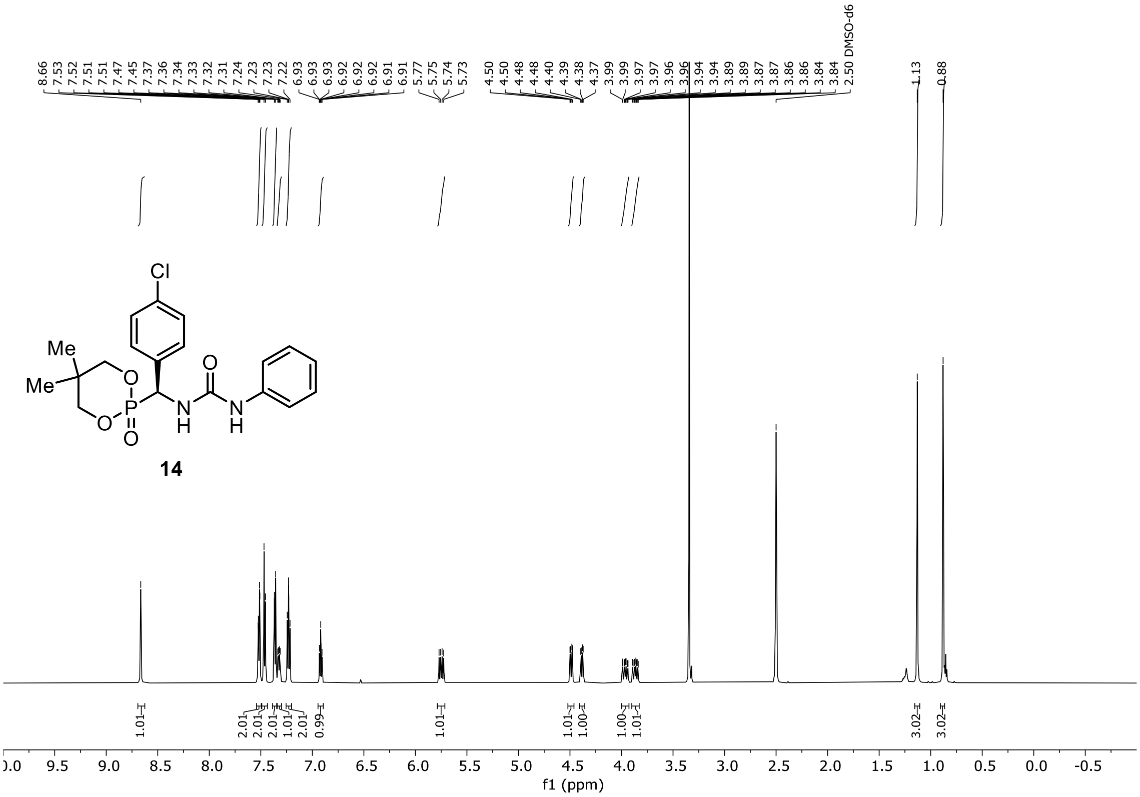

14

^{13}C-NMR (151 MHz, DMSO-d_6)

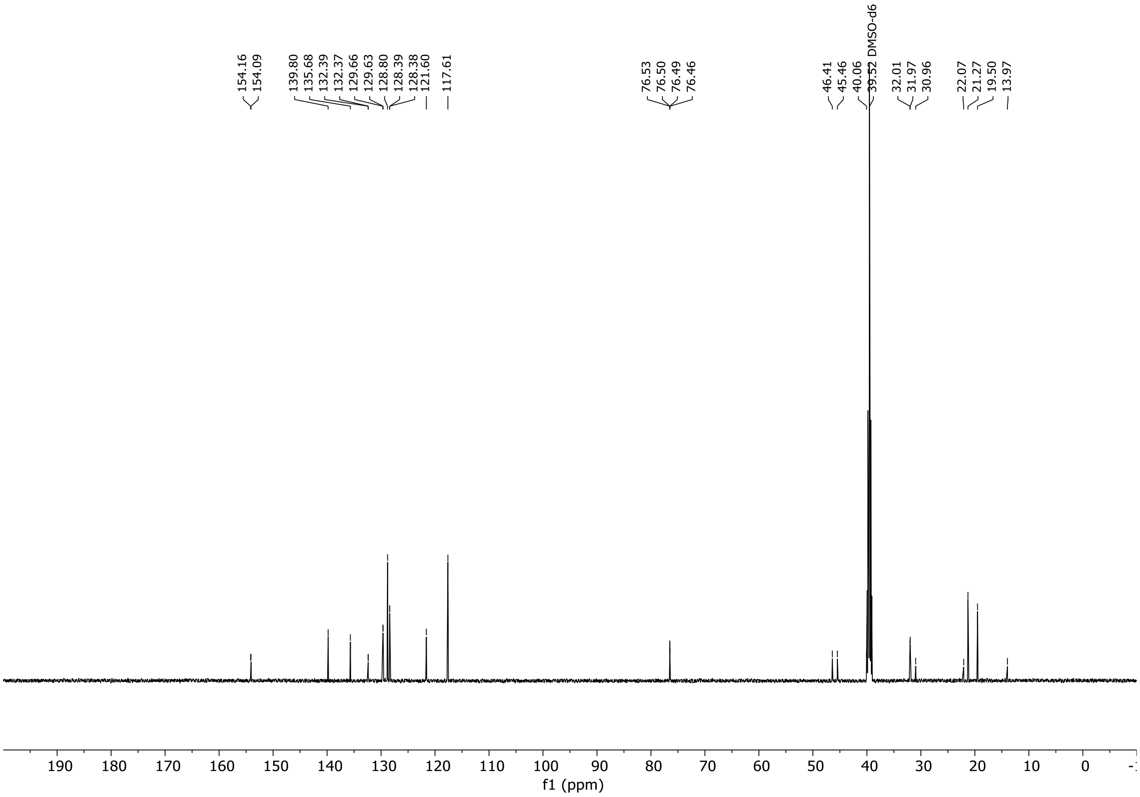

^{31}P-NMR (162 MHz, DMSO-d_6)

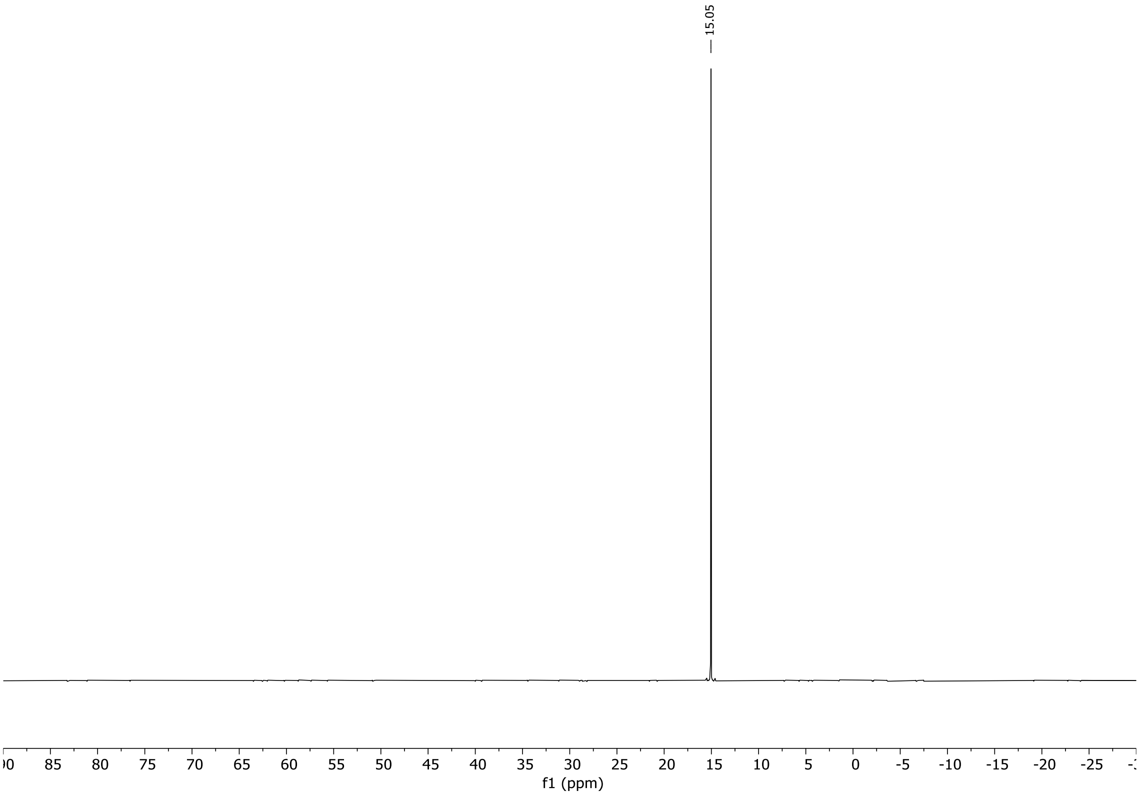

^{1}H-NMR (600 MHz, DMSO-d_6)

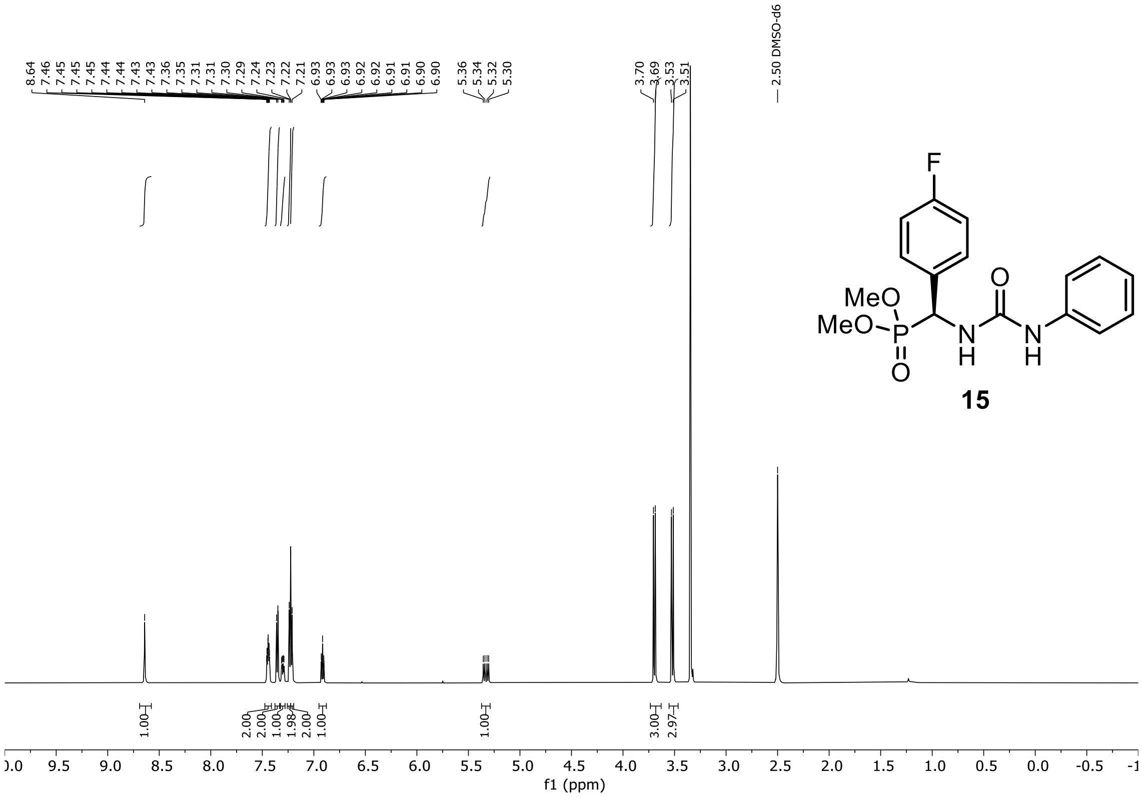

^{13}C-NMR (151 MHz, DMSO-d_6)

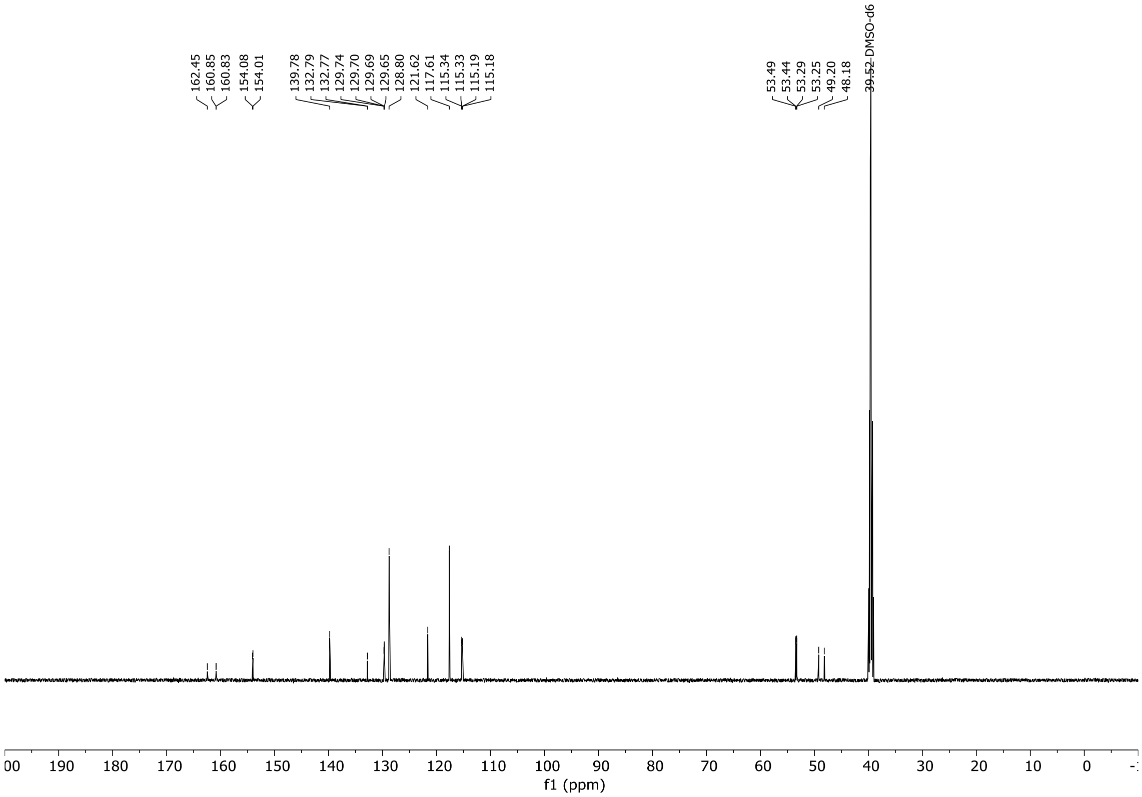

^{31}P-NMR (162 MHz, DMSO-d_6)

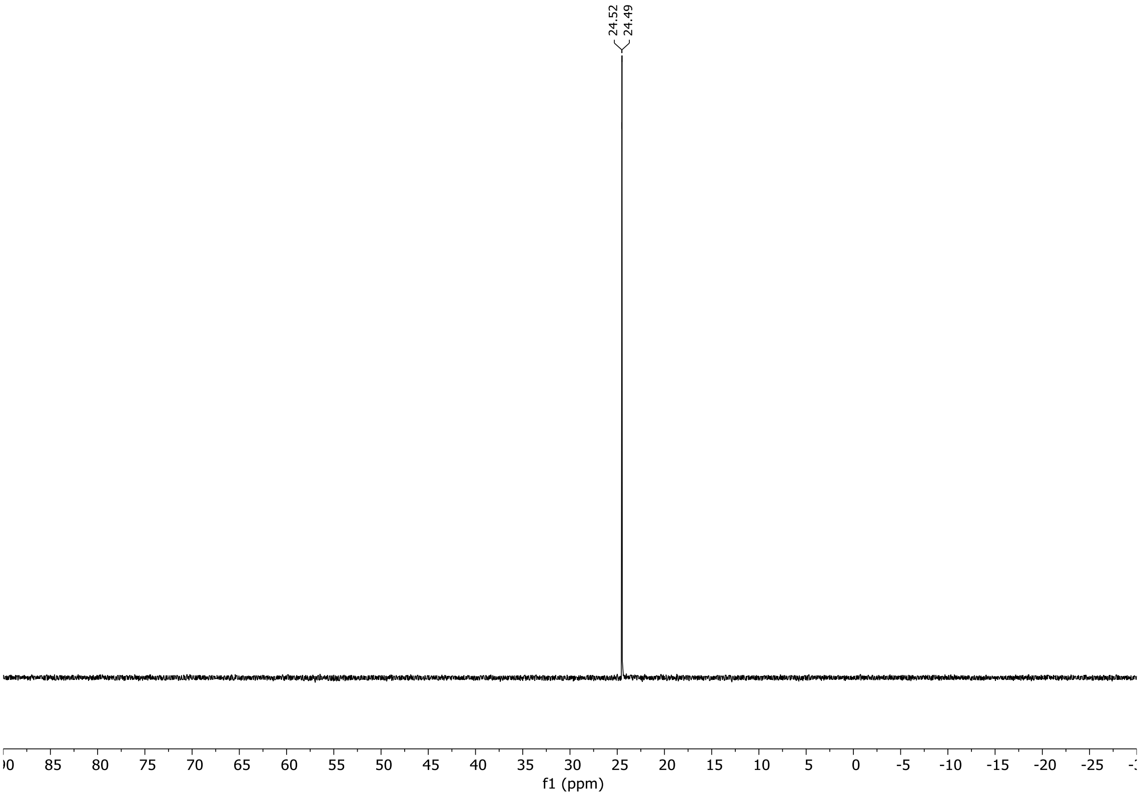

^{19}F-NMR (376 MHz, DMSO-d_6)

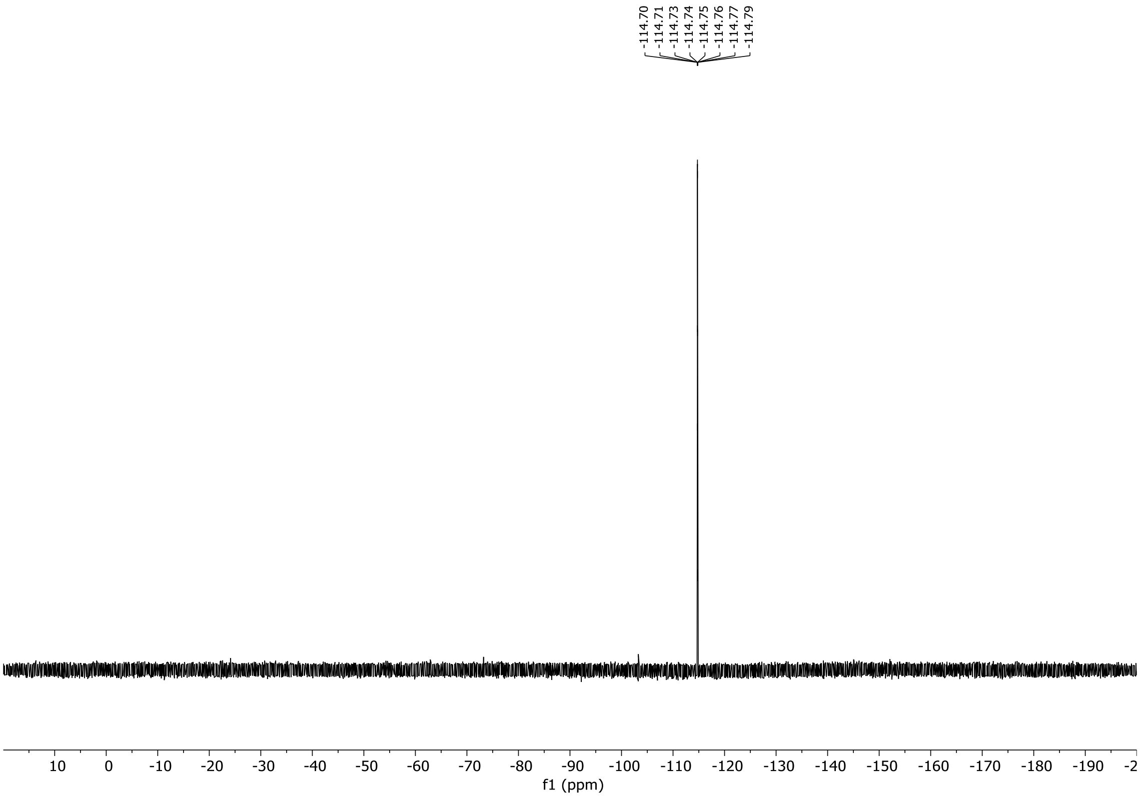

^{1}H-NMR (600 MHz, DMSO-d_6)

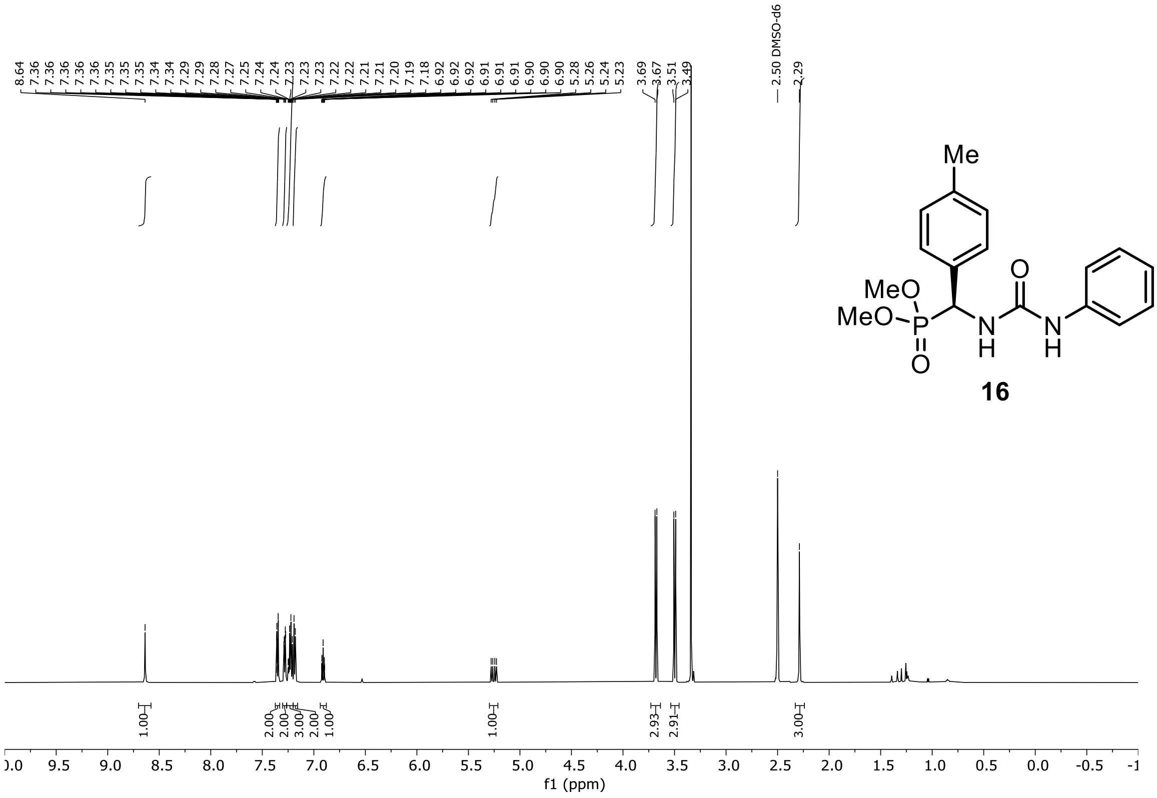

^{13}C-NMR (151 MHz, DMSO-d_6)

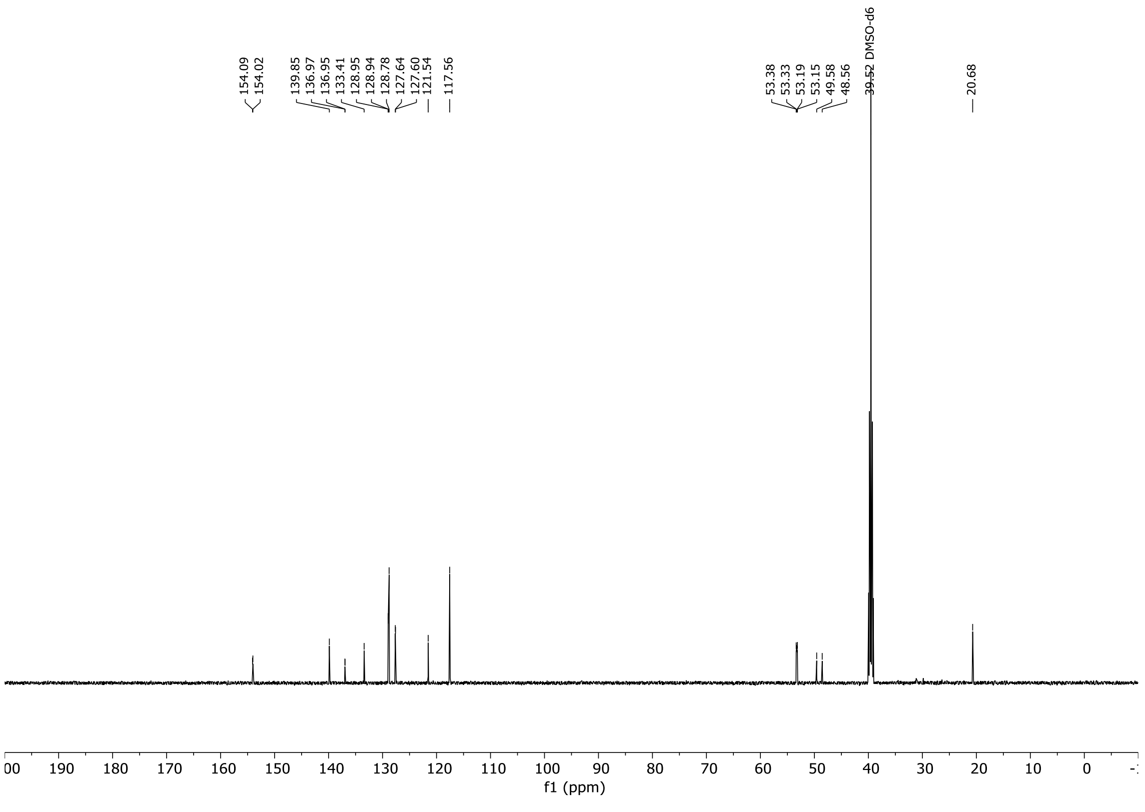

^{31}P-NMR (162 MHz, DMSO-*d*$_6$)

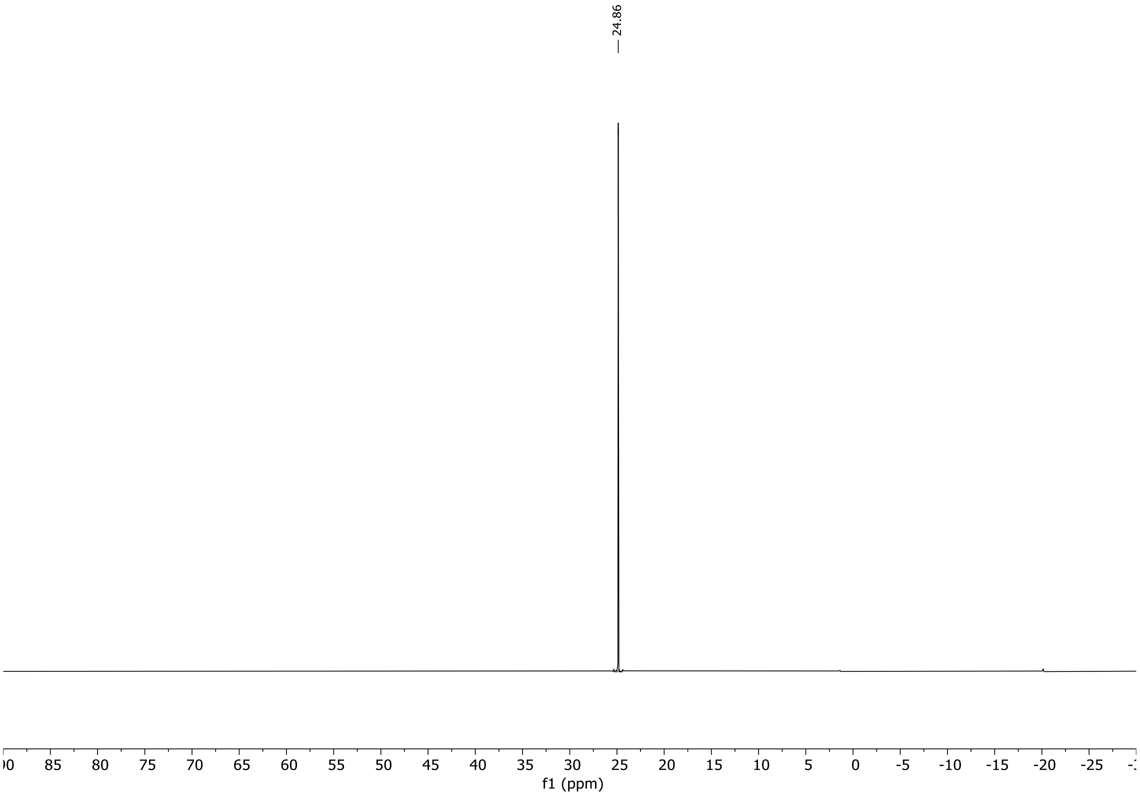

^{1}H-NMR (600 MHz, DMSO-d_6)

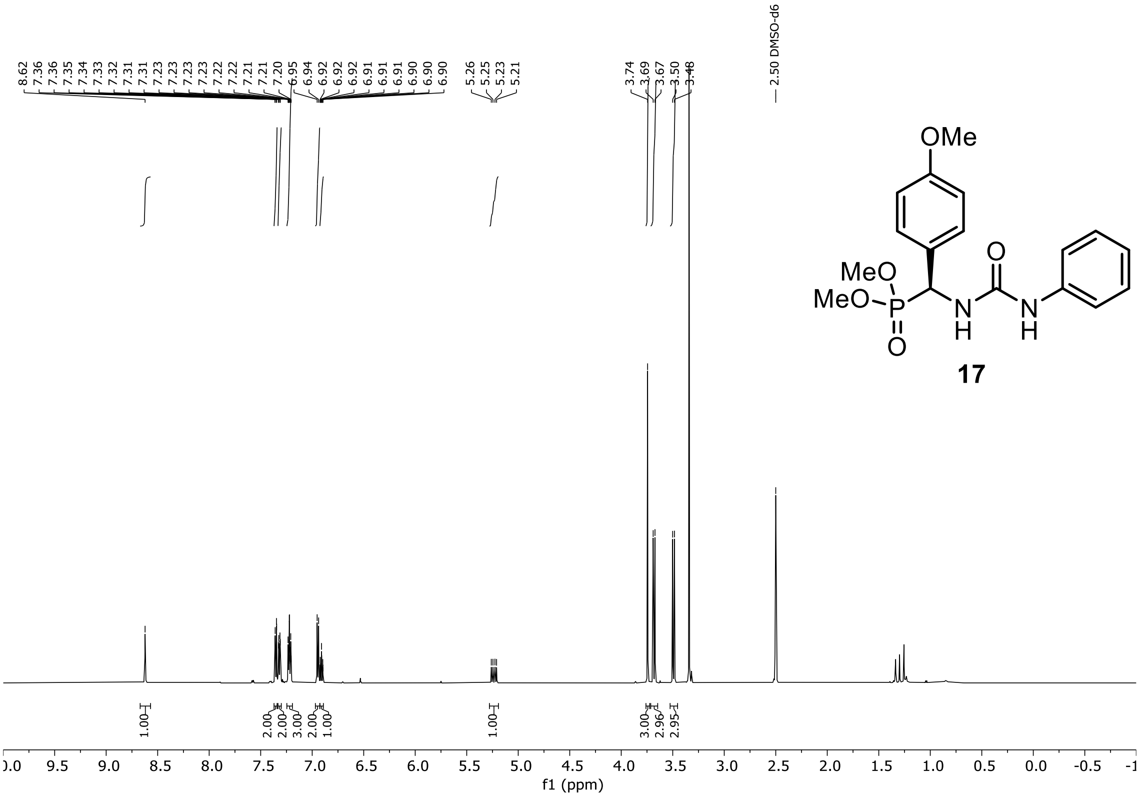

^{13}C-NMR (151 MHz, DMSO-d_6)

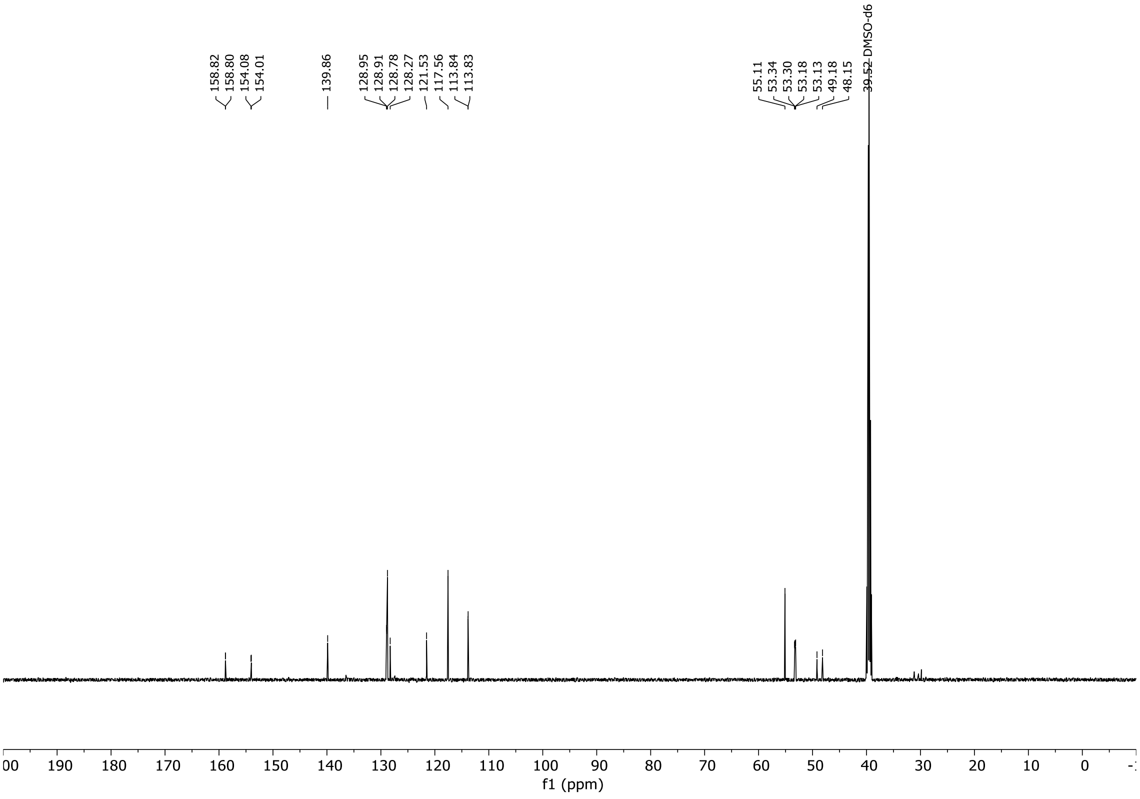

^{31}P-NMR (162 MHz, DMSO-d_6)

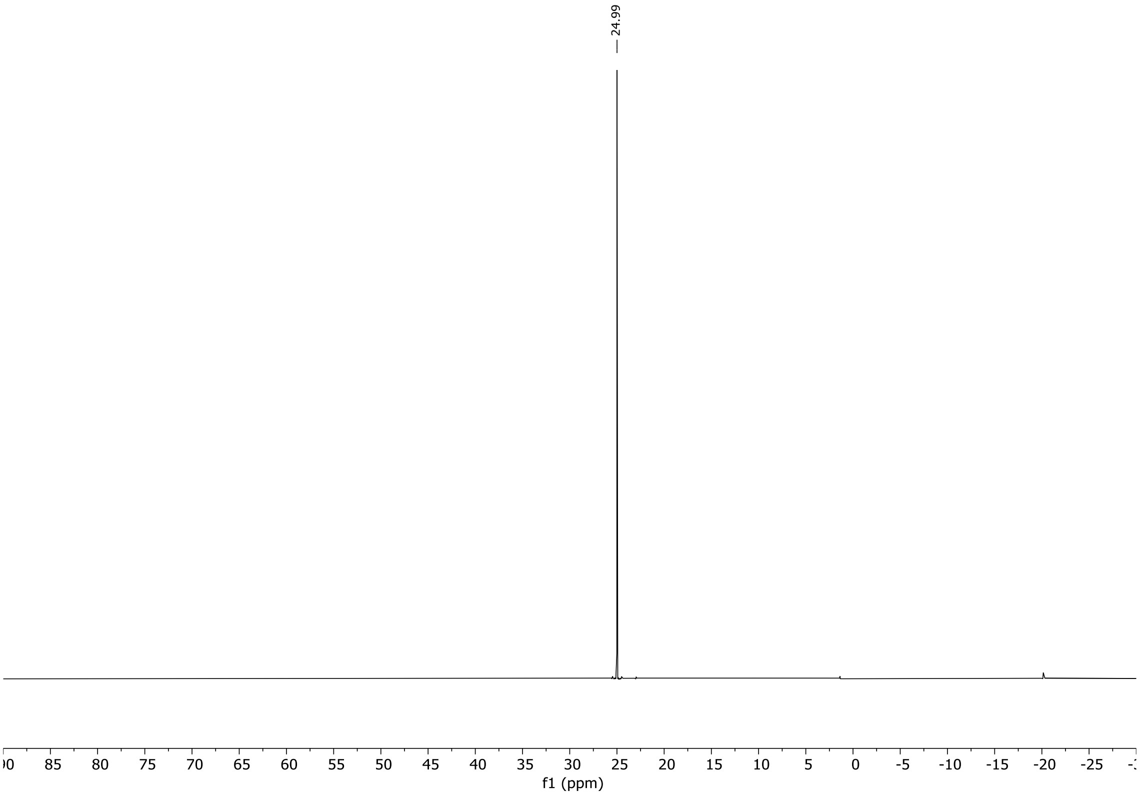

^{1}H-NMR (600 MHz, MeOD-*d*$_4$)

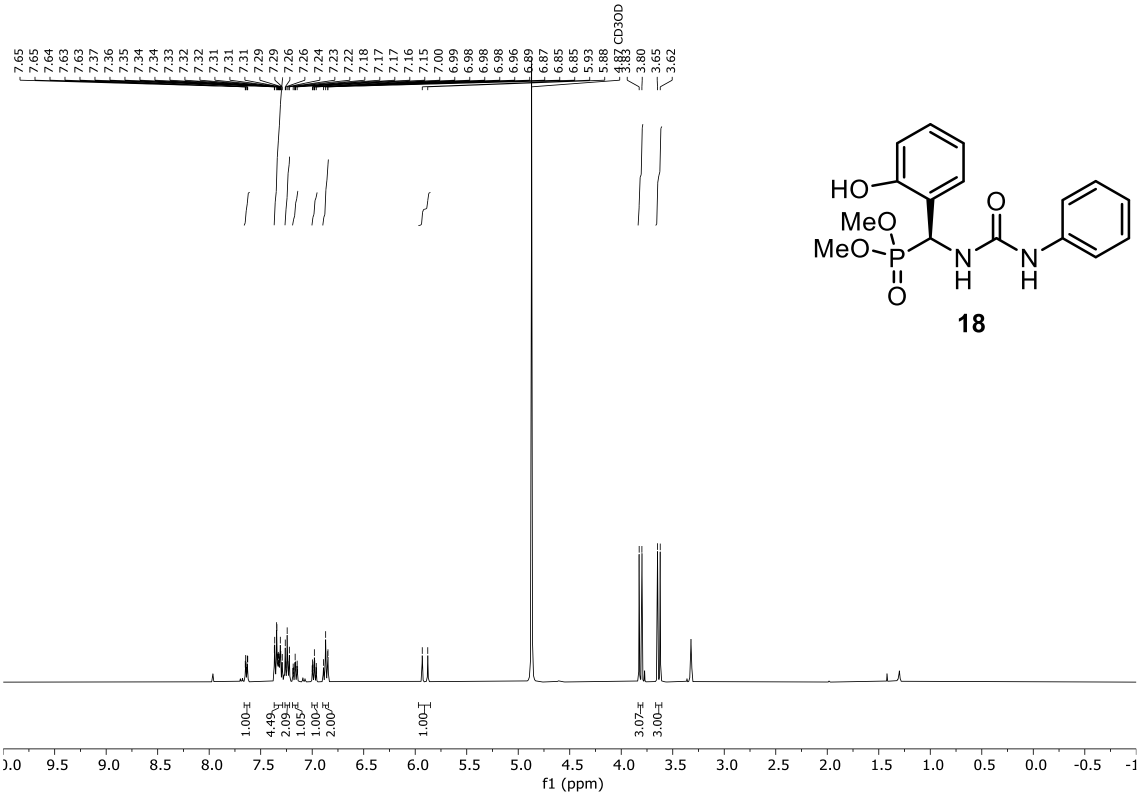

18

^{13}C-NMR (151 MHz, DMSO-*d*$_6$)

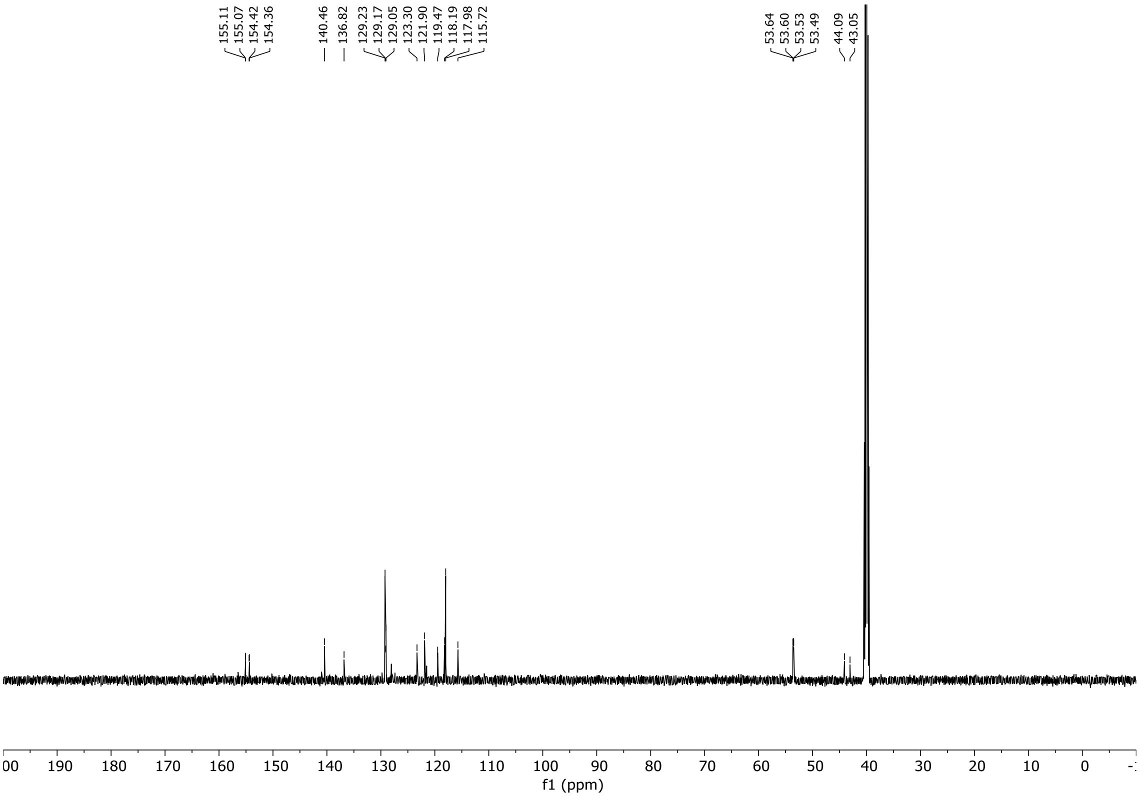

^{31}P-NMR (162 MHz, DMSO-d_6)

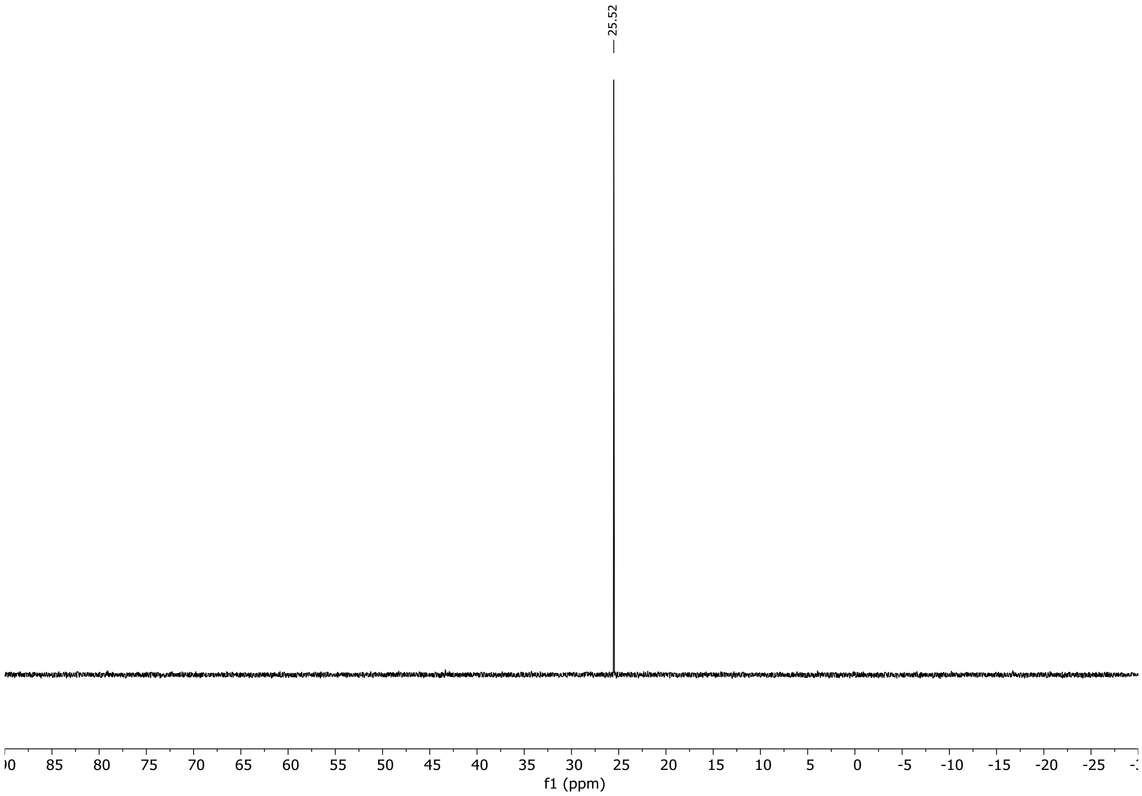

^{1}H-NMR (600 MHz, DMSO-d_6)

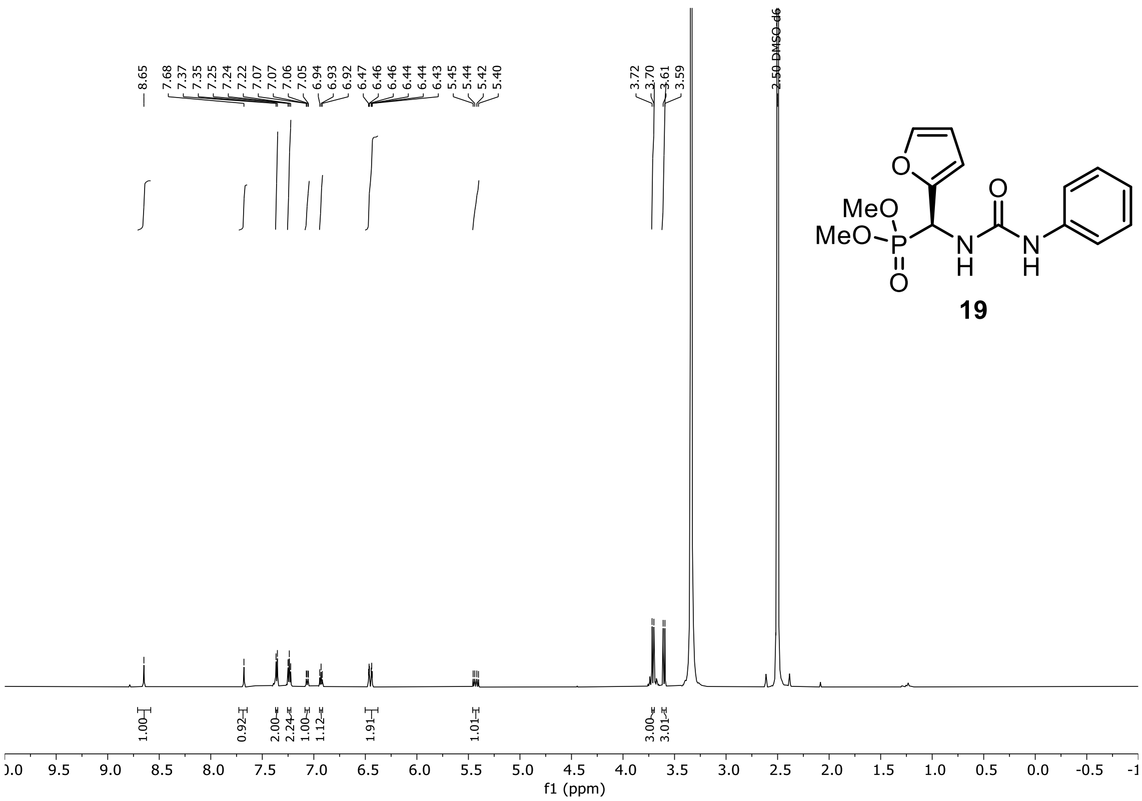

^{13}C-NMR (151 MHz, DMSO-d_6)

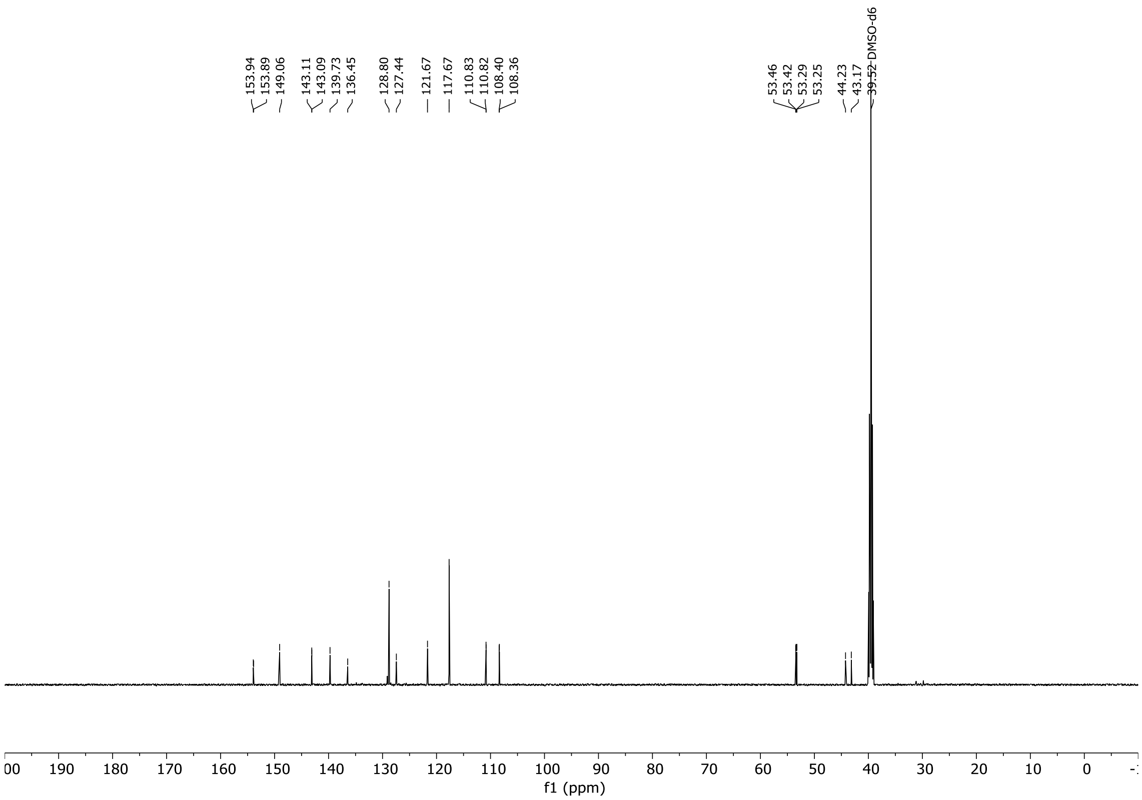

^{31}P-NMR (243 MHz, DMSO-d_6)

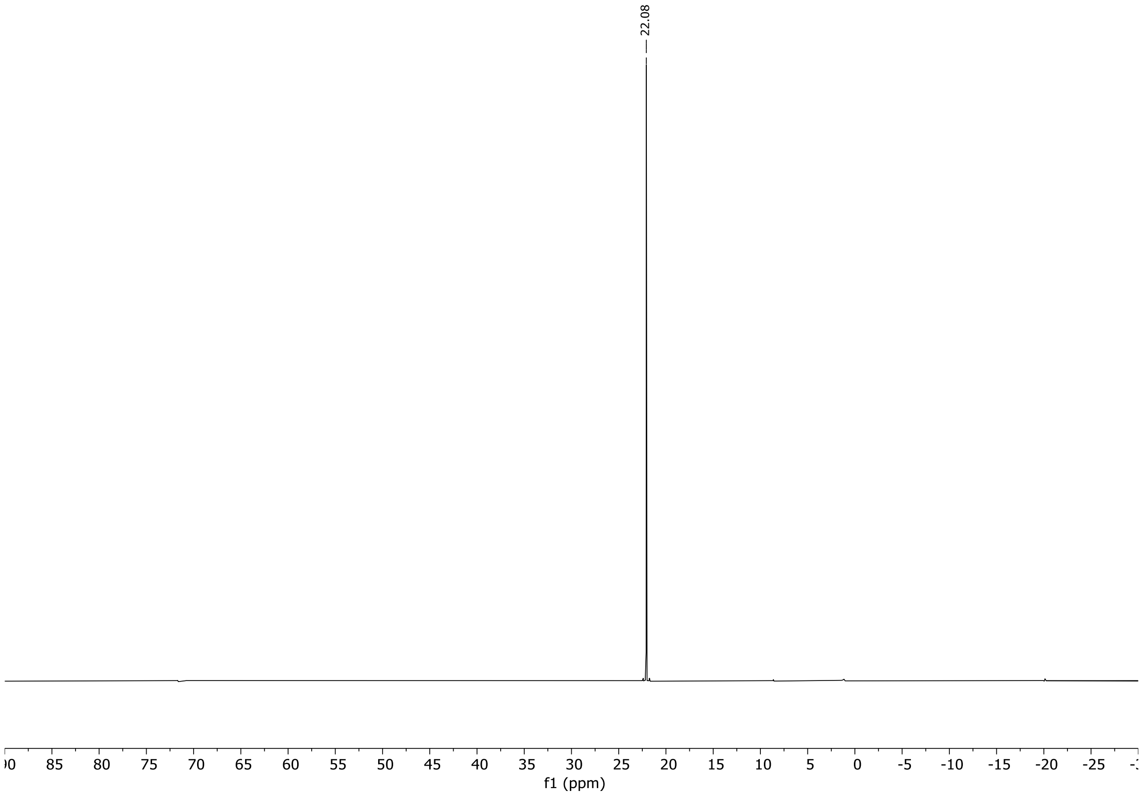

^{1}H-NMR (600 MHz, DMSO-d_6)

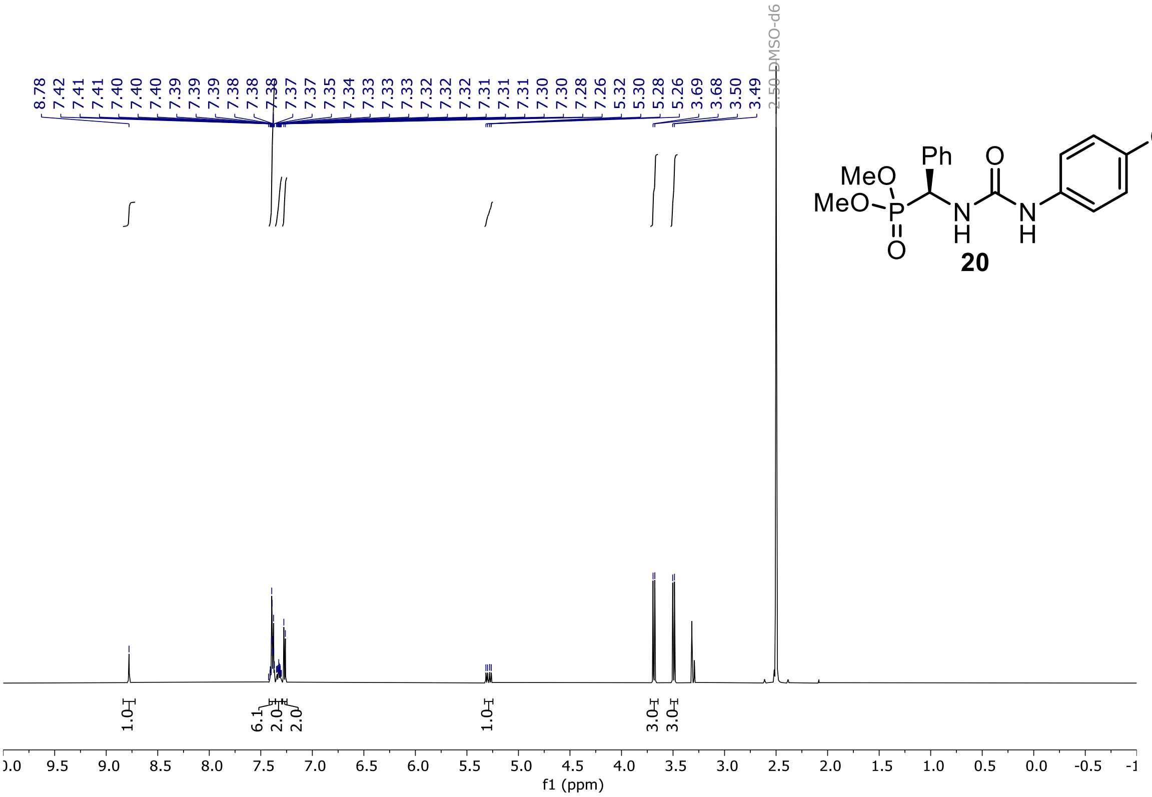

20

^{13}C-NMR (151 MHz, DMSO-d_6)

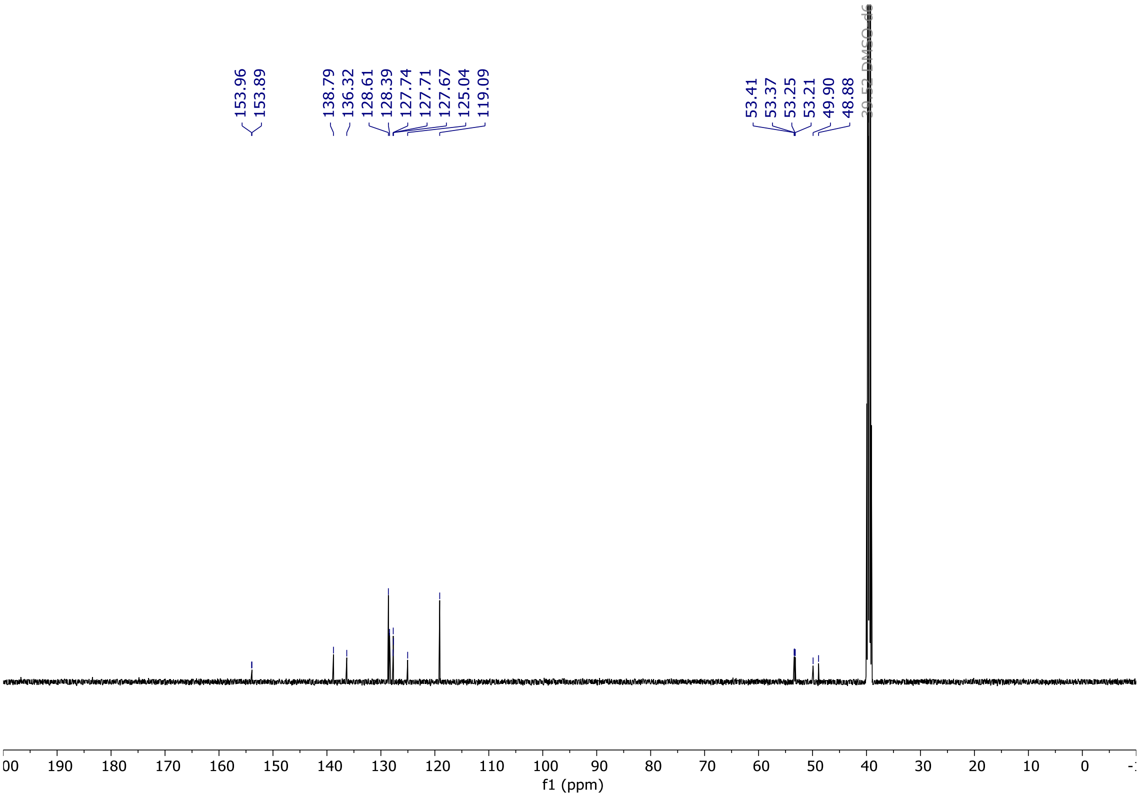

^{31}P-NMR (243 MHz, DMSO-d_6)

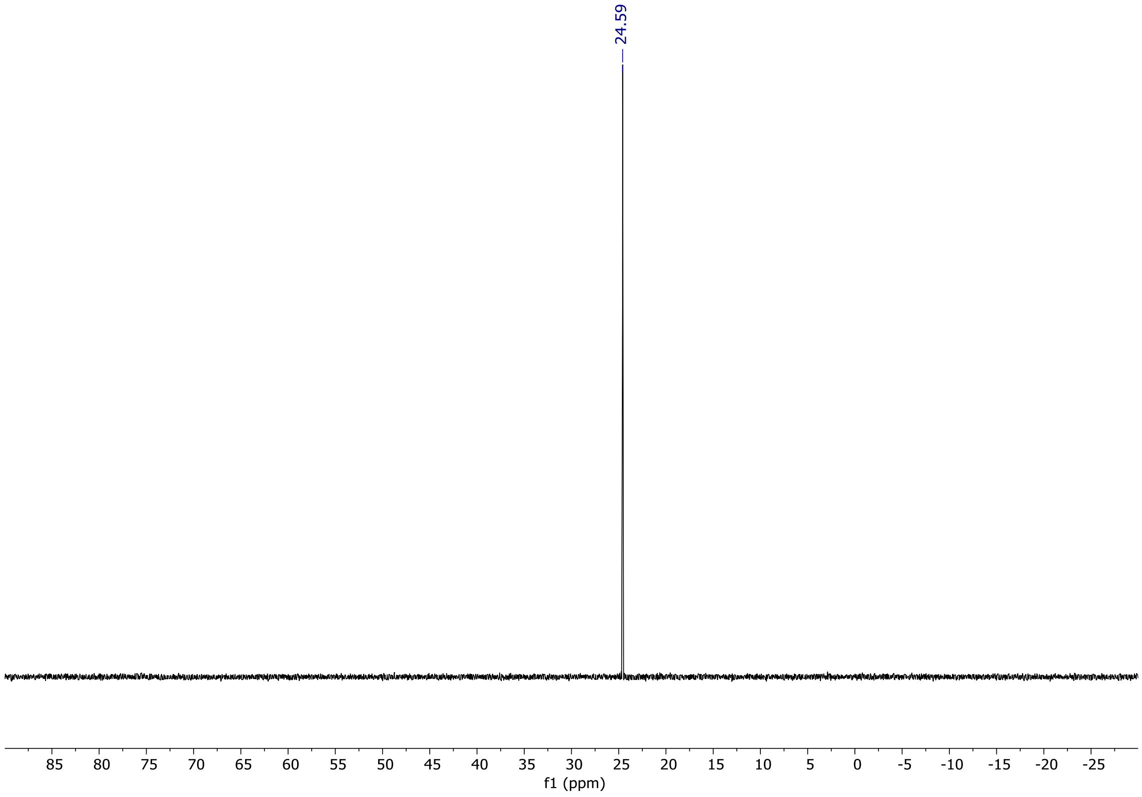

^{1}H-NMR (600 MHz, DMSO-d_6)

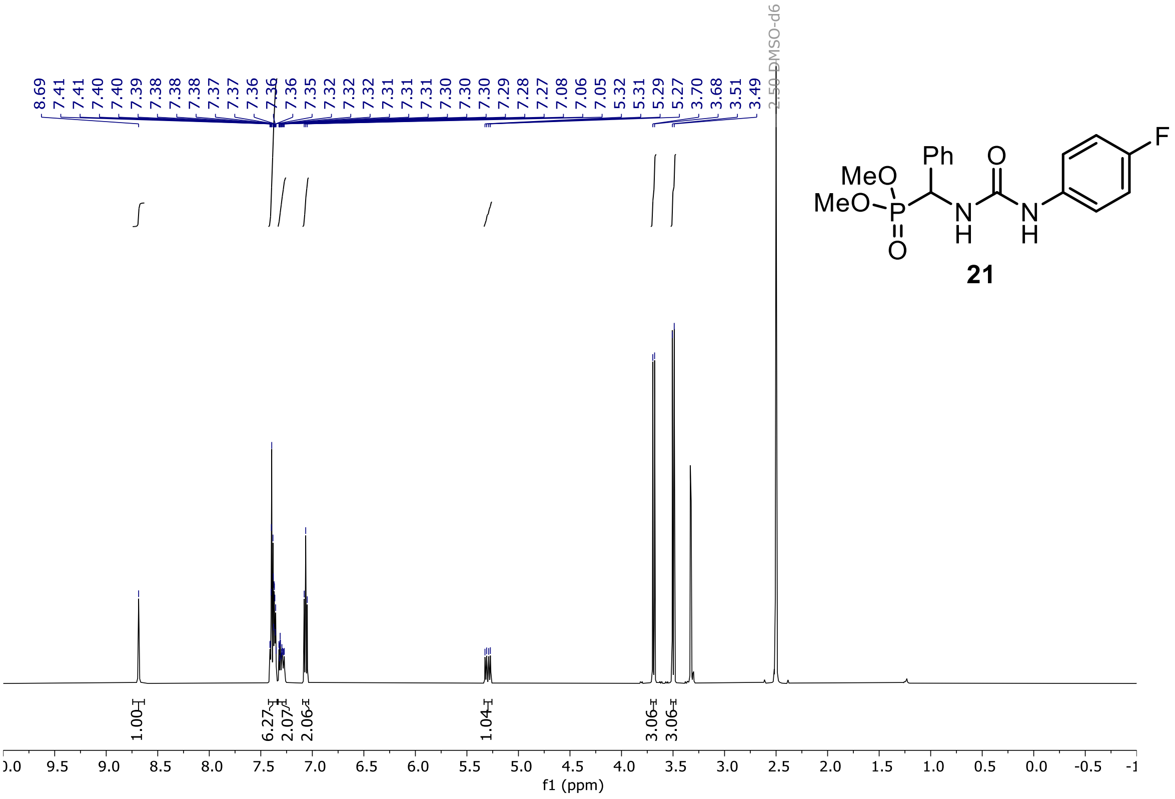

^{13}C-NMR (151 MHz, DMSO-d_6)

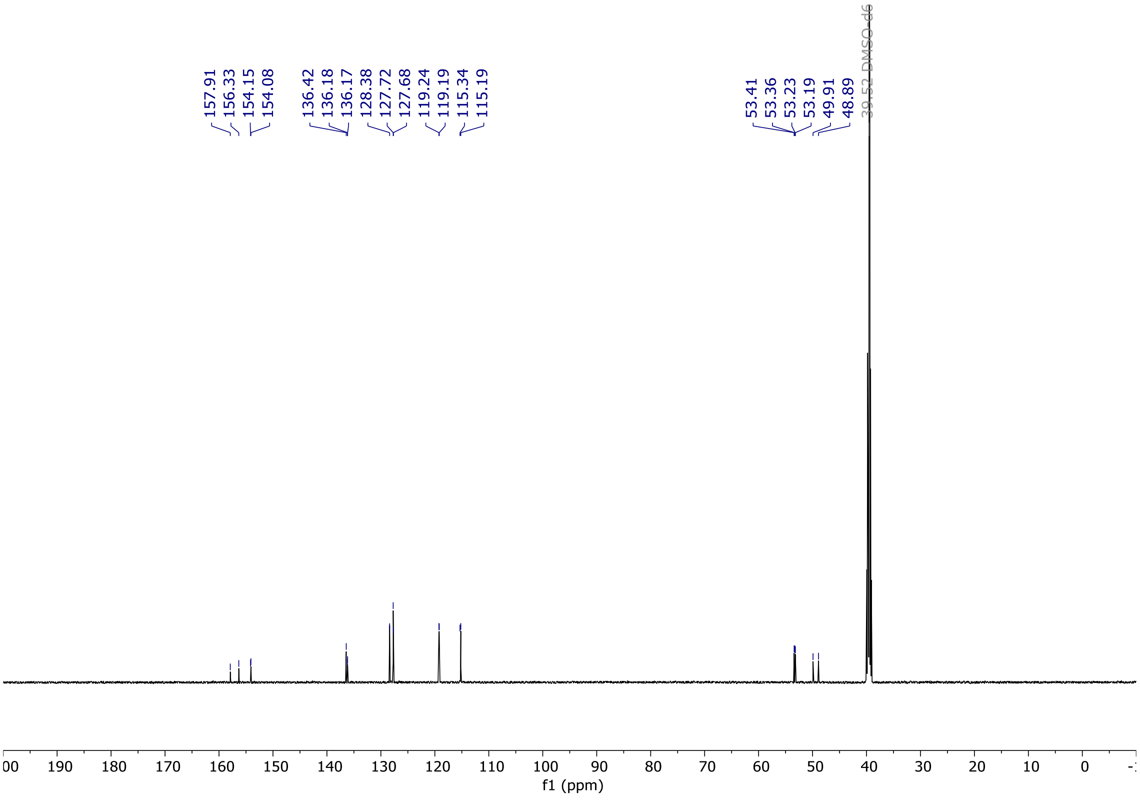

^{31}P-NMR (243 MHz, DMSO-d_6)

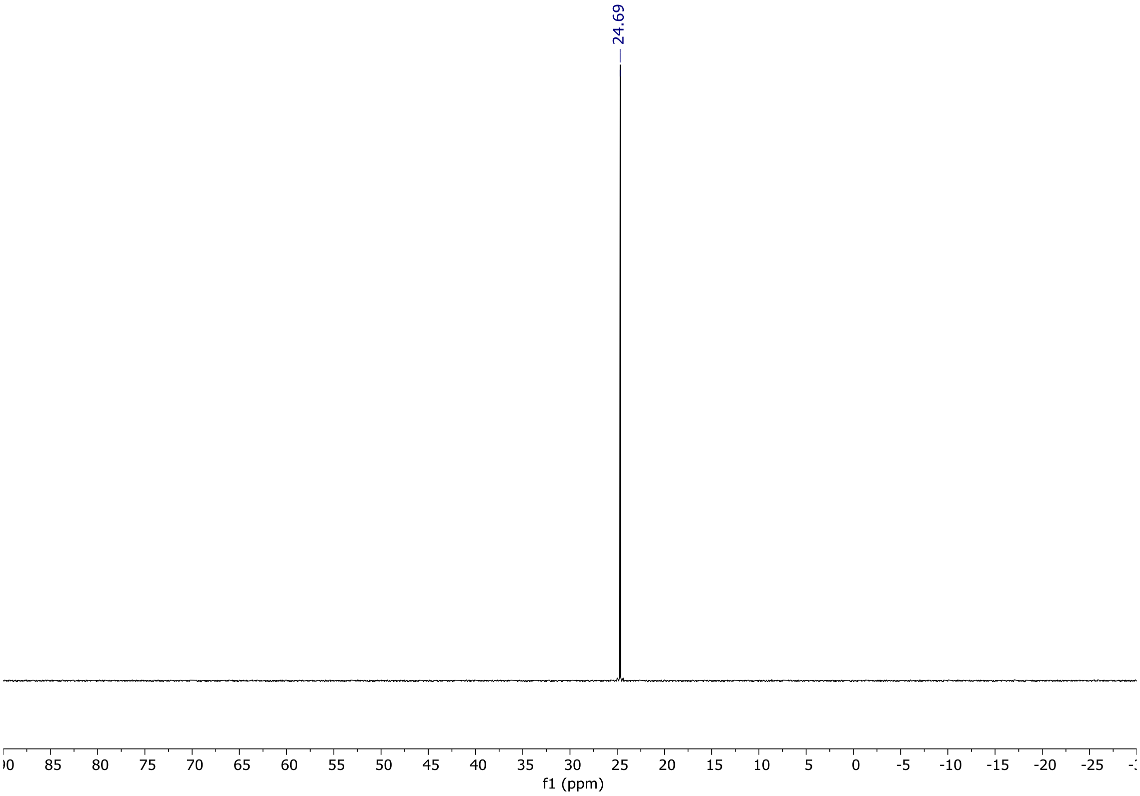

^{19}F-NMR (565 MHz, DMSO-d_6)

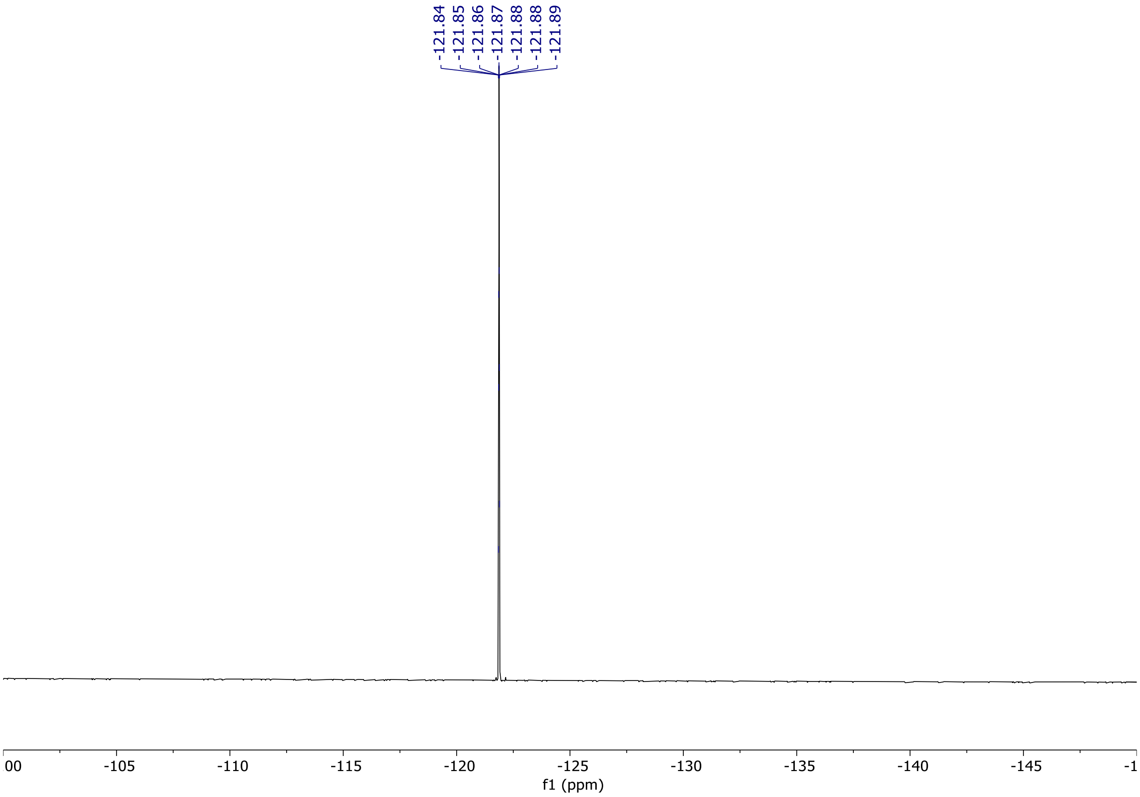

^{1}H-NMR (600 MHz, DMSO-d_6)

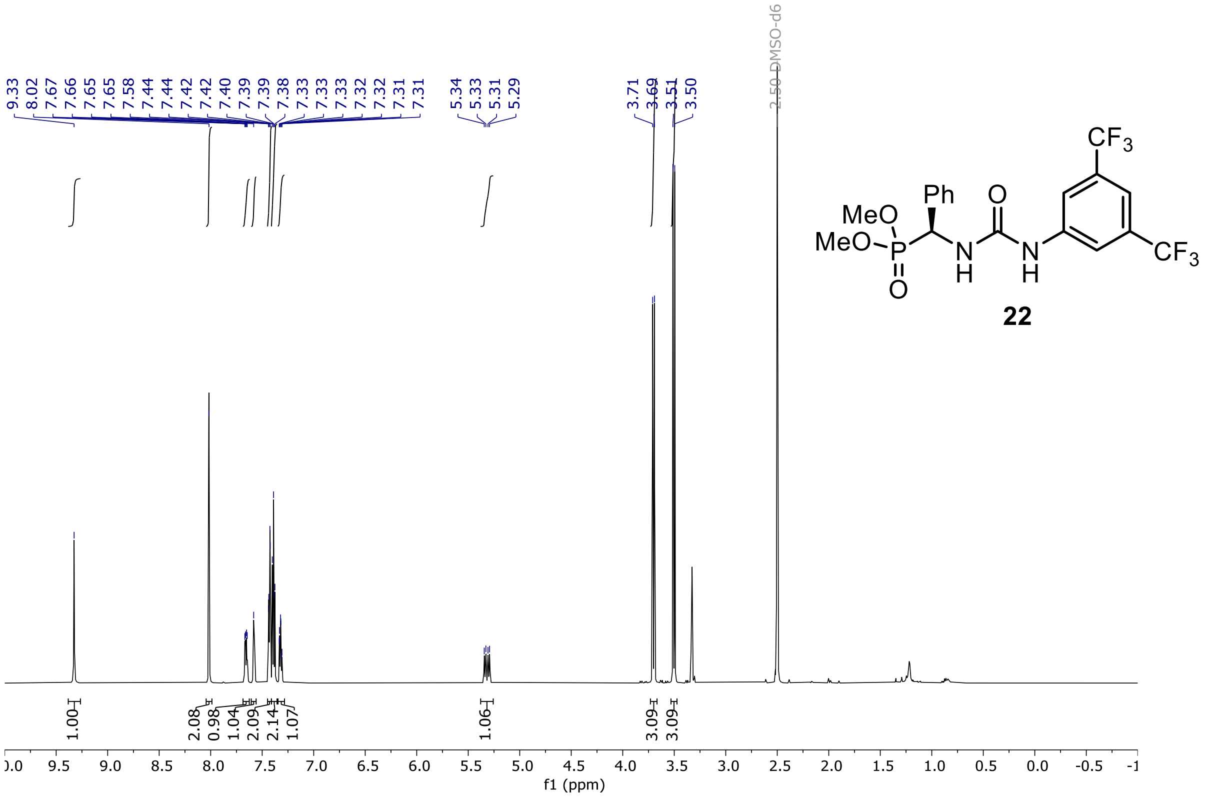

^{13}C-NMR (151 MHz, DMSO-d_6)

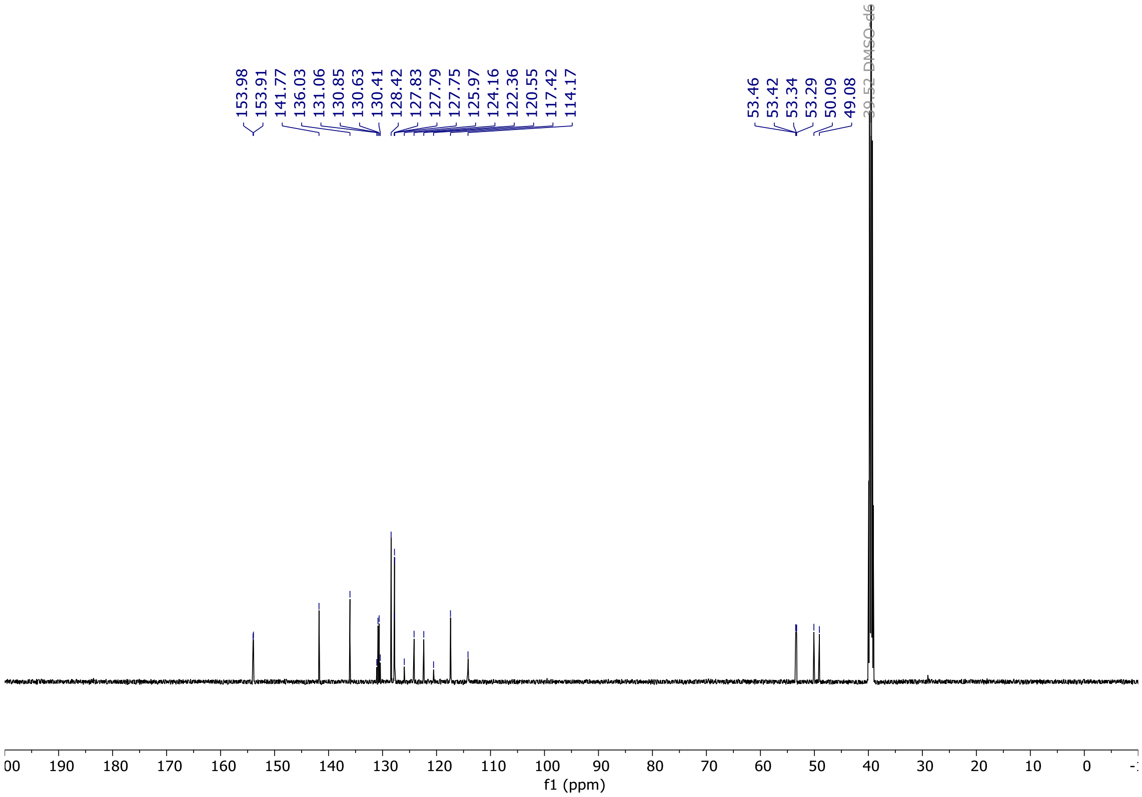

^{31}P-NMR (243 MHz, DMSO-d_6)

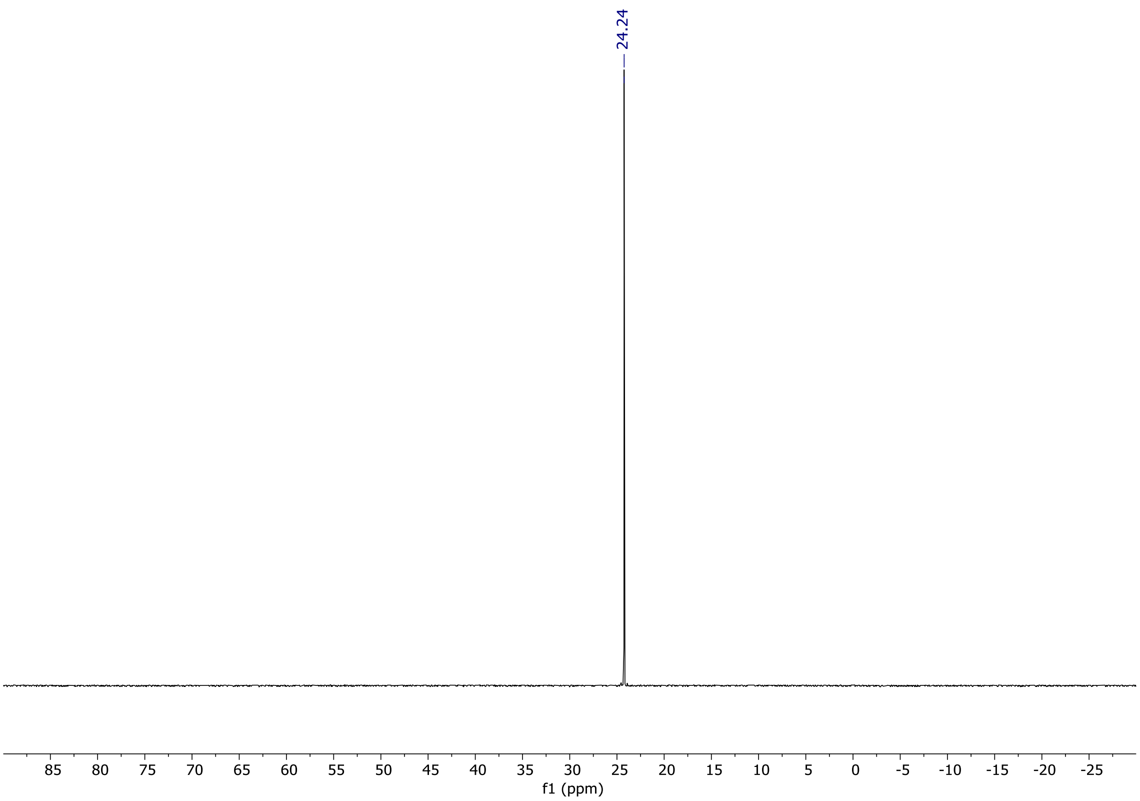

^{19}F-NMR (565 MHz, DMSO-d_6)

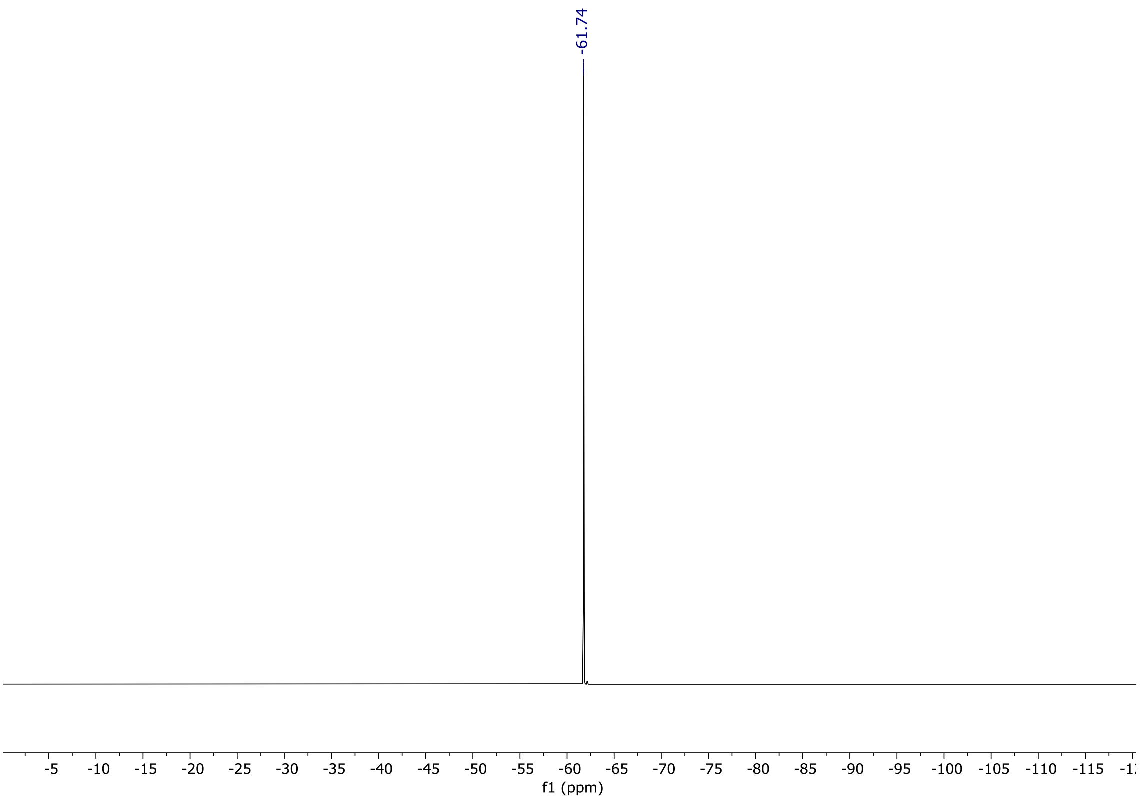

^{1}H-NMR (600 MHz, DMSO-*d*$_6$)

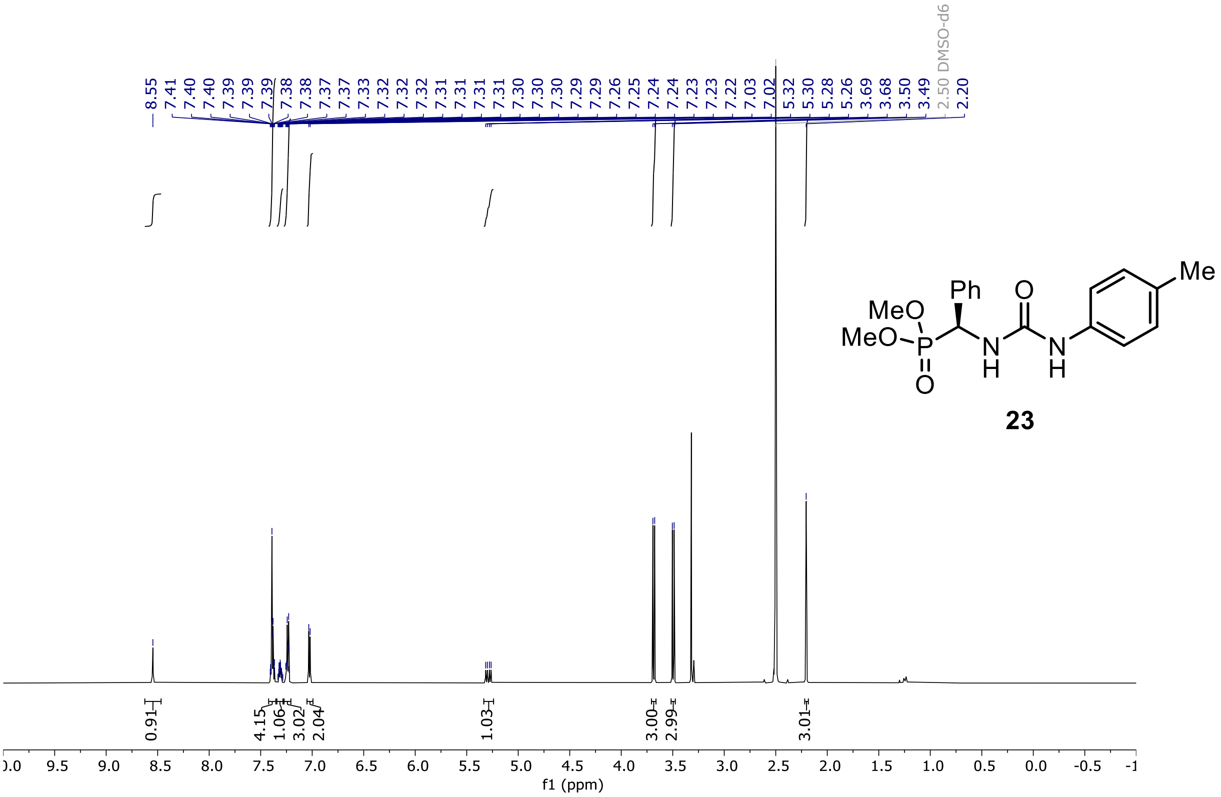

23

^{13}C-NMR (151 MHz, DMSO-*d*$_6$)

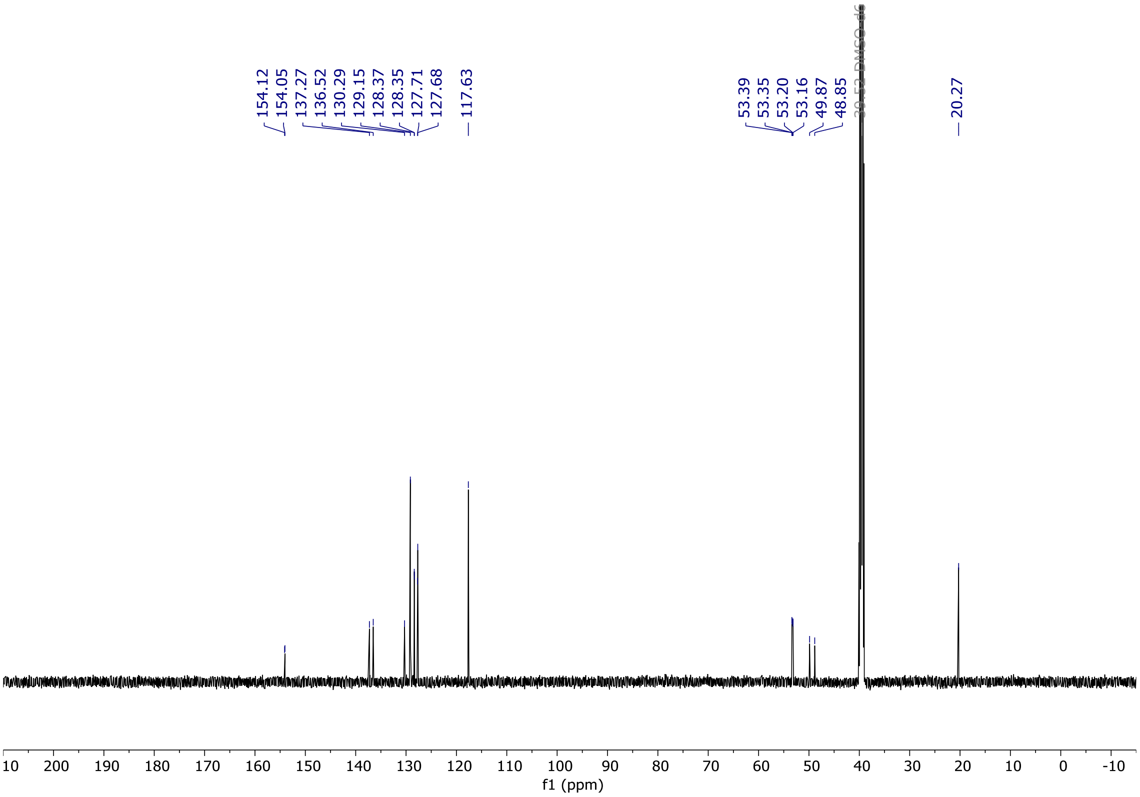

^{31}P-NMR (243 MHz, DMSO-d_6)

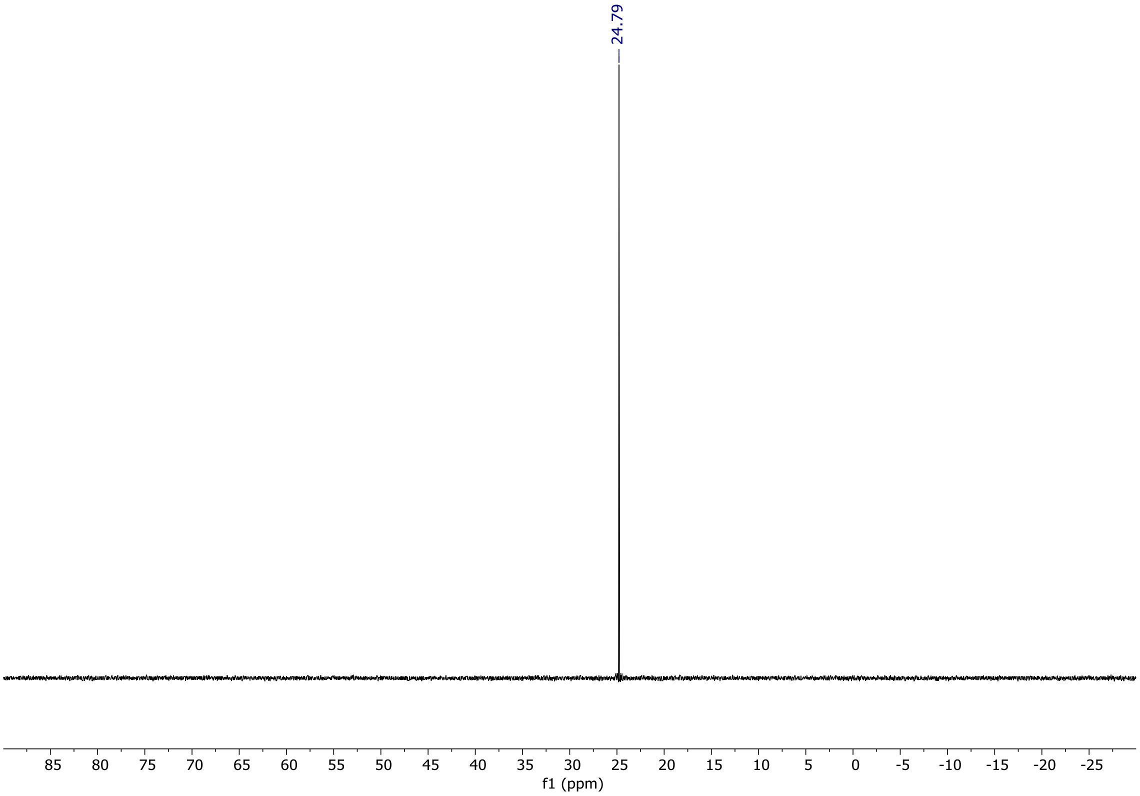

^{1}H-NMR (600 MHz, DMSO-d_6)

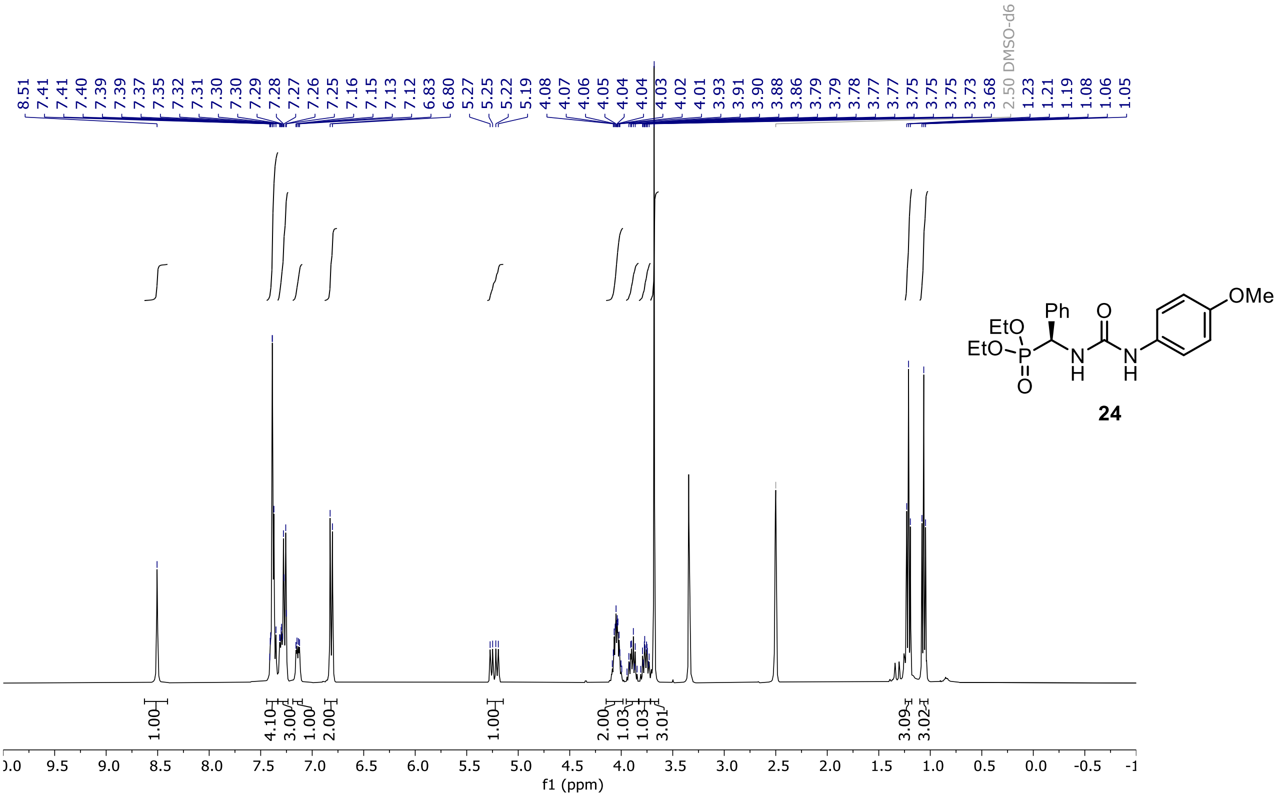

24

^{13}C-NMR (151 MHz, DMSO-d_6)

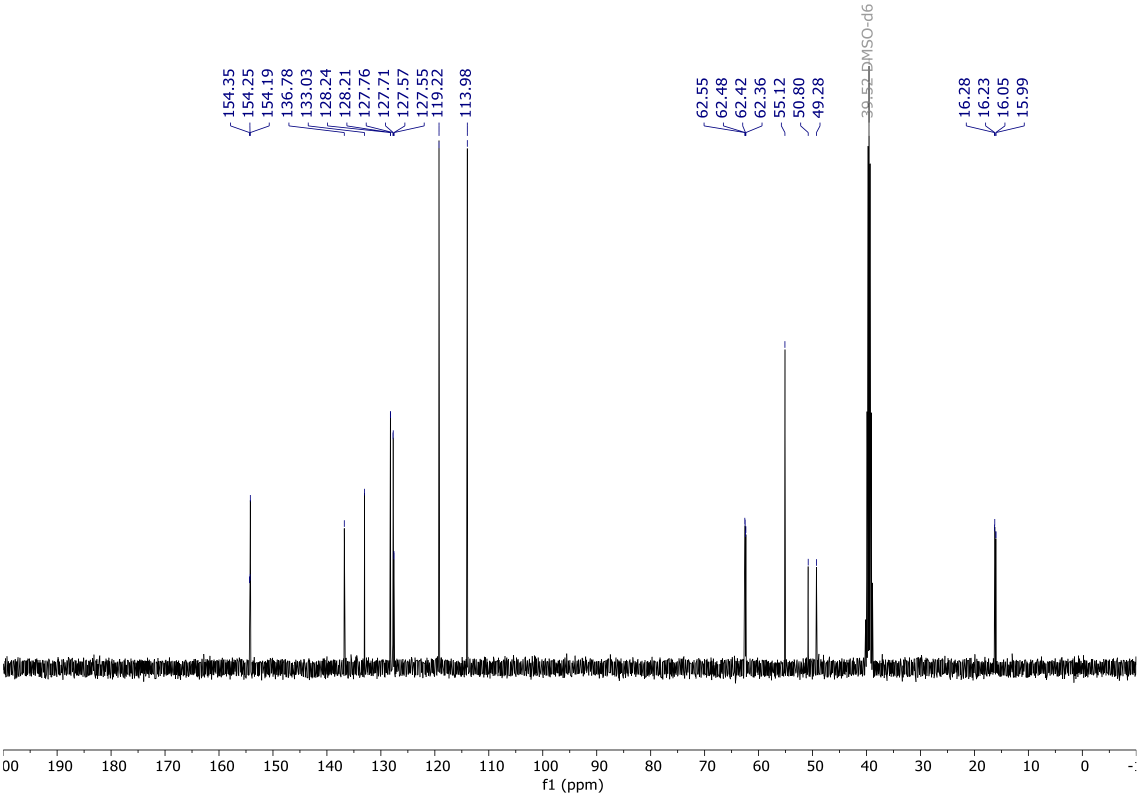

^{31}P-NMR (243 MHz, DMSO-d_6)

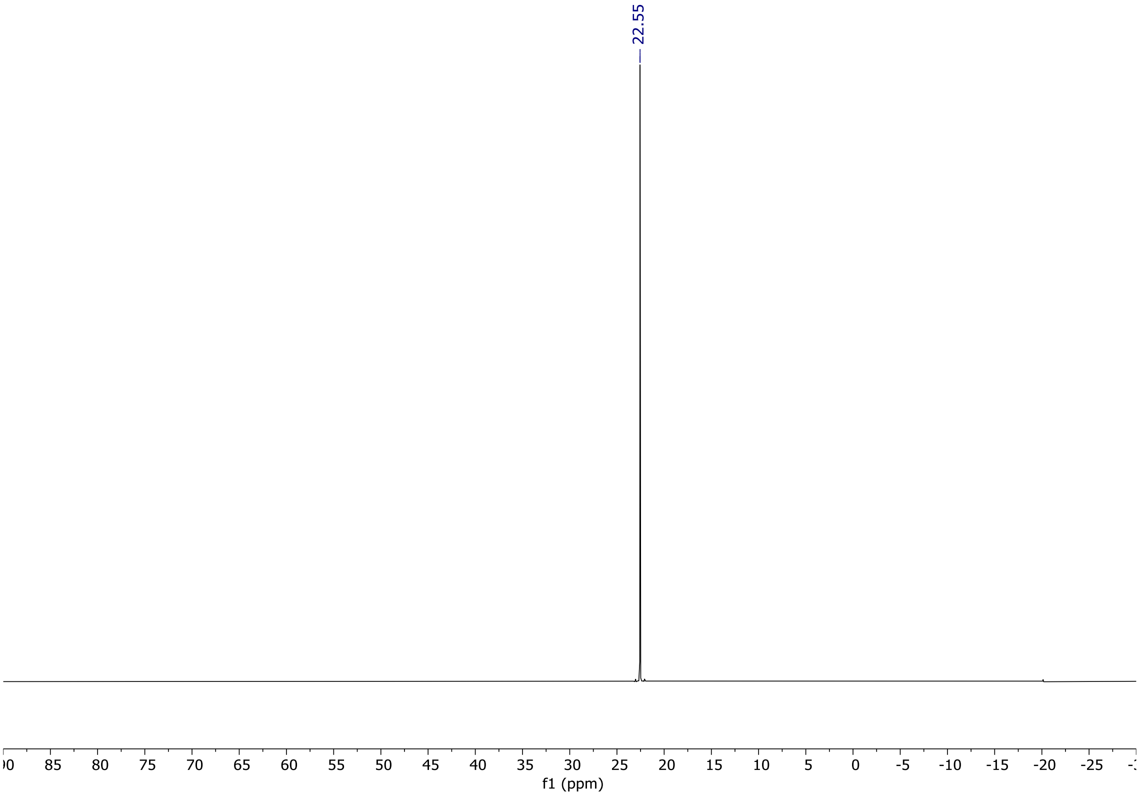

^{1}H-NMR (400 MHz, DMSO-d_6)

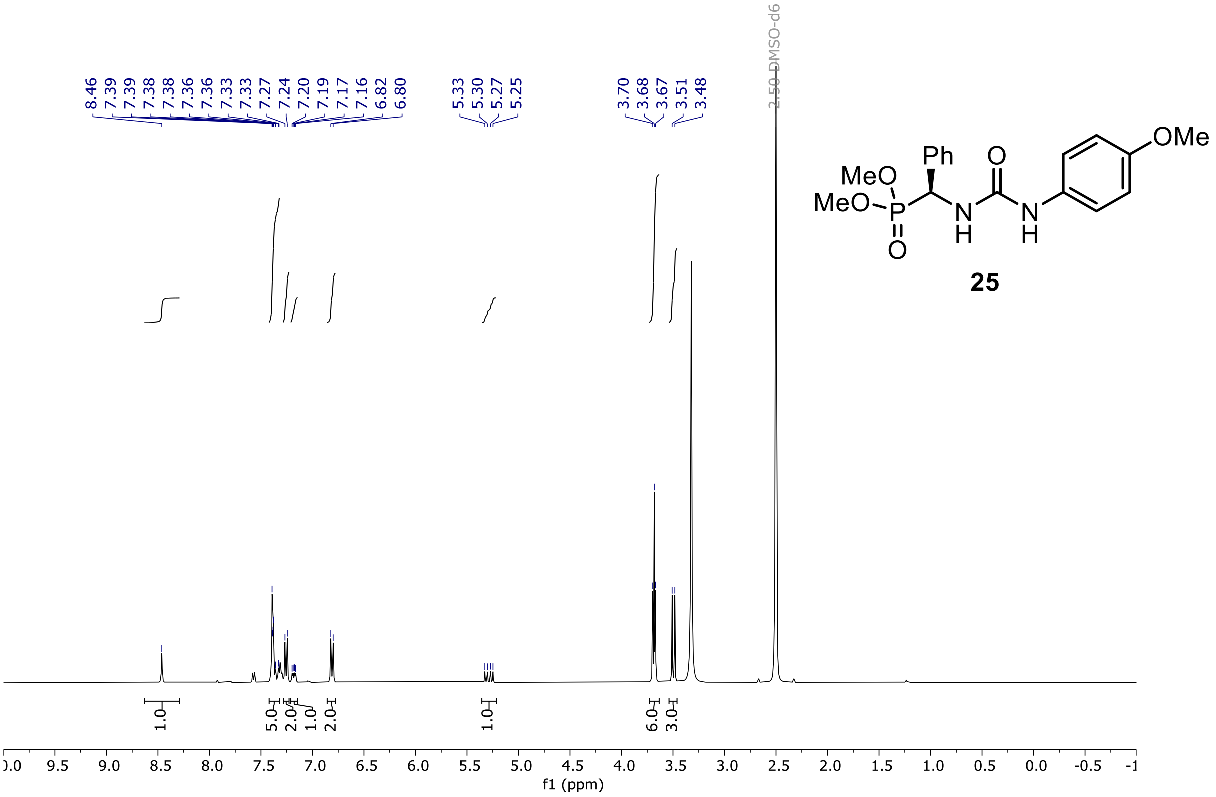

25

^{13}C-NMR (101 MHz, DMSO-d_6)

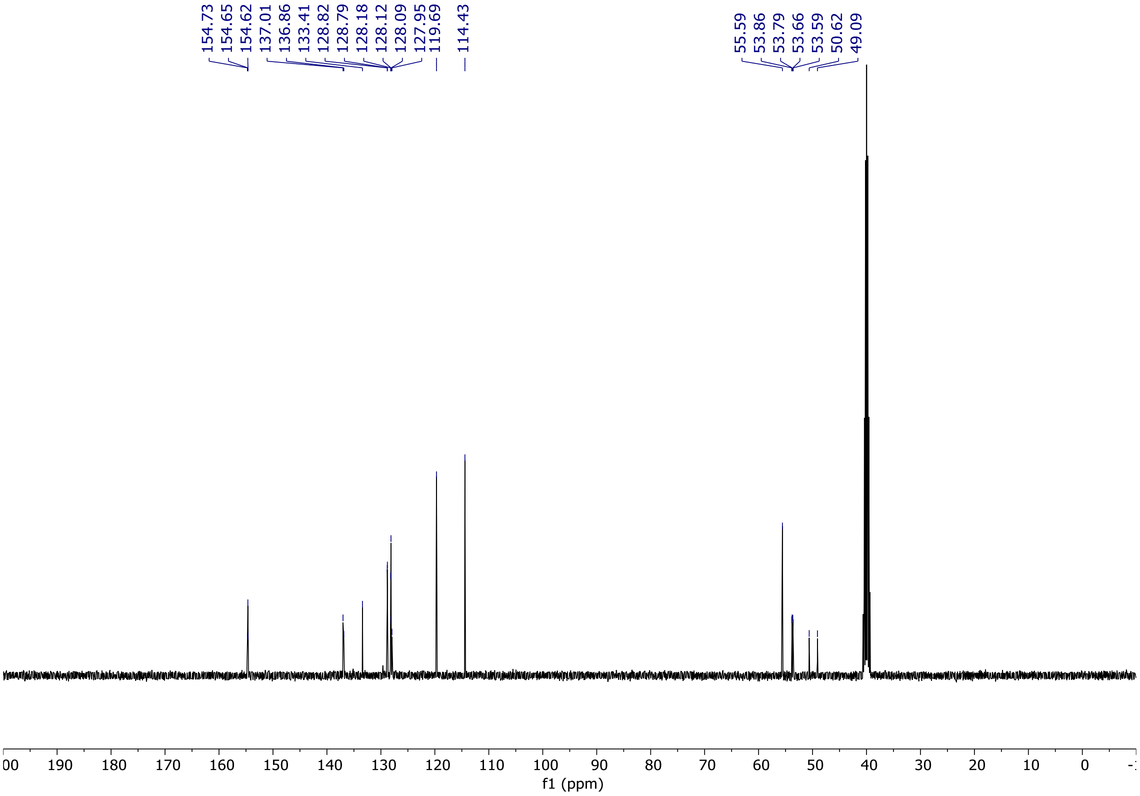

^{31}P-NMR (162 MHz, DMSO-d_6)

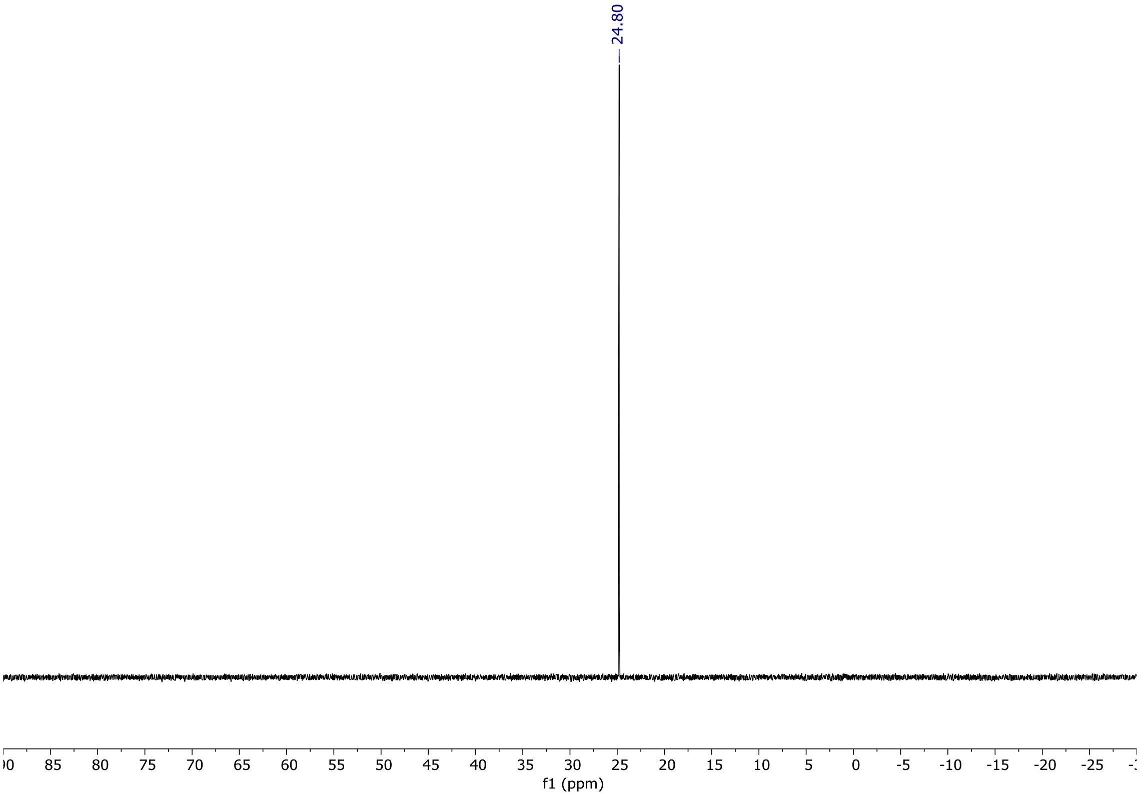

^{1}H-NMR (400 MHz, DMSO-d_6)

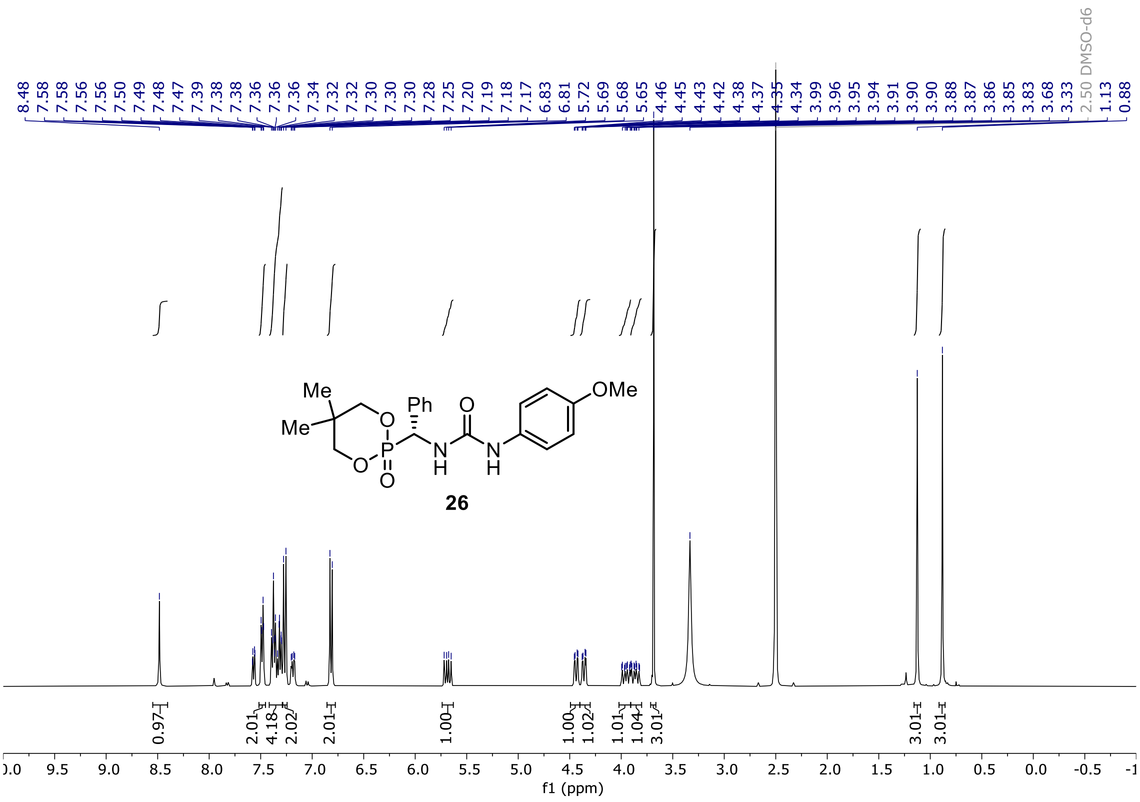

^{13}C-NMR (101 MHz, DMSO-d_6)

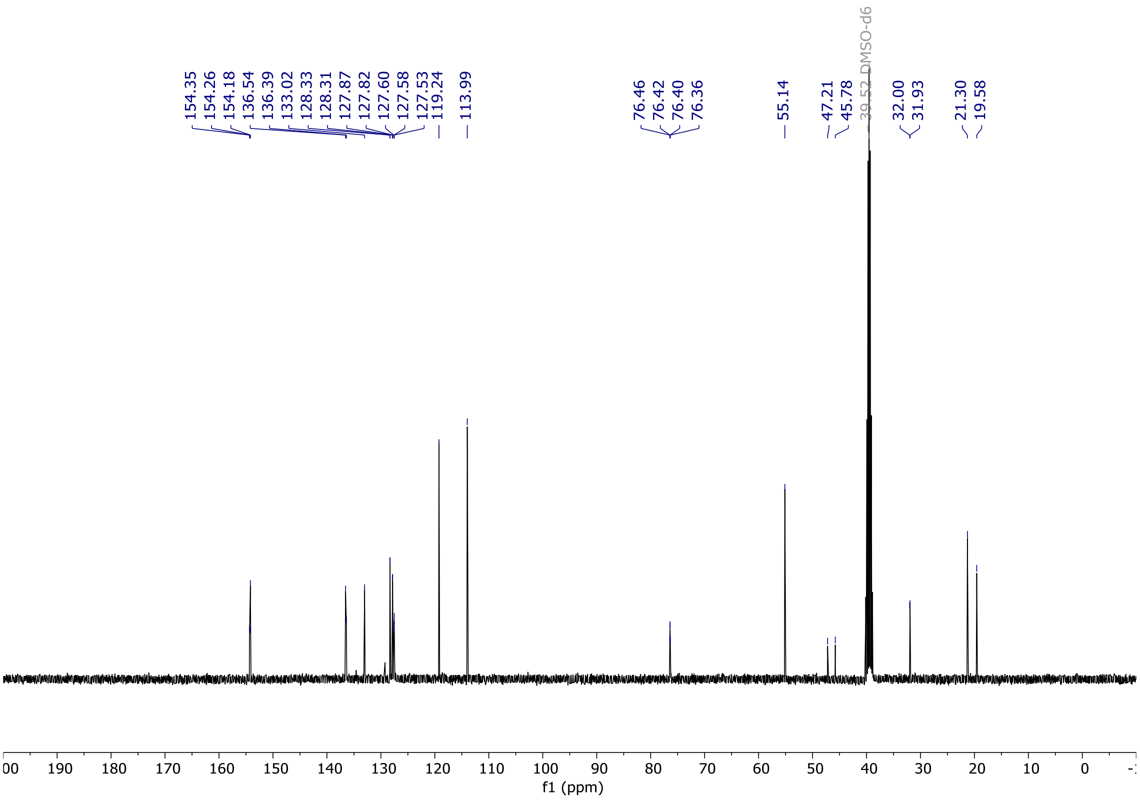

^{31}P-NMR (162 MHz, DMSO-d_6)

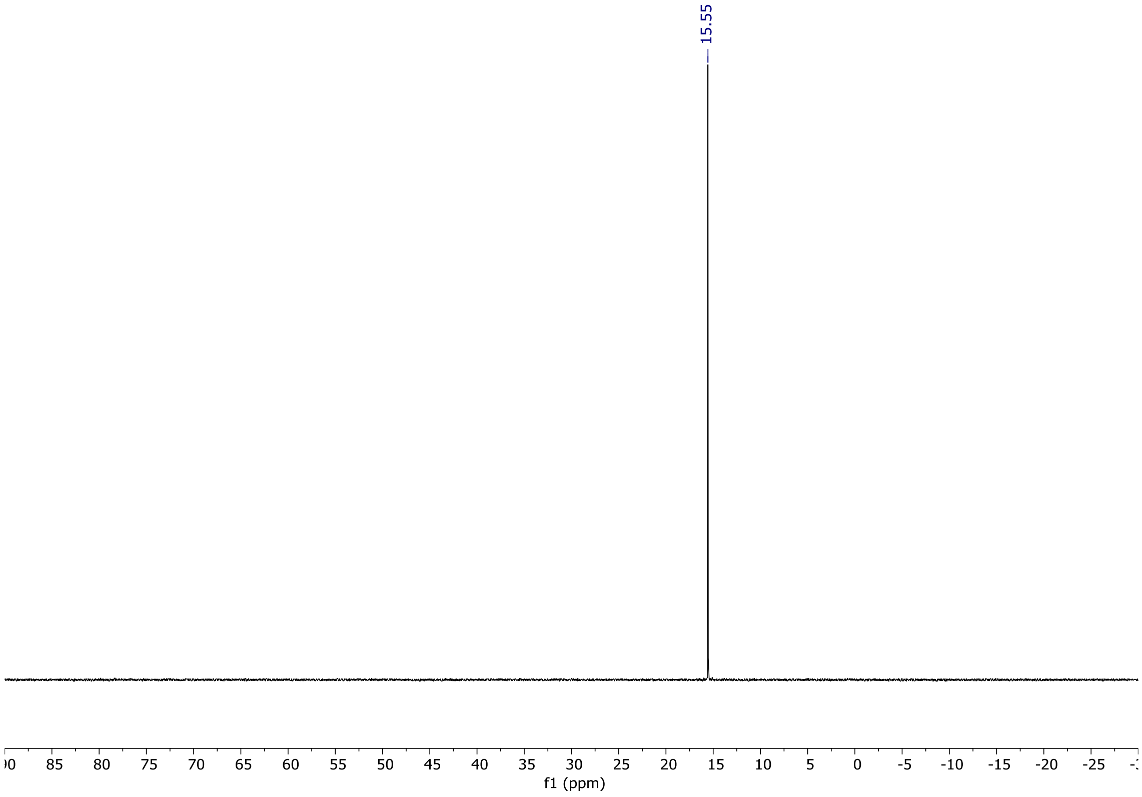

^{1}H-NMR (600 MHz, DMSO-d_6)

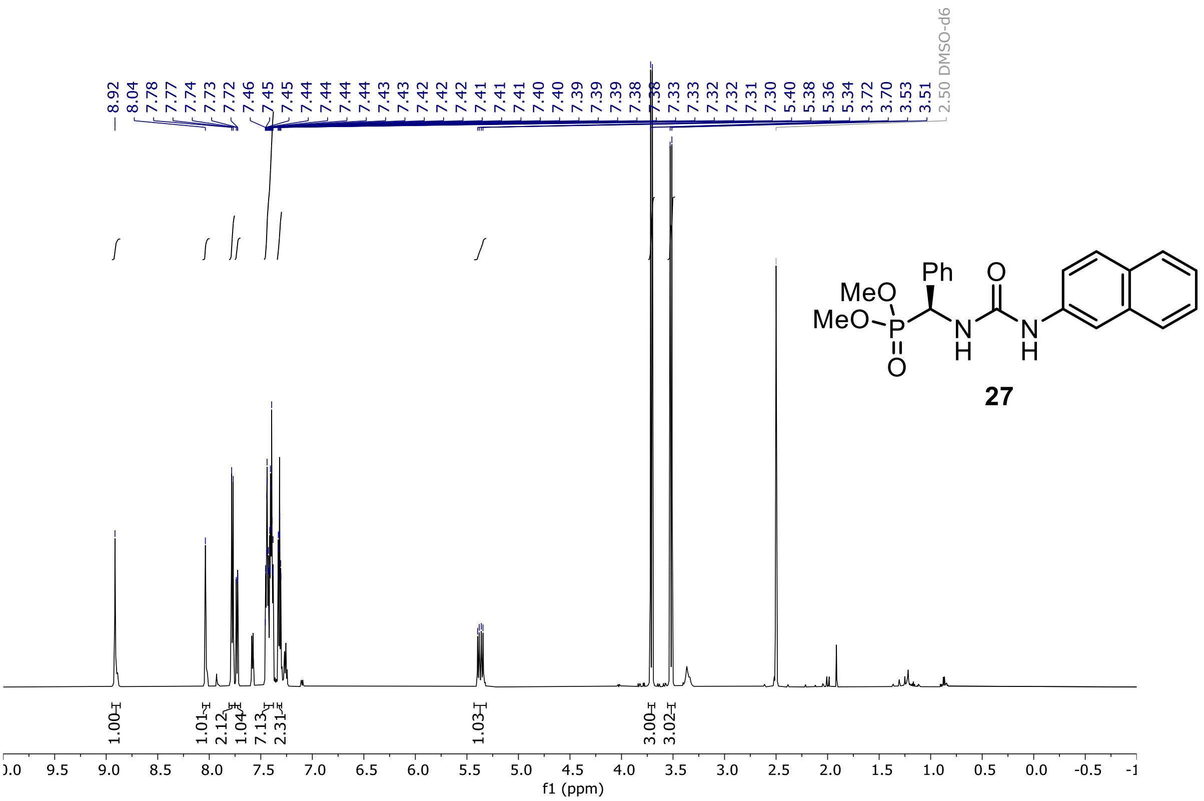

^{13}C-NMR (151 MHz, DMSO-d_6)

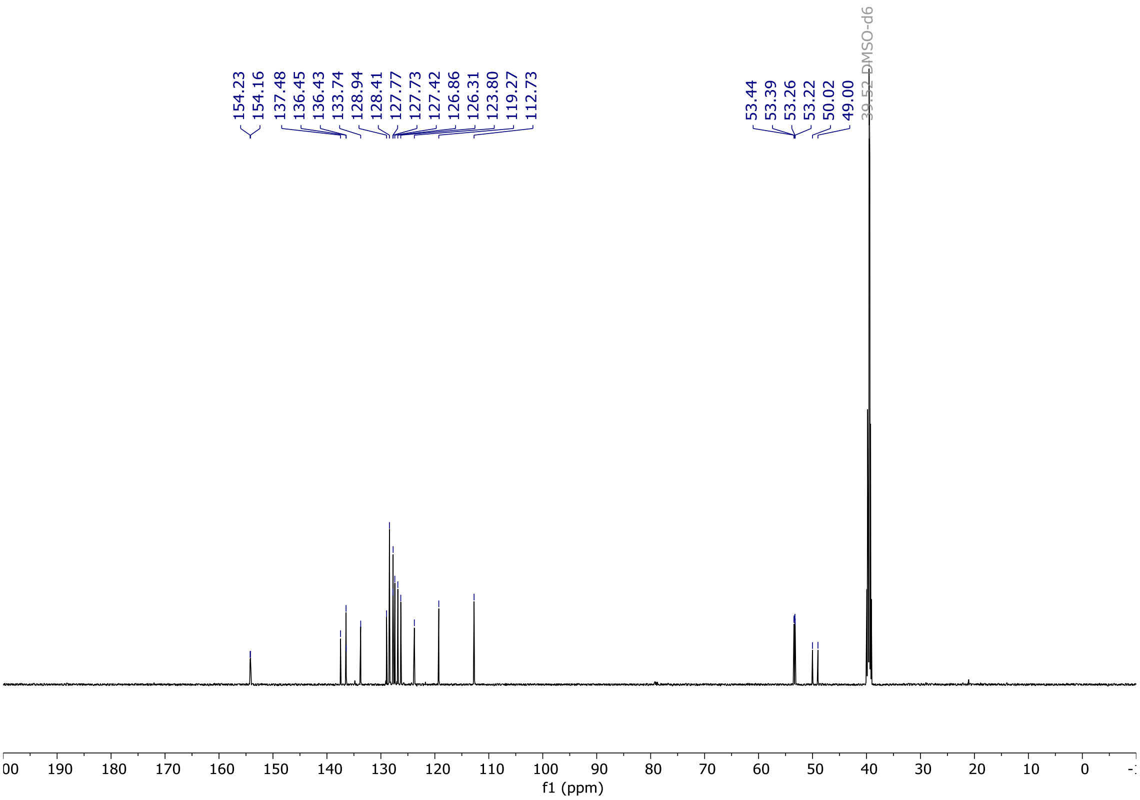

^{31}P-NMR (243 MHz, DMSO-d_6)

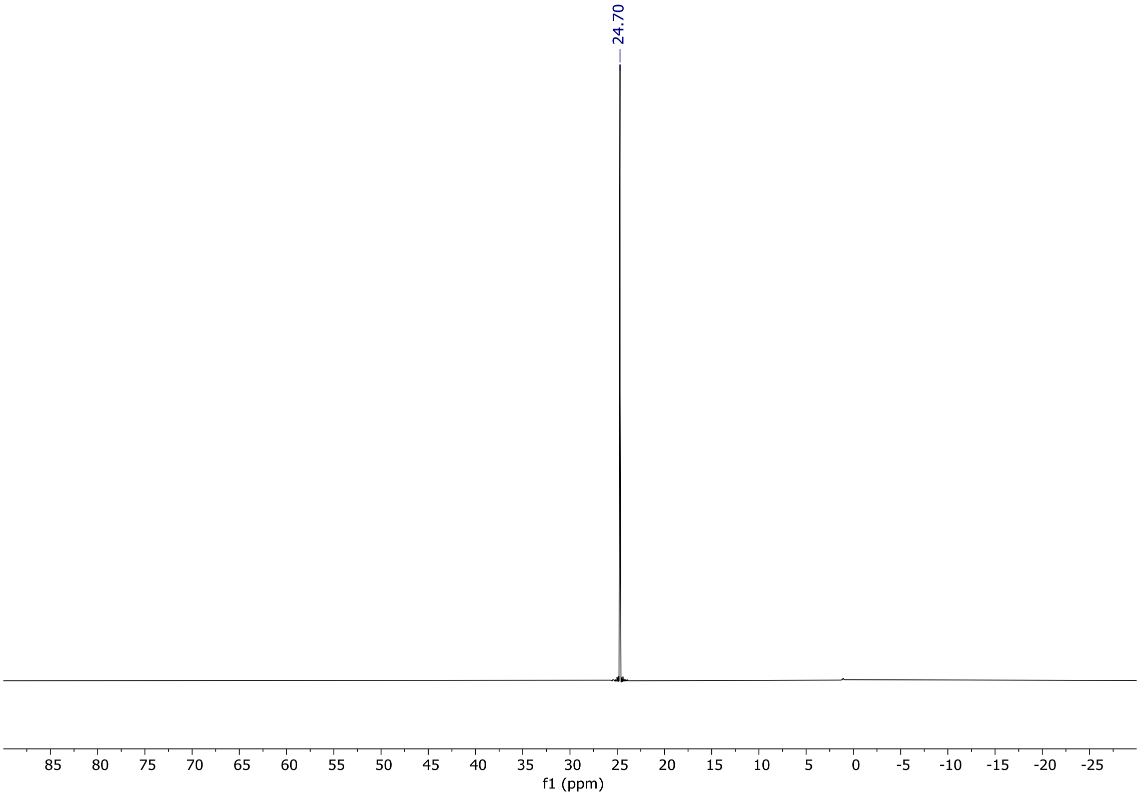

^{1}H-NMR (600 MHz, DMSO-d_6)

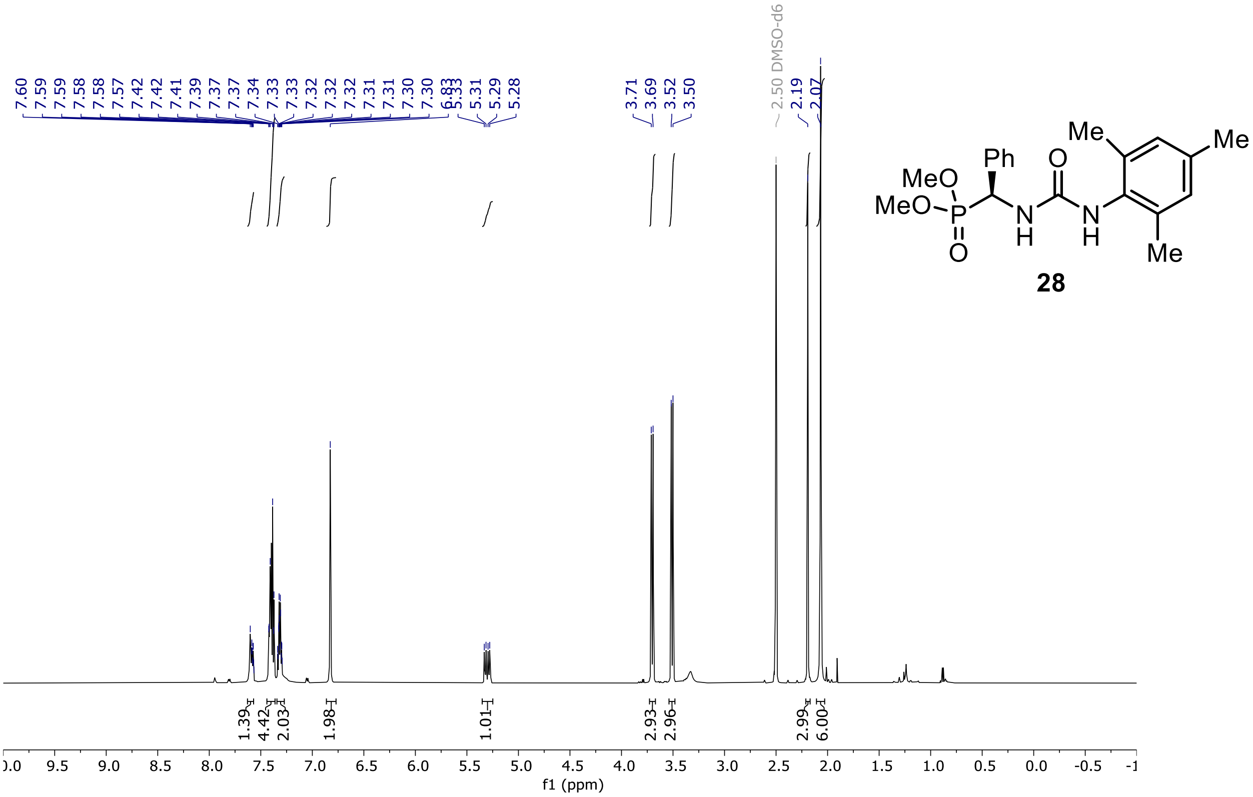

^{13}C-NMR (151 MHz, DMSO-d_6)

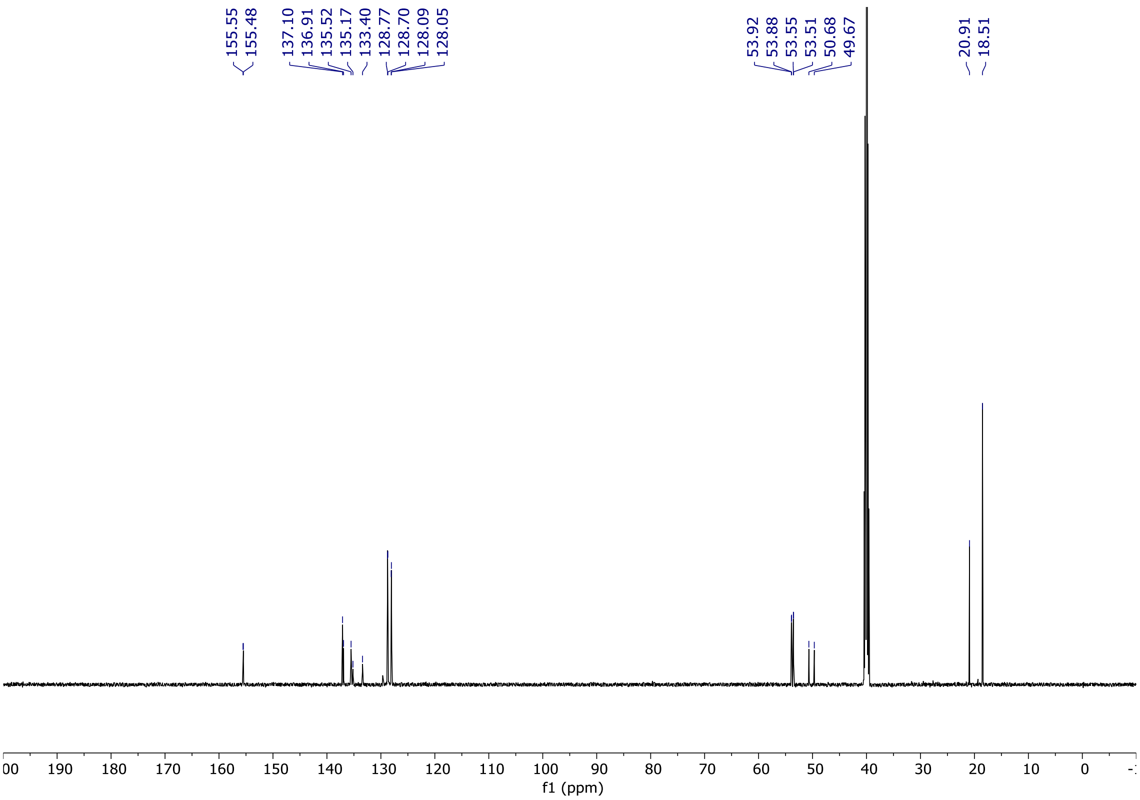

^{31}P-NMR (162 MHz, DMSO-d_6)

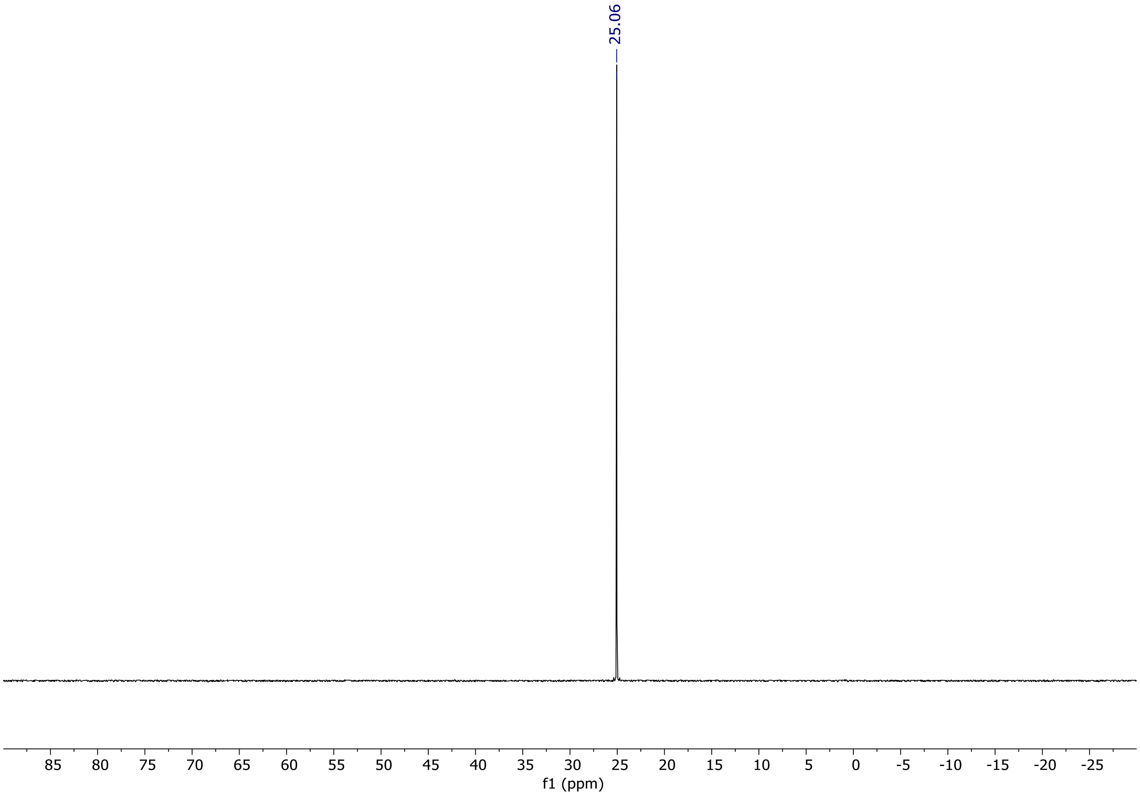

11. *e.r.* determination by NMR and/or HPLC

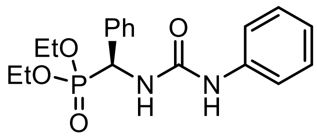

3

^{31}P-NMR (162 MHz, $CDCl_3$)

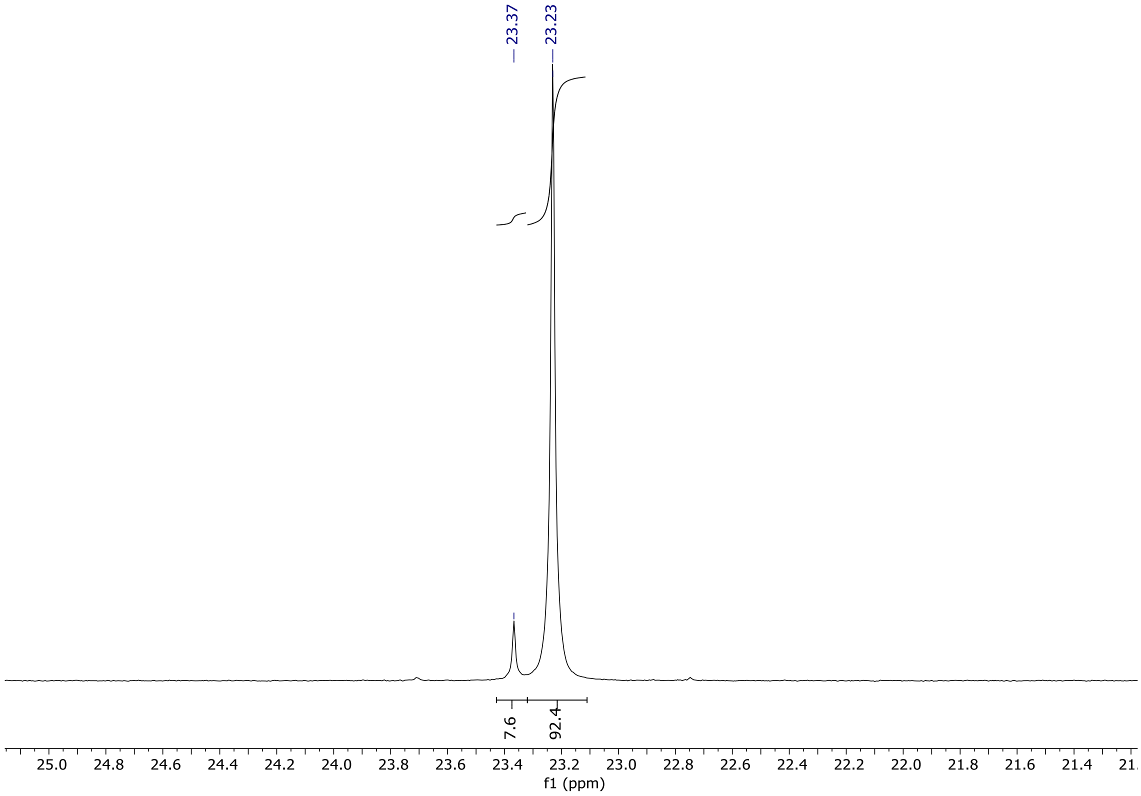

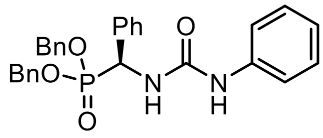

4

^{31}P-NMR (162 MHz, $CDCl_3$)

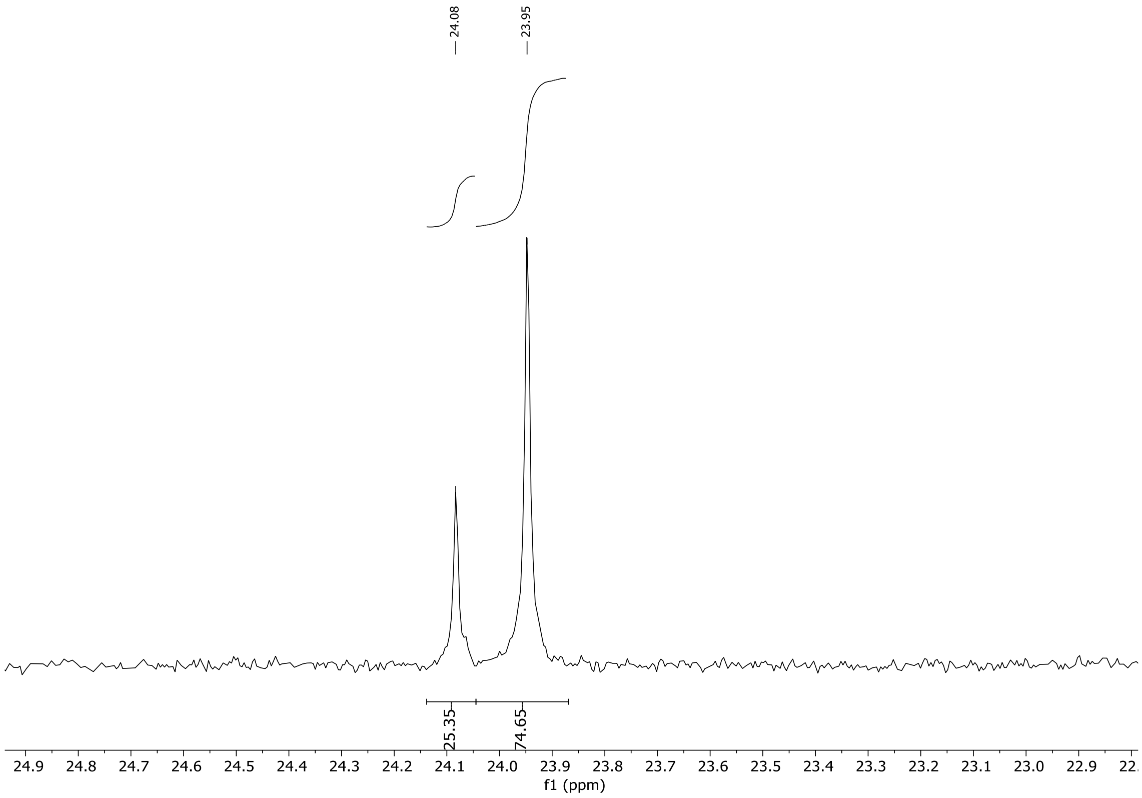

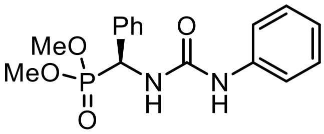

5

^{31}P-NMR (162 MHz, $CDCl_3$)

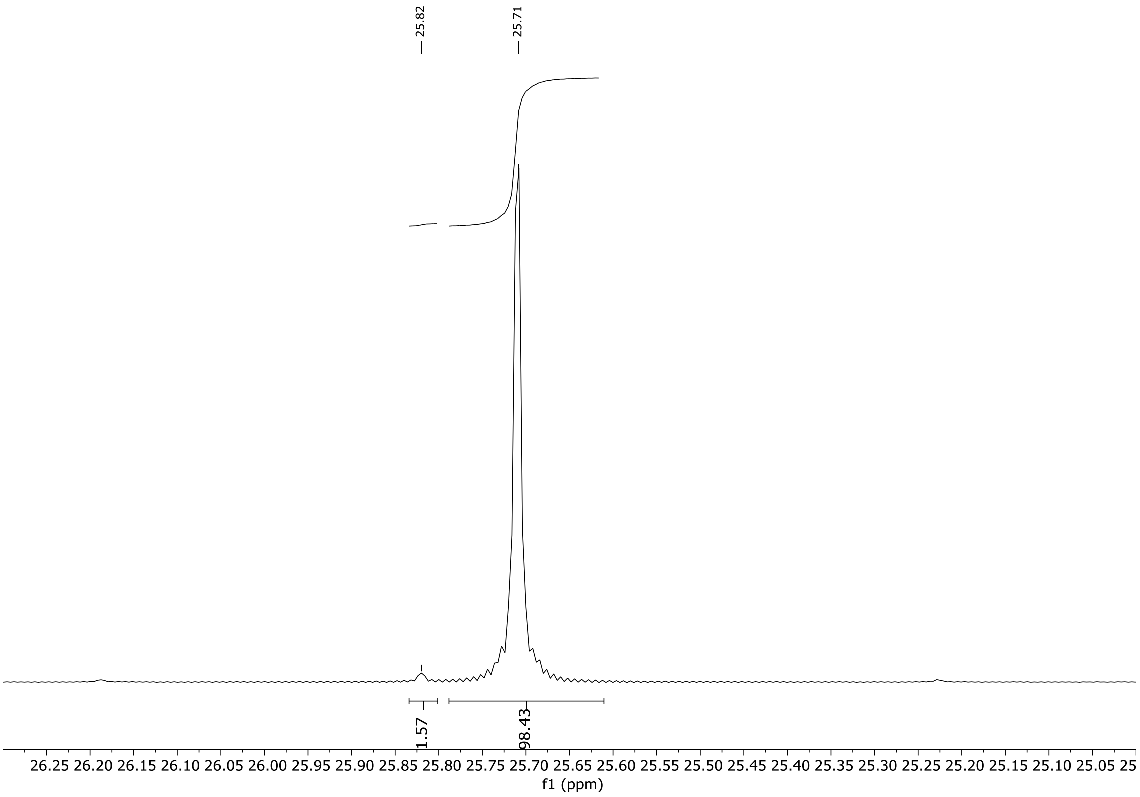

SFC chromatogram of racemic 5 (obtained by hydrophosphonylation of arylidene urea **6** with dimethyl phosphite in absence of chiral catalyst)

Conditions: Chiralpak ID, ^{i}PrOH 1-40%, 20 min, 4 mL/min, λ_{abs} = 240 nm, t_R(*R*) = 13.8 min, t_R(*S*) = 14.9 min

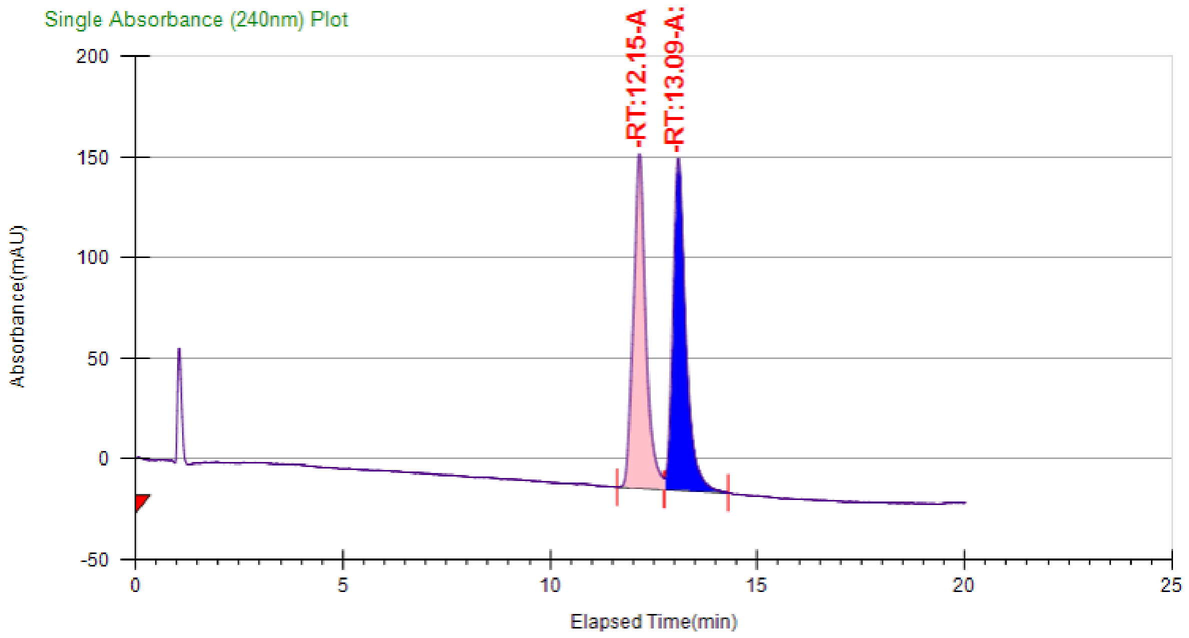

Peak Information

Peak No	% Area	Area	Ret. Time
1	49.2377	3463.9398	12.15 min
2	50.7623	3571.202	13.09 min

SFC chromatogram of enantioenriched (*S*)-5 (obtained by purification of crude material of α-ureidophosphonate **5** by column chromatography, see Section 6.1)

Conditions: Chiralpak ID, ^{i}PrOH 1-40%, 20 min, 4 mL/min, λ_{abs} = 240 nm, $t_R(R)$ = 13.8 min, $t_R(S)$ = 14.9 min

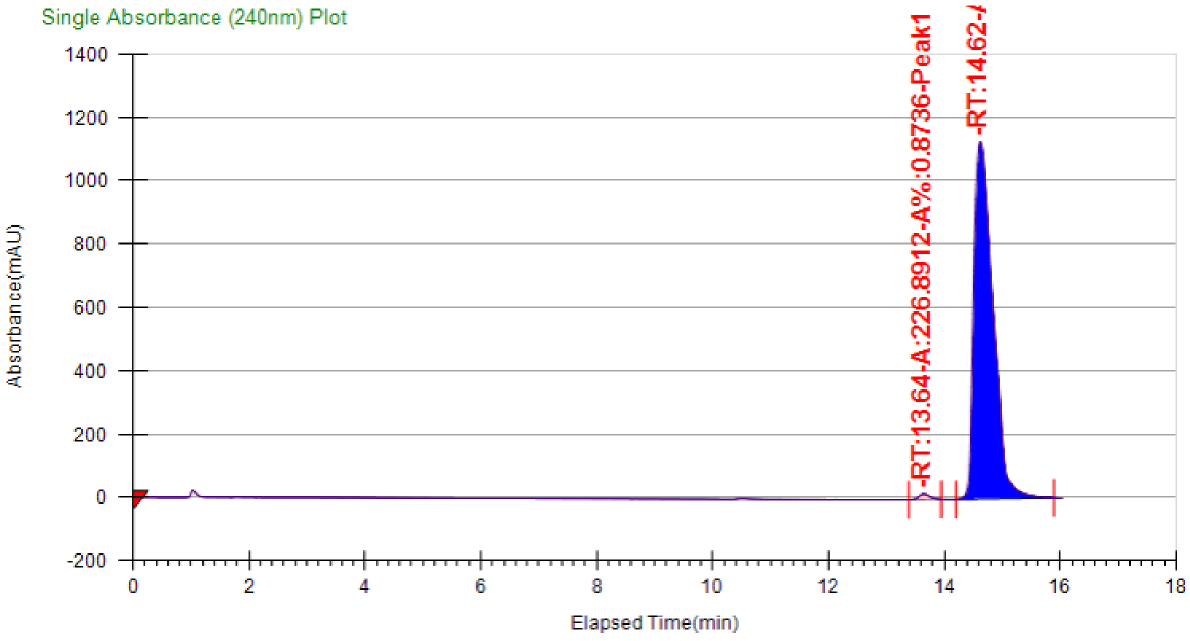

Peak Information

Peak No	% Area	Area	Ret. Time
1	0.8736	226.8912	13.64 min
2	99.1264	25745.759<br>2	14.62 min

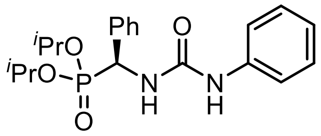

7

^{31}P-NMR (162 MHz, $CDCl_3$)

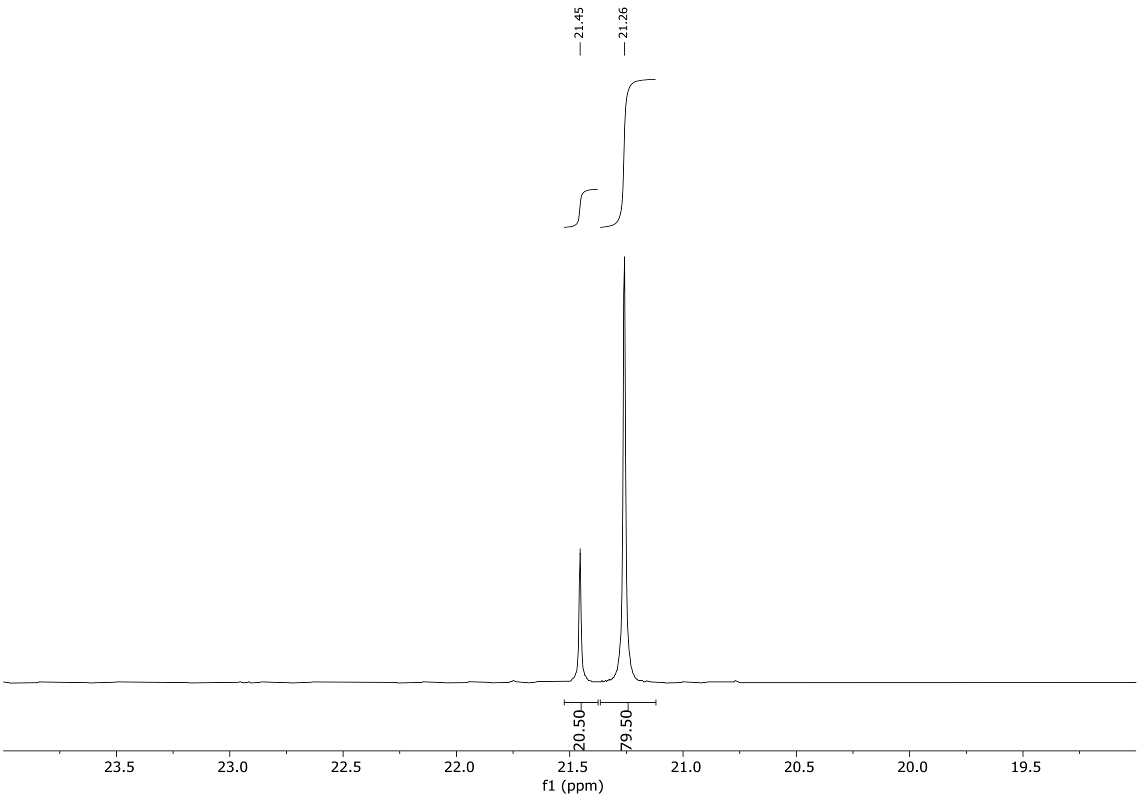

HPLC chromatogram of racemic 7 (obtained by purification of crude material of α-ureidophosphonate **7** by column chromatography, see Section 6.1)

Conditions: Lux Cellulose-4, *n*-heptane/^{i}PrOH 90:10, 40 °C, 1 mL/min, λ_{abs} = 240 nm, $t_R(S)$ = 7.8 min, $t_R(R)$ = 10.3 min.

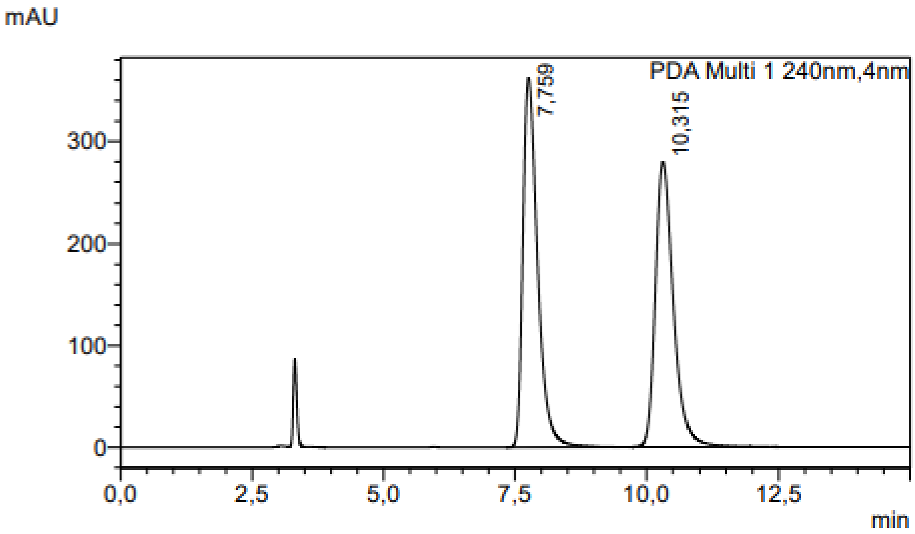

Peak Table

PDA Ch1 240nm

Peak#	Ret. Time	Area%
1	7,759	51,433
2	10,315	48,567
Total		100,000

HPLC chromatogram of enantioenriched (*S*)-7

Conditions: Lux Cellulose-4, *n*-heptane/^{i}PrOH 90:10, 40 °C, 1 mL/min, λ_{abs} = 240 nm, $t_R(S)$ = 8.2 min, $t_R(R)$ = 11.1 min.

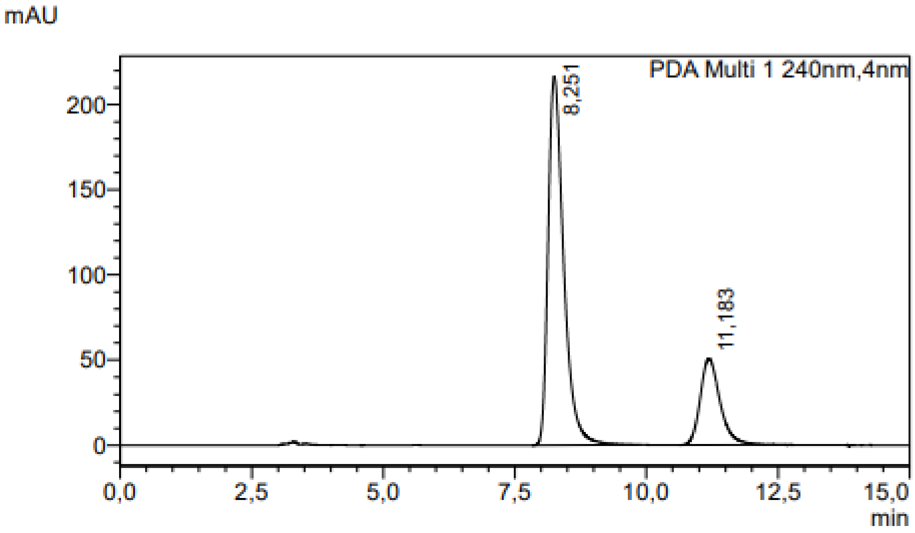

Peak Table

PDA Ch1 240nm

Peak#	Ret. Time	Area%
1	8,251	76,991
2	11,183	23,009
Total		100,000

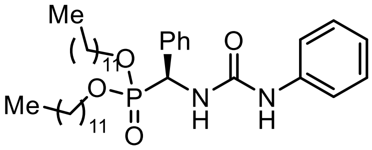

8

^{31}P-NMR (162 MHz, $CDCl_3$)

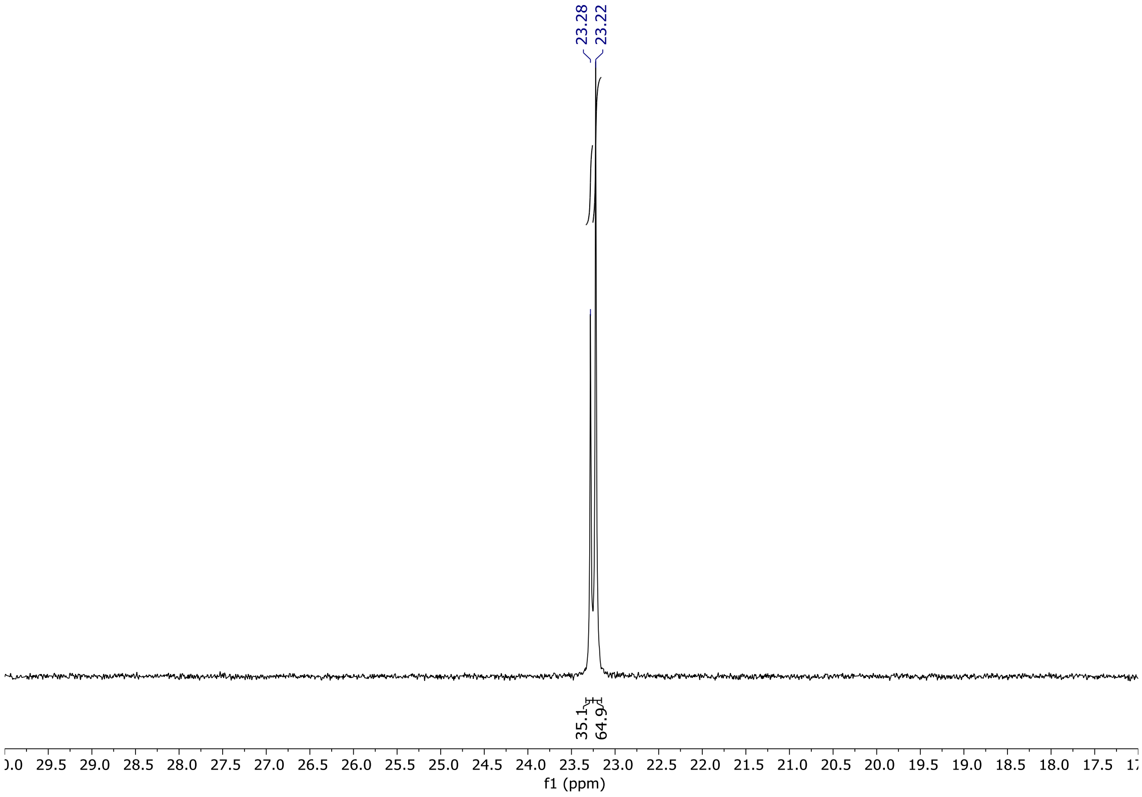

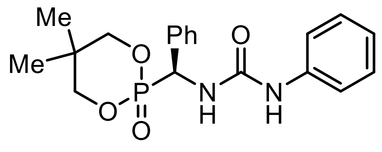

9

^{31}P-NMR (162 MHz, CDCl$_3$)

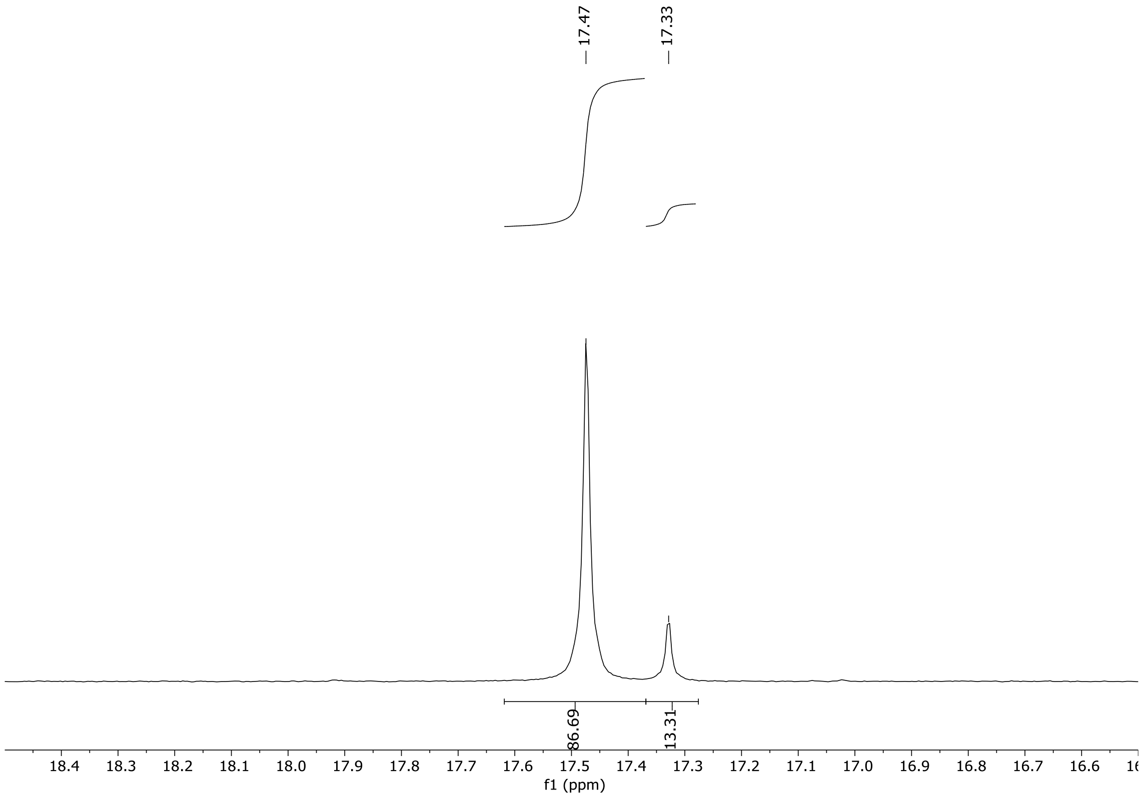

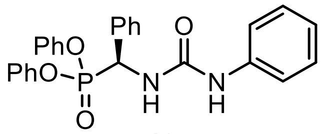

10

^{31}P-NMR (162 MHz, $CDCl_3$)

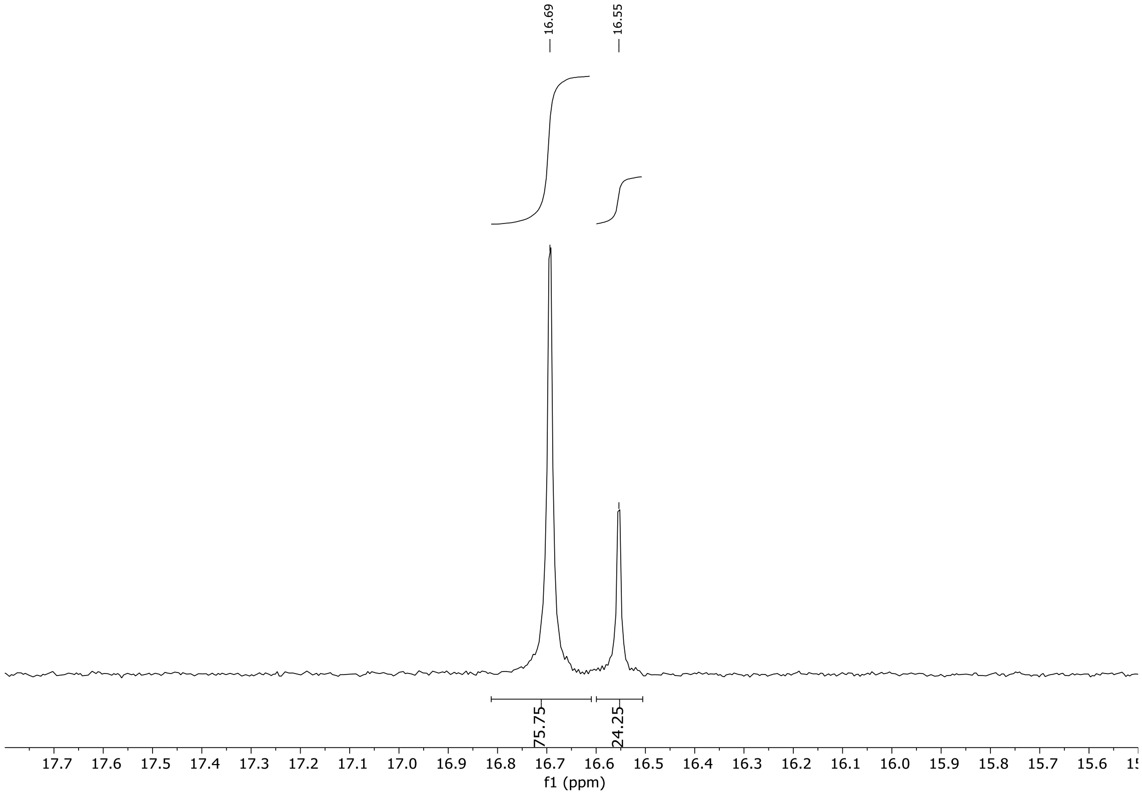

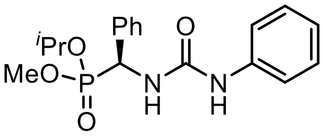

11

(1:1 mixture of diastereomers)

^{31}P-NMR (162 MHz, $CDCl_3$)

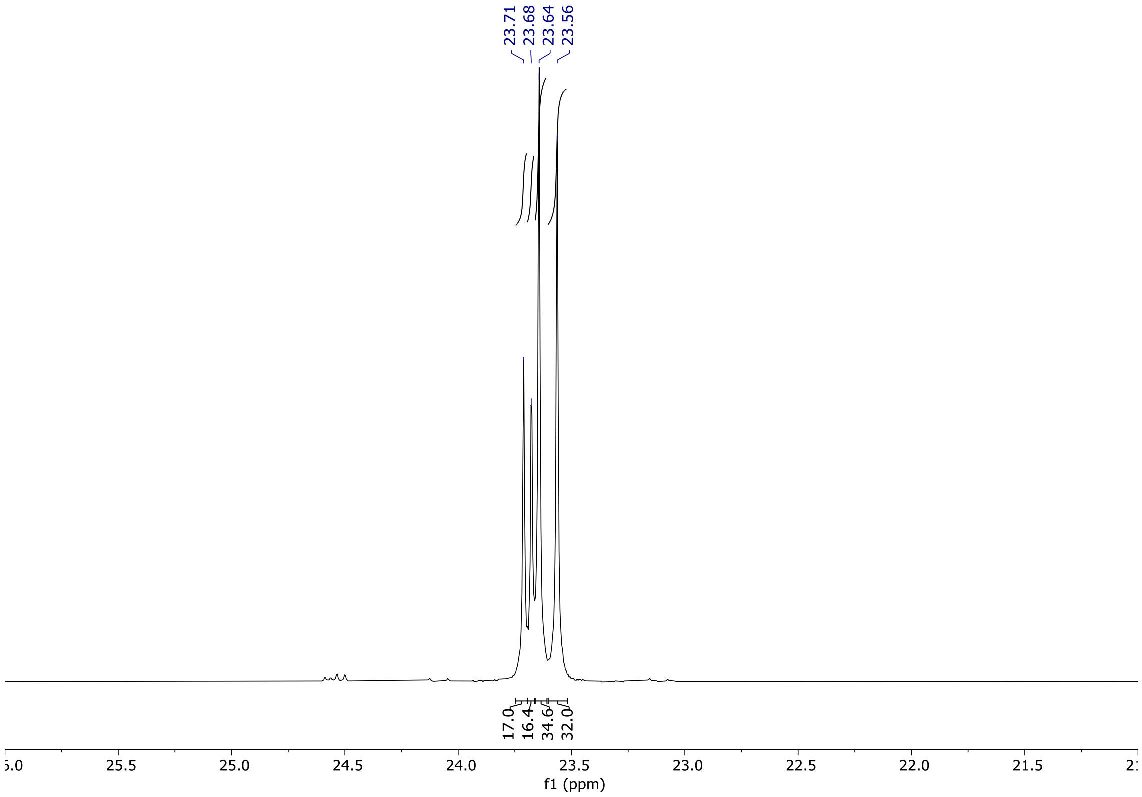

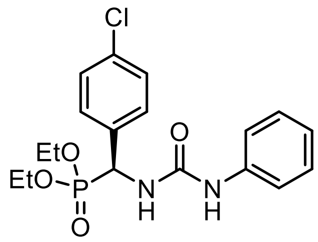

12

^{31}P-NMR (162 MHz, CDCl$_3$)

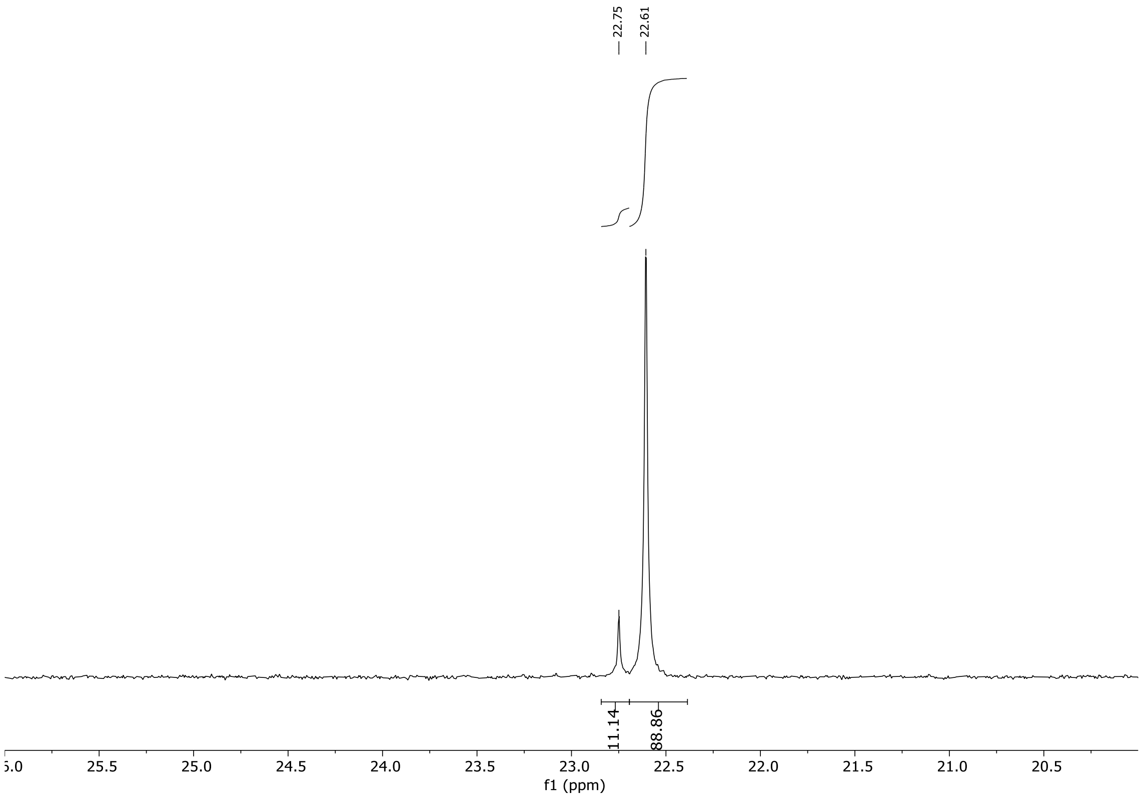

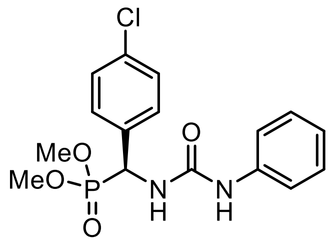

13

^{31}P-NMR (162 MHz, $CDCl_3$)

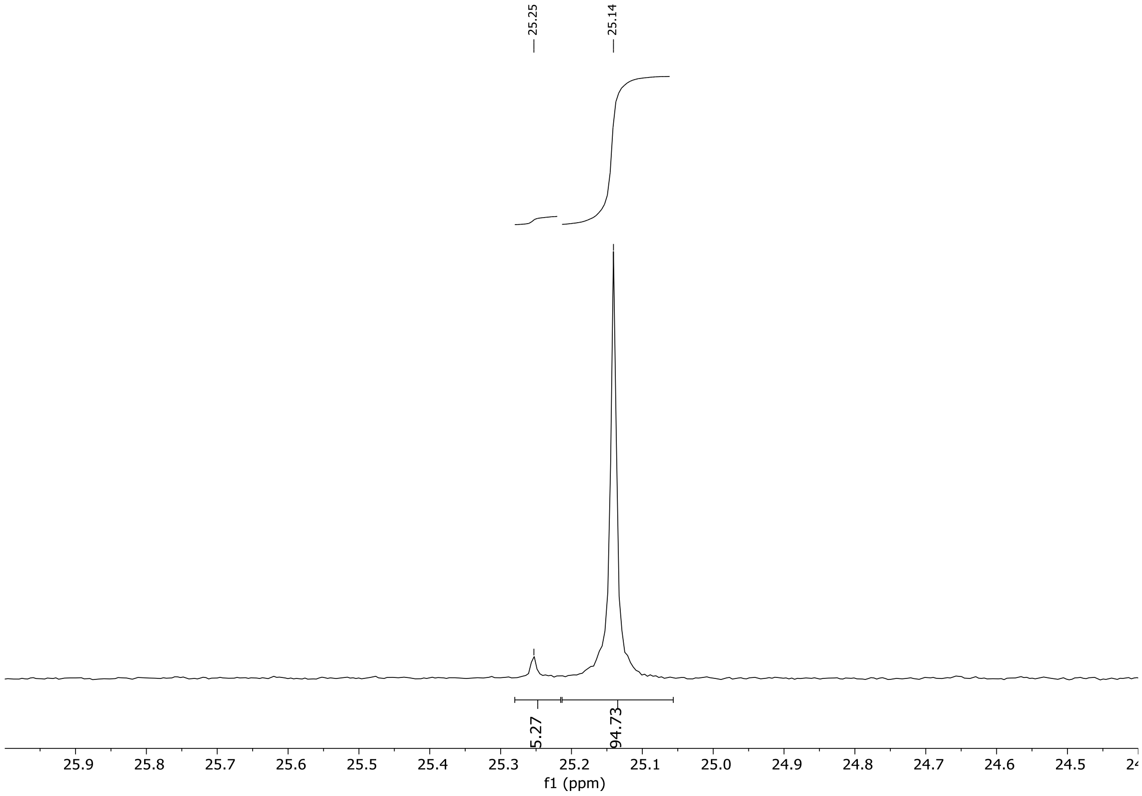

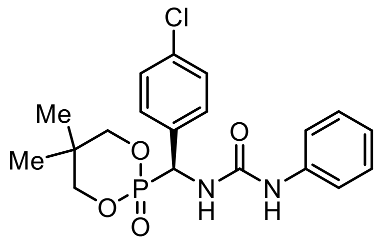

14

^{31}P-NMR (162 MHz, CDCl$_3$)

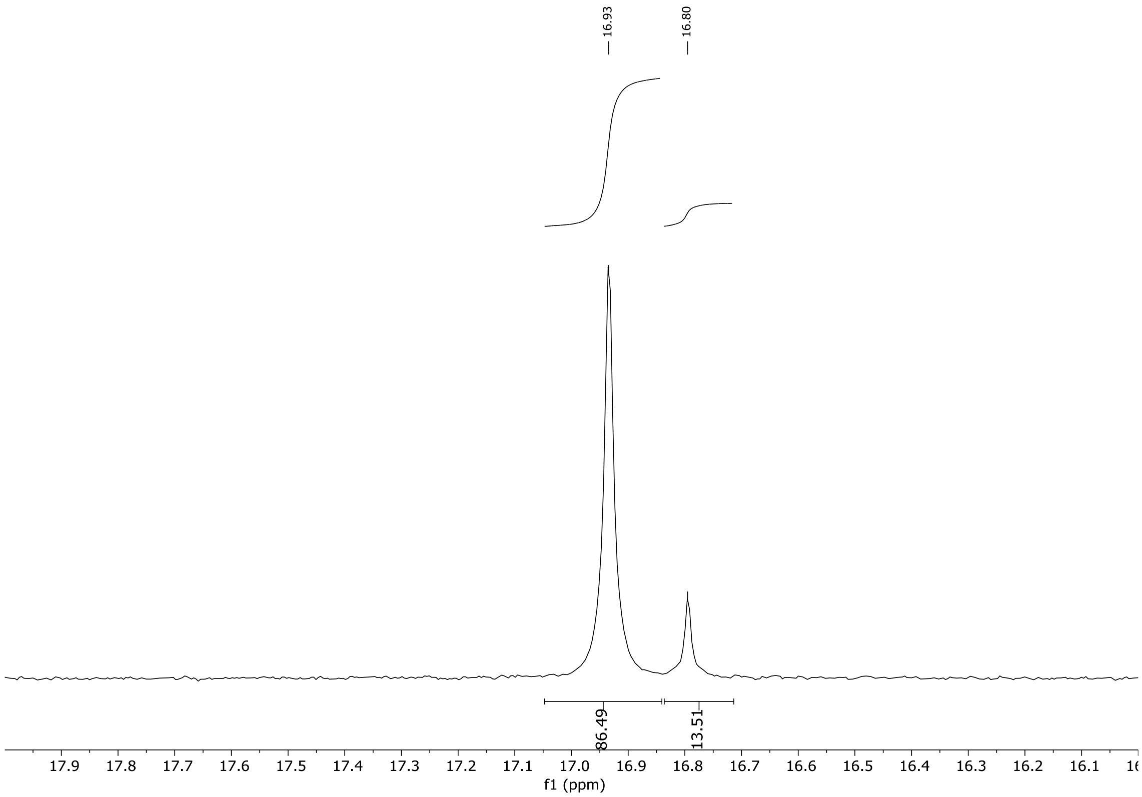

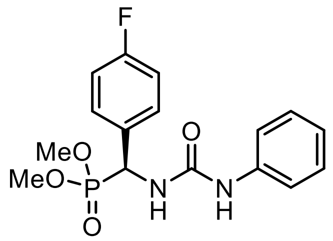

15

^{31}P-NMR (162 MHz, $CDCl_3$)

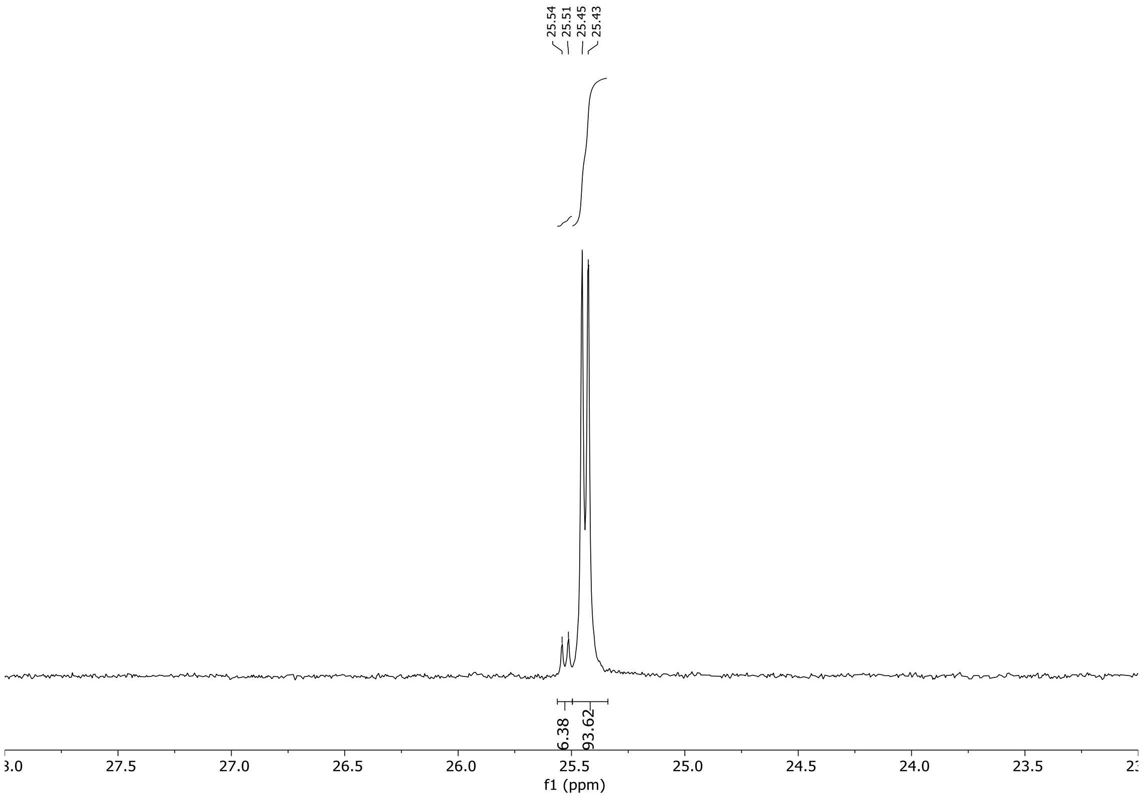

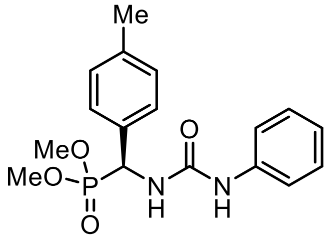

16

^{31}P-NMR (162 MHz, $CDCl_3$)

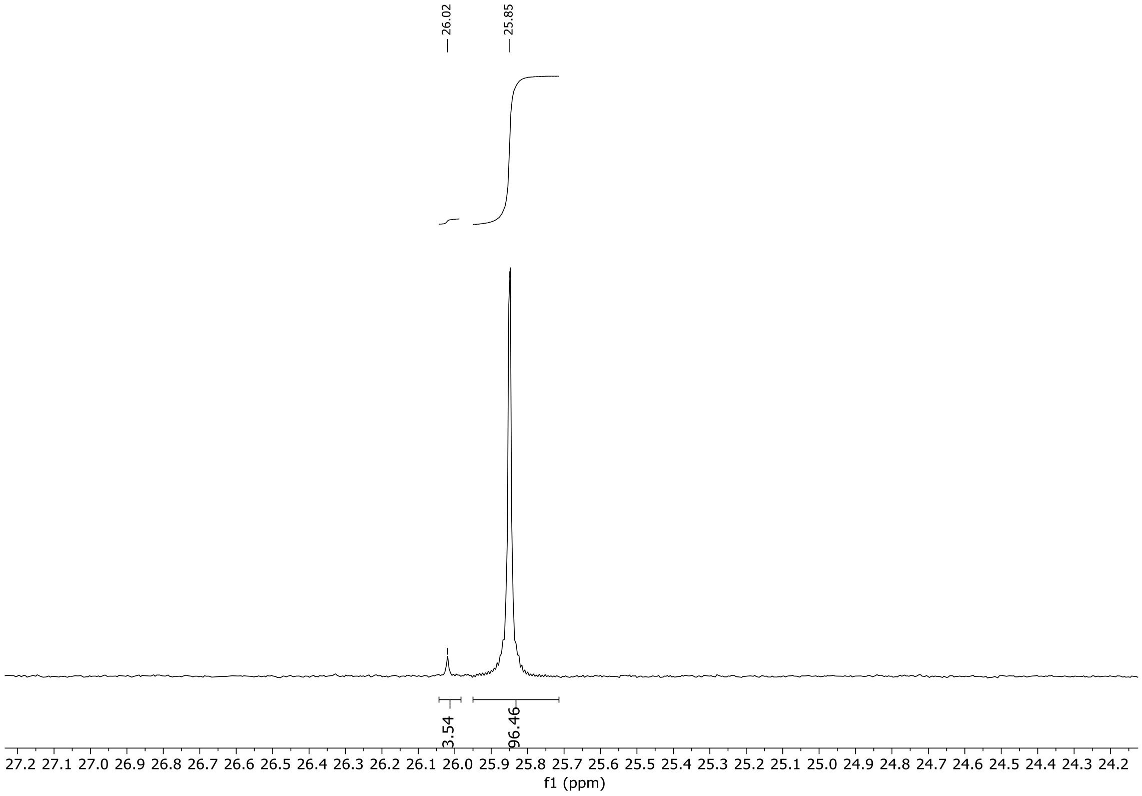

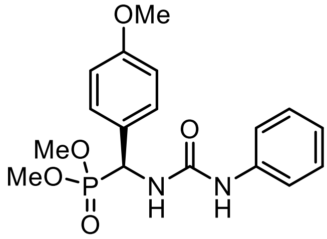

17

^{31}P-NMR (162 MHz, CDCl$_3$)

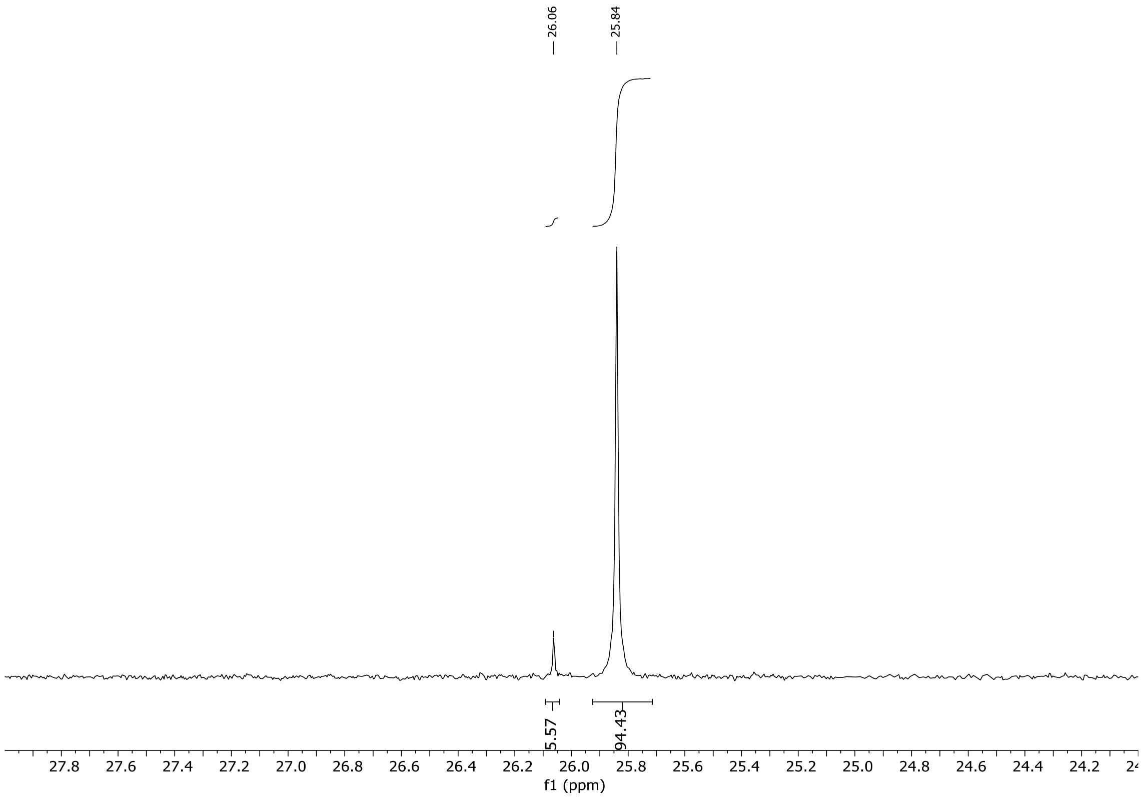

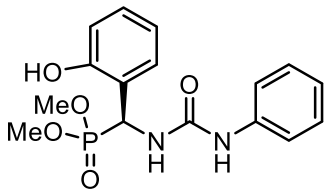

18

^{31}P-NMR (162 MHz, $CDCl_3$)

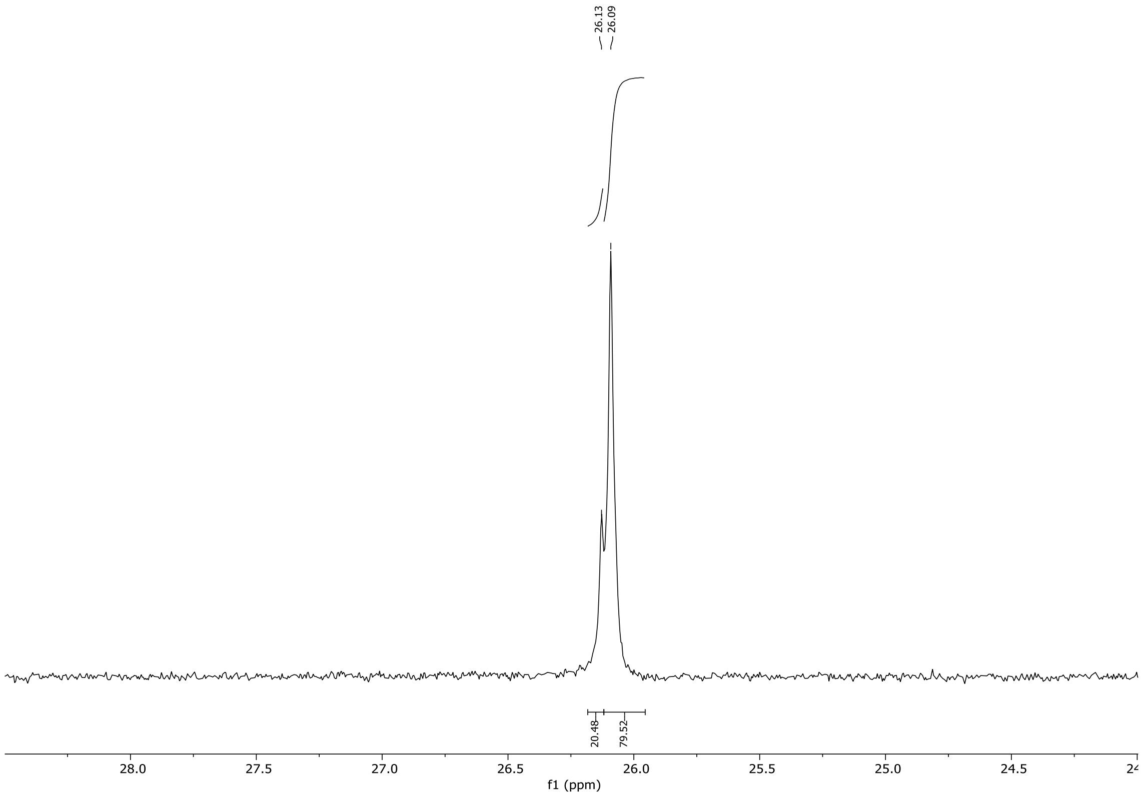

SFC chromatogram of racemic 18 (obtained by hydrophosphonylation of arylidene urea **S21** with dimethyl phosphite in absence of chiral catalyst)

Conditions: **SFC** Trefoil AMY1, CO_2:MeOH 97:3 → 50:50% in 4.5 min, 6 min, 1.8 mL/min, λ_{abs} = 238 nm, $t_R(R)$ = 2.9 min, $t_R(S)$ = 3.1 min

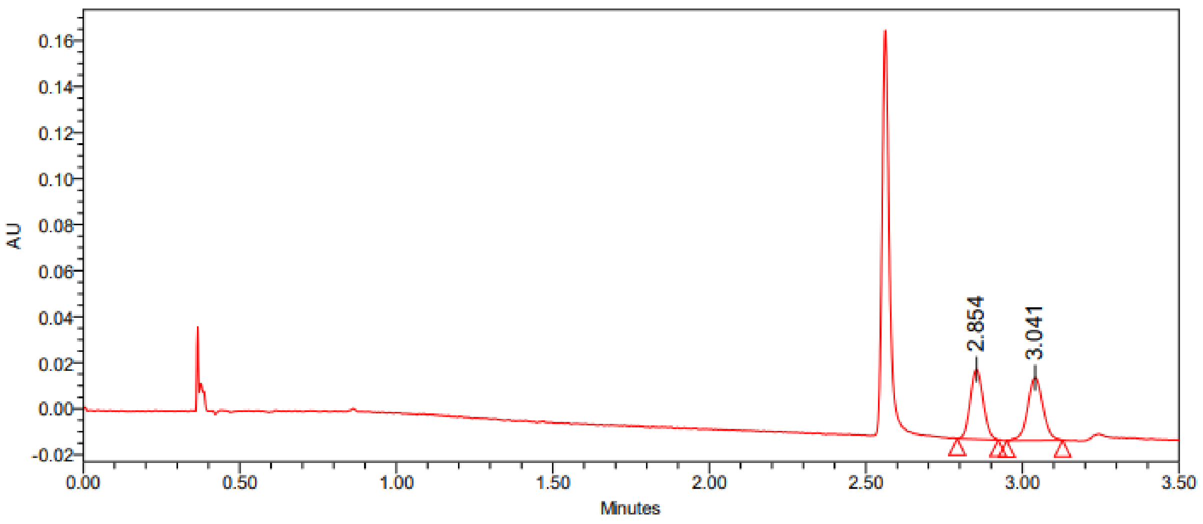

Peak Results

	Name	RT	Area	% Area	Height
1		2.854	87621	48.60	30464
2		3.041	92682	51.40	27337

SFC chromatogram of enantioenriched (*S*)-18

Conditions: **SFC** Trefoil AMY1, CO_2:MeOH 97:3 → 50:50% in 4.5 min, 6 min, 1.8 mL/min, λ_{abs} = 238 nm, $t_R(R)$ = 2.9 min, $t_R(S)$ = 3.1 min

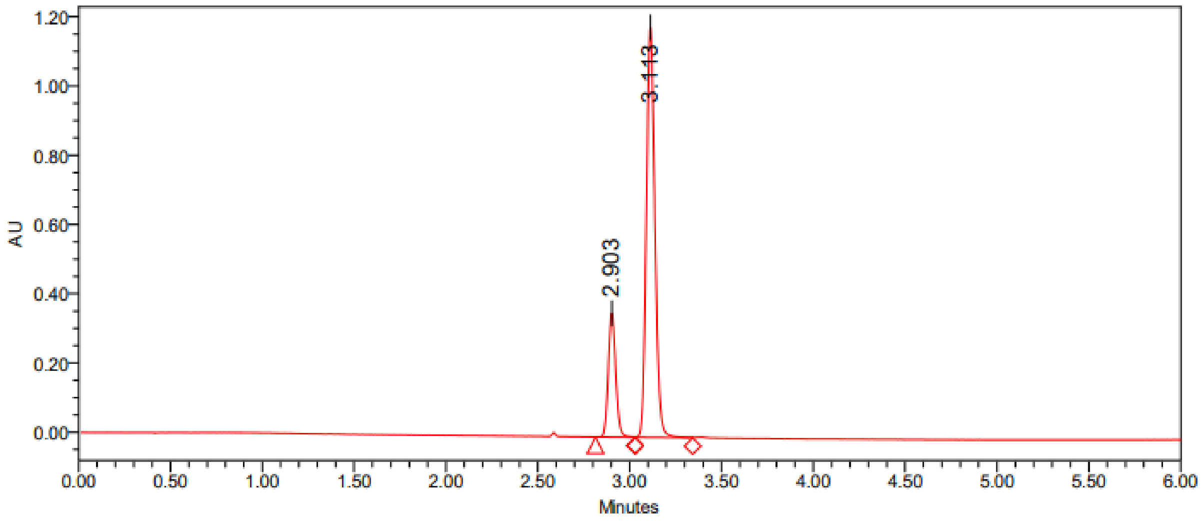

Peak Results

	Name	RT	Area	% Area	Height
1		2.903	1009844	20.87	358293
2		3.113	3827918	79.13	1184455

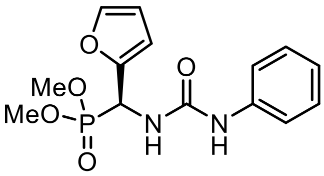

19

^{31}P-NMR (162 MHz, $CDCl_3$)

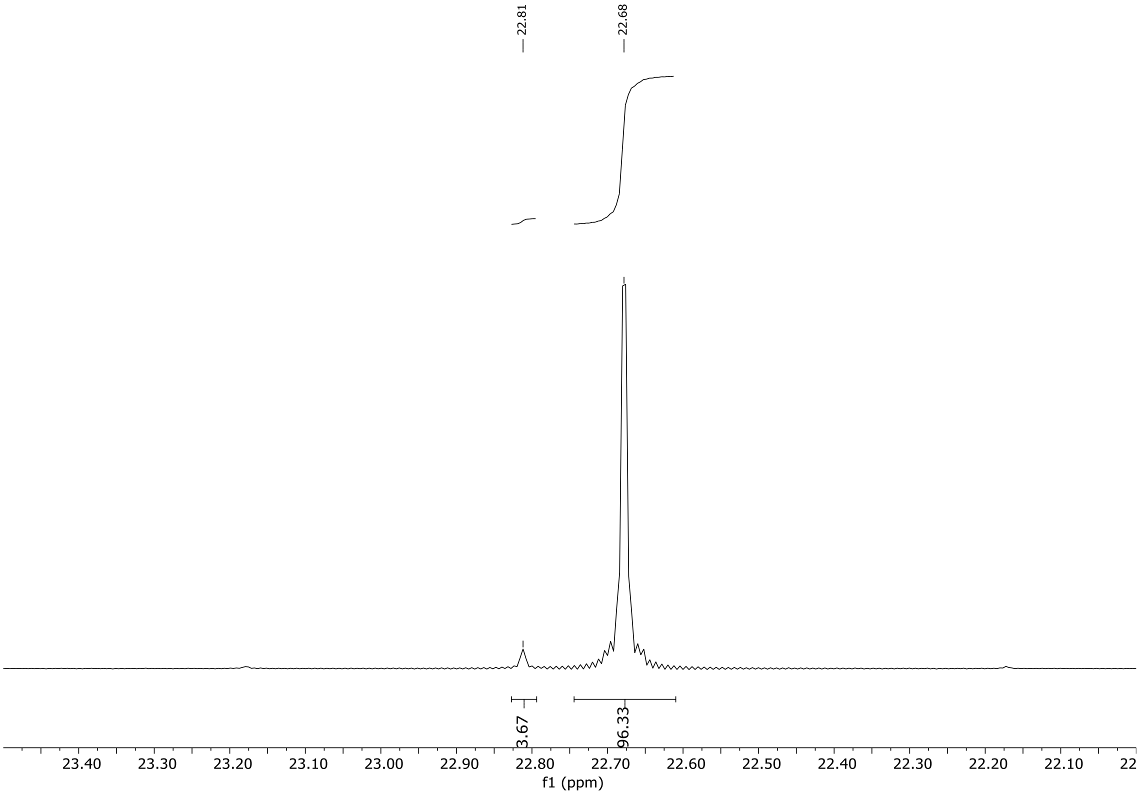

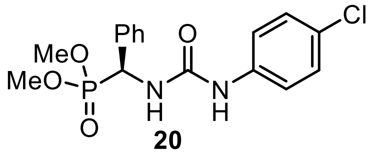

20

^{31}P-NMR (162 MHz, CDCl$_3$)

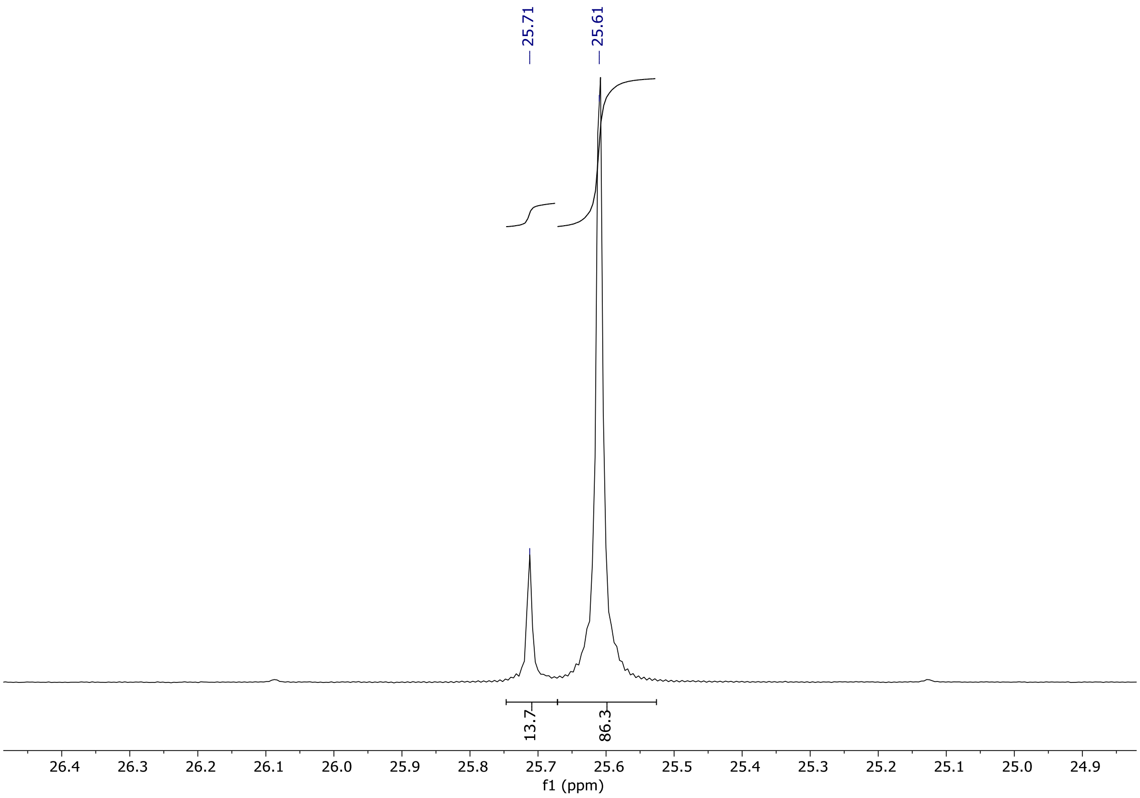

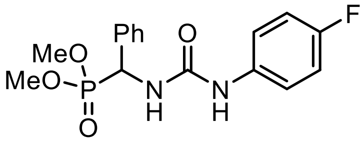

21

^{31}P-NMR (162 MHz, CDCl$_3$)

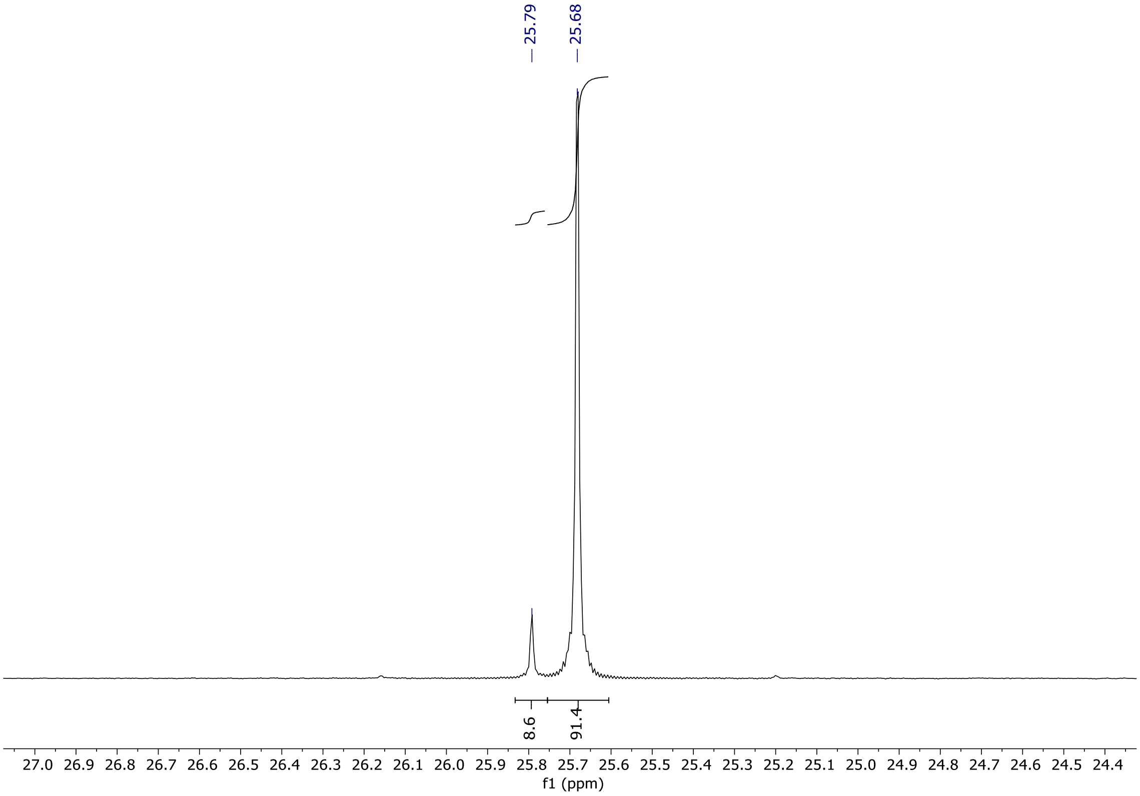

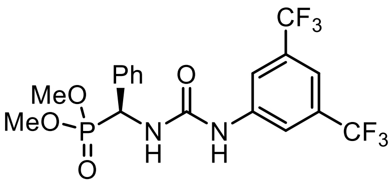

22

^{31}P-NMR (162 MHz, $CDCl_3$)

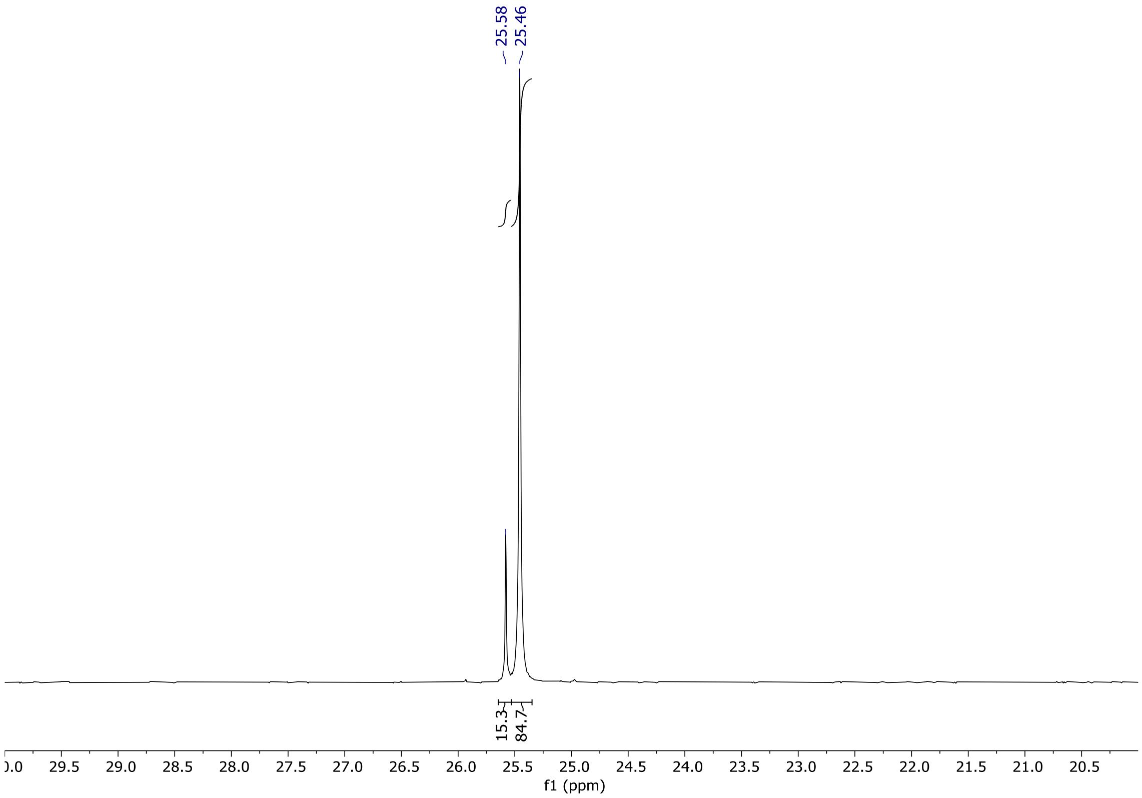

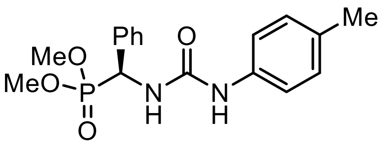

23

^{31}P-NMR (162 MHz, CDCl3)

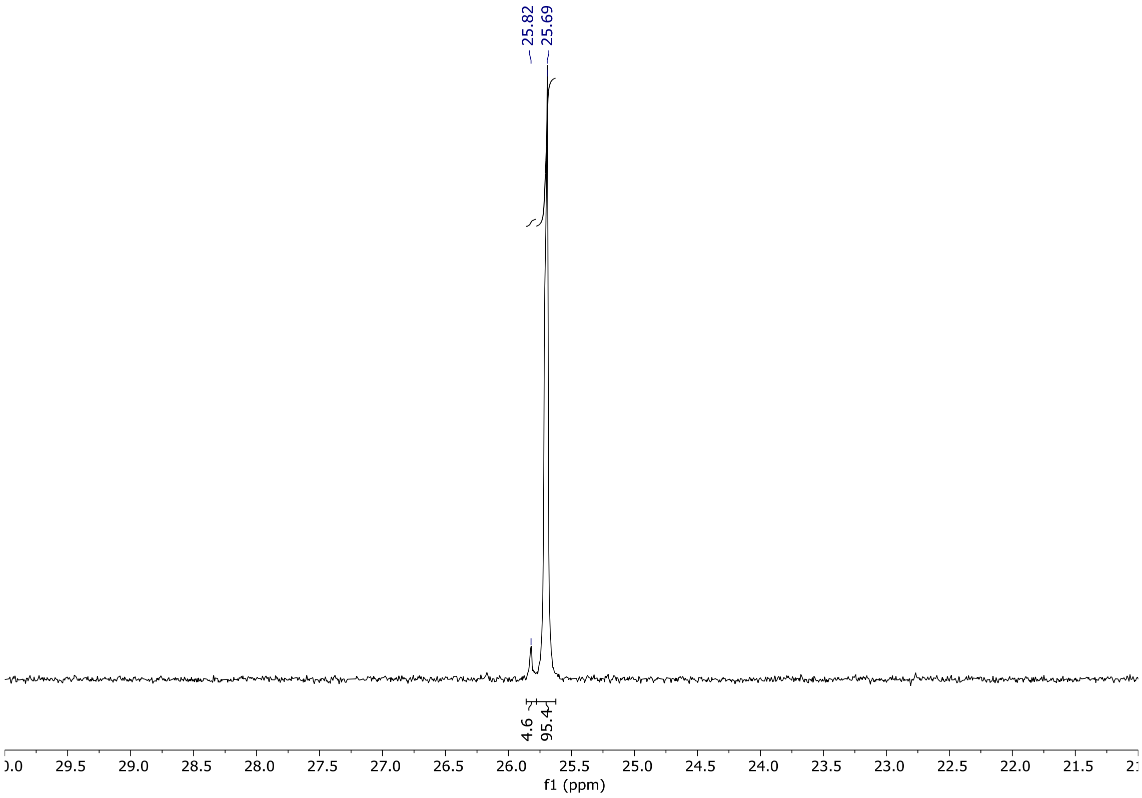

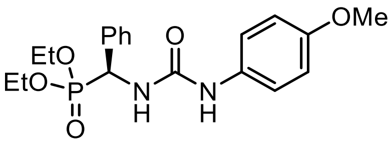

24

^{31}P-NMR (162 MHz, CDCl$_3$)

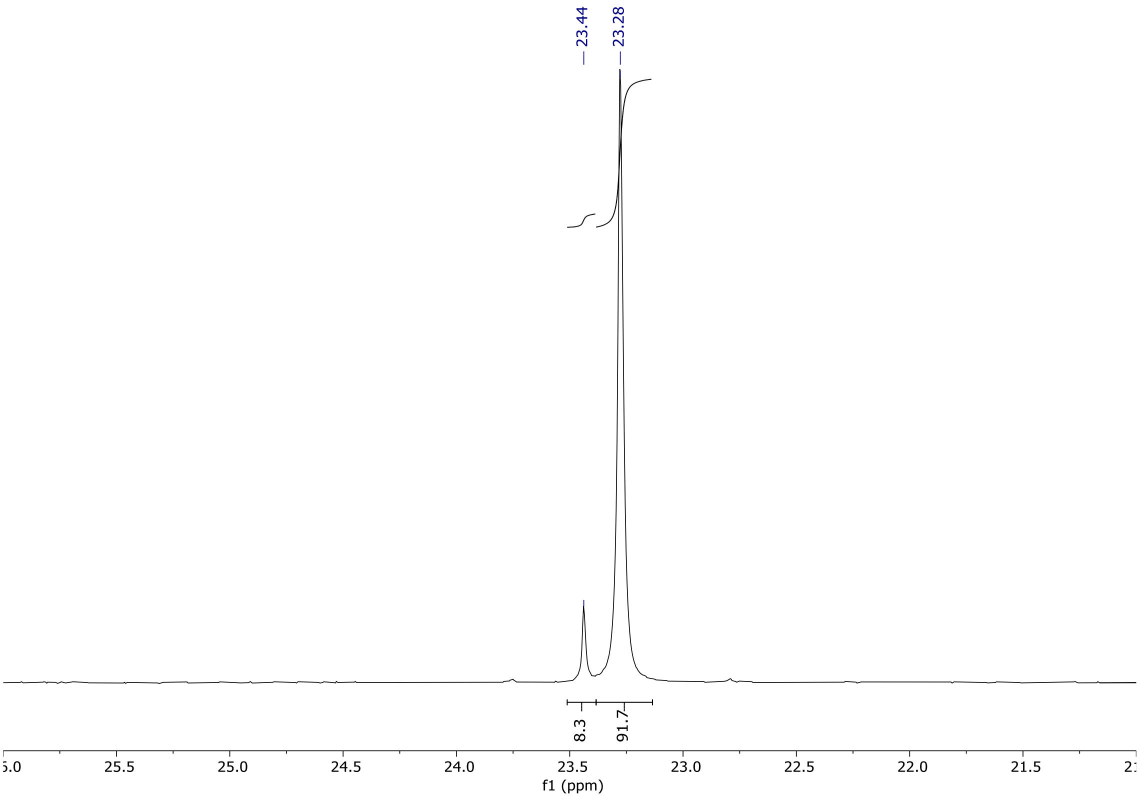

HPLC chromatogram of racemic 24 (obtained by mixing the *R* and *S* enantiomers)

Conditions: Chiralpak AY-H Lux 5µm Amylose-2, *n*-heptane/^{i}PrOH 90:10, 40 °C, 1 mL/min, λ_{abs}= 244 nm)

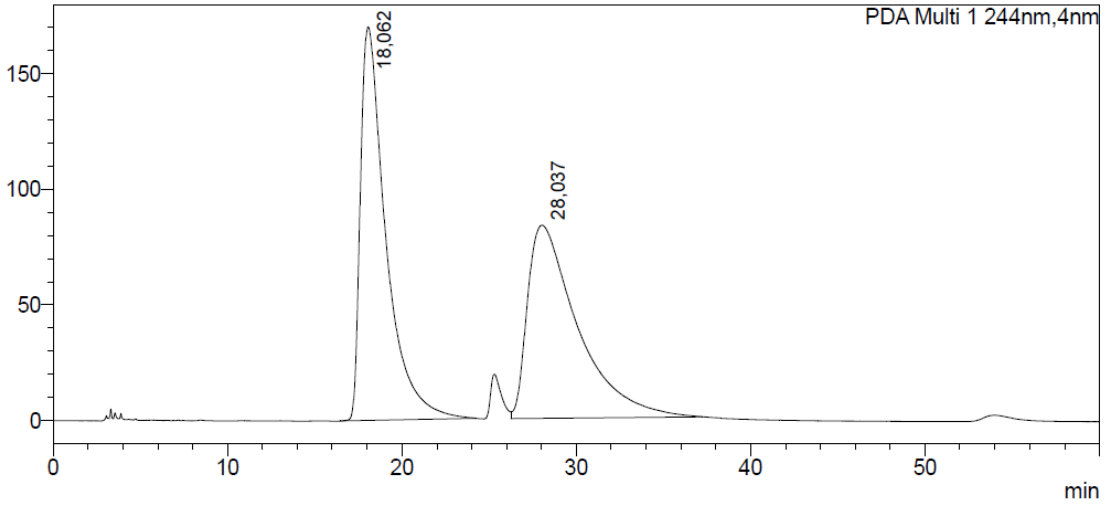

Peak#	Ret. Time	Area	Area%
1	18,062	17259362	50,774
2	28,037	16733468	49,226
Total		33992830	100,000

HPLC chromatogram of enantioenriched 24

Conditions: Chiralpak AY-H Lux 5µm Amylose-2, *n*-heptane/^{i}PrOH 90:10, 40 °C, 1 mL/min, λ_{abs}= 244 nm)

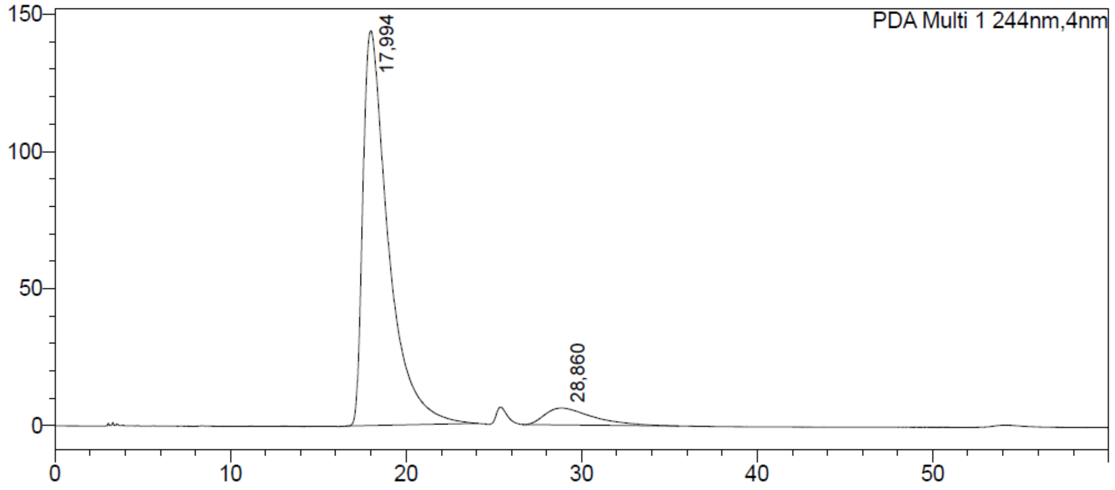

Peak#	Ret. Time	Area	Area%
1	17,994	14490438	92,568
2	28,860	1163456	7,432
Total		15653894	100,000

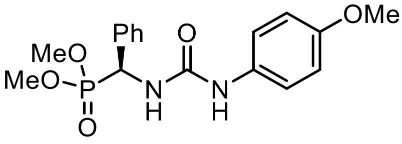

25

^{31}P-NMR (162 MHz, CDCl₃)

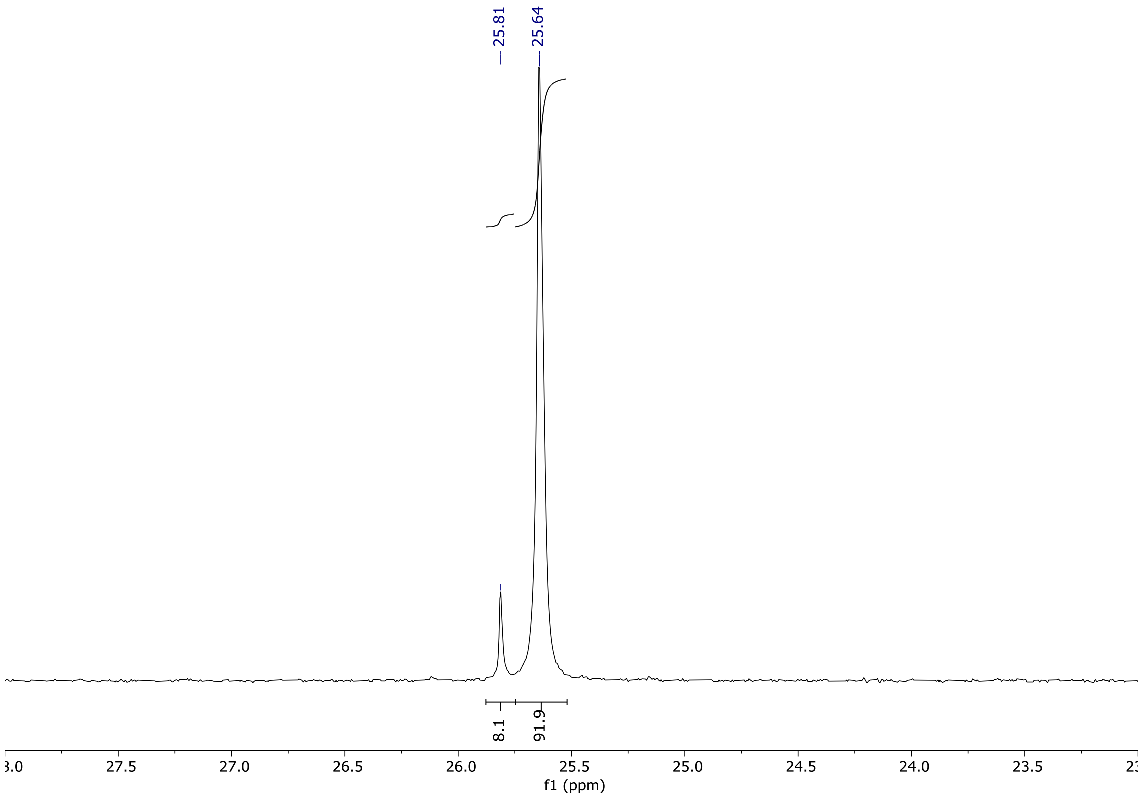

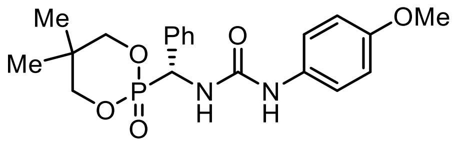

26

^{31}P-NMR (162 MHz, CDCl$_3$)

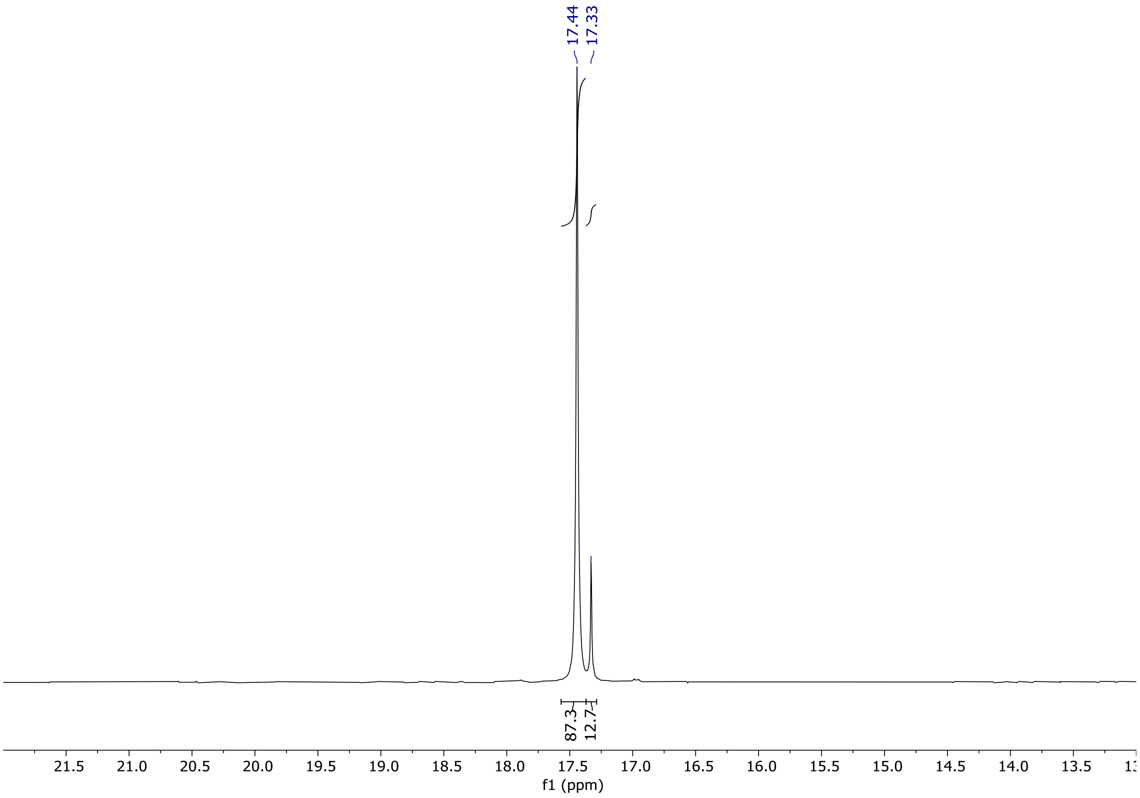

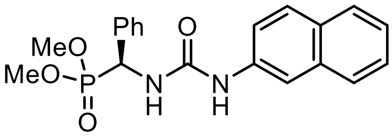

27

^{31}P-NMR (162 MHz, $CDCl_3$)

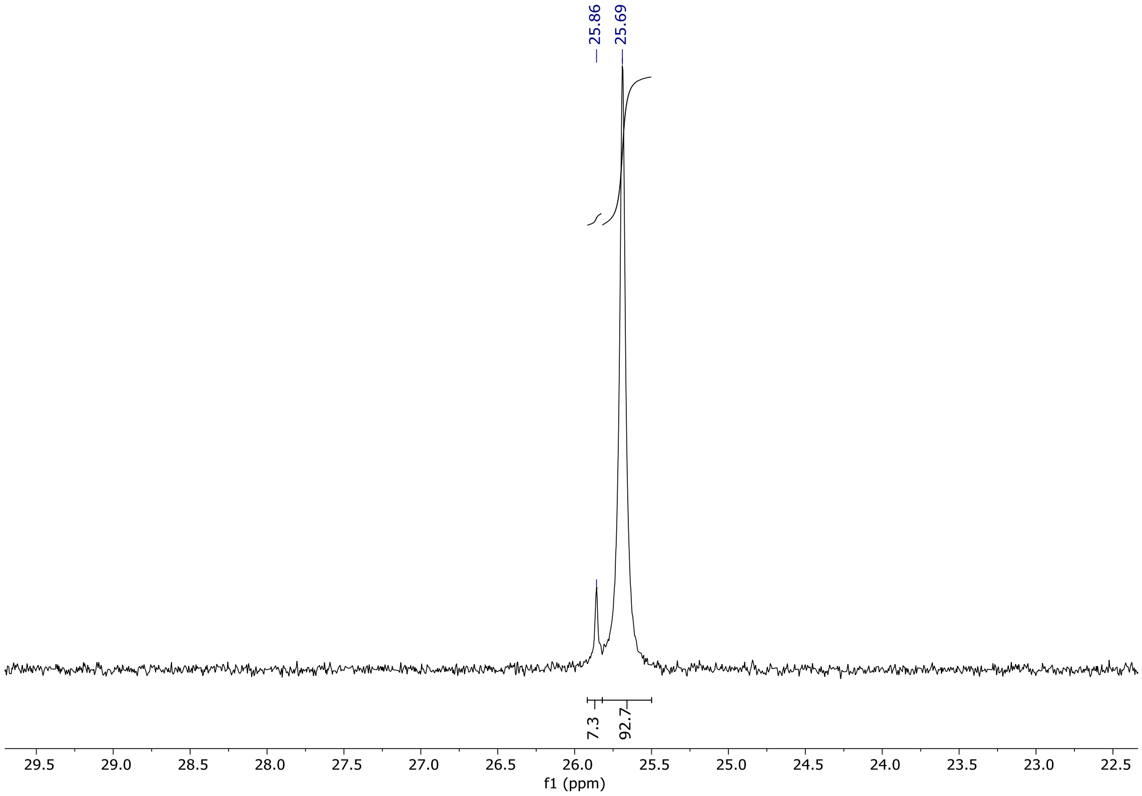

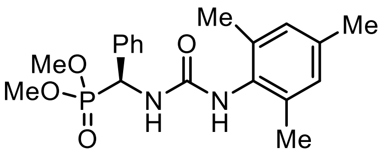

28

^{1}H-NMR (600 MHz, CD_2Cl_2)

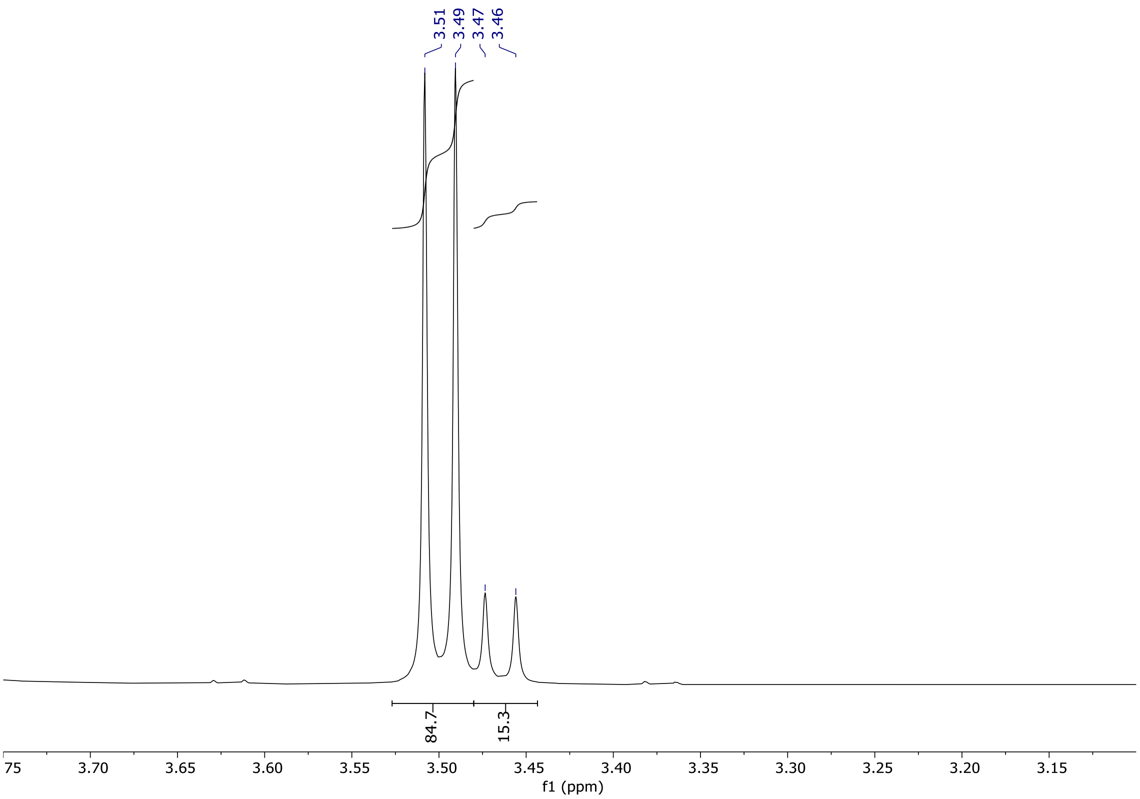

NOTE: Signal corresponding to one of the methyl groups of the dimethylphosphonate moiety.
